# Signaling pathway network alterations in human ovarian cancers identified with quantitative mitochondrial proteomics

**DOI:** 10.1007/s13167-019-00170-5

**Published:** 2019-06-08

**Authors:** Na Li, Xianquan Zhan

**Affiliations:** 1 Key Laboratory of Cancer Proteomics of Chinese Ministry of Health, Xiangya Hospital, Central South University, 87 Xiangya Road, Changsha, 410008 Hunan People’s Republic of China; 2 Hunan Engineering Laboratory for Structural Biology and Drug Design, Xiangya Hospital, Central South University, 87 Xiangya Road, Changsha, 410008 Hunan People’s Republic of China; 3 State Local Joint Engineering Laboratory for Anticancer Drugs, Xiangya Hospital, Central South University, 87 Xiangya Road, Changsha, 410008 Hunan People’s Republic of China; 4 National Clinical Research Center for Geriatric Disorders, Xiangya Hospital, Central South University, 88 Xiangya Road, Changsha, 410008 Hunan People’s Republic of China

**Keywords:** Ovarian cancer, Mitochondrial proteomics, Pathway networks, Hub molecules, Biomarkers, Predictive preventive personalized medicine

## Abstract

**Relevance:**

Molecular network changes are the hallmark of the pathogenesis of ovarian cancers (OCs). Network-based biomarkers benefit for the effective treatment of OC.

**Purpose:**

This study sought to identify key pathway–network alterations and network-based biomarkers for clarification of molecular mechanisms and treatment of OCs.

**Methods:**

Ingenuity Pathway Analysis (IPA) platform was used to mine signaling pathway networks with 1198 human tissue mitochondrial differentially expressed proteins (mtDEPs) and compared those pathway network changes between OCs and controls. The mtDEPs in important cancer-related pathway systems were further validated with qRT-PCR and Western blot in OC cell models. Moreover, integrative analysis of mtDEPs and Cancer Genome Atlas (TCGA) data from 419 patients was used to identify hub molecules with molecular complex detection method. Hub molecule–based survival analysis and multiple multivariate regression analysis were used to identify survival-related hub molecules and hub molecule signature model.

**Results:**

Pathway network analysis revealed 25 statistically significant networks, 192 canonical pathways, and 5 significant molecular/cellular function models. A total of 52 canonical pathways were activated or inhibited in cancer pathogenesis, including antigen presentation, mitochondrial dysfunction, GP6 signaling, EIF2 signaling, and glutathione-mediated detoxification. Of them, mtDEPs (TPM1, CALR, GSTP1, LYN, AKAP12, and CPT2) in those canonical pathway and molecular/cellular models were validated in OC cell models at the mRNA and protein levels. Moreover, 102 hub molecules were identified, and they were regulated by post-translational modifications and functioned in multiple biological processes. Of them, 62 hub molecules were individually significantly related to OC survival risk. Furthermore, multivariate regression analysis of 102 hub molecules identified significant seven hub molecule signature models (HIST1H2BK, ALB, RRAS2, HIBCH, EIF3E, RPS20, and RPL23A) to assess OC survival risks.

**Conclusion:**

These findings provided the overall signaling pathway network profiling of human OCs; offered scientific data to discover pathway network-based cancer biomarkers for diagnosis, prognosis, and treatment of OCs; and clarify accurate molecular mechanisms and therapeutic targets. These findings benefit for the discovery of effective and reliable biomarkers based on pathway networks for OC predictive and personalized medicine.

**Electronic supplementary material:**

The online version of this article (10.1007/s13167-019-00170-5) contains supplementary material, which is available to authorized users.

## Introduction

Ovarian cancer (OC) is one of the gynecologic malignancies, with high morbility and mortality, and is often diagnosed at its late stage [1]. It is a challenge to develop novel molecular biomarkers related to the early diagnosis, treatment, and prognosis in OC personalized medicine [2]. However, OC is a multifaceted disease that involved multifactors, complex biological processes, and unpredictable consequences [3]. It is not reasonable for a single molecule as biomarker for accurate predictive, preventive, and personalized medicine (PPPM) practice [2]. Numerous molecule alterations at different levels, including DNAs (genome), RNAs (transcriptome), proteins (proteome), and metabolites (metabolome), are involved in development and progression of OC and enriched in different pathway network systems [4]. Recent research showed that proteoform diversity made ones think about more complex tumorigenesis [5]. Molecular pathways and networks could help one understand the interactome from a view point of systematic biology [6]. Therefore, recognition of integrated molecular network variations would identify key biomarkers and therapeutic targets and benefit potential molecular mechanisms of OC toward personalized medicine.

High-throughput proteomics offers a promising approach to identify effective cancer biomarkers [7]. Especially, subcellular proteomics is able to directly investigate the targeted organelle that is associated with the specific biological functions [8]. Mitochondria, as a ubiquitous subcellular organelle, not only provide cell energy, but also are the key links of metabolism, signaling, cellular differentiation, cell cycle, and cell death in eukaryotic cells [9]. Mitochondrial pathways are involved in many diseases, including malignant tumors, diabetes, Parkinson’s disease, Alzheimer’s disease, and cardiovascular disease [10, 11]. Thus, mitochondrial proteomics has become a hotspot in the study of disease to discover new biomarkers and molecular targets for drug discovery and therapeutic intervention in recent years [12]. Our previous studies identified 5115 mitochondrial expressed proteins (mtEPs) and 70 statistically significant KEGG pathways based on 5115 mtEPs in human OCs [13], which provided the mitochondrial protein expression profiling and overall pathway profiling in human OC. Furthermore, we identified 1198 mitochondrial differentially expressed proteins (mtDEPs) in human OCs compared to controls and revealed the changes of energy metabolism pathway system including glycolysis, Kreb’s cycle, and oxidative phosphorylation [13, 14]. In fact, besides energy metabolism pathway system, 1198 mtDEP-involved pathway networks are very complex. It is necessary to completely and comprehensively investigate the entire pathway networks involved those 1198 mtDEPs to identify key pathway networks and hub molecules for human OC.

Ingenuity Pathway Analysis (IPA) (http://www.ingenuity.com) is a popular and extensively used pathway network analysis platform to mine statistically significant canonical pathways, network, and network-based hub molecules from differentially expressed proteomic data [15]. The “hub molecules” with many interaction partners might be assigned to specific biological function in the interaction network, with inherent characteristics such as (i) tend to be composed of many distinct or repetitive structural domains, (ii) tend to be signal transduction and transcription, and (iii) multiple post-translational modifications (PTMs) [16]. Molecular Complex Detection (MCODE) plugin is a reliable method to distinguish hub molecules from non-hub molecules [17]. Furthermore, integrative analysis of functions, co-expression, chromosome location, subcellular localizations, and prognostic values of hub molecules may play significant roles in the diagnosis and treatment of OC.

This study used IPA pathway analysis platform to reconstruct and compare biological pathways and networks based on our previous identified 1198 mtDEPs in OCs compared to controls; used MCODE plugin to identify hub molecules; and comprehensively analyzed the functions, co-expression, chromosome location, subcelluar locations, and prognostic values of hub molecules. Additionally, the prognostic model of multiple hub molecule signature was constructed with multivariable regression method to predict risk score in OC patients. These results provide scientific data to further classify the molecular mechanisms of OC and discover key signaling pathway network and pathway network-based molecule panel biomarkers for OCs.

## Materials and methods

### OC tissue specimen

A total of 18 ovarian tissue samples including seven epithelial OC tissues (high-degrade, poorly or moderately differentiated carcinoma cells) and 11 control ovaries with benign gynecologic disease (fibroids, adenomyosis, ovary serous cystadenoma, cervical intraepithelial neoplasia, atypical hyperplasia of endometrium, and pelvic organ prolapse) were obtained from Department of Gynecology, Xiangya Hospital, Central South University, China. This study was approved by the Medical Ethics Committee of Xiangya Hospital, and the written informed consent was obtained from each patient. Both OC and control tissues were verified by histological analysis. Each tissue sample was immediately placed in liquid nitrogen and then stored at − 80 °C.

### Ovarian cancer mtDEP dataset

A total of 1198 mtDEPs dataset was obtained with iTRAQ quantitative proteomics analysis of mitochondrial samples that were isolated and purified from human epithelial OC and control ovary tissues [13, 14]. Briefly, ovarian tissue samples were fully minced in pieces and homogenized in mitochondrial isolation buffer that contained Nagarse, followed by differential centrifugation to obtain crude mitochondria. The crude mitochondria were further centrifuged with Nycodenz gradient to purify mitochondria. The purified mitochondria samples were verified by Western blot and electron microscopy. The prepared OC and control mitochondria were digested with trypsin. The tryptic peptides were labeled with iTRAQ reagents, fractionated with SCX chromatography. The iTRAQ-labeled peptides were mixed equally and were subjected to LC–MS/MS analysis on a Q Exactive mass spectrometer (Thermo Scientific). MS/MS spectra were acquired and used to search protein database with MASCOT engine (Matrix Science, London, UK; version 2.2), embedded into Proteome Discoverer 1.4. mtDEPs were determined with the intensity differences of iTRAQ reporter ions. The detailed experimental procedure was described in our previous studies [14, 18].

### Ingenuity Pathway Analysis

Ingenuity Pathway Analysis (IPA) software is a well-cited stand-alone bioinformatics analysis tool to analyze signaling pathways and molecular networks. For the mtDEP data, the Swiss-Prot accession numbers and the corresponding fold changes between OC and control ovaries were input to the IPA data upload workflow. The matched genes/molecules can be indexed automatically with IPA, and the output table contains detailed description of mapped gene/molecules. The input IDs (proteins and genes) were grouped into different categories, including the all IDs (all input IDs), unmapped IDs (no match is found in the IPA system), and mapped IDs (match the corresponding molecules and recognize the duplicate IDs). For the duplicate IDs, the identifier with the highest fold change or the first instance was used in the pathway analysis.

### Cell lines and cell culture

OC cell lines TOV-21G and SKOV3 cells and control cell line IOSE80 cells were purchased from Keibai Academy of Science (Nanjing, China). TOV-21G cells were cultured in RPMI-1640 medium, and SKOV3 and IOSE80 cells were cultured in DMEM medium (Corning, NY, USA), supplemented with 10% fetal bovine serum (FBS, Gibco). All these cells were maintained with 5% CO_2_ and atmosphere at 37 °C.

### RNA extraction and qRT-PCR analyses

Total RNAs from cell lines were extracted with TRizol reagent (Invitrogen) according to the manufacturer’s instructions. Total RNAs were reversely transcribed into cDNAs and then used to perform qRT-PCR with SYBR Premix ExTaq (TaKaRa). Beta-actin was used as an internal control for mRNA quantification.

### Western blotting

Equal amounts of proteins were separated by 10% SDSPAGE gels and transferred onto PVDF membranes. The proteins on the membrane were incubated with primary antibodies against TPM1, CALR, GSTP1, CPT2, and AKAP12 (1:500; Sangon) and LYN and β-actin (1:1000; Santa Cruz Biotechnology) at 4 °C overnight and then were incubated for 2 h with horseradish peroxidase-conjugated goat antirat secondary antibody (1:5000; Santa Cruz Biotechnology) at room temperature.

### Identification of hub molecules with molecular complex detection

To evaluate their interactive associations, all mtDEPs were mapped to the STRING database. Subsequently, the protein–protein interactions (PPIs) were analyzed by Cytoscape software (version 3.2.1; National Resource for Network Biology) to obtain the PPI network. The criteria of hub molecule searching were set as the molecular complex detection (MCODE) score > 6, and a statistically significant difference (*p* < 0.05). GO biological process (BP) of those hub molecules was analyzed with Cytoscape ClueGO (two-sided hypergeometric test, adjusted *P* value < 0.05 corrected with Benjamini–Hochberg). Chromosome location, cell location, post-translational modifications (PTM), and analysis of prognostic values for these hub molecules were performed by R package (https://www.r-project.org/), SysPTM (http://lifecenter.sgst.cn/SysPTM/), and Kaplan–Meier plotter (http://kmplot.com/private/index.php.p=home), respectively. Biomarkers that had been reported were checked by CooLGeN (http://ci.smu.edu.cn/CooLGeN/Home.php).

### TCGA data of OC patients

TCGA data portal provides a platform for researchers to search, download, and analyze datasets generated from TCGA database (http://cancergenome.nih.gov/). Level 3 RNA-seq V2 data were obtained from the TCGA data of 419 OC patients. The expression data of hub molecules were extracted to do co-expression analysis by RStudio.

### Statistical analysis

For the qPCR and Western blot analysis, each experiment was repeated at least three times; data were expressed as the mean ± SD of triplicates. The Student’s *t* test was used to assess differences between-group in vitro studies with a statistical significance (*P* < 0.05). Statistical analyses were performed using SPSS 13.0 (SPSS Inc.). A Benjamini–Hochberg was used to calculate the adjusted *p* value to determine the probability of the association between the genes in the experimental dataset and the canonical pathway in the IPA database. The level of statistical significance was set to *p* < 0.05. Activation *z* score was calculated to evaluate that the canonical pathway was activated or inhibited. If *z* score ≥ 2, it means that the canonical pathway was more likely to be activated. If *z* score ≤ − 2, it means that the canonical pathway was more likely to be inhibited. The hub genes were subjected to SPSS20 to perform multivariate regression analysis to calculate the regression coefficient for each gene with statistical significance of *p* < 0.05.

## Results

### Significant signaling pathways of human OC mined from OC mtDEP dataset

A total of 1198 mtDEPs between OCs and controls were identified with iTRAQ-based quantitative proteomics (Supplementary Table 1), and of them, 1174 identifiers were mapped with IPA to the corresponding molecules (genes; proteins) (Supplementary Table 1) for IPA pathway network analysis. Each identifier was annotated with a Swiss-Prot accession number, gene name, protein name, subcellular location, family, and potential targets of drugs. Based on those 1174 identifiers, a total of 192 statistically significant canonical pathways (*p* < 0.05) were identified to involve the identified mtDEPs (Supplementary Table 2). Those 192 statistically significant canonical pathways were further filtered by |*z*| score ≥ 2; 52 statistically significant canonical pathways might be activated or inhibited (Supplementary Table 2). Here, the parameter of *z* score represents signaling pathways were activated (*z* score ≥ 2) or inhibited (*z* score ≤ − 2). Further analysis of the relationships between those 52 activated or inhibited canonical pathways and cancer biology revealed 29 cancer-related pathways (Supplementary Figure 1), with the detailed information on those 29 cancer-related canonical pathways (*p* < 0.05; |*z*| ≥ 2). The important tumor-associated pathways were antigen presentation pathway (Supplementary Figure 1 item 1; Table 1), mitochondrial dysfunction (Supplementary Figure 1 item 2; Table 1), GP6 signaling pathway (Supplementary Figure 1 item 3; Table 1), EIF2 signaling (Supplementary Figure 1 item 4; Table 1), and glutathione-mediated detoxification (Supplementary Figure 1 item 5; Table 1).

**Table 1:** The important tumor-associated canonical pathways include antigen presentation pathway, mitochondrial dysfunction, GP6 signaling pathway, EIF2 signaling, and glutathione-mediated detoxification

Pathway name	Protein	Coverage (%)	Unique peptides	PSMs	MW (kDa)	Calc. pI	Ratio (T/N)	*p* value (*t* test)
Antigen presentation pathway	HLA-A protein (fragment) (J7RE54_HUMAN)	51.28	1	19	31.60	5.67	1.70	5.24E−03
	MHC class I antigen (fragment) (D0W033_HUMAN)	37.73	1	12	31.57	5.94	2.69	4.15E−03
	MHC class I antigen (fragment) (A0A0K0KRA3_HUMAN)	47.51	1	11	21.19	7.02	4.12	5.31E−04
	MHC class I antigen (fragment) (A0A0K0KSD4_HUMAN)	48.62	1	13	21.22	7.87	1.82	5.58E−03
	MHC class I antigen (fragment) (E3SWK8_HUMAN)	53.04	1	15	21.12	7.37	2.21	2.77E−02
	MHC class II antigen (fragment) (Q67AU1_HUMAN)	31.18	1	4	10.84	5.10	3.58	6.74E−03
	MHC class II antigen (fragment) (A0A0S2C388_HUMAN)	25.4	5	7	28.68	7.43	2.01	1.13E−02
	MHC class II antigen (fragment) (S6AP35_HUMAN)	45.08	1	21	41.16	6.62	1.58	4.23E−02
	MHC class II antigen (fragment) (A0A142L067_HUMAN)	24.11	3	5	25.87	7.15	1.67	1.55E−02
	HLA class II histocompatibility antigen, DR alpha chain (A0A0G2JMH6_HUMAN)	32.68	2	47	28.60	5.00	1.82	8.26E−04
	MHC class II antigen (fragment) (N1NSB5_HUMAN)	52.81	2	10	10.89	6.52	1.84	1.55E−03
	HLA-DRB5 protein (fragment) (A0A024F8S6_HUMAN)	42.48	1	23	25.73	6.52	0.38	2.40E−04
	MHC class Ib antigen (fragment) (I3RW89_HUMAN)	25.42	3	11	33.78	5.40	1.60	3.08E−02
	Protein disulfide-isomerase A3 (PDIA3_HUMAN)	70.89	27	324	56.75	6.35	1.71	4.04E−03
	Protein disulfide-isomerase A3 (fragment) (H7BZJ3_HUMAN)	74.8	1	58	13.51	7.30	1.87	1.35E−03
	Calreticulin variant (fragment) (Q53G71_HUMAN)	62.32	21	156	46.89	4.45	1.98	1.19E−02
	Antigen peptide transporter 2 (X5CMH5_HUMAN)	28.31	17	36	77.65	7.85	1.60	1.20E−03
Mitochondrial dysfunction	Cytoplasmic aconitate hydratase (ACOC_HUMAN)	13.27	10	13	98.34	6.68	0.65	6.04E−03
	Apoptosis-inducing factor 1, mitochondrial (AIFM1_HUMAN)	46.66	24	158	66.86	8.95	1.59	2.76E−03
	ATP synthase mitochondrial F1 complex assembly factor 1 (ATPF1_HUMAN)	29.57	8	17	36.41	7.96	1.90	9.90E−03
	ATP synthase mitochondrial F1 complex assembly factor 2 (ATPF2_HUMAN)	21.45	5	10	32.75	7.09	1.54	1.57E−04
	B cell CLL/lymphoma 2, isoform CRA_b (A0A024R2C4_HUMAN)	4.88	1	1	22.32	6.96	0.31	3.30E−05
	Cytochrome c oxidase copper chaperone (fragment) (H7C4E5_HUMAN)	12.07	1	1	6.41	7.69	2.75	2.33E−03
	Cytochrome c oxidase subunit 4 isoform 1, mitochondrial (COX41_HUMAN)	36.09	7	48	19.56	9.51	1.52	4.37E−03
	Cytochrome c oxidase subunit 4 isoform 2, mitochondrial (COX42_HUMAN)	5.85	1	1	20.00	9.63	0.21	6.28E−04
	Cytochrome c oxidase subunit 6C (COX6C_HUMAN)	52	7	30	8.78	10.39	1.54	5.97E−03
	Cytochrome c oxidase subunit 7A2, mitochondrial (CX7A2_HUMAN)	27.71	2	15	9.39	9.76	1.55	3.89E−02
	Cytochrome c oxidase subunit 7A-related protein, mitochondrial (COX7R_HUMAN)	54.39	4	8	12.61	9.42	1.88	5.33E−03
	Cytochrome c (fragment) (C9JFR7_HUMAN)	57.43	6	42	11.33	9.66	2.43	1.11E−03
	Glutathione peroxidase 7 (GPX7_HUMAN)	33.16	5	9	20.98	8.27	1.66	2.77E−04
	Serine protease HTRA2, mitochondrial (HTRA2_HUMAN)	13.1	4	7	48.81	10.07	1.58	8.22E−03
	Amine oxidase (flavin-containing) B (AOFB_HUMAN)	43.46	18	143	58.73	7.50	0.41	2.86E−04
	Thioredoxin, mitochondrial (THIOM_HUMAN)	36.75	3	18	18.37	8.29	1.52	5.11E−03
	Cytochrome b-c1 complex subunit 6, mitochondrial (QCR6_HUMAN)	51.65	5	18	10.73	4.44	1.59	1.63E−02
	Voltage-dependent anion-selective channel protein 1 (VDAC1_HUMAN)	81.27	17	431	30.75	8.54	1.55	4.97E−03
GP6 signaling pathway	Disintegrin and metalloproteinase domain-containing protein 10 (ADA10_HUMAN)	10.83	7	9	84.09	7.77	1.57	3.42E−04
EIF2 signaling	Collagen alpha-1(X) chain (COAA1_HUMAN)	1.91	1	1	66.12	9.67	0.24	2.71E−04
	Collagen alpha-1(XVI) chain (COGA1_HUMAN)	1.81	3	4	157.65	7.84	0.43	7.50E−04
	Collagen alpha-1(I) chain (CO1A1_HUMAN)	14.96	7	87	138.86	5.80	0.30	8.28E−05
	Collagen, type I, alpha 1, isoform CRA_a (D3DTX7_HUMAN)	18.53	2	61	84.69	6.24	0.36	5.47E−05
	Collagen alpha-2(I) chain (A0A087WTA8_HUMAN)	13.27	15	55	129.07	9.01	0.29	1.84E−03
	Collagen alpha-1(II) chain (CO2A1_HUMAN)	3.03	3	3	141.70	6.92	0.17	7.01E−04
	Collagen alpha-1(III) chain (CO3A1_HUMAN)	6.41	9	21	138.48	6.61	0.30	6.85E−04
	Collagen alpha-2(IV) chain (CO4A2_HUMAN)	6.43	7	18	167.45	8.66	0.39	4.18E−03
	Collagen, type V, alpha 2, isoform CRA_b (D3DPH5_HUMAN)	2.14	2	2	86.03	6.06	0.24	3.59E−03
	Collagen alpha-1(VI) chain (CO6A1_HUMAN)	32.78	27	57	108.46	5.43	0.40	1.54E−03
	Collagen alpha-2(VI) chain (CO6A2_HUMAN)	18.84	18	50	108.51	6.21	0.44	5.27E−04
	Collagen alpha-3(VI) chain (E7ENL6_HUMAN)	31.1	1	165	277.95	8.18	0.37	4.07E−04
	Collagen alpha-6(VI) chain (CO6A6_HUMAN)	0.71	1	2	247.02	6.89	0.40	2.11E−03
	Collagen alpha-1(VIII) chain (CO8A1_HUMAN)	4.84	3	8	73.32	9.61	0.33	9.61E−04
	Inositol 1,4,5-trisphosphate receptor type 1 (ITPR1_HUMAN)	12.8	23	48	313.73	6.04	0.59	6.55E−05
	Laminin subunit alpha-2 (A0A087WYF1_HUMAN)	3.85	10	12	343.20	6.37	0.58	3.10E−03
	Laminin subunit gamma-2 (LAMC2_HUMAN)	4.02	4	4	130.89	6.19	1.60	4.91E−03
	Protein kinase C (A0A169TED2_HUMAN)	11.61	6	10	76.64	7.47	0.57	4.56E−03
	B cell CLL/lymphoma 2, isoform CRA_b (A0A024R2C4_HUMAN)	4.88	1	1	22.32	6.96	0.31	3.30E−05
	Eukaryotic translation initiation factor 3 subunit C (B4E2Z6_HUMAN)	19.52	11	20	90.48	5.71	0.62	6.44E−04
	Eukaryotic translation initiation factor 3 subunit D (EIF3D_HUMAN)	12.77	5	10	63.93	6.05	0.64	3.21E−03
	Eukaryotic translation initiation factor 3 subunit E (EIF3E_HUMAN)	26.29	11	18	52.19	6.04	0.65	1.78E−04
	Eukaryotic translation initiation factor 3 subunit F (B4DMT5_HUMAN)	31.27	8	14	33.22	5.59	0.64	1.04E−03
	Eukaryotic translation initiation factor 3 subunit G (fragment) (K7ER90_HUMAN)	9.25	2	2	25.43	5.49	0.61	7.81E−04
	Eukaryotic translation initiation factor 3 subunit H (A0A087WZK9_HUMAN)	22.92	6	9	39.56	6.39	0.58	3.64E−03
	Eukaryotic translation initiation factor 3 subunit I (EIF3I_HUMAN)	17.23	6	9	36.48	5.64	0.65	1.56E−03
	Eukaryotic translation initiation factor 3 subunit K (K7ES31_HUMAN)	20.44	2	4	15.86	6.74	0.65	5.63E−03
	Eukaryotic translation initiation factor 3 subunit L (B4DQF6_HUMAN)	6.97	4	7	62.65	6.16	0.63	1.71E−03
	Eukaryotic initiation factor 4A-II (E7EQG2_HUMAN)	44.48	6	32	41.26	5.64	0.62	1.08E−02
	40S ribosomal protein S30 (RS30_HUMAN)	16.95	1	2	6.64	12.15	0.53	3.97E−03
	78 kDa glucose-regulated protein (GRP78_HUMAN)	56.73	35	376	72.29	5.16	1.77	1.87E−04
	Polypyrimidine tract binding protein 1, isoform CRA_b (A6NLN1_HUMAN)	12.71	5	14	56.48	9.38	0.50	9.42E−04
	60S ribosomal protein L12 (RL12_HUMAN)	44.85	5	12	17.81	9.42	0.57	1.62E−03
	60S ribosomal protein L23a (RL23A_HUMAN)	31.41	5	13	17.68	10.45	0.65	7.46E−03
	60S ribosomal protein L9 (RL9_HUMAN)	39.58	7	20	21.85	9.95	0.64	2.02E−03
	RPLP1 protein (Q6FG99_HUMAN)	44.74	2	8	11.56	4.37	0.56	1.05E−03
	40S ribosomal protein S12 (RS12_HUMAN)	53.79	7	21	14.51	7.21	0.65	1.54E−03
	40S ribosomal protein S19 (RS19_HUMAN)	39.31	6	25	16.05	10.32	0.56	3.77E−04
	40S ribosomal protein S20 (RS20_HUMAN)	25.21	3	10	13.36	9.94	0.64	5.19E−05
	40S ribosomal protein S21 (Q8WVC2_HUMAN)	12.35	1	3	8.84	8.50	0.63	3.09E−03
	40S ribosomal protein S28 (RS28_HUMAN)	46.38	3	8	7.84	10.70	0.54	5.04E−03
	40S ribosomal protein S29 (A0A087WTT6_HUMAN)	13.21	1	2	6.11	9.14	0.66	3.27E−03
	40S ribosomal protein S3 (RS3_HUMAN)	65.43	15	46	26.67	9.66	0.62	5.61E−05
	Ras-related protein R-Ras (RRAS_HUMAN)	29.36	4	11	23.47	6.93	0.64	1.88E−02
	Ras-related protein R-Ras2 (RRAS2_HUMAN)	18.63	2	7	23.38	6.01	0.66	6.54E−03
Glutathione-mediated detoxification	Glutathione *S*-transferase kappa 1 (GSTK1_HUMAN)	66.37	13	129	25.48	8.41	2.13	4.24E−07
	Glutathione *S*-transferase Mu 1 (GSTM1_HUMAN)	48.17	3	24	25.70	6.70	0.46	6.84E−04
	Glutathione *S*-transferase Mu 2 (GSTM2_HUMAN)	61.47	8	31	25.73	6.37	0.49	4.40E−03
	Glutathione *S*-transferase Mu 3 (GSTM3_HUMAN)	52.44	4	43	26.54	5.54	0.37	5.16E−04
	Glutathione *S*-transferase Mu 4 (A0A0A0MR85_HUMAN)	26.61	1	14	25.55	5.90	0.59	8.21E−03
	Glutathione *S*-transferase Mu 5 (GSTM5_HUMAN)	36.7	2	25	25.66	7.39	0.59	4.01E−04
	Glutathione *S*-transferase pi (fragment) (C7DJS2_HUMAN)	42.38	1	14	16.66	5.10	2.02	3.78E−02
	Maleylacetoacetate isomerase (MAAI_HUMAN)	26.85	1	7	24.20	8.54	1.59	3.61E−03
	Maleylacetoacetate isomerase (G3V5T0_HUMAN)	28.71	1	7	22.60	7.69	2.07	8.20E−04

Along with the advancement in tumor immunology, the immune-checkpoint blockade therapy has been an important part in the mode of combined therapy of tumor. One of the most important immune checkpoint pathways has been applied between the PD-1 receptor expressed on activated T cells and its ligands, programmed death-1 ligand (PD-L1) and PD-L2. With the arrival of PD-1 in the domestic antitumor drug market, the hot topic of tumor immunotherapy appears frequently in people’s view. A large number of studies on immunotherapy combined with other therapies are also in full swing. Treatment approaches that target the PD-L1 have yielded objective responses in a subset of individuals with advanced carcinomas in some clinical trials [19]. The mtDEPs enriched in antigen presentation pathway (Supplementary Figure 1 item 1) indicated some potential antitumor-related immune molecules. Antigen presentation pathway describes a key immune process that is essential for T cell immune response triggering. Thus, the new biomarkers investigated in the antigen presentation pathway included CALR (fold change = 1.97, *p* = 0.013), HLA-A (fold change = 2.68, *p* = 0.004), HLA-C (fold change = 1.82, *p* = 0.005), HLA-DPA1 (fold change = 3.57, *p* = 0.006), HLA-DPB1 (fold change = 2.00, *p* = 0.011), HLA-DQB1 (fold change = 2.00, *p* = 0.011), HLA-DRA (fold change = 1.81, *p* = 0.0008), HLA-DRB1 (fold change = 1.66, *p* = 0.015), HLA-DRB5 (fold change = 0.38, *p* = 0.0002), HLA-E (fold change = 1.60, *p* = 0.030), PDIA3 (fold change = 1.87, *p* = 0.001), and TAP2 (fold change = 1.60, *p* = 0.001), which may represent novel therapeutic targets for the prediction and eventual improvement of the response to therapy in patients with OC. Some key molecules, such as CALR, HLA-A, HLA-DRB1, PDIA3, HLA-DQB1, HLA-E, and TAP2, have been implicated in various types of cancers and emphasized their important values related to the tumor progression [20]. Immunotherapy or immunotherapy combined with other therapies made the internal environment expose a vast array of antigens, so further understanding on the process of antigen presentation pathway is particularly important. Meanwhile, tumor immune-related pathways were also enriched, such as B cell development pathway, cd28 signaling in T Helper cell, chemokine signaling, IL-1 signaling, IL15 signaling, IL-2 signaling, IL-3 signaling, IL-4 signaling, and IL-8 signaling. Those enriched pathways indicated mitochondria play important roles in mediating tumor immune.

During the last decade, a great attention has also been paid to energy metabolic reprogramming of cancer. However, cancer basic studies fail to reach a consistent conclusion on mitochondrial function in cancer energy metabolism [21]. The mtDEPs enriched in mitochondrial dysfunction pathway (Supplementary Figure 1 item 2) provided molecular markers of the abnormal energy metabolism between cancer tissues and control tissues, including ACO1 (fold change = 0.65, *p* = 0.006), AIFM1 (fold change = 1.58, *p* = 0.002), ATPAF1 (fold change = 1.90, *p* = 0.009), ATPAF2 (fold change = 1.54, *p* = 0.0001), BCL2 (fold change = 0.30, *p* = 0.00003), COX17 (fold change = 2.74, *p* = 0.004), COX4I1 (fold change = 1.51, *p* = 0.004), COX4I2 (fold change = 0.20, *p* = 0.0006), COX6C (fold change = 1.54, *p* = 0.005), COX7A2 (fold change = 1.54, *p* = 0.03), COX7A2L (fold change = 1.87, *p* = 0.005), CYCS (fold change = 2.42, *p* = 0.001), GPX7 (fold change = 1.66, *p* = 0.0002), HTRA2 (fold change = 1.57, *p* = 0.008), MAOB (fold change = 0.41, *p* = 0.0002), TXN2 (fold change = 1.52, *p* = 0.005), and UQCRH (fold change = 1.59, *p* = 0.01). Some key molecules identified with quantitative mitochondrial proteomics, such as BCL2, PRDX5, AIFM1, VDAC1, HTRA2, COX1, and CYTB, were reported in previous studies [13, 14]. Meanwhile, energy metabolism–related pathways were also enriched in glucose metabolism pathways and in lipid metabolism pathways, such as acetone degradation I pathway, fatty acid beta-oxidation I, fatty acid beta-oxidation III, TCA cycle II (eukaryotic), and oxidative phosphorylation pathway. Those enriched pathways indicated mitochondria play important roles in maintaining the energy metabolism of tumor cells. Mitochondrial dysfunction pathway in combination with the changes of these energy metabolism pathways made ones pay a great attention on energy metabolic reprogramming of cancer.

GP6 is widely recognized as a requisite factor for the formation of platelet aggregation on a collagen surface under blood flow. Currently, platelets play a critical role in cancer development, progression, and spread of malignancy. Platelets activated by cancer cells were always detected in vitro, and the similar results appeared in clinical studies that increased levels of platelet activation in cancer patients. Moreover, platelets are in all probability involved in the development of venous thromboembolism (a frequent complication of malignant disease) in cancer patients associated with high mortality [22]. Those data suggest that continuous activation and thus exhaustion of platelets were involved in cancer-associated venous thromboembolism and cancer mortality. The mtDEPs enriched in GP6 signaling pathway (Supplementary Figure 1 item 3) showed those molecules may took part in continuous activation of platelets to affect cancer development, progression, and spread of malignancy. The mtDEPs enriched in GP6 signaling pathway provided molecular markers between cancer tissues and control tissues, including ADAM10 (fold change = 1.57, *p* = 0.0003), COL10A1 (fold change = 0.24, *p* = 0.0002), COL16A1 (fold change = 0.43, *p* = 0.0007), COL1A1 (fold change = 0.30, *p* = 0.00008), COL1A2 (fold change = 0.29, *p* = 0.001), COL2A1 (fold change = 0.17, *p* = 0.0007), COL3A1 (fold change = 0.30, *p* = 0.0006), COL4A2 (fold change = 0.39, *p* = 0.004), COL5A2 (fold change = 0.24, *p* = 0.003), COL6A1 (fold change = 0.40, *p* = 0.001), COL6A2 (fold change = 0.44, *p* = 0.0005), COL6A6 (fold change = 0.40, *p* = 0.002), COL8A1 (fold change = 0.33, *p* = 0.0009), FGFR4 (fold change = 0.39, *p* = 0.00001), ITPR1 (fold change = 0.59, *p* = 0.00006), LAMA2 (fold change = 0.58, *p* = 0.003), LYN (fold change = 1.54, *p* = 0.03), PRKCA (fold change = 0.57, *p* = 0.004), and PTPN11 (fold change = 0.37, *p* = 0.001). Some tumor-related molecules in this pathway are frequently reported in recent years. PTPN11 is a member of the protein tyrosine phosphatase family. PTPs are well known to be signaling molecules that regulate a series of cellular processes, including cell migration, differentiation, metabolic control, cell growth, oncogenic transformation, and mitotic cycle [23]. Protein kinase C (PKC) is a family of serine- and threonine-specific protein kinases, which can be activated by calcium or the second messenger diacylglycerol. PKC family members are involved in a variety of protein phosphorylation targets and are known to modulate activity of diverse cellular signaling pathways. Members of the PKC family have been reported to play roles in various cellular processes, such as cell transformation, cell adhesion, cell volume control, and cell cycle checkpoint [24].

The translation initiation factor EIF2 catalyzes the first regulated step of protein synthesis initiation, promoting the binding of the initiator tRNA to 40S ribosomal subunits. EIF2 is composed of 3 nonidentical subunits, the 36-kD EIF2-alpha subunit (EIF2S1), the 38-kD EIF2-beta subunit (EIF2S2), and the 52-kD EIF2-gamma subunit (EIF2S3). EIF2S1, EIF2S2, and EIF2S3 were all reported to associate tumor progression [25]. The mtDEPs enriched in EIF2 signaling pathway (Supplementary Figure 1 item 4) showed cancer cell launched the new translation process respect to control group. Glutathione-mediated detoxification pathway (Supplementary Figure 1 item 5) also has been reported to associate tumor progression [26]. Cancer cells were bound to generate a large number of reactive oxygen species (ROS), and it was no doubted that producing ROS in excess can be harmful. Glutathione peroxidase is the body’s primary antioxidant that is found in every cell. The mtDEPs enriched in glutathione-mediated detoxification pathway might prove that cancer cells developed sophisticated systems to self-protect and respond to their environment.

Beyond those important tumor-associated pathways, the other significant pathways were also closely related to the tumor occurrence and development, such as mTOR signaling, Gap junction signaling, OC signaling, protein ubiquitination pathway, and PPAR-α/RXRα activation (Supplementary Table 2). Mitochondria play important roles in multiple cellular signaling pathways and have broad biological activities. All those indicated signaling pathways that involved ovarian mitochondrial proteins were reliable for further studies of OC.

### Molecular and cellular functions provide significant tumor-related molecules

**Fig. 1:**
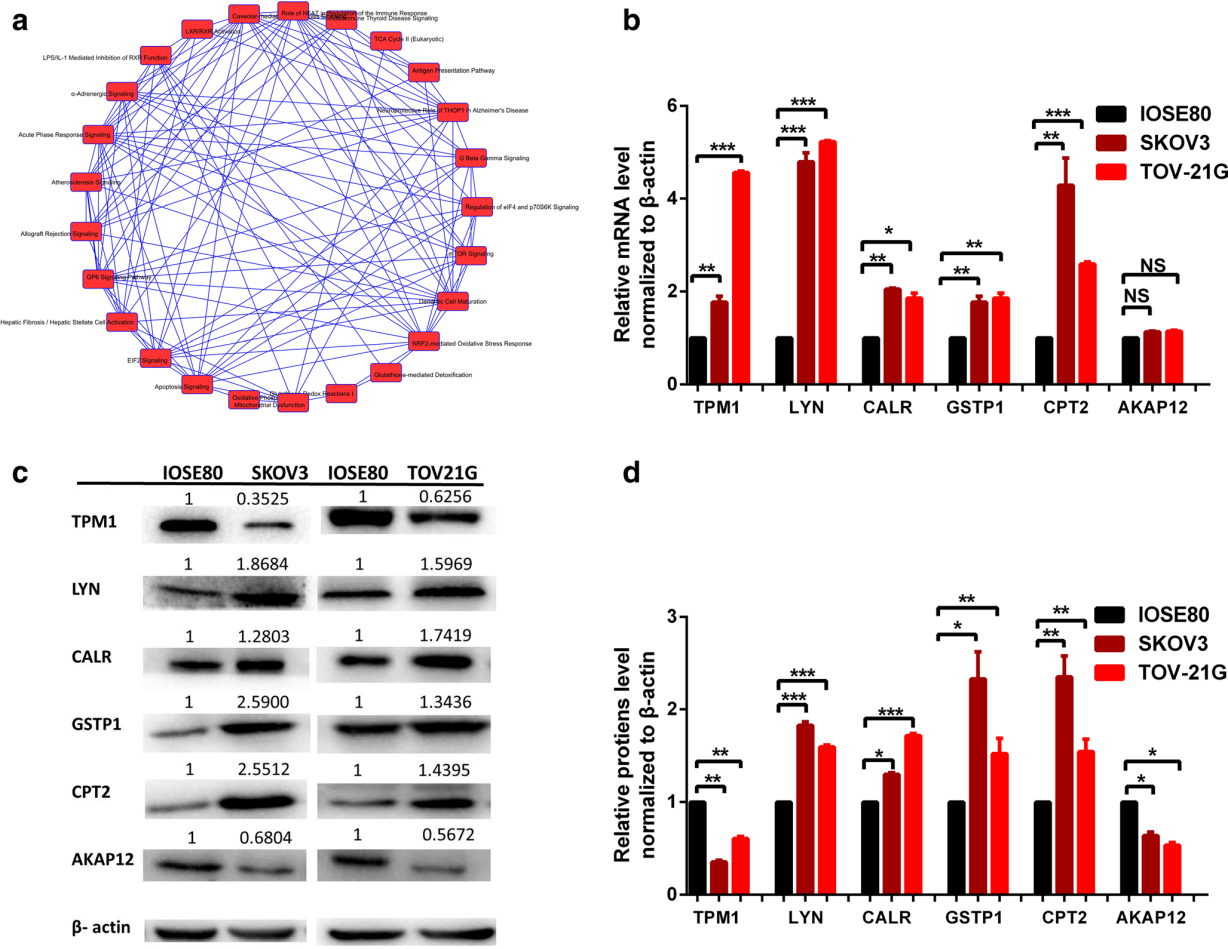
Pathway overlapping analysis and validation of cancer associated canonical pathways. **a** The interactions among those identified canonical pathways. **b** qRT-PCR quantitative analysis of the expression levels of TPM1, LYN, CALR, GSTP1, CPT2, and AKAP12 in OC cells TOV21G and SKOV3 compared to control cells IOSE80. **c** Protein expression levels of TPM1, LYN, CALR, GSTP1, CPT2, and AKAP12 in OC cells TOV21G and SKOV3 compared to control cells IOSE80. **d** Western blot quantitative analysis of TPM1, LYN, CALR, GSTP1, CPT2, and AKAP12 in OC cells TOV21G and SKOV3 compared to control cells IOSE80. *n* = 3. *p < 0.05; **p < 0.01; ***p < 0.001. NS no significance

The analyzed molecular and cellular functions include cell movement, cell death and survival, cell-to-cell signaling and interaction, free radical scavenging, and lipid metabolism (Supplementary Figure 2). Those identified molecules were related to tumor cells proliferation, migration, invasion, signal transduction, and energy metabolism. Analysis of cellular localizations of proteins in the human network found that the same molecule could appear in different cellular function networks and that the same cellular function could recruit different proteins in diverse cellular localizations. The result indicated that cancer was a multifaceted disease that involved multifactors, complex biological processes, and unpredictable consequences. It is not reasonable for the use of a single molecule as biomarker for accurate PPPM practice. Consistency was obtained by pathway overlapping analysis, which showed the interaction among those identified canonical pathways and the high biological complexity of the organism (Fig. 1a). The analyzed molecular and cellular functions provided the corresponding biomarkers with the significantly altered expressions, including that SPIN1, SPINT2, APCS, FBN1, COL4A1, TGM1, TCN1, PUM1, BCL2, S100A14, SUN2, VTCN1, HLA-A1, PKP3, COMT, TPM1, UBC, MBP, MFAP5, NCAM1, SSPN, MBP, COL1A1, and MANF were related to cell death and survival; that S100A14, VTCN1, MUC1, CD63, SLC3A2, LPAP1, NCAM1, SELENOK, SUN2, HLA-A, DCN, COL4A1, and FBN were related to cell movement; that SPINT2, COL3A1,CXCL12, COL4A1, LABA, BCL2, MC2, and HLA-A were related to cell-to-cell signaling and interaction; that DCN, NAMPT, APCS, SSPN, MUC1, CYCS, ABCB10, BNIP3, CYCS, ACTB10, BNIP3, CYBA, ACTB, BCL2, EEF1A2, and SLCI5A10 were related to free radical scavenging; and that MBP, TNFSF10, APCS, SLC27A4, SSPN, NCAM, EEF1A2, ACAA1, CLC25A10, COMT, and BCL2 were related to lipid metabolism.

### qRT-PCR and Western blot validated OC mtDEPs

To validate mtDEPs identified by iTRAQ quantitative mitochondrial proteomics, the protein expressions of the identified mtDEPs were examined, including TPM1 (fold change = 0.32, *p* = 0.0001, enriched in OC pathway), CALR (fold change = 1.97, *p* = 0.013, enriched in antigen presentation pathway), GSTP1 (fold change = 2.02, *p* = 0.037, enriched in glutathione-mediated detoxification-table pathway), CPT2 (fold change = 2.05, *p* = 0.01, enriched in lipid metabolism network), AKAP12 (fold change = 0.52, *p* = 0.003, cell movement network), and LYN (fold change = 1.54, *p* = 0.037, enriched in GP6 signaling pathway), with the expression levels of mRNAs and proteins of those DEPs in the cultured OC cells TOV21G and SKOV3 and control cells IOSE80. The significant changes in the mRNA and protein expression levels of TPM1, CALR, GSTP1, CPT2, AKAP12, and LYN were observed in the cultured cells by qRT-PCR and Western blot, respectively (Fig. 1b–d). The results of Western blot in the prepared samples had a good consistency with the results of iTRAQ quantitative mitochondrial proteomics.

### Networks derived from OC mtDEP data

A total of 1174 identifiers were mapped with IPA network analysis to the corresponding molecules (genes; proteins). Among those OC quantitative mitochondrial proteomics data, 25 networks were identified to involve mtDEPs (Supplementary Figure 3). Those 25 networks had cross-talks and similar biological functions during ovary carcinogenesis and were classified into multiple functional clusters. Cluster 1 included networks 12, 19, and 21, which are involved in nucleic acid metabolism, gene expression, and DNA replication. EIF family members, ZNF207, ANXA5, CDKN2A, SNP1, FHL2, TRAP1, RNA polymerase II, PKC(s), and NME4 play key roles in cluster 1. Cluster 2 included networks 2, 3, 6, 10, 12, 13, 16, and 17, which are involved in amino acid metabolism, post-translation modification, protein folding, and protein synthesis. NTRK1, SHMT2, GLDC, AURKAIP1, MRPS15, MRPS21, HYOU1, EMC family members, PDIA4, PDIA6, HMGA, XRCC6, H2AFX, H2AFY, ATP6AP2, CAVINI, CAVIN3, SEPT family members, Syntaxin, BLC2L1, A2FM1, SLC25A5, VDAC1, EIF family members, LGALS3BP, COX4I1, COX6C, MT-CO1, MT-CO2, and GSTP1 play key roles in cluster 2. Cluster 3 included networks 1, 8, 10, 11, 22, and 24, which are involved in cell morphology, cellular movement, cellular assembly and organization, cellular function and maintenance, cell death and survival, cellular development, cellular growth, and proliferation. FLNB, PDLIM7, FLNA, SVIL, NCL, YBX1, SPTBN1, SPTAN1, KRT19, KRT18, KRT8, VIM, DSP, TELO2, DNAJB1, Syntaxin, SNAP29, CAVIN1, ATP6AP2, SEPT family members, AIFM1, VDAC1, SLC25A5, BCL2L2, TUFM, DPYSL5, DPYSL2, TUBB4A, tubulin, LPAR1, ALB, LGALS3, and COL1A1 play key roles in cluster 3. Cluster 4 included networks 7, 12, 14, and 19, which are involved in cancer. COL6A1, PLOP1, collagen type V2, collagen, EIF family members, RPS20, ribosomal 40s subunit, PNKB, CDKN2A, TRAP1, ANXA5, ZNF207, MCM, and RAN polymerase II play key roles in cluster 4. Cluster 5 included networks 4, 5, 9, 15, 18, 20, 23, and 25, which are involved in other diseases including metabolic disease and organismal injury and abnormalities.

### The hub molecules were identified within OC tissues

**Fig. 2:**
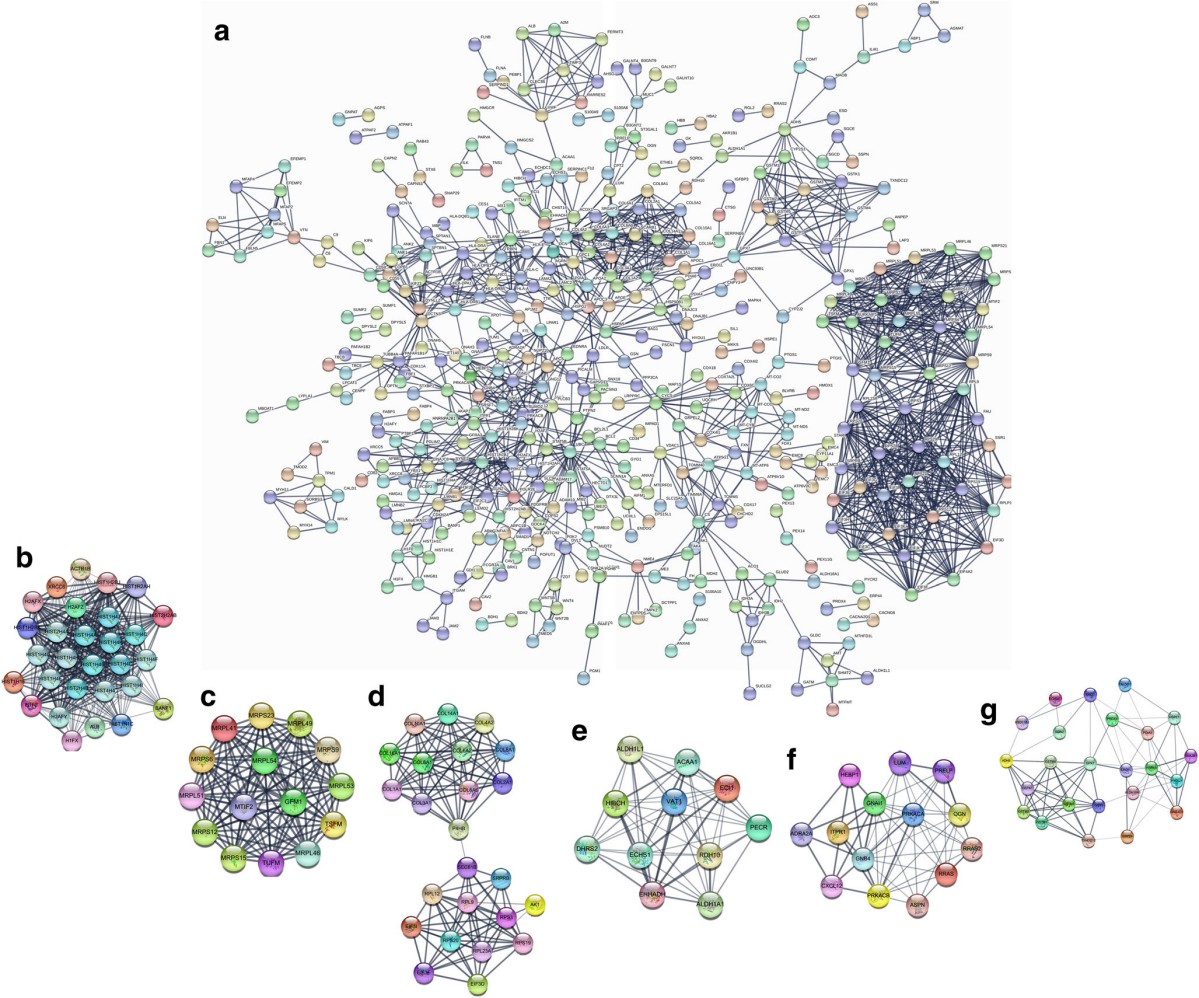
The hub molecules were identified within OC tissues. **a** The protein–protein interactions (PPIs) with combined scores greater than 0.4 were selected to construct PPI network. **b**–**g** The entire PPI network was analyzed using MCODE, and six modules (module 1 score = 24.357, module 2 score = 15, module 3 score = 10.435, module 4 score = 10.4, module 5 score = 8.769, and module 6 score = 6.609) were obtained

**Fig. 3:**
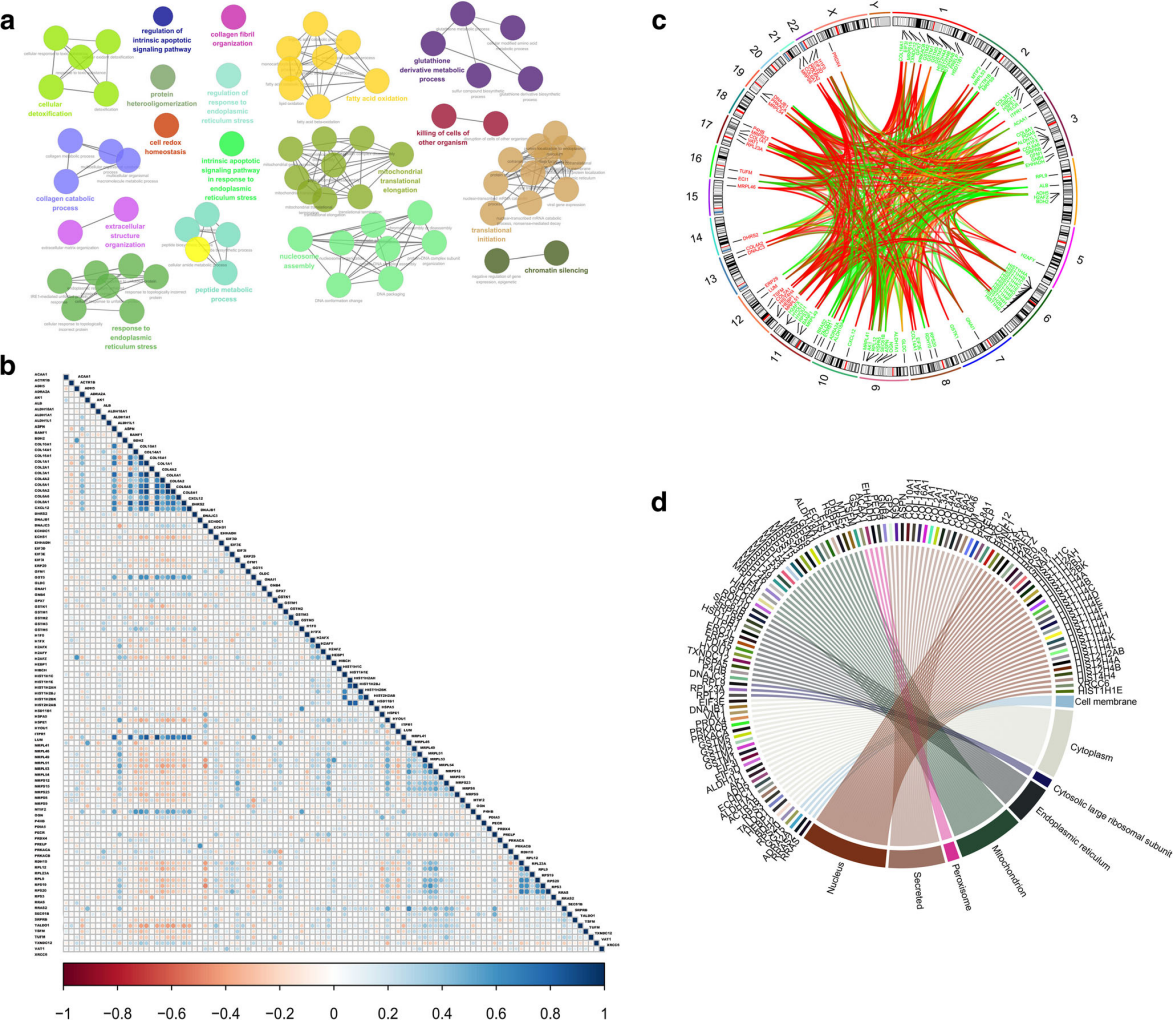
Biological processes, co-expression and co-localization analysis of hub molecules. **a** The hub molecules were classified according to the biological process. **b** The results of co-expression at the mRNA level between hub molecules. **c** The results of chromosome location and co-expression at the protein level between hub molecules. **d** The cell location of those hub molecules

The PPIs with combined scores greater than 0.4 were selected to construct PPI network (Fig. 2a). The entire PPI network was analyzed using MCODE, and six modules (module 1 score = 24.357, module 2 score = 15, module 3 score = 10.435, module 4 score = 10.4, module 5 score = 8.769, and module 6 score = 6.609) were chosen (Fig. 2b–g). Thus, a total of 102 hub molecules were identified in OCs (Table 2). Those hub molecules assisted in improving the understanding of the key molecular mechanisms underlying OC development, and the results may help the further study of the biological mechanism of OCs. GO analysis indicated that hub molecules were significantly enriched in biological processes, such as nuclear-transcribed mRNA catabolic process, translation, peptide metabolic process, and protein targeting to membrane (Fig. 3a; Supplementary Table 3).

**Table 2:** A total of 102 hub molecules were identified in ovarian cancer

ID	Gene	Description	Location	Ratio (tumor/control)
P09110	ACAA1	Acetyl-CoA acyltransferase 1	Cytoplasm	2.70
P42025	ACTR1B	ARP1 actin–related protein 1 homolog B	Cytoplasm	0.64
P11766	ADH5	Alcohol dehydrogenase 5 (class III), chi polypeptide	Cytoplasm	0.52
P08913	ADRA2A	Adrenoceptor alpha 2A	Plasma membrane	0.37
P00568	AK1	Adenylate kinase 1	Cytoplasm	0.61
P02768	ALB	Albumin	Extracellular space	0.34
P54886	ALDH18A1	Aldehyde dehydrogenase 18 family member A1	Cytoplasm	1.74
P00352	ALDH1A1	Aldehyde dehydrogenase 1 family member A1	Cytoplasm	0.44
O75891	ALDH1L1	Aldehyde dehydrogenase 1 family member L1	Cytoplasm	0.64
Q9BXN1	ASPN	Asporin	Extracellular space	0.45
O75531	BANF1	Barrier to autointegration factor 1	Nucleus	0.65
Q9BUT1	BDH2	3-Hydroxybutyrate dehydrogenase 2	Cytoplasm	0.51
Q03692	COL10A1	Collagen type X alpha 1 chain	Extracellular space	0.24
Q05707	COL14A1	Collagen type XIV alpha 1 chain	Extracellular space	0.47
Q07092	COL16A1	Collagen type XVI alpha 1 chain	Extracellular space	0.43
P02452	COL1A1	Collagen type I alpha 1 chain	Extracellular space	0.30
P02458	COL2A1	Collagen type II alpha 1 chain	Extracellular space	0.17
P02461	COL3A1	Collagen type III alpha 1 chain	Extracellular space	0.30
P08572	COL4A2	Collagen type IV alpha 2 chain	Extracellular space	0.39
P12109	COL6A1	Collagen type VI alpha 1 chain	Extracellular space	0.40
P12110	COL6A2	Collagen type VI alpha 2 chain	Extracellular space	0.44
A6NMZ7	COL6A6	Collagen type VI alpha 6 chain	Extracellular space	0.40
P27658	COL8A1	Collagen type VIII alpha 1 chain	Extracellular space	0.33
P48061	CXCL12	C-X-C motif chemokine ligand 12	Extracellular space	0.35
Q13268	DHRS2	Dehydrogenase/reductase 2	Nucleus	0.39
P25685	DNAJB1	DnaJ heat shock protein family (Hsp40) member B1	Nucleus	0.59
Q13217	DNAJC3	DnaJ heat shock protein family (Hsp40) member C3	Cytoplasm	1.62
Q9NTX5	ECHDC1	Ethylmalonyl-CoA decarboxylase 1	Cytoplasm	1.69
P30084	ECHS1	Enoyl-CoA hydratase, short chain 1	Cytoplasm	1.52
P42126	ECI1	Enoyl-CoA delta isomerase 1	Cytoplasm	1.64
Q08426	EHHADH	Enoyl-CoA hydratase and 3-hydroxyacyl CoA dehydrogenase	Cytoplasm	1.62
O15371	EIF3D	Eukaryotic translation initiation factor 3 subunit D	Cytoplasm	0.64
P60228	EIF3E	Eukaryotic translation initiation factor 3 subunit E	Cytoplasm	0.65
Q13347	EIF3I	Eukaryotic translation initiation factor 3 subunit I	Cytoplasm	0.65
P30040	ERP29	Endoplasmic reticulum protein 29	Cytoplasm	1.81
Q96RP9	GFM1	G elongation factor mitochondrial 1	Cytoplasm	1.88
P36269	GGT5	Gamma-glutamyltransferase 5	Plasma membrane	0.57
P23378	GLDC	Glycine decarboxylase	Cytoplasm	2.24
P63096	GNAI1	G protein subunit alpha i1	Plasma membrane	0.48
Q9HAV0	GNB4	G protein subunit beta 4	Plasma membrane	0.56
Q96SL4	GPX7	Glutathione peroxidase 7	Cytoplasm	1.66
Q9Y2Q3	GSTK1	Glutathione *S*-transferase kappa 1	Cytoplasm	2.13
P09488	GSTM1	Glutathione *S*-transferase mu 1	Cytoplasm	0.46
P28161	GSTM2	Glutathione *S*-transferase mu 2	Cytoplasm	0.49
B4E2J2	GSTM3	Glutathione *S*-transferase mu 3	Cytoplasm	0.37
P46439	GSTM5	Glutathione *S*-transferase mu 5	Cytoplasm	0.59
P07305	H1F0	H1 histone family member 0	Nucleus	0.32
Q92522	H1FX	H1 histone family member X	Nucleus	0.34
P16104	H2AFX	H2A histone family member X	Nucleus	0.33
O75367	H2AFY	H2A histone family member Y	Nucleus	0.47
P0C0S5	H2AFZ	H2A histone family member Z	Nucleus	0.25
Q9NRV9	HEBP1	Heme binding protein 1	Cytoplasm	0.62
Q6NVY1	HIBCH	3-Hydroxyisobutyryl-CoA hydrolase	Cytoplasm	1.58
P16403	HIST1H1C	Histone cluster 1 H1 family member c	Nucleus	0.51
P10412	HIST1H1E	Histone cluster 1 H1 family member e	Nucleus	0.34
Q96KK5	HIST1H2AH	Histone cluster 1 H2A family member h	Nucleus	0.61
P06899	HIST1H2BJ	Histone cluster 1 H2B family member j	Nucleus	0.31
O60814	HIST1H2BK	Histone cluster 1 H2B family member k	Nucleus	0.52
Q8IUE6	HIST2H2AB	Histone cluster 2 H2A family member b	Nucleus	0.22
P28845	HSD11B1	Hydroxysteroid 11-beta dehydrogenase 1	Cytoplasm	0.52
P11021	HSPA5	Heat shock protein family A (Hsp70) member 5	Cytoplasm	1.77
P61604	HSPE1	Heat shock protein family E (Hsp10) member 1	Cytoplasm	1.86
Q9Y4L1	HYOU1	Hypoxia upregulated 1	Cytoplasm	1.73
Q14643	ITPR1	Inositol 1,4,5-trisphosphate receptor type 1	Cytoplasm	0.59
P51884	LUM	Lumican	Extracellular space	0.45
Q8IXM3	MRPL41	Mitochondrial ribosomal protein L41	Cytoplasm	1.53
Q9H2W6	MRPL46	Mitochondrial ribosomal protein L46	Cytoplasm	1.53
Q13405	MRPL49	Mitochondrial ribosomal protein L49	Cytoplasm	1.56
Q4U2R6	MRPL51	Mitochondrial ribosomal protein L51	Cytoplasm	1.57
Q96EL3	MRPL53	Mitochondrial ribosomal protein L53	Cytoplasm	1.55
Q6P161	MRPL54	Mitochondrial ribosomal protein L54	Cytoplasm	1.79
O15235	MRPS12	Mitochondrial ribosomal protein S12	Cytoplasm	2.27
P82914	MRPS15	Mitochondrial ribosomal protein S15	Cytoplasm	1.50
Q9Y3D9	MRPS23	Mitochondrial ribosomal protein S23	Cytoplasm	1.78
P82932	MRPS6	Mitochondrial ribosomal protein S6	Cytoplasm	2.59
P82933	MRPS9	Mitochondrial ribosomal protein S9	Cytoplasm	1.63
P46199	MTIF2	Mitochondrial translational initiation factor 2	Cytoplasm	1.55
P20774	OGN	Osteoglycin	Extracellular space	0.47
P07237	P4HB	Prolyl 4-hydroxylase subunit beta	Cytoplasm	1.56
Q14554	PDIA5	Protein disulfide isomerase family A member 5	Cytoplasm	1.51
Q9BY49	PECR	Peroxisomal trans-2-enoyl-CoA reductase	Cytoplasm	1.57
Q13162	PRDX4	Peroxiredoxin 4	Cytoplasm	1.64
P51888	PRELP	Proline and arginine rich end leucine rich repeat protein	Extracellular space	0.34
P17612	PRKACA	Protein kinase cAMP-activated catalytic subunit alpha	Cytoplasm	0.57
P22694	PRKACB	Protein kinase cAMP-activated catalytic subunit beta	Cytoplasm	0.50
Q8IZV5	RDH10	Retinol dehydrogenase 10	Nucleus	2.05
P30050	RPL12	Ribosomal protein L12	Nucleus	0.57
P62750	RPL23A	Ribosomal protein L23a	Cytoplasm	0.65
P32969	RPL9	Ribosomal protein L9	Nucleus	0.64
P39019	RPS19	Ribosomal protein S19	Cytoplasm	0.56
P60866	RPS20	Ribosomal protein S20	Cytoplasm	0.64
P23396	RPS3	Ribosomal protein S3	Cytoplasm	0.62
P10301	RRAS	RAS related	Cytoplasm	0.64
P62070	RRAS2	RAS related 2	Plasma membrane	0.66
P60468	SEC61B	Sec61 translocon beta subunit	Cytoplasm	2.11
Q9Y5M8	SRPRB	SRP receptor beta subunit	Cytoplasm	1.55
P37837	TALDO1	Transaldolase 1	Cytoplasm	0.66
P43897	TSFM	Ts translation elongation factor, mitochondrial	Cytoplasm	1.62
P49411	TUFM	Tu translation elongation factor, mitochondrial	Cytoplasm	1.52
O95881	TXNDC12	Thioredoxin domain containing 12	Cytoplasm	1.93
Q99536	VAT1	Vesicle amine transport 1	Plasma membrane	0.64
P12956	XRCC6	X-ray repair cross complementing 6	Nucleus	0.59

Spatiotemporal coordination is a key factor in biological processes. Some hub molecules in PPI networks tend to be co-expressed or co-localized with partial hub molecules more strongly than others; a difference is possibly related to functional differences between the hub molecules [16]. In previous research, it has been suggested that various co-expression and co-localization were reflected in the molecular characteristics or structures of the hub molecules [16]. Drawing graphics of hub molecules co-expression and co-localization greatly help understand clear function and biological mechanisms of OCs. The mRNA expression data of 102 hub molecules across 419 OC patients were extracted from TCGA database (Supplementary Table 4), and the results of co-expression, correlation coefficient, and *p* value were obtained with Rstudio (Fig. 3b). The high correlations among hub molecules were found, such as COL10A1 and COL1A1, COL1A1 and COL3A1, LUM and ASPN, and HIST1H1C and HIST1H2BK. Further studies should focus on those hub molecules with high correlation, which indicated that spatiotemporal dynamics was encoded. Co-localization analysis includes chromosome location (Fig. 3c) and cell location (Fig. 3d). Multiple post-translational modifications (PTMs) of those hub molecules were predicted with SysPTM database (Supplementary Table 5), including phosphorylation, acetylation, methylation, palmitoylation, glycosylation, and interchain disulfide bridge. Those robust findings suggested that the co-expression and location of hub molecules may be regulated by PTMs.

**Fig. 4:**
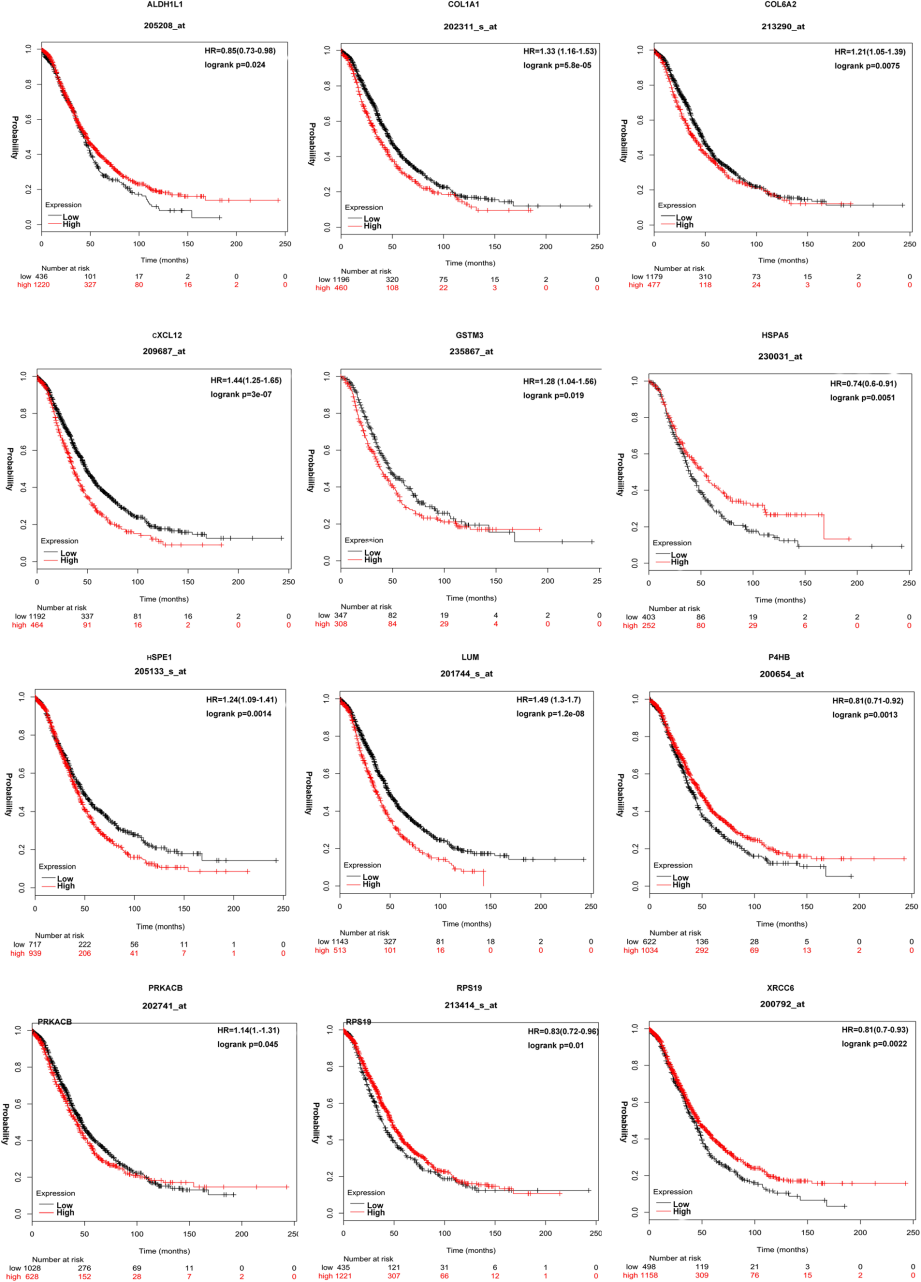
Kaplan–Meier (KM) survival curve of hub molecules ALDH1L1, COL1A1, COL6A2, CXCL12, GSTM3, HSPA5, HSPE1, LUM, P4HB, PRKACB, RPS19, and XRCC6 in OCs

The KMplot results (*p* < 0.05) revealed that 62 of 102 hub molecules resulted in significant OC overall survival. A total of 12 hub molecules, including CXCL12 (fold change = 0.34, *p* = 0.0051), HSPA5 (fold change = 1.77, *p* = 0.0001), P4HB (fold change = 1.56, *p* = 0.007), HSPE1 (fold change = 1.86, *p* = 0.003), LUM (fold change = 0.45, *p* = 0.001), XRCC6 (fold change = 0.59, *p* = 0.0002), ALDH1L1 (fold change = 0.64, *p* = 0.002), RPS19 (fold change = 0.56, *p* = 0.0003), GSTM3 (fold change = 0.37, *p* = 0.0005), PRKACB (fold change = 0.50, *p* = 0.002), COL1A1 (fold change = 0.30, *p* = 0.00008), and COL6A2 (fold change = 0.44, *p* = 0.0005), were coincidence with previous studies (Fig. 4). The other 50 hub molecules that have not been reported previously may be new findings in OCs, such as ASPN, COL3A1, COL6A6, COL10A1, DNAJB1, GFM1, GNAI1, GNB4, GSTM5, HEBP1, HIBCH, HIST1H4D, HIST1H4F, HYOU1, MRPL46, MRPL53, MRPS12, OGN, PDIA5, PRELP, RDH10, RRAS, and RRAS2 (Supplementary Figure 4).

Moreover, survival risk score system was constructed based on hub molecule signature. Those 102 hub molecules were subjected to SPSS 20 to construct a survival risk score system by multivariate regression analysis. The regression coefficient for hub molecule was generated (Table 3). The significance level was set as *p* < 0.05. The survival risk score was calculated as follows: Survival risk score = (0.136 × expression level of HIST1H2BK) + (0.171 × expression level of ALB) + (0.115 × expression level of RRAS2) + (0.101 × expression level of HIBCH) + (− 0.120 × expression level of EIF3E) + (0.228 × expression level of RPS20) + (− 0.184 × expression level of RPL23A). Higher score of this model indicated longer survival time or lower mortality risk for OC patients.

**Table 3:** Regression model for survival risk score system based on 102 hub molecules as independent variables and overall survival (OS: days) as dependent variable*

Independent variables	Unstandardized coefficients		Standardized coefficients	*t*	Significance (*p* value)
	B	Std. error	Beta		
(Constant)	− 770.138	689.051		− 1.118	0.264
HIST1H2BK	106.767	37.540	.136	2.844	0.005
ALB	422.058	115.081	.171	3.667	0.000
RRAS2	195.161	79.392	.115	2.458	0.014
HIBCH	166.011	77.862	.101	2.132	0.034
EIF3E	− 163.306	68.674	− .120	− 2.378	0.018
RPS20	241.445	63.608	.228	3.796	0.000
RPL23A	− 223.377	71.427	− .184	− 3.127	0.002

*ANOVA, df = 7, *F* = 8.625, *p* = 0.000

## Discussion

Molecular network changes are the hallmark in the pathogenesis of OCs and benefit for the discovery of effective and reliable biomarkers for early diagnosis, prognostic evaluation, or targeted therapy to prolong survival time of the patients [27]. Mitochondria are dynamic organelles that are essential for biological process and play key roles in energy metabolism, immunity adjustment, cell cycle, cell proliferation and apoptosis, and autophagy [28]. Mitochondrial dysfunctions could have a broad impact on the human diseases, including cancer [29]. In recent years, some studies have been focusing on mitochondria contributing to malignant transformation and carcinoma progression [11]. Mitochondrial biomarkers exhibit important scientific merits in human medical researches [30]. For example, the increased expression of trefoil factor family 3 (TFF3) is identified in a variety of cancers. TFF3 silencing induces the mitochondria-mediated apoptosis signaling pathway by enhancing BAX translocating to the mitochondria and by increasing the expression of the mitochondrial pro-apoptotic proteins. Thus, it is useful to develop mitochondrial biomarkers related to carcinogenesis [31]. In addition, as important intracellular organelles, the mitochondria play a critical role in regulating cancer signaling pathway, by structural derangement and metabolic modulation. For example, PINK1–PARK2 pathway increases mitochondrial iron accumulation, which activated the Warburg effect and inflammasome in tumor cells. These findings demonstrated that mitochondrial disorders may lead to cancer development [32].

In our long-term program of mitochondrial proteomics in OCs, we firstly quantitatively mapped 5115 mitochondrial proteins in OC mitochondrial samples and 52 statistically significant KEGG pathways from those mapped 5115 mitochondrial proteins [13], which is the basic data and reference profiling for in-depth study of OC mitochondrial functions. Furthermore, mtDEPs (*n* = 1198) were identified to offer a direct data for analysis of mitochondrial function abnormalities in OCs [14, 18]. Here we used those 1198 mtDEPs to construct 192 statistically canonical pathways and 25 networks and extracted 102 hub molecules, and 62 hub molecules were significantly related to cancer survival rates. Among 192 significantly IPA-canonical pathways derived from 1198 mtDEPs, 18 mtDEP-derived IPA canonical pathways were also included in the list of 52 significant KEGG pathways derived from 5115 mapped mtEPs [13], and two energy metabolic pathways (oxidative phosphorylation, and TCA cycle) were also revealed with KEGG pathway analysis from mapped mtEPs data [13] and quantitative mtDEPs data [14], with activation *z* score > 2 (Supplementary Table 6). Therefore, compared to our previously reported KEGG pathways from mapped mtEP data and mtDEPs data, this study provided much more comprehensive, complete, and overall signaling pathway profiling and networks mined from 1198 mtDEPs with IPA analysis from the point of systematic view, annotated activation and inhibition of canonical pathways based on *z* score, and revealed significant seven hub molecule signature models (HIST1H2BK, ALB, RRAS2, HIBCH, EIF3E, RPS20, and RPL23A) to assess EOC survival risks. Comprehensive analysis of all obtained data, including canonical pathways, networks, TCGA data, and experimental validated data, revealed several signaling pathways and networks were significantly associated with OC through regulating fundamental biologic behavior of tumor, such as immune dysregulation, tumor promotion inflammation, deregulating cellular energetics, cell death, proliferation signaling, and tissue invasion and metastasis. Two important OC-associated pathway network systems are in-depth discussed here, including immune dysregulation and cellular energetics.

Immune dysregulation signaling pathway system: An emerging hallmark of tumor evading immune dysregulation was still an unresolved issue as to how immune system plays in resisting formation and progression of tumors or micrometastases [33]. Immune surveillance proposes immune system monitored cells and tissues and eliminated incipient cancer cells. However, solid tumors sometimes appeared defective immunological monitoring and could evade eradication. Tumor evading immune dysregulation might be correlated with prognosis in many tumors. For example, the heavily infiltrated with CTLs and NK cells in colon and ovarian tumors was related to a better prognosis [34]. This study enriched a series of pathways related to immune system, including altered T cell and B cell signaling in rheumatoid arthritis, calcium-induced T lymphocyte apoptosis, cd28 signaling in T helper cell, icos signaling in T helper cells, nur77 signaling in T lymphocytes, T helper cell differentiation, B cell development, crosstalk between dendritic cells, and natural killer cells. T cell, B cell, and natural killer cells are the main immune cells in vivo, and the findings were similar to what has been previously reported. This study provided a number of immune-related pathways on OC, and those identified proteins may act as antitumor immunity to block tumor formation and progression in humans. For example, perspectives of TLR2 (T cell and B cell signaling in rheumatoid arthritis pathway) agonists in vaccine-adjuvant immunotherapy for cancer have been reported [35]. Other proteins, including HLA-A, HLA-DMA, HLA-DQA1, HLA-DQB1, HLA-DRA, HLA-DRB1, and HLA-DRB5, were not reported on tumor–host immunological interactions, which indicated the proteins need further studies to testify them as novel OC biomarkers for tumor immunology. CAPN2, ITPR1, PPP3CA, and PRKCA were enriched in calcium-induced T lymphocyte apoptosis pathway, and not reported on tumor-host immunological interactions, but those proteins were repeatedly reported relevant to human cancers [36]. It remains unknown whether those molecules influence development of carcinomas by immune system. SHP2, encoded by the PTPN11 gene (cd28 signaling in T helper cell), is a member of the protein tyrosine phosphatase family. SHP2 was involved in multiple cell signaling pathways, including Ras/MAPK and Hippo/YAP pathways, and led to the progression of various cancer types including breast cancer, gastric, and leukemia. Meanwhile, SHP2 also interacted with immune checkpoint receptors to regulate T cell activation. Thus, SHP2 was a key protein to activate T cell immune responses toward cancer cells [37]. In addition, other identified markers of immune-related pathways may also provide clues about crosstalk between tumor and immunity, including icos signaling in T helper cells (CD40, FGFR4, HLA-A, HLA-DMA, HLA-DQA1, HLA-DQB1, HLA-DRA, HLA-DRB1, HLA-DRB5, ITPR1, PPP3CA, and PTPN11), nur77 signaling in T lymphocytes (BCL2, CYCS, HLA-A, HLA-DMA, HLA-DQA1, HLA-DQB1, HLA-DRA, HLA-DRB1, HLA-DRB5, and PPP3CA), T helper cell differentiation (CD40, HLA-A, HLA-DMA, HLA-DQA1, HLA-DQB1, HLA-DRA, HLA-DRB1, and HLA-DRB5), B cell development (CD40, HLA-A, HLA-DMA, HLA-DQA1, HLA-DQB1, HLA-DRA, HLA-DRB1, and HLA-DRB5), and crosstalk between dendritic cells and natural killer cells (ACTB, ACTC1, CD40, FSCN1, HLA-A, HLA-C, HLA-DRA, HLA-DRB1, HLA-DRB5, HLA-E, and TNFSF10).

Cellular energetic signaling pathway network: An emerging hallmark of reprogramming energy metabolism made researches refocus efforts to a novel anticancer strategy on cancer cells. The study of tumor cell energy metabolism is multiangle, which focused on glucose metabolism and lipid metabolism in the past 10 years. The metabolism of glucose to lactic acid in the presence of oxygen had been recognized in cancer cells, commonly called the Warburg effect [38]. However, the reverse Warburg effect was put forward in 2009 and provided complementary mechanisms for cancer energy metabolism. Cancer cells secreted ROS to induce oxidative stress and aerobic glycolysis in mesenchymal cells. In turn, mesenchymal cells produced lots of nourishment to the adjacent cancer cells [39]. Novel evidence is shedding light on alterations in lipid metabolism-associated pathways, which have often been discounted for past years. All of the evidence suggests that lipid disorder is closely related to tumorigenesis. This study enriched a series of pathways related to energy metabolism, including TCA cycle II (eukaryotic), acetone degradation I, fatty acid beta-oxidation I, fatty acid beta-oxidation III, ketogenesis pathway, leptin signaling in obesity, and superpathway of cholesterol biosynthesis. Those identified proteins may act as antitumor energy metabolism to block tumor formation and progression in humans. For example, by microarray analysis, one confirmed that fumarate hydratase messenger RNA (TCA cycle II pathway) was low expression in renal cancer cells. Consistence with the possibility that altered gene expression of fumarate hydratase represented one change to a more anaerobic state [40]. Overnutrition was known as a confirmed independent cancer risk factor. However, the oncogenic mechanisms remain poorly understood. Enoyl-CoA hydratase-1(ECHS1), the enzyme involved in the oxidation of fatty acids (fatty acid beta-oxidation I pathway), regulates mTOR signaling and cellular apoptosis by sensing nutrients. Overnutrition suppressed enoyl-CoA hydratase-1 (ECHS1) activity and linked to increased risk of cancer [41]. Moreover, other identified markers of energy metabolism-related pathways may also provide clues about crosstalk between tumor and energy metabolism, including acetone degradation I (CYP19A1, CYP1B1, CYP2J2, CYP2S1, CYP4B1, and CYP4X1), fatty acid beta-oxidation III (ECI1, and EHHADH), ketogenesis pathway (ACAT2, BDH1, BDH2, and HMGCS2), and leptin signaling in obesity (FGFR4, JAK2, PDIA3, PLCB3, PLCH1, PRKACA, PRKACB, PRKAR2B, and PTPN11).

Moreover, some other canonical pathways and hub molecules also get ones to pay more attention to, including iron homeostasis signaling pathway, endoplasmic reticulum stress pathway, inhibition of matrix metalloproteases, mTOR signaling, protein ubiquitination pathway, RAS activation, RhoA signaling, role of tissue factor in cancer, and role of JAK family kinase in IL-6 type cytokine signaling. All those pathways are worth further studying to verify their relationship with OCs.

In addition to the pathway analysis, the key molecules in those pathway network system were also extensive analyzed with multiple methods, including molecular and cellular functions, network, and hub molecule analyses, which were potential biomarkers for the development of an OC, such that CXCL12, HSPA5, P4HB, HSPE1, LUM, ALDH1L1, RPS19, GSTM3, PRKACB, COL1A1, and COL6A2, as reported were significantly related to overall survival. To some degree, these results indicate that our findings were consistent with previous studies and also make new discoveries. Mitochondrial changes lead to morphology and functions transformation and influence downstream metabolic processes. Mitochondrial proteins can be developed as molecular biomarkers or therapeutic targets in the development of new interventions to selectively kill cancer versus normal cells [42]. For example, mitochondria or mitochondrial reactive oxygen species (ROS) in a cancer provided novel targets for anticancer therapy. Mitochondrial ROS are characterized by overproduction in cancer cells, which promotes cancer progression by modifying gene expressions, inducing genomic instability and participating in signaling pathways. Designing novel and selective mitochondria-targeted agents may help to increase therapeutic specificity and reduce drug toxicity of these agents. Mitochondria-targeted antioxidants are found to be effective based on the oncogenic role of ROS [43]. In addition, mitochondria-targeted drugs stimulated mitophagy and blocked cancer cell proliferation. Traditional methods targeting the mitochondria of cancer cells always aimed at influencing antiapoptotic proteins or inducing changing energy metabolism. Currently, tumor-associated mitochondrial antigens were recognized by the immune system, which provides a novel way to mitochondria-targeted drugs development [44].

## Strength and limitation

This study focused on mitochondria proteome and its involved molecular network alterations in OCs to reveal mitochondria-related signaling pathways and candidate biomarkers. Seven epithelial OC tissues and 11 control ovaries with benign gynecologic disease were used to prepare OC and control mitochondria for identification of 1198 mtDEPs, followed by construction of 25 statistically significant networks and 192 canonical pathways. Some important molecules, including TPM1, CALR, GSTP1, CPT2, AKAP12, and LYN, in those pathway networks were further verified in OC cells (TOV-21G and SKOV3) compared to normal ovarian cells (IOSE80), which showed the consistent results with the results of tissue mitochondrial proteomics. These findings provided the scientific data to better understand the roles of OC mitochondrial proteome from systems biology angle. Here, one should realize that (i) mitochondria from 7 OC tissues were mixed as the cancer mitochondrial sample, and mitochondria from 11 control ovaries were mixed as the control mitochondrial sample, and then the cancer and control mitochondrial samples were used for quantitative proteomics analysis to decrease between-individual heterogeneity. However, for identification of mtDEPs as biomarkers, it is necessary to verify them in an expanded OC and control ovary tissues in future. (ii) For OC cell lines, there were many types of OC cell lines, including epithelial cells (e.g., SKOV3, TOV-21G, and OV-1063), epithelial-like cells (e.g., UWB1.289, UACC-2727, and UACC-1598), and endometrioid OC cells (e.g., IGROV, and TOV112D). Cell lines SKOV3 and TOV-21G used in this study were from American Type Culture Collection (ATCC), and they were frequently used in OC-related studies. SKOV3 isolated from ovarian adenocarcinoma was one of serous epithelial ovarian carcinoma cell lines and moderately differentiated. TOV-21G from ovarian surface epithelium was isolated from primary malignant adenocarcinoma. SKOV3 and TOV-21G were all tumorigenic in nude mice to indicate malignancy degree of tumor. The control ovarian cell line (IOSE80) was also from normal ovarian surface epithelium, and it is reasonable to be used as control group. In addition, three cell lines SKOV3, TOV-21G, and IOSE80 in this study were consistent in cell origin (namely epithelial cells) with our analyzed OC and control tissues; thus, those three cell lines can be used to verify our tissue proteomics results. However, for identification of mtDEPs as biomarker or therapeutic targets, it is necessary to use more OC cell lines and normal control cells for future validation. (iii) In order to determine the clinical values of 102 hub molecules derived from 1198 mtDEPs, the present survival analysis and multivariate regression analysis of those hub molecules were not based on those 7 OC and 11 control ovary tissues used for proteomic analysis, but based on 419 OC patients in the TCGA database through obtaining TCGA mRNA data corresponding to 102 OC-tissue mtDEPs. Therefore, survival analysis and multivariate regression model analyses were derived from mtDEP-corresponding mRNA data among 419 OC patients and their clinical survival data. Thereby, the sample size (*n* = 419) was acceptable for analysis of hub molecules, survival analysis, and survival regression model in the mRNA level. However, protein is the final performer of gene. It would be necessary to further verify those hub molecule biomarkers and seven hub molecule signature models (HIST1H2BK, ALB, RRAS2, HIBCH, EIF3E, RPS20, and RPL23A) in the protein level among significantly increased clinical samples.

## Conclusions and expert recommendations

Molecular pathway network changes are the hallmark of OC pathogenesis. This study provided overall signaling pathway network change profiling of OC based on the analysis of 1198 mtDEPs between OC and control tissues, including 25 statistically significant networks, 192 statistically significant canonical pathways, and 52 activated or inhibited pathways. A total of 102 important hub molecules (proteins; genes) were identified with integrative analysis of 1198 mtDEPs and TCGA transcriptomic data of 419 OC patients, including 62 hub molecules related to survival risk of OCs. Moreover, statistically significant seven hub molecule signature models (HIST1H2BK, ALB, RRAS2, HIBCH, EIF3E, RPS20, and RPL23A) were constructed for assessment of survival risk of OC patients. These findings provided pathway network database of OCs. Moreover, in-depth analysis of hub molecules and construction of seven hub molecule signature models could assist in discovery of potential biomarkers and novel mechanisms of ovarian carcinogenesis.

We recommend this research article to promote mitochondria-based molecular network studies in OCs from multiparameter systematic opinion and emphasize the importance of OC multiomics such as integrating proteomics and transcriptomics in basic research and translational and application research in the field of personalized medicine in OCs. Especially, molecular network-based biomarkers are important for reliable and effective in personalized diagnosis and prognosis assessment of OCs, and molecular network-based clarification of molecular mechanisms is important for discovery of effective and reliable drug targets for OC personalized treatment. The identified survival-related hub molecules and seven hub molecule signature models are important resource of pattern biomarker for personalized medicine in OCs.

## Electronic supplementary material

ESM 1 (PDF 5356 kb)

ESM 2 (PDF 3550 kb)

ESM 3 (PDF 3766 kb)

ESM 4 (PDF 2864 kb)

ESM 5 (XLS 242 kb)

ESM 6 (XLS 142 kb)

ESM 7 (XLS 51 kb)

ESM 8 (XLS 794 kb)

ESM 9 (XLS 86 kb)

ESM 10 (XLS 44 kb)
