# Supplementary material for: Signaling pathway network alterations in human ovarian cancers identified with quantitative mitochondrial proteomics
Source: EPMA J. 2019 Jun 8;10(2):153–72. doi: 10.1007/s13167-019-00170-5 (PMC6562010; doi:10.1007/s13167-019-00170-5)

Supplementary Figure 1

Cancer-related canonical pathways (p<0.05, and |z|>=2) were identified from ovarian cancer mitochondrial differentially expressed proteins (mtDEPs).

1. Antigen presentation pathway
2. Mitochondial dysfunction
3. GP6 signaling pathway
4. EIF2 signaling
5. Glutathione-mediated detoxification
6. α -Adrenergic signaling
7. Creb signaling
8. CXCR4 signaling in neurons
9. Regulation of EIF4 and p70S6K signaling
10. Endothlin signaling
11. Ephrin receptor signaling
12. Fatty acid beta-oxidation I
13. FLMP signaling in neutrophils
14. G beta gamma signaling
15. Gaq signaling
16. GNRN signaling
17. IGF-1 signaling
18. IL2 signaling
19. IL-8 signaling
20. Integrin signaling
21. mTOR signaling
22. Ovarian cancer signaling
23. Oxidative phosphorylation
24. P2Y Purigenic receptor signaling pathway
25. Phospholipase c signaling
26. Signaling by Rho family GTPases
27. Antiproliferative role of somatostatin signaling
28. Tec kinse signaling
29. Thrombin signaling

1. **Antigen presentation pathway**

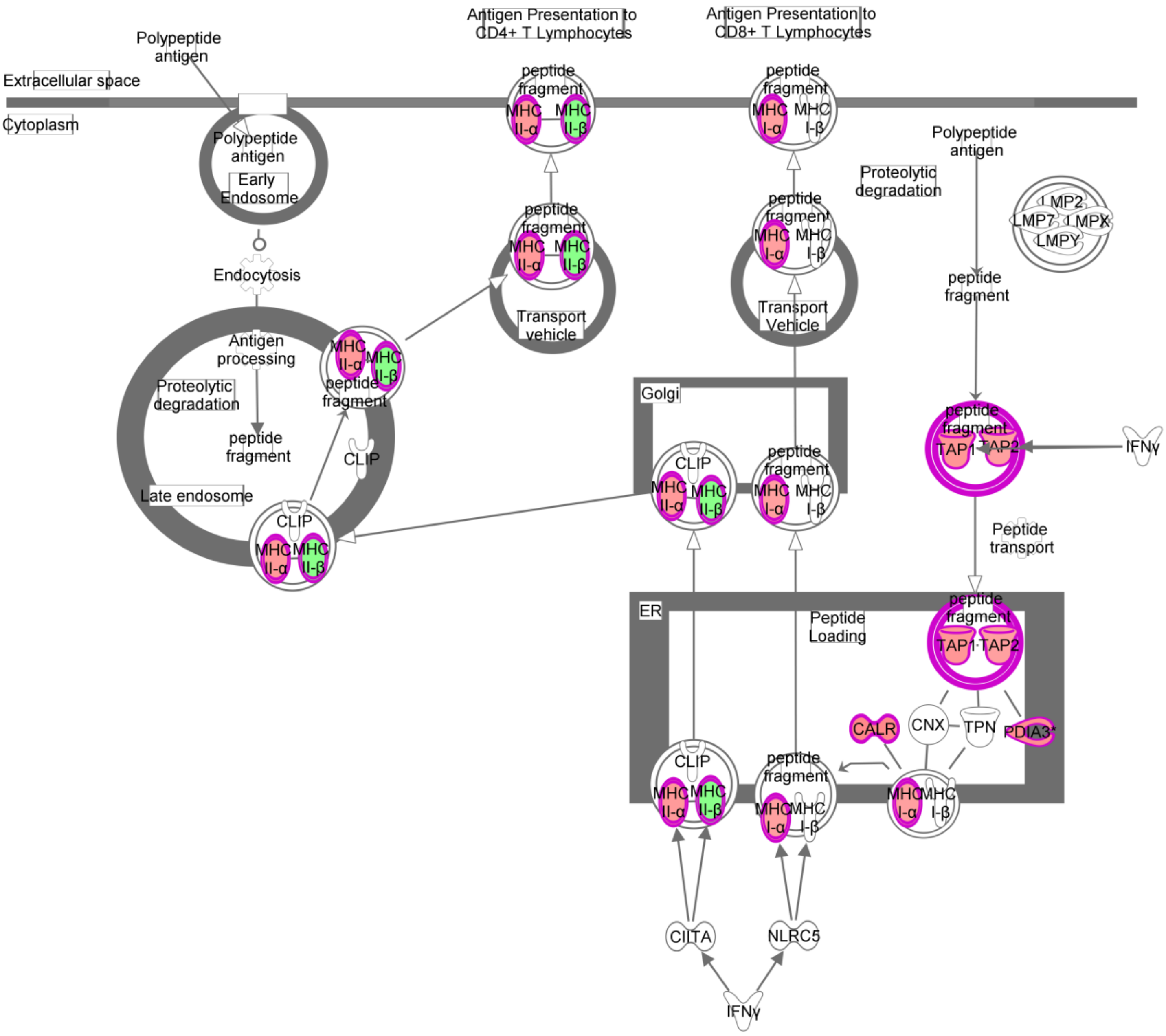

2. Mitochondial dysfunction

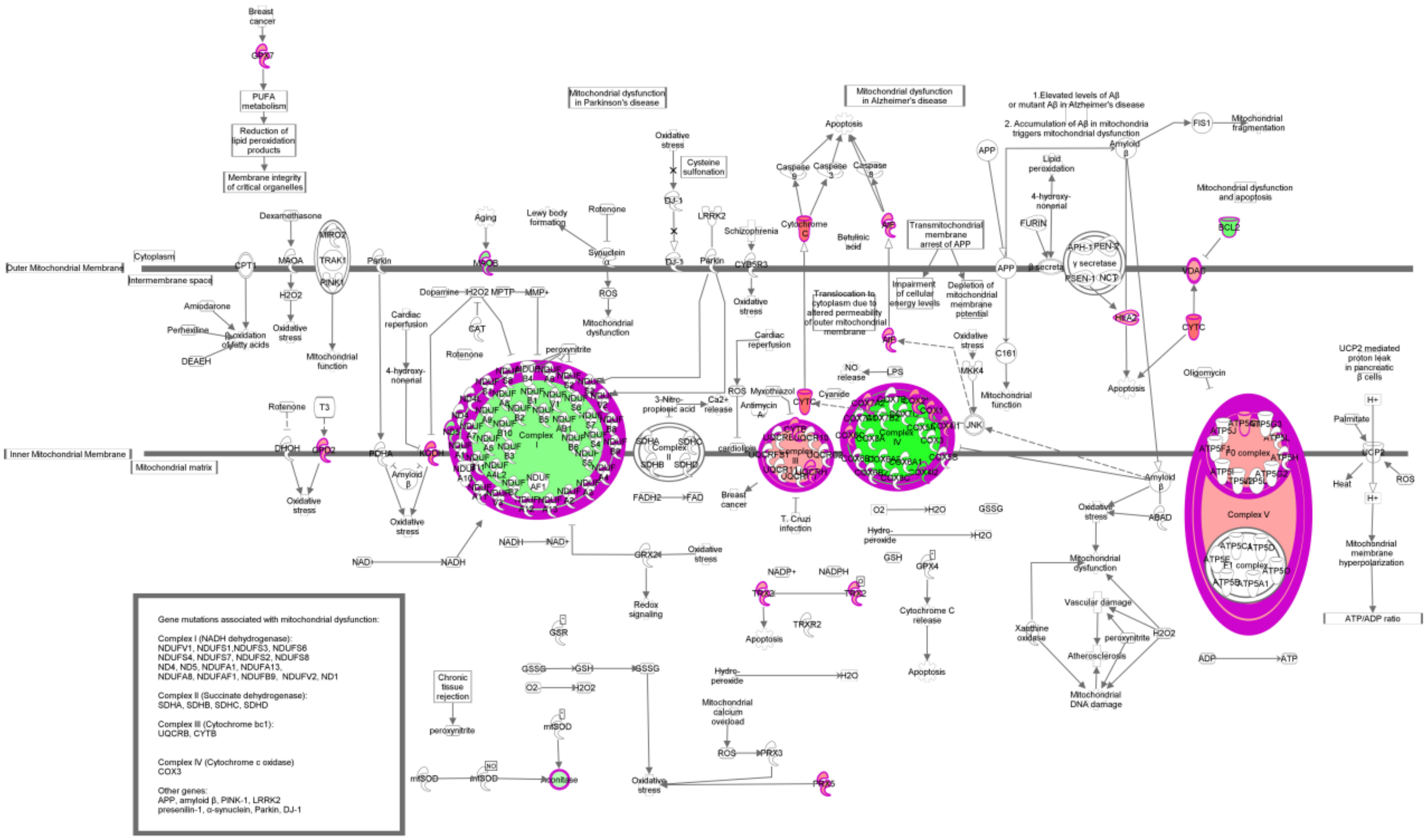

3. GP6 signaling pathway

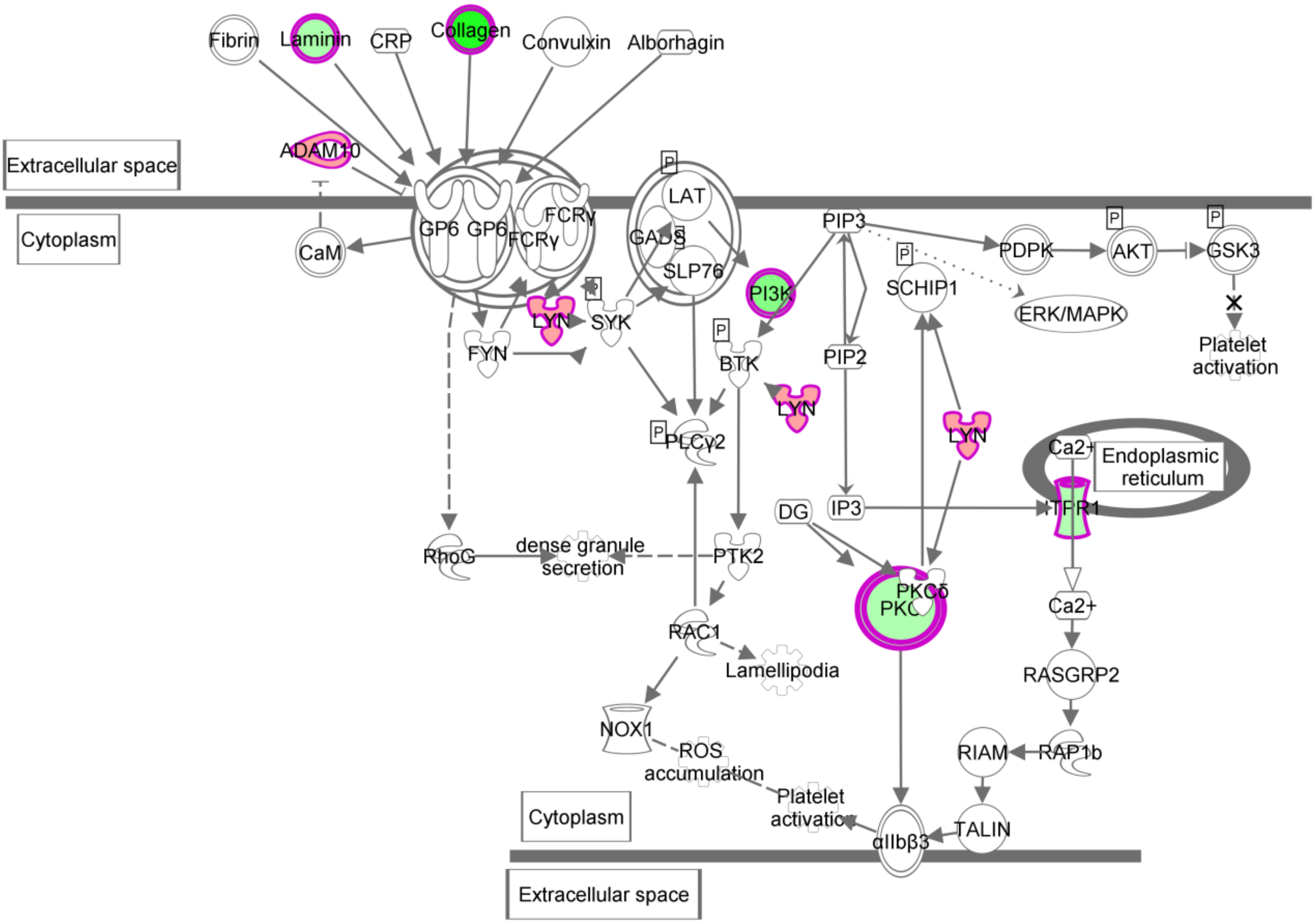

4. EIF2 signaling

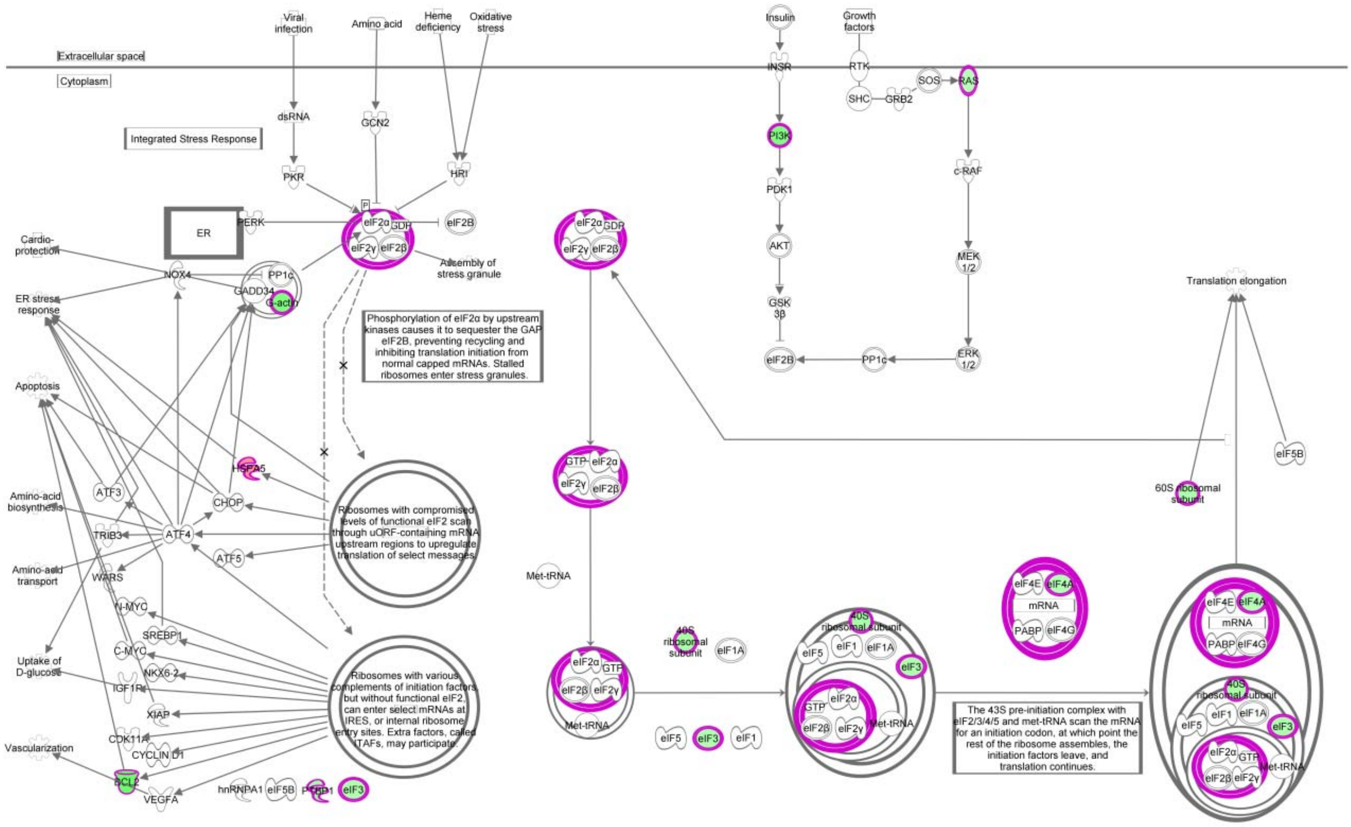

5. Glutathione-mediated detoxification

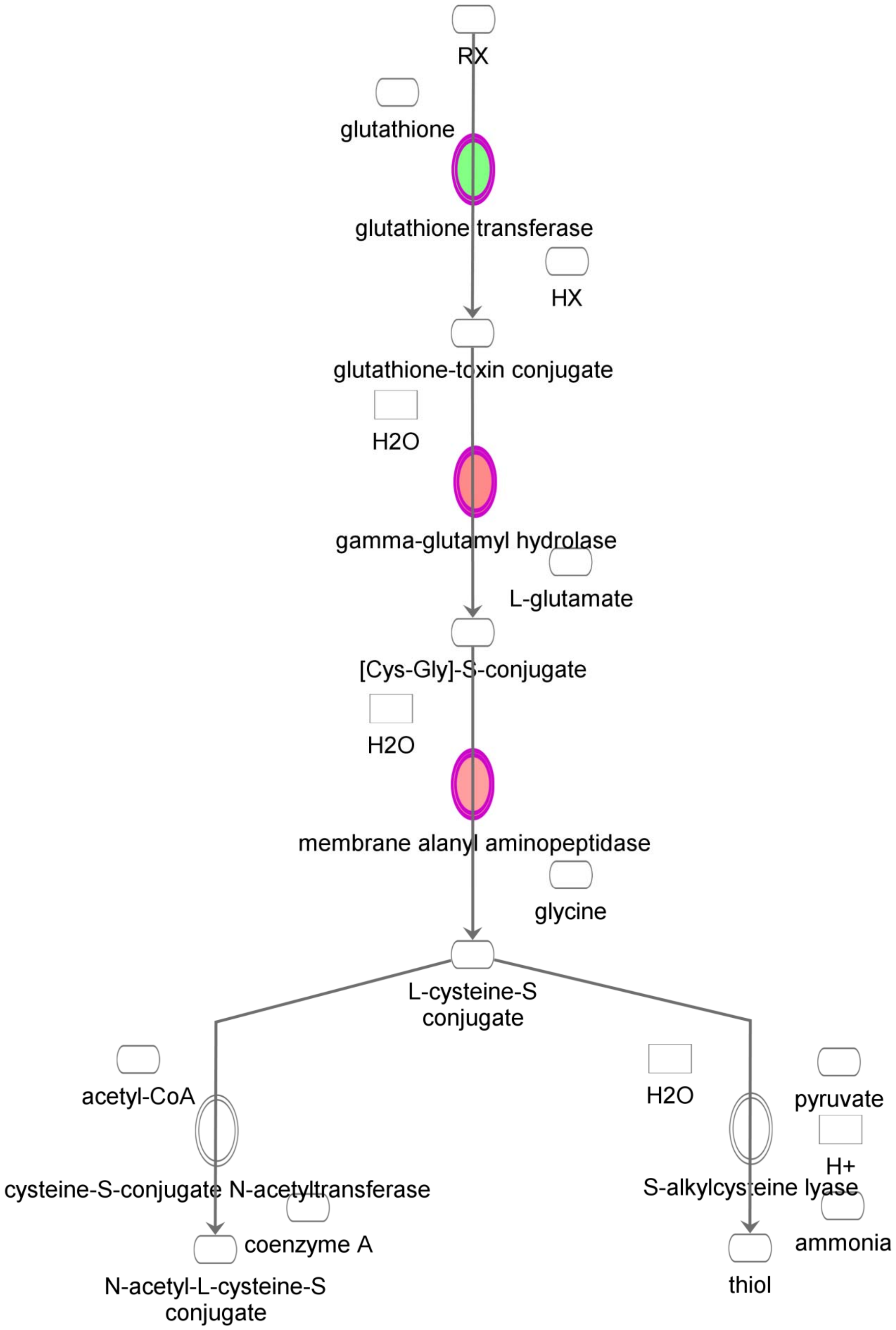

6. α -Adrenergic signaling

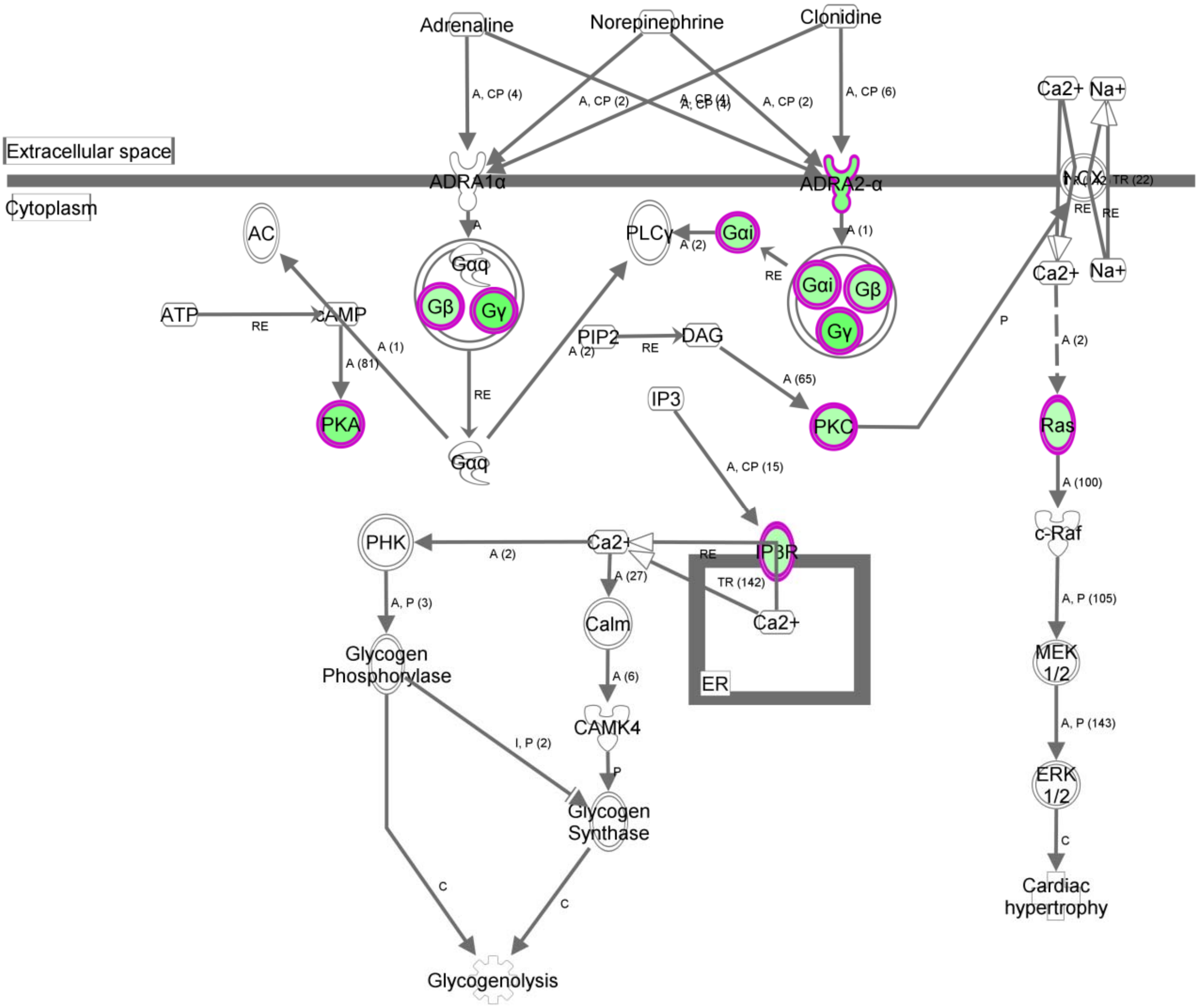

7. **Creb signaling**

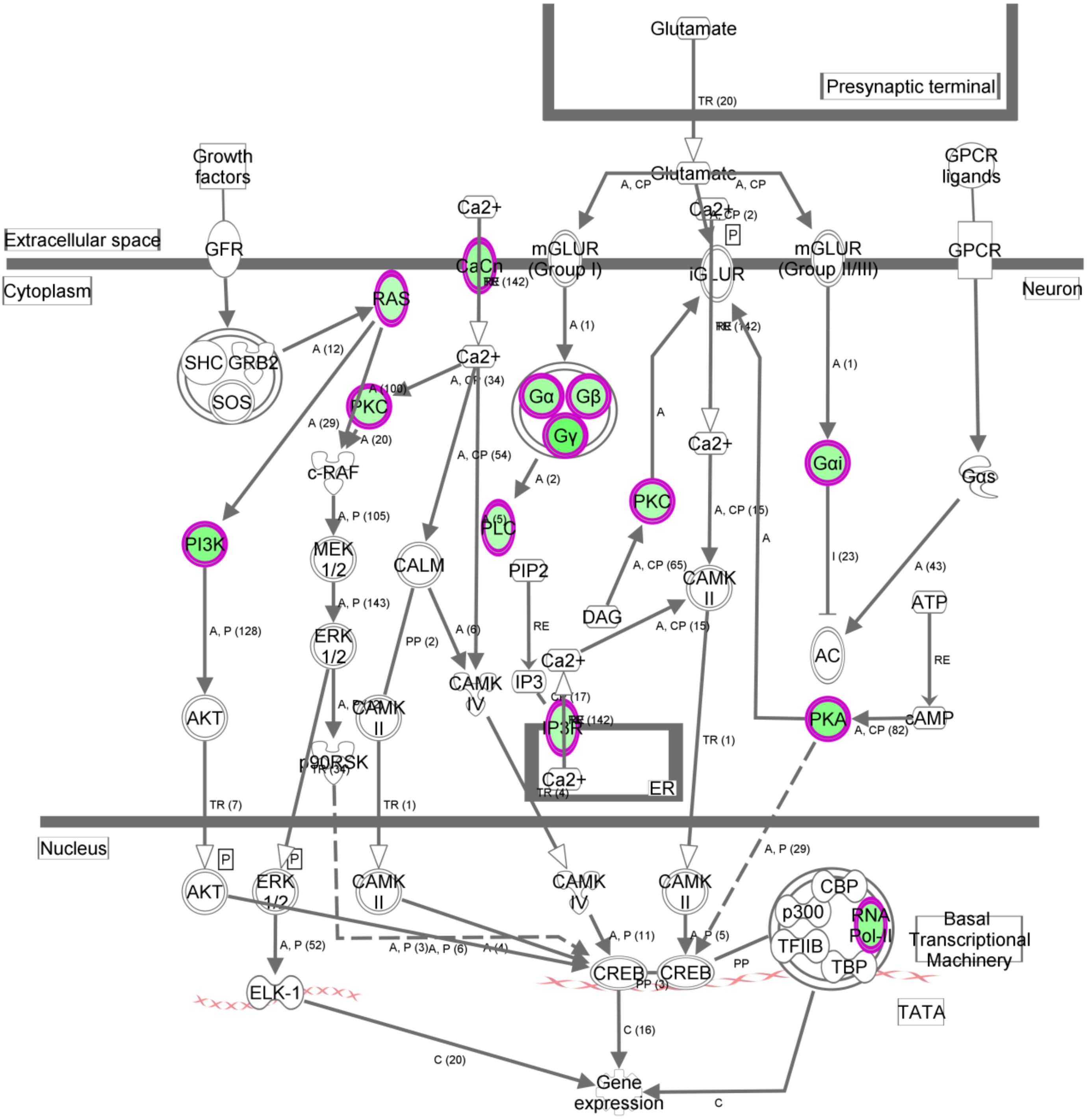

8. CXCR4 signaling in neurons

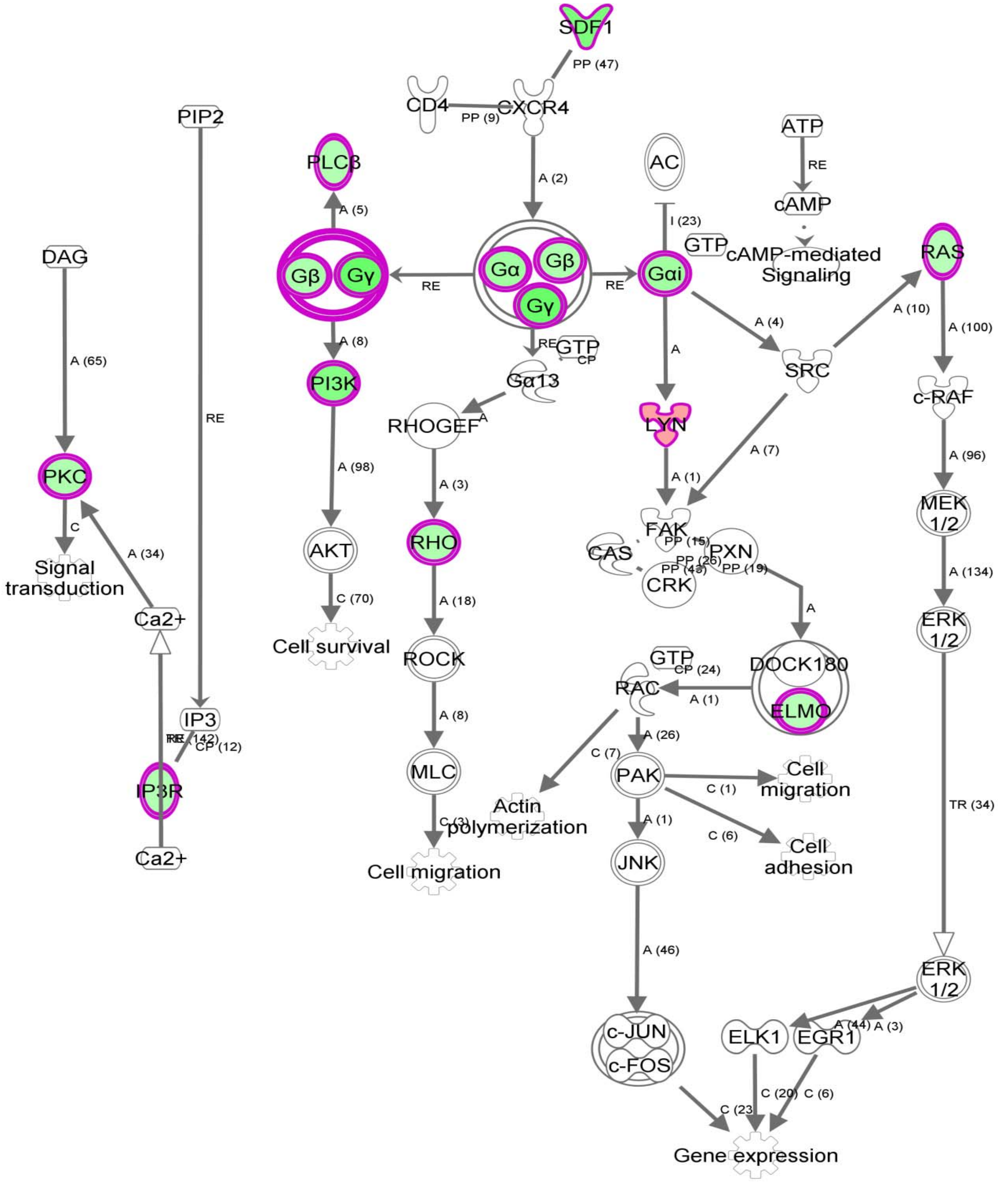

9. Regulation of EIF4 and p70S6K signaling

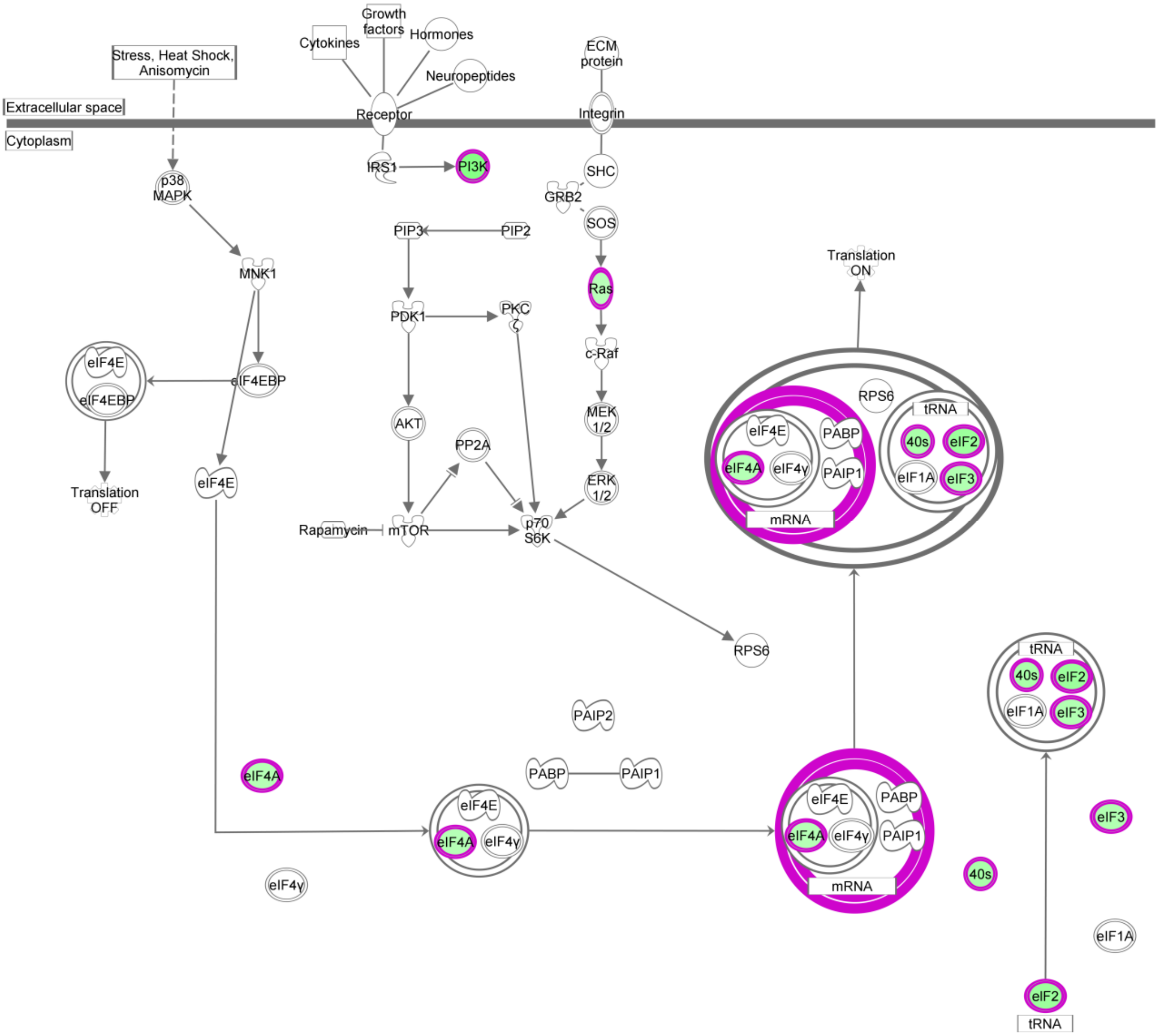

10. Endothlinm signaling

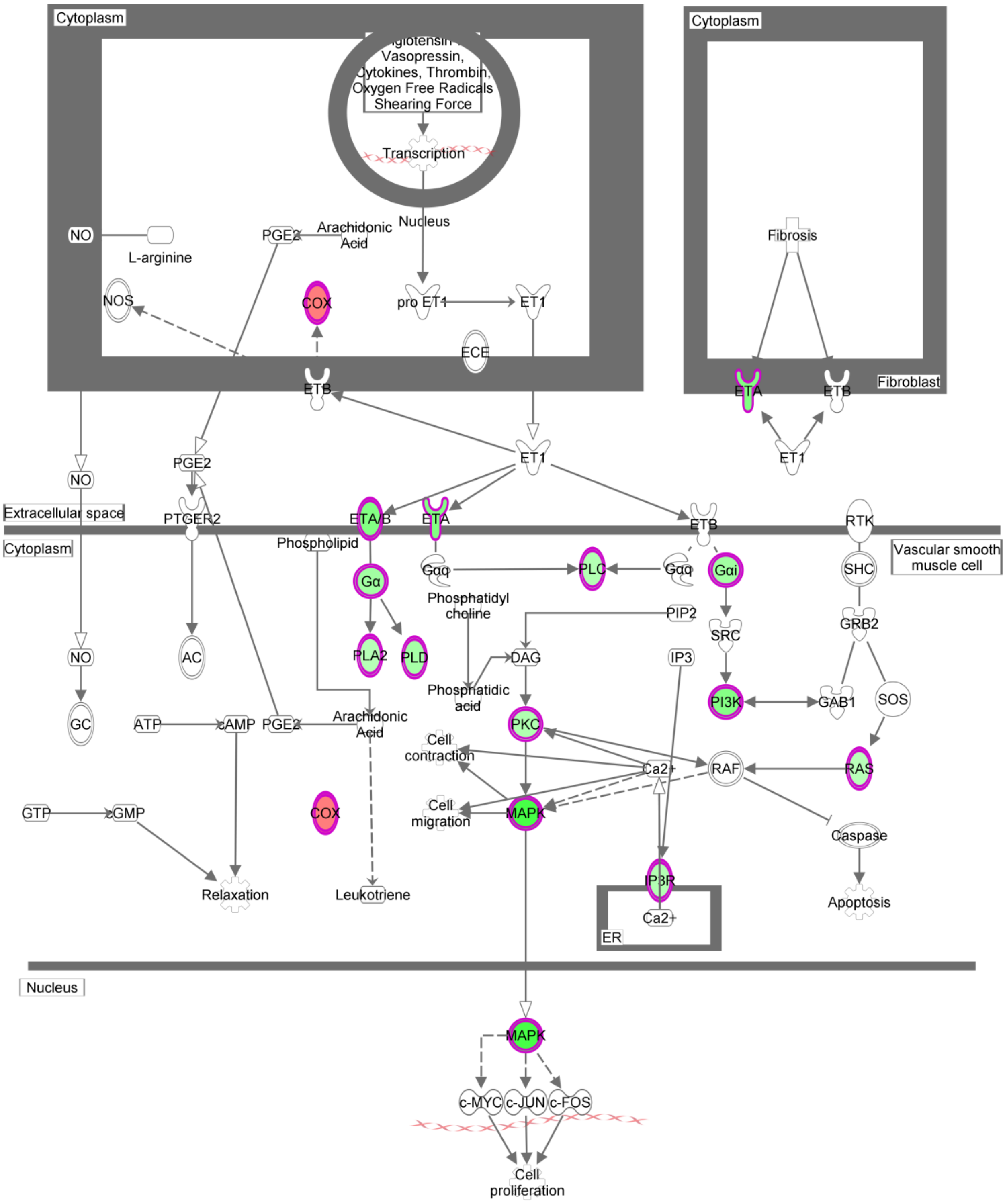

11. Ephrin receptor signaling

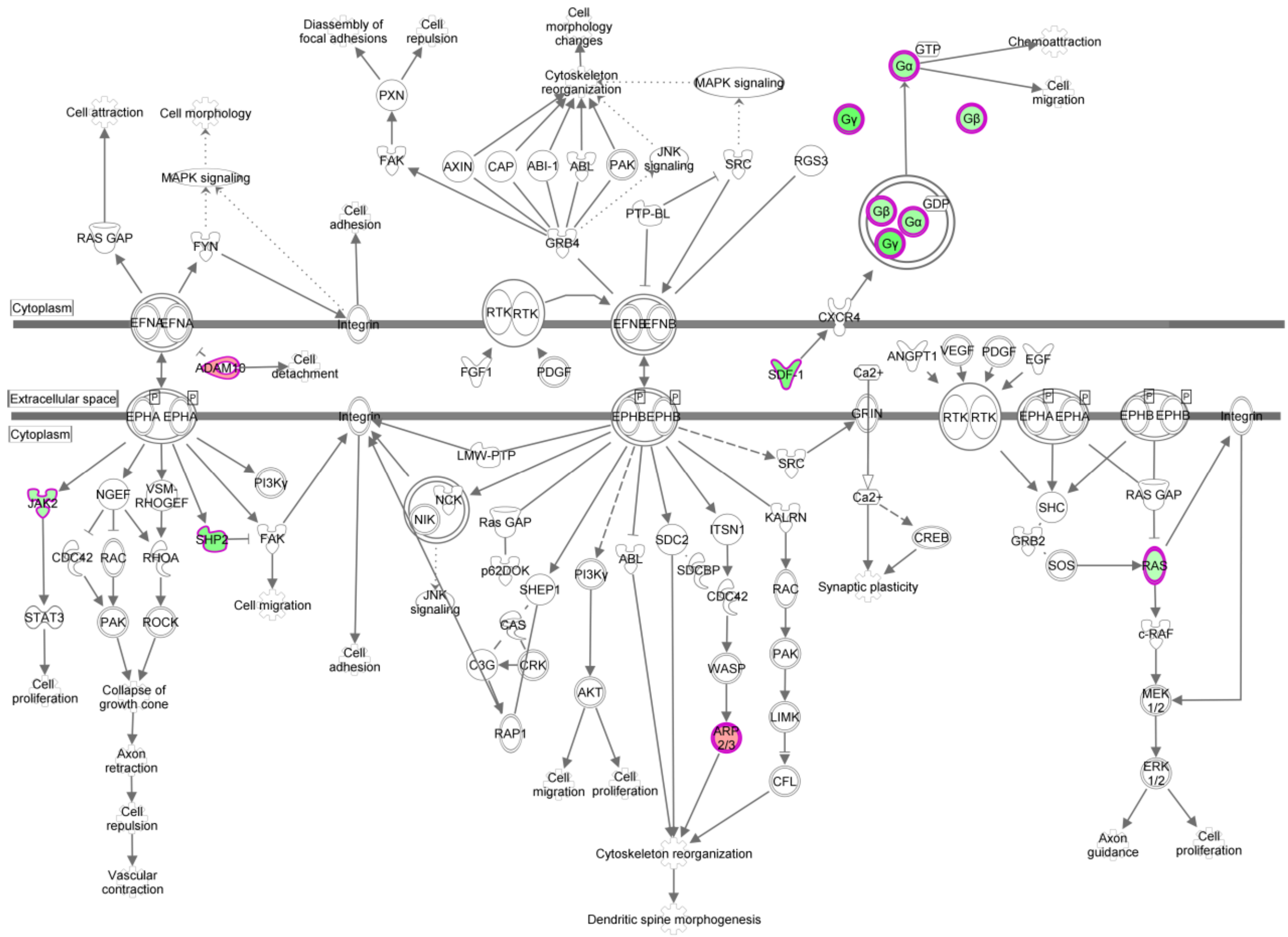

12. Fatty acid beta-oxidation I

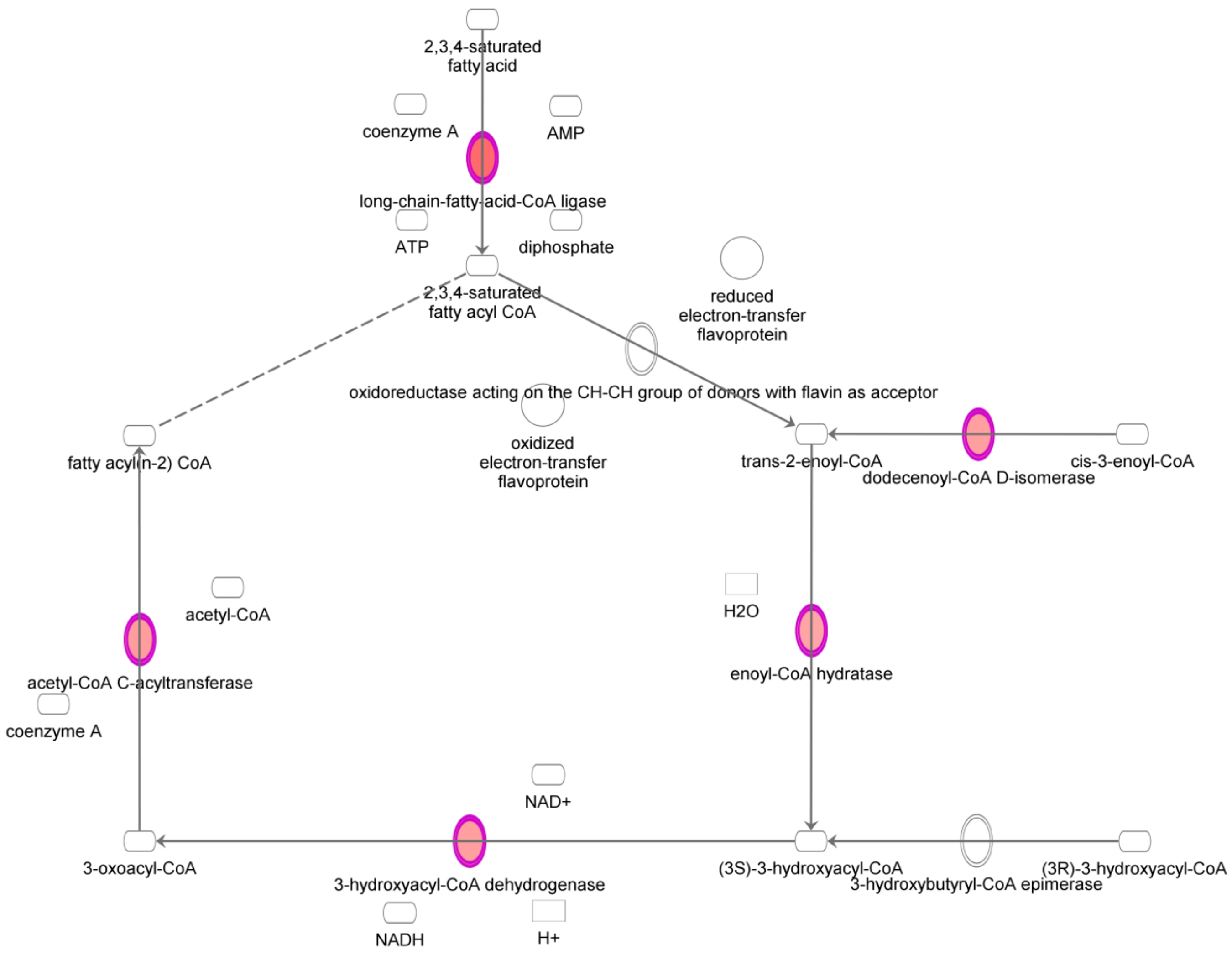

13. FLMP signaling in neutrophils

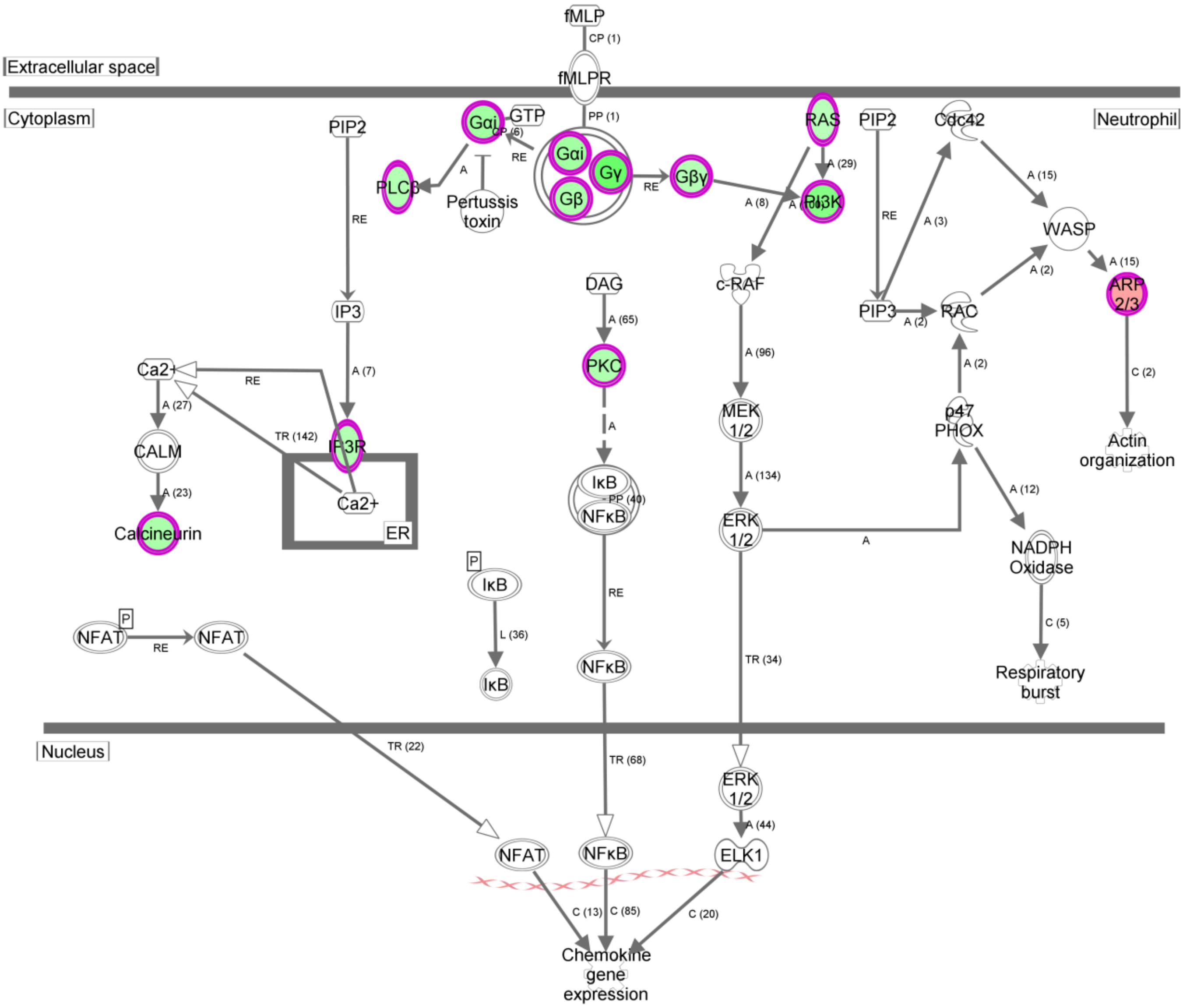

14. G beta gamma signaling

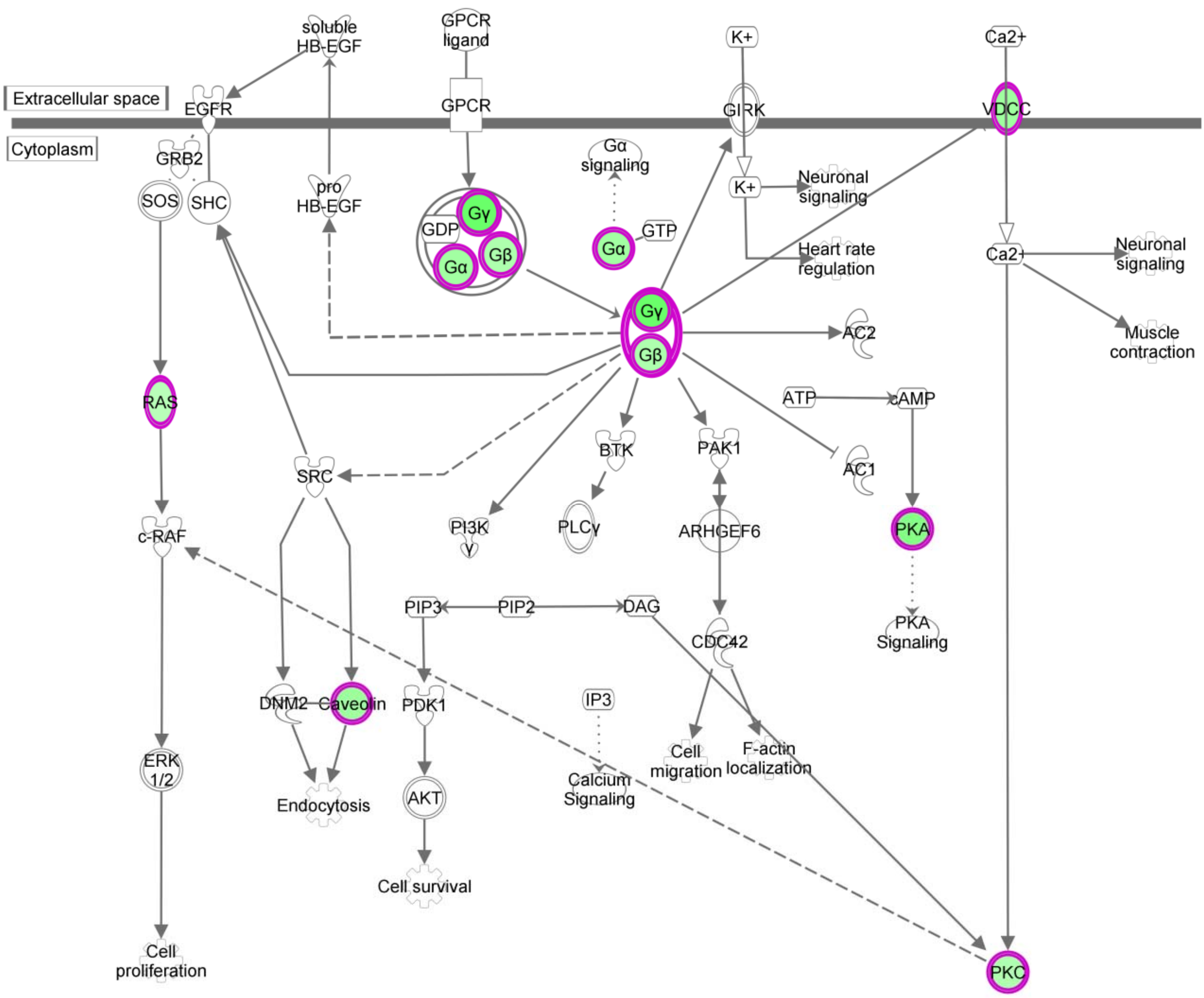

15. Gaq signaling

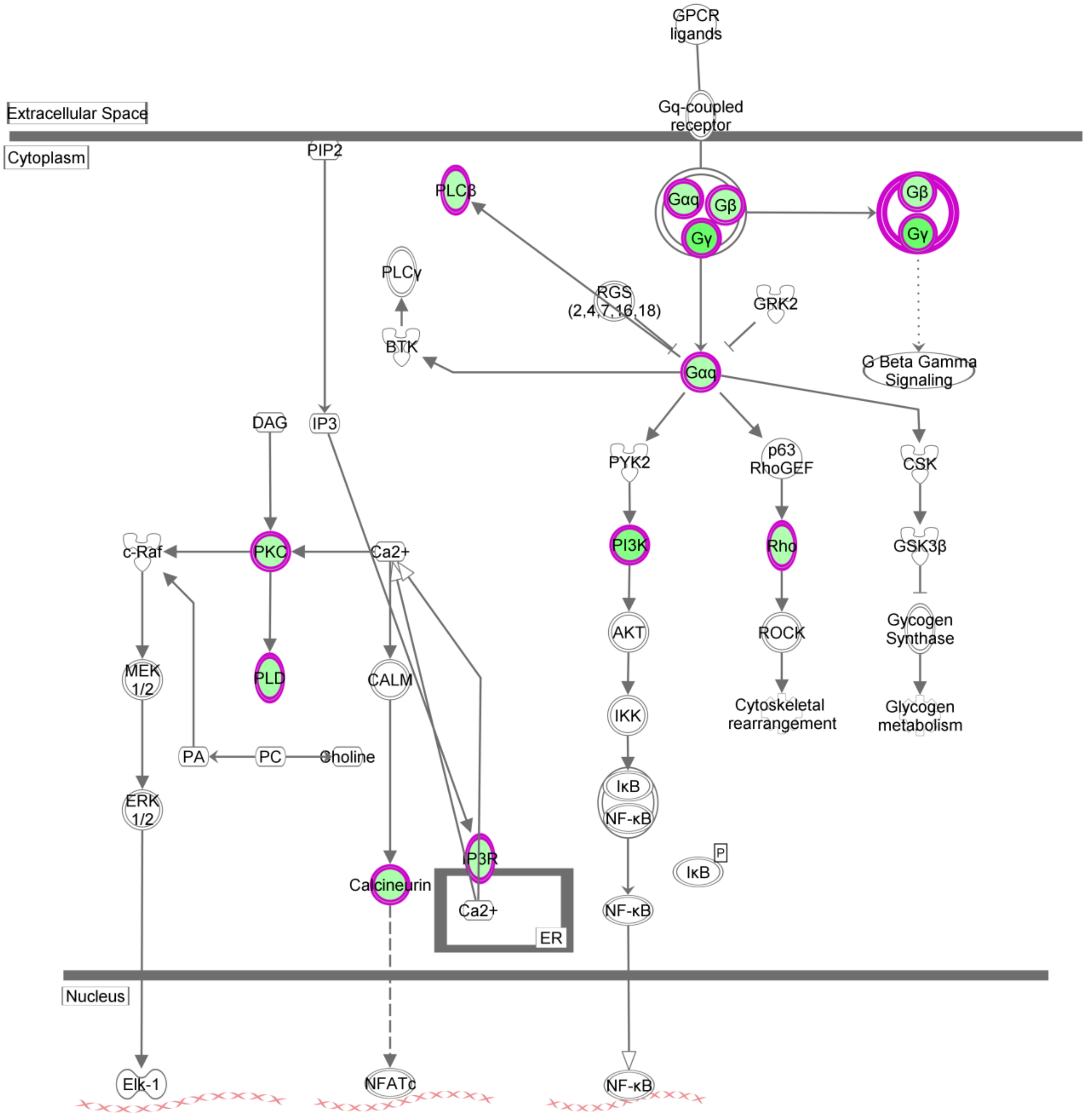

16. GNRN signaling IGF-1 signaling

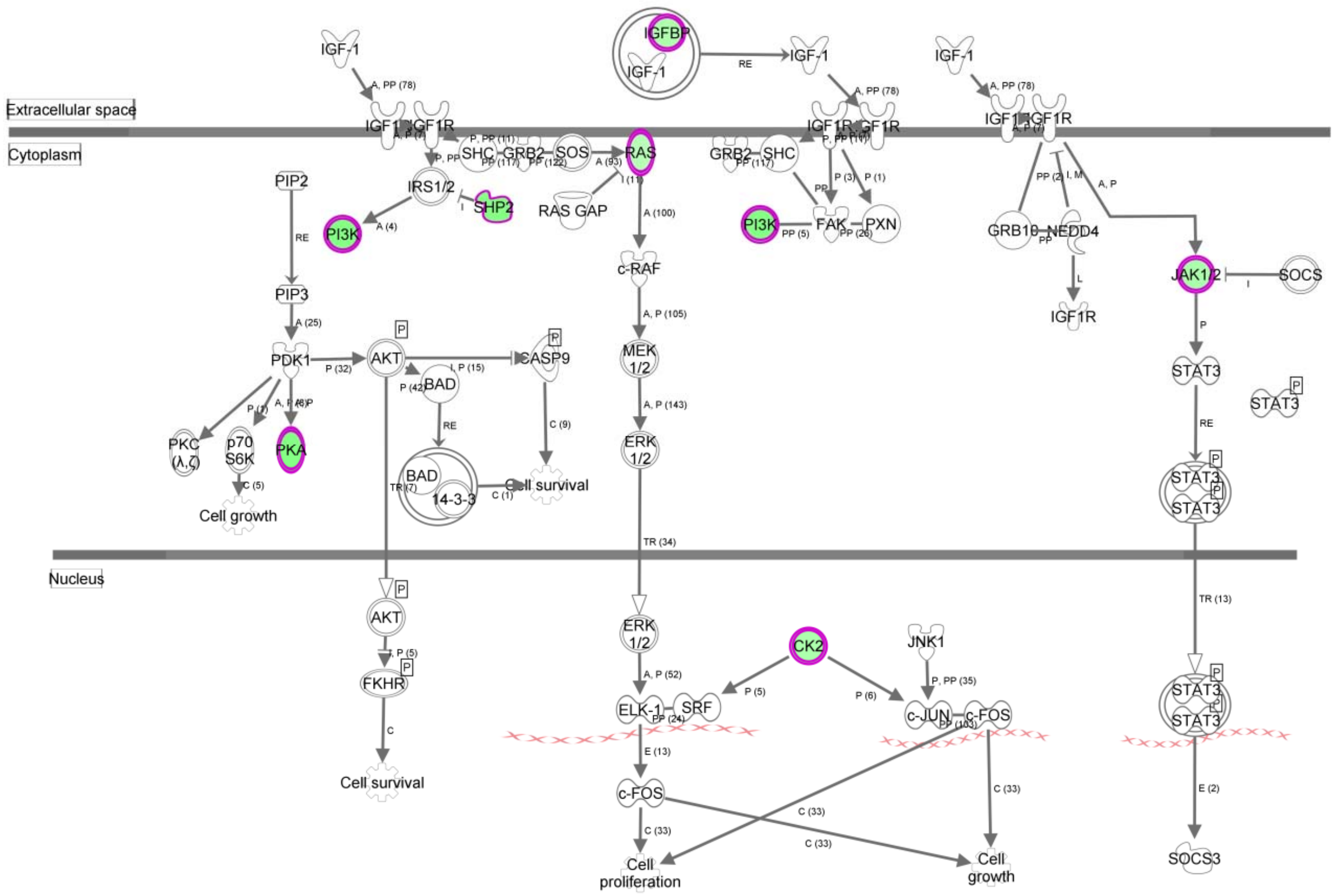

17. IL2 signaling

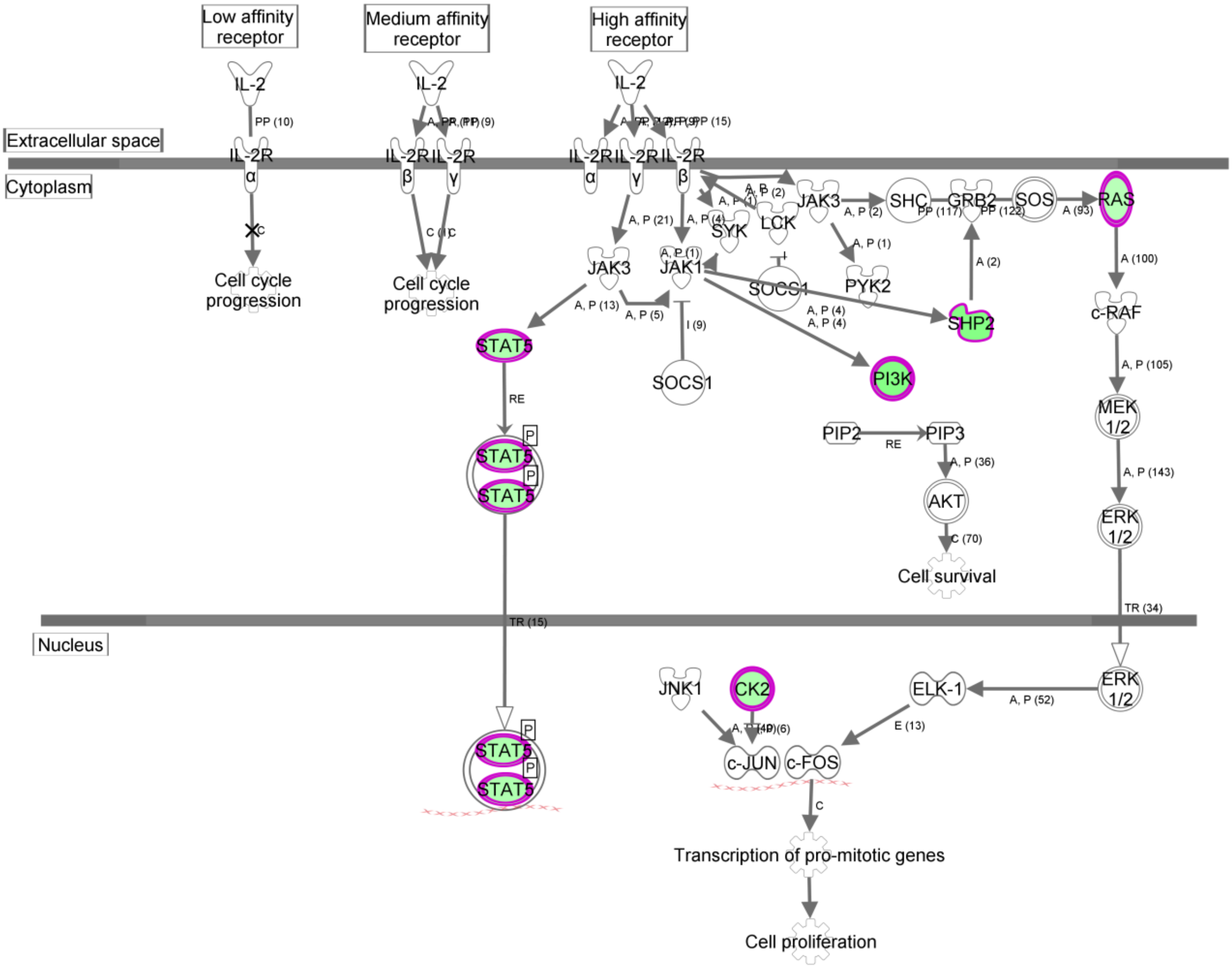

18. IL-8 signaling

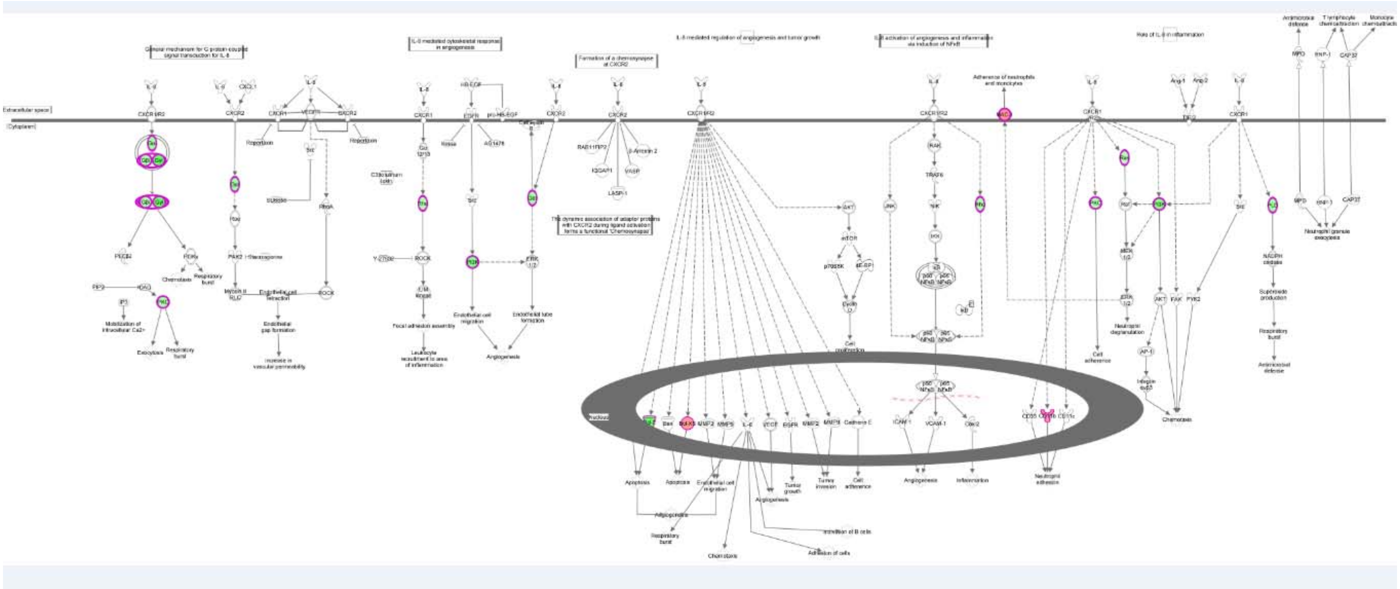

19. Integrin signaling

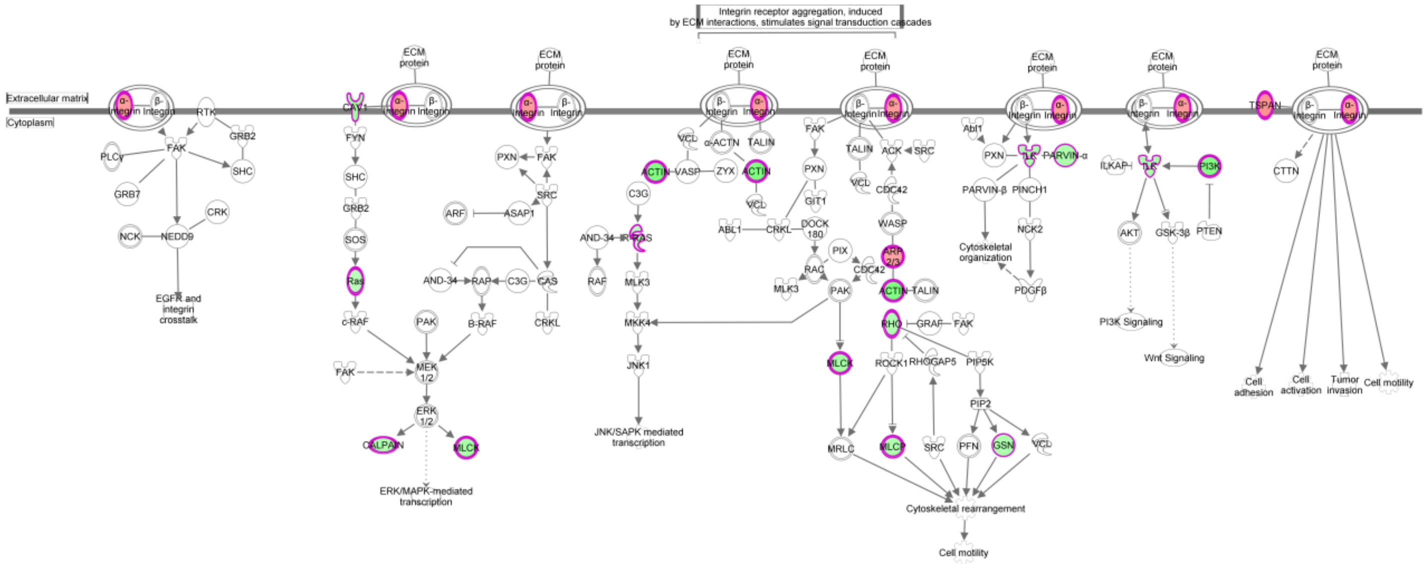

20. mTOR signaling

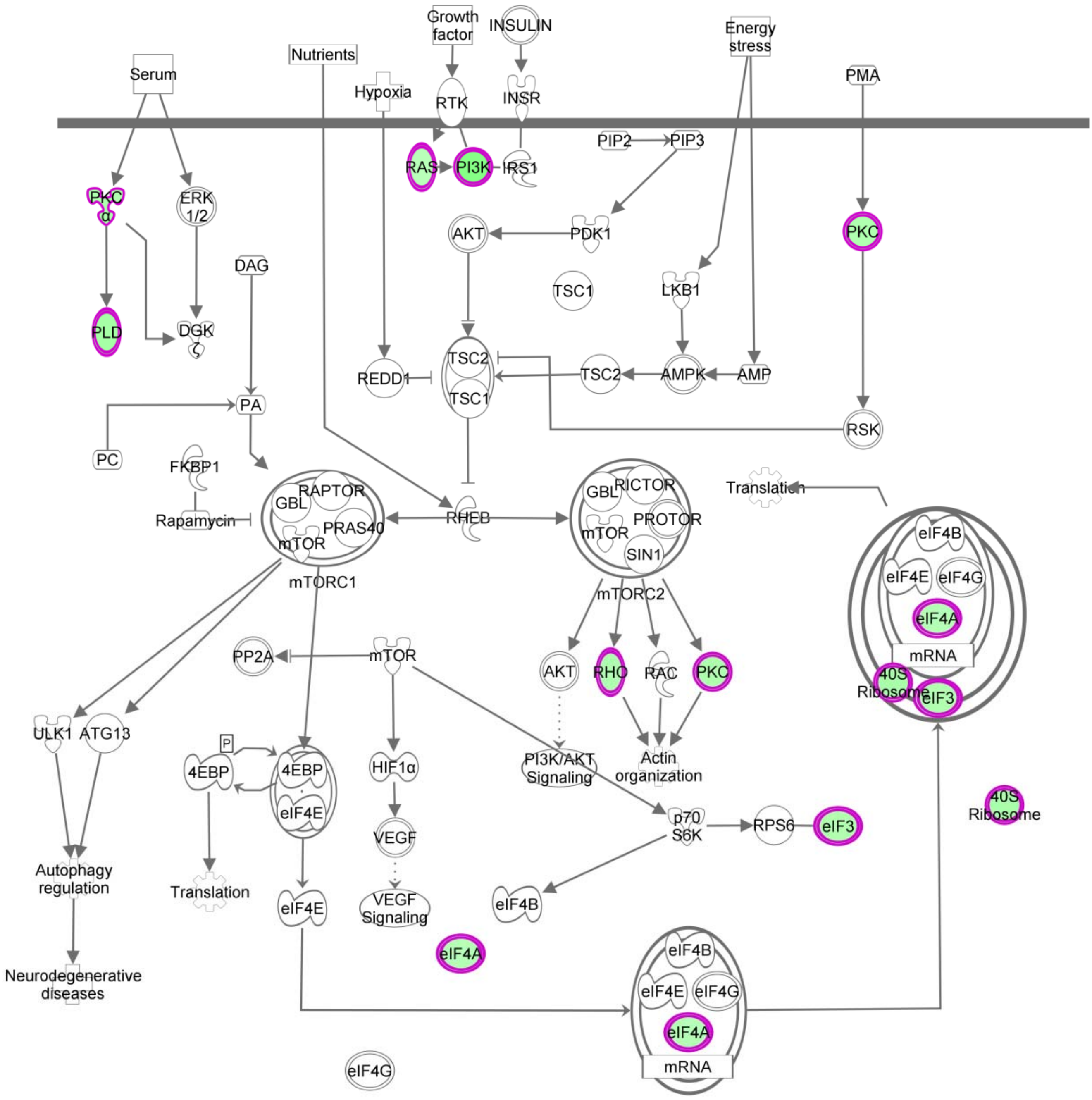

21. Ovarian cancer signaling

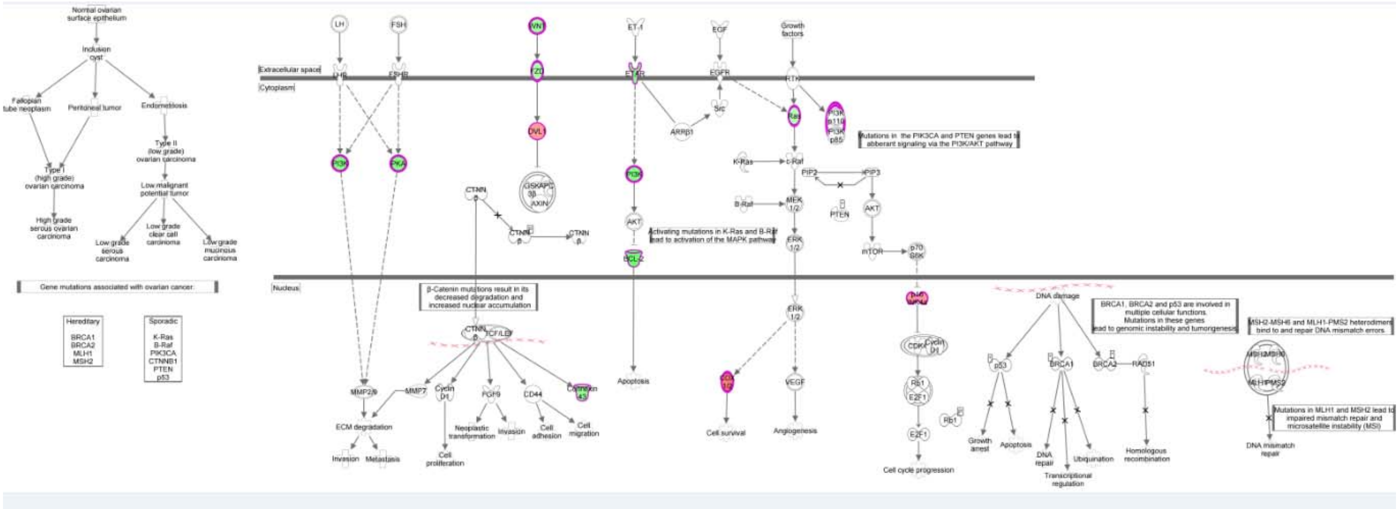

22. Oxidative phosphorylation

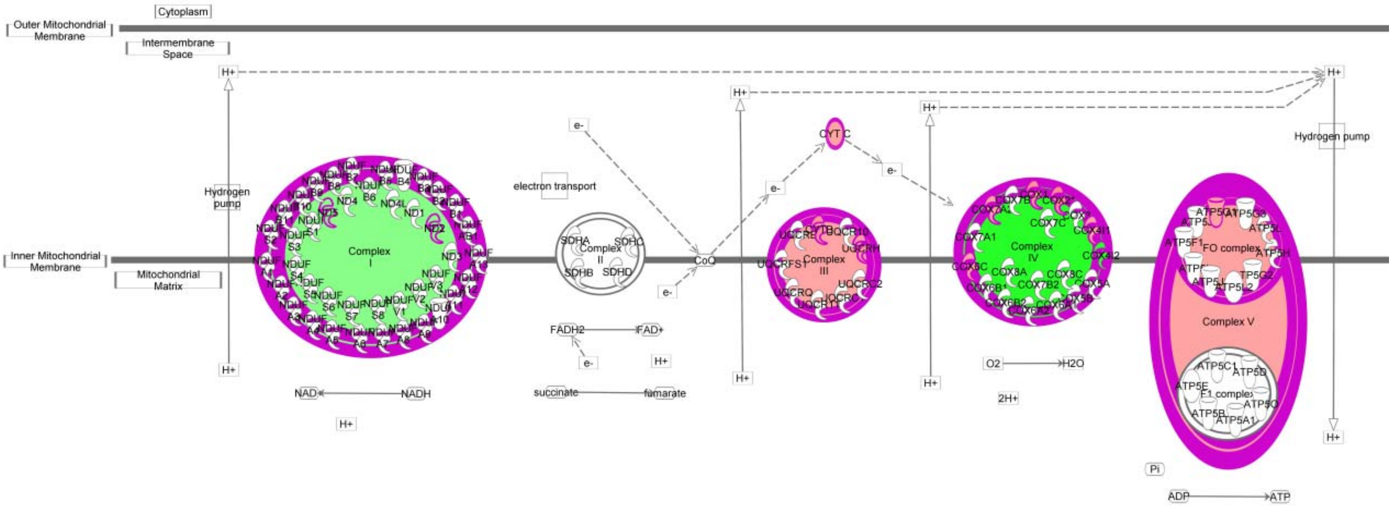

23. P2Y Purigenic receptor signaling pathway

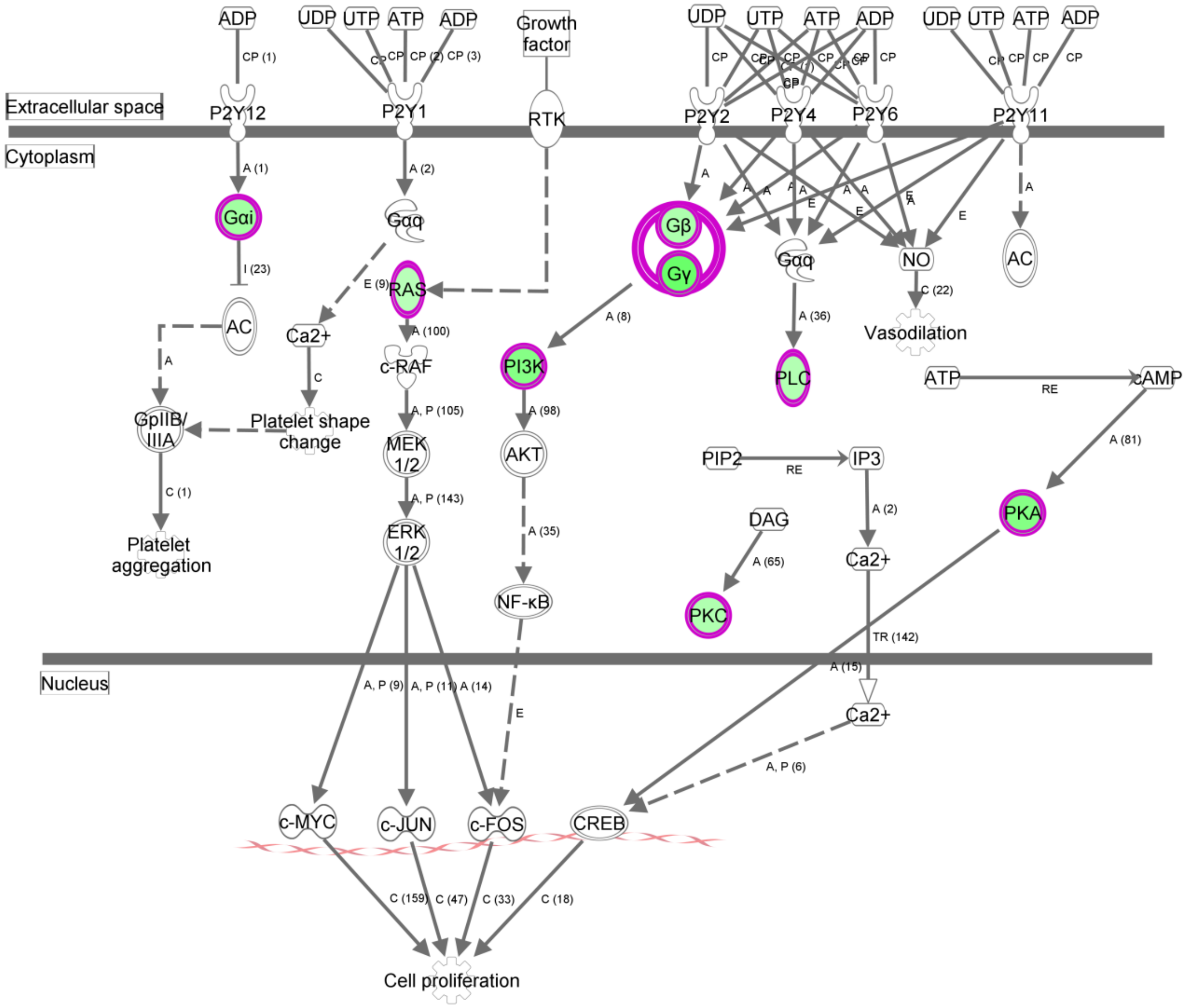

24. Phospholipase c signaling

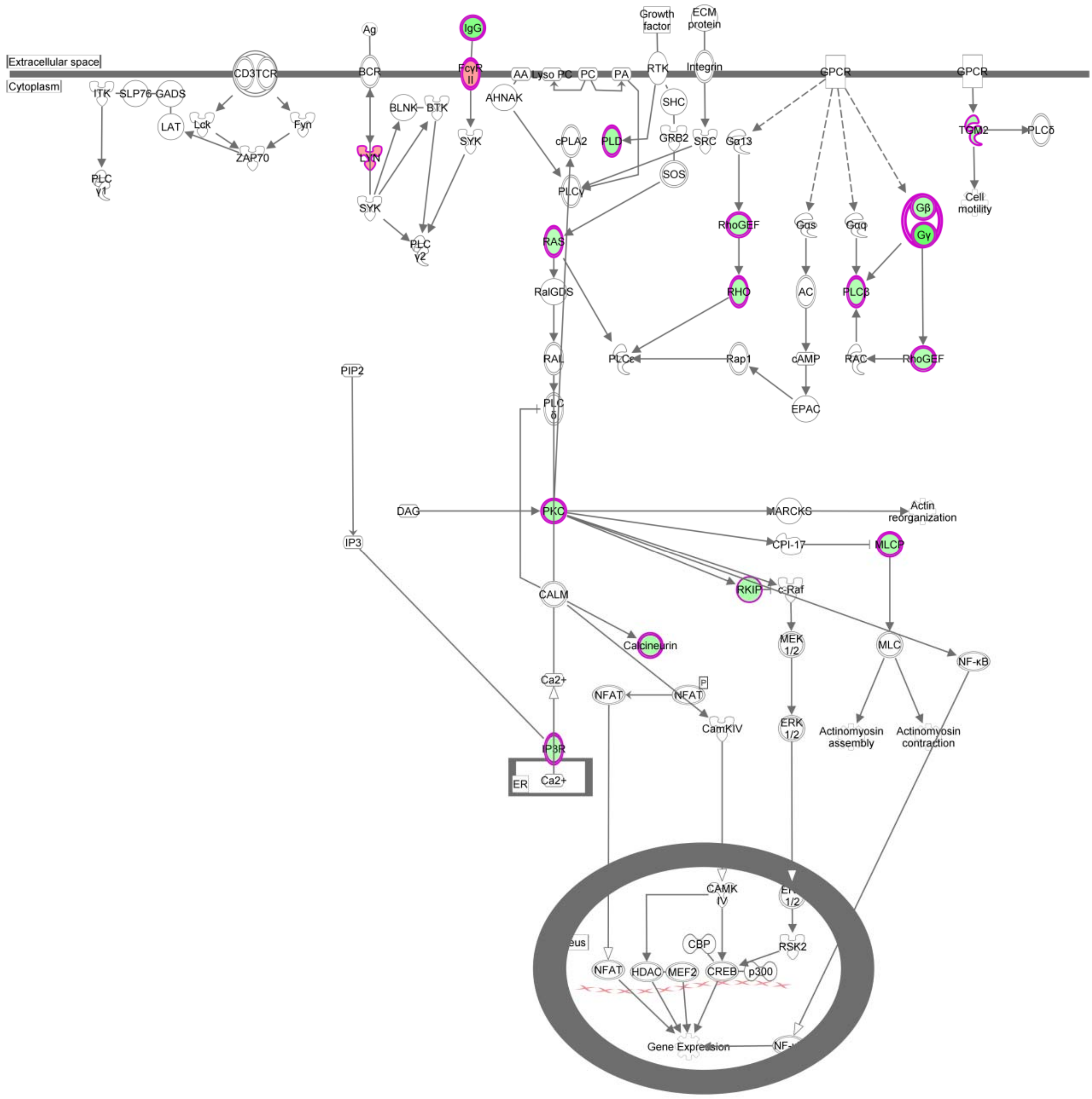

25. Signaling by Rho family GTPases

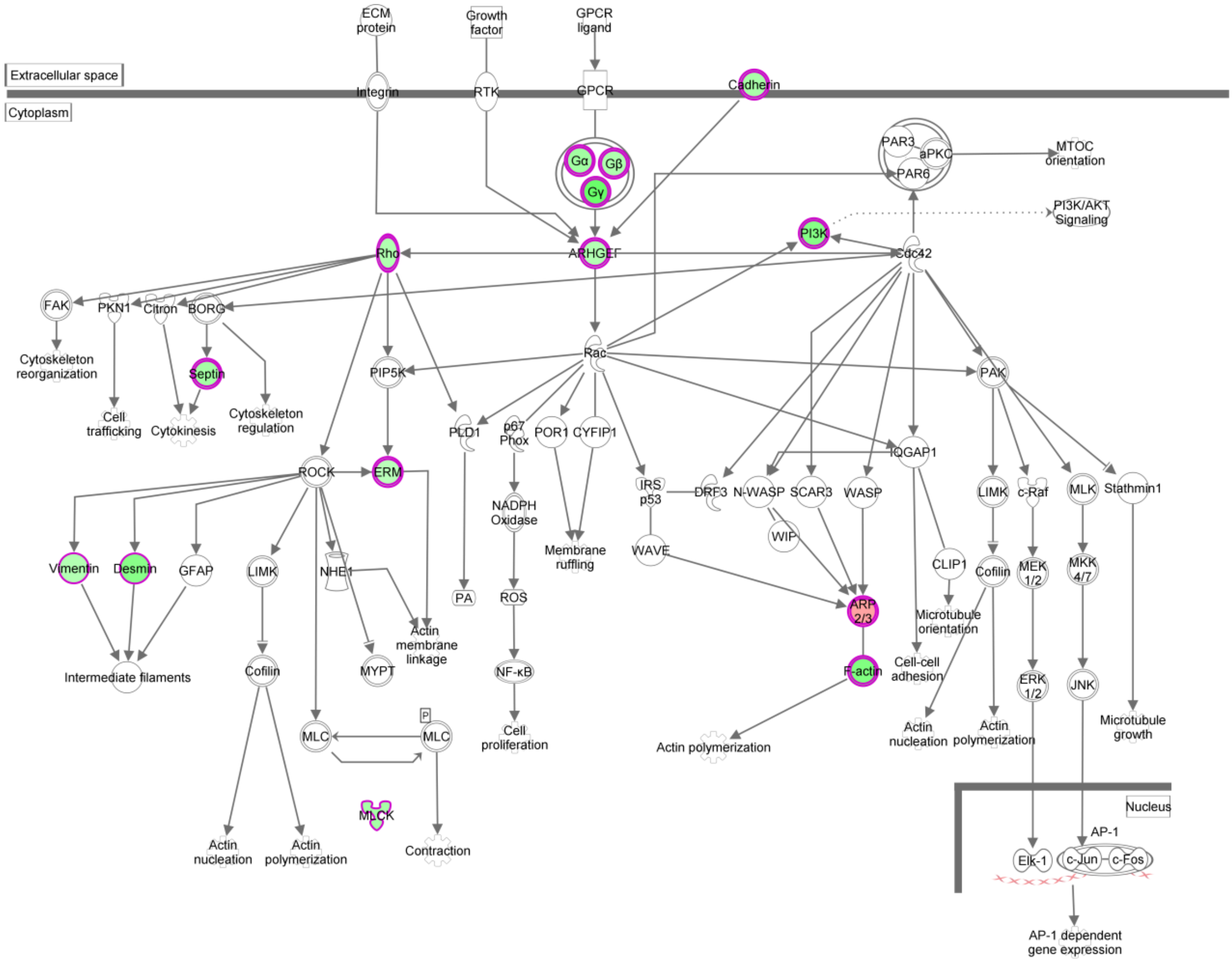

26. Antiproliferative role of somatostatin signaling

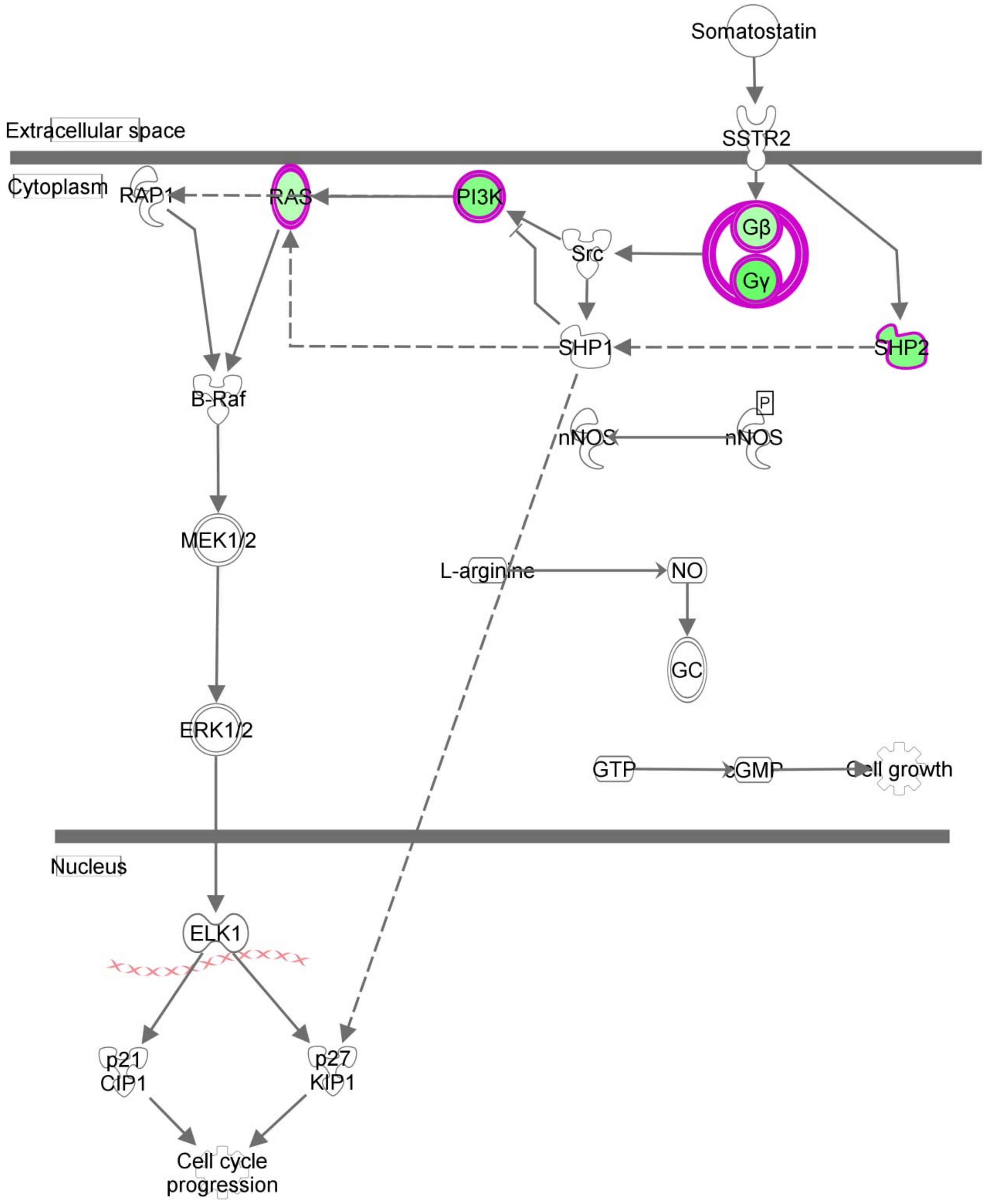

27. Tec kinse signaling

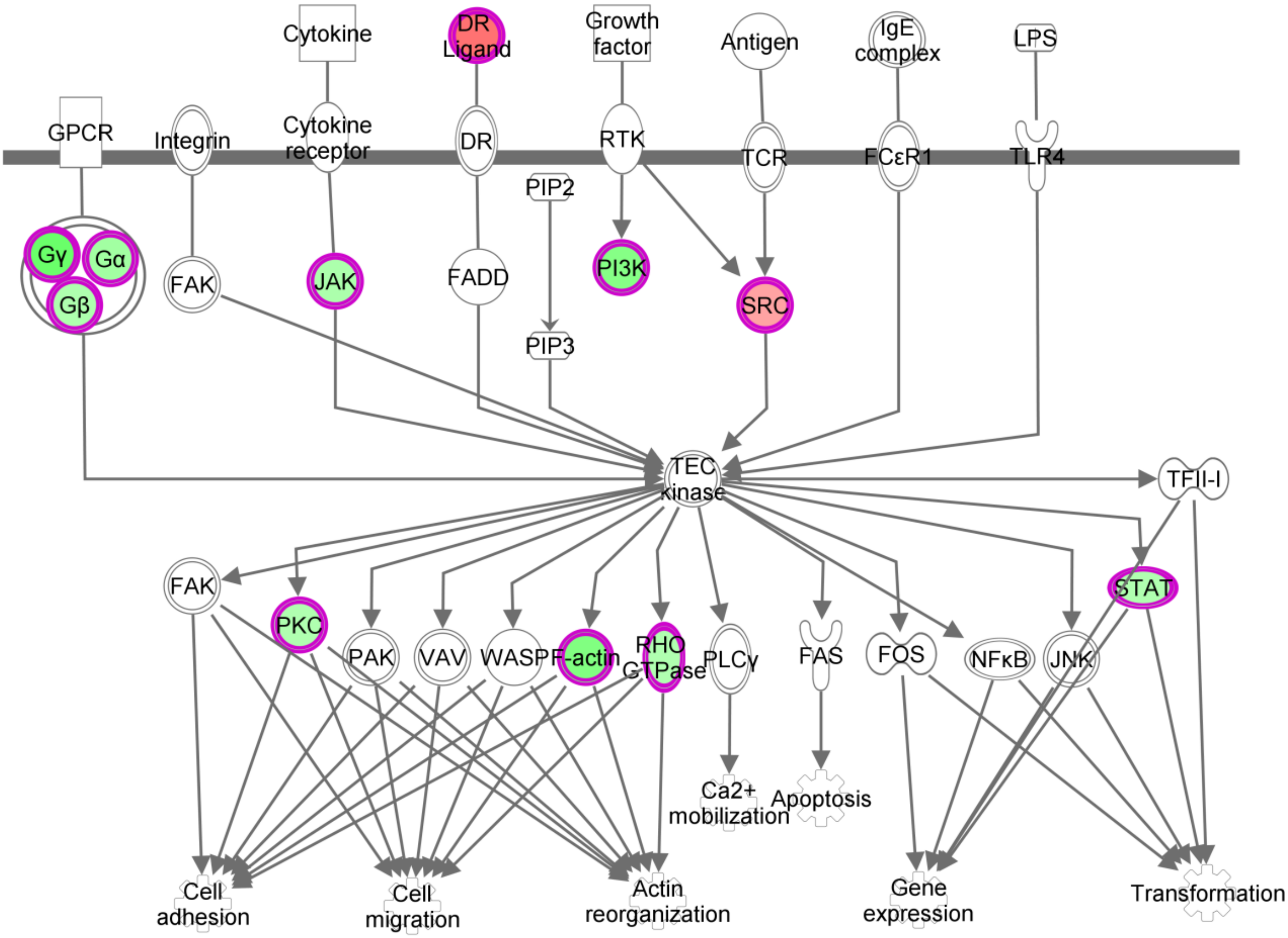

28. Thrombin signaling

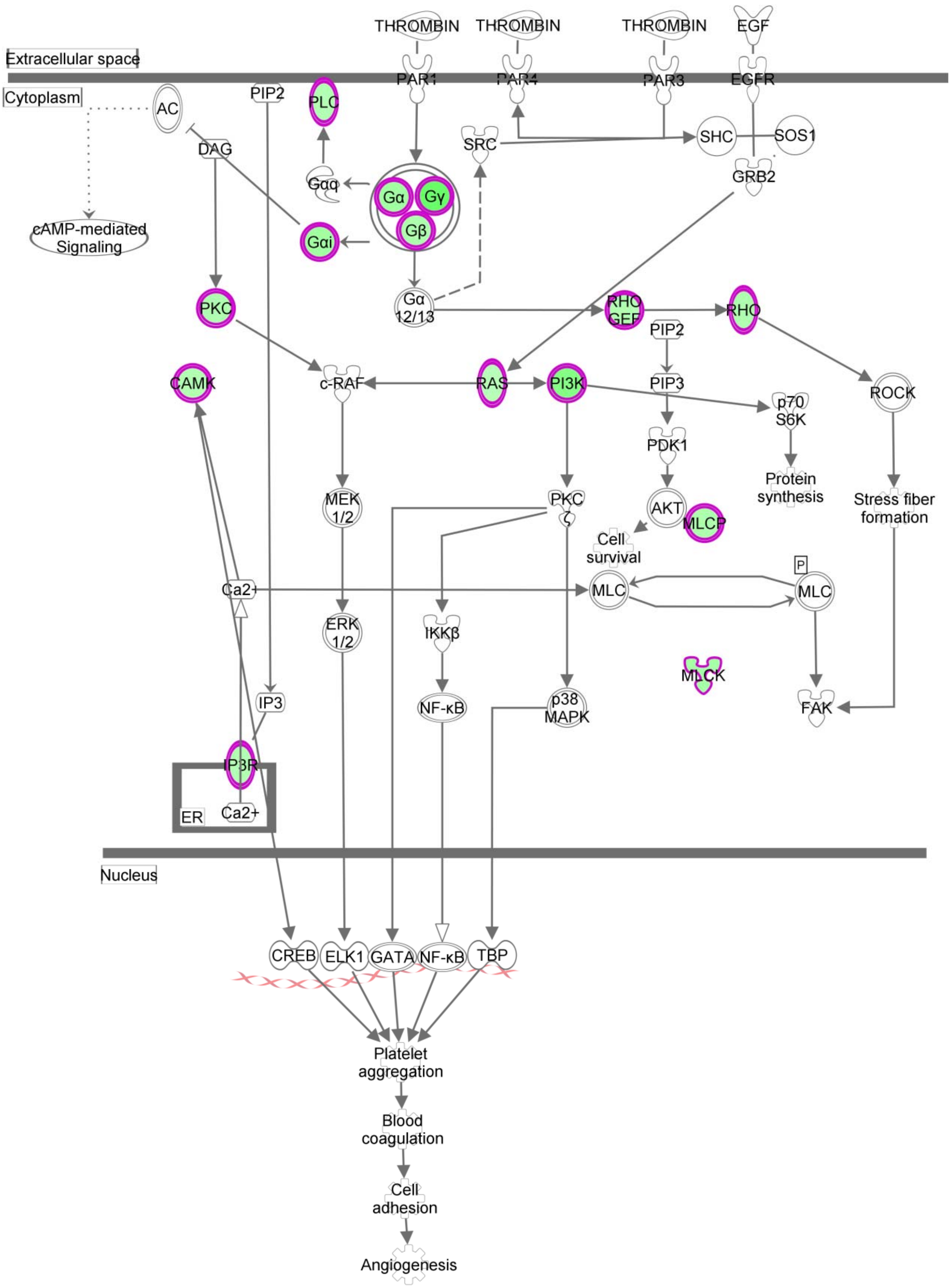

Supplement: Supplementary file 1 — (PDF 5356 kb) [file 13167_2019_170_MOESM1_ESM.pdf]
