# Supplementary material for: Signaling pathway network alterations in human ovarian cancers identified with quantitative mitochondrial proteomics
Source: EPMA J. 2019 Jun 8;10(2):153–72. doi: 10.1007/s13167-019-00170-5 (PMC6562010; doi:10.1007/s13167-019-00170-5)

Supplementary Figure 2

Molecular and cellular functions provide some significant tumor-related molecules.

1. **Cell death and survival (including apoptosis, cell death, cell survival, cell viability, necrosis).**
2. **Cell movement.**
3. **Cell-to-cell signaling and interaction.**
4. **Free radical scavenging.**
5. **Lipid metabolism.**

1.Apoptosis

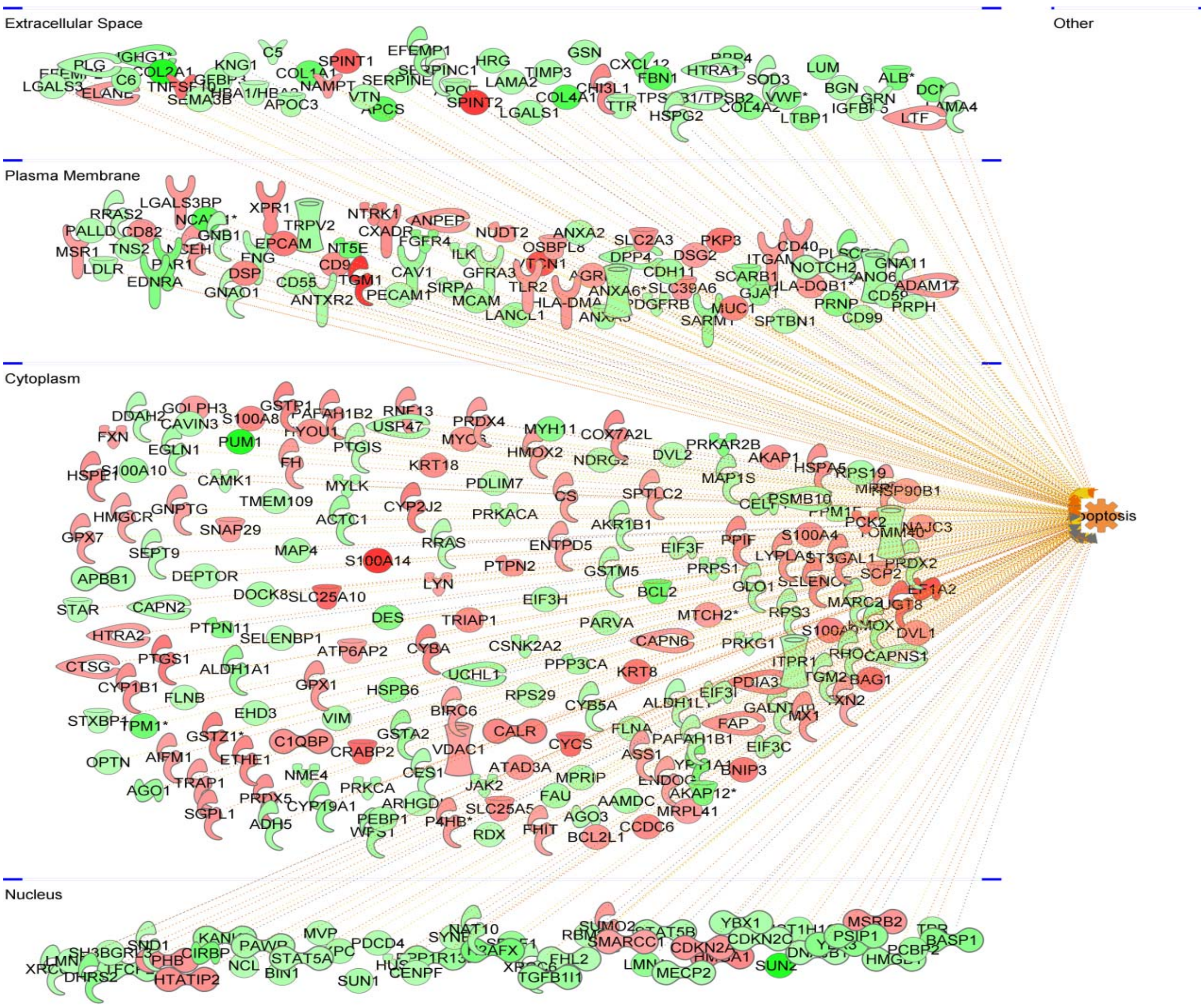

2. Cell death

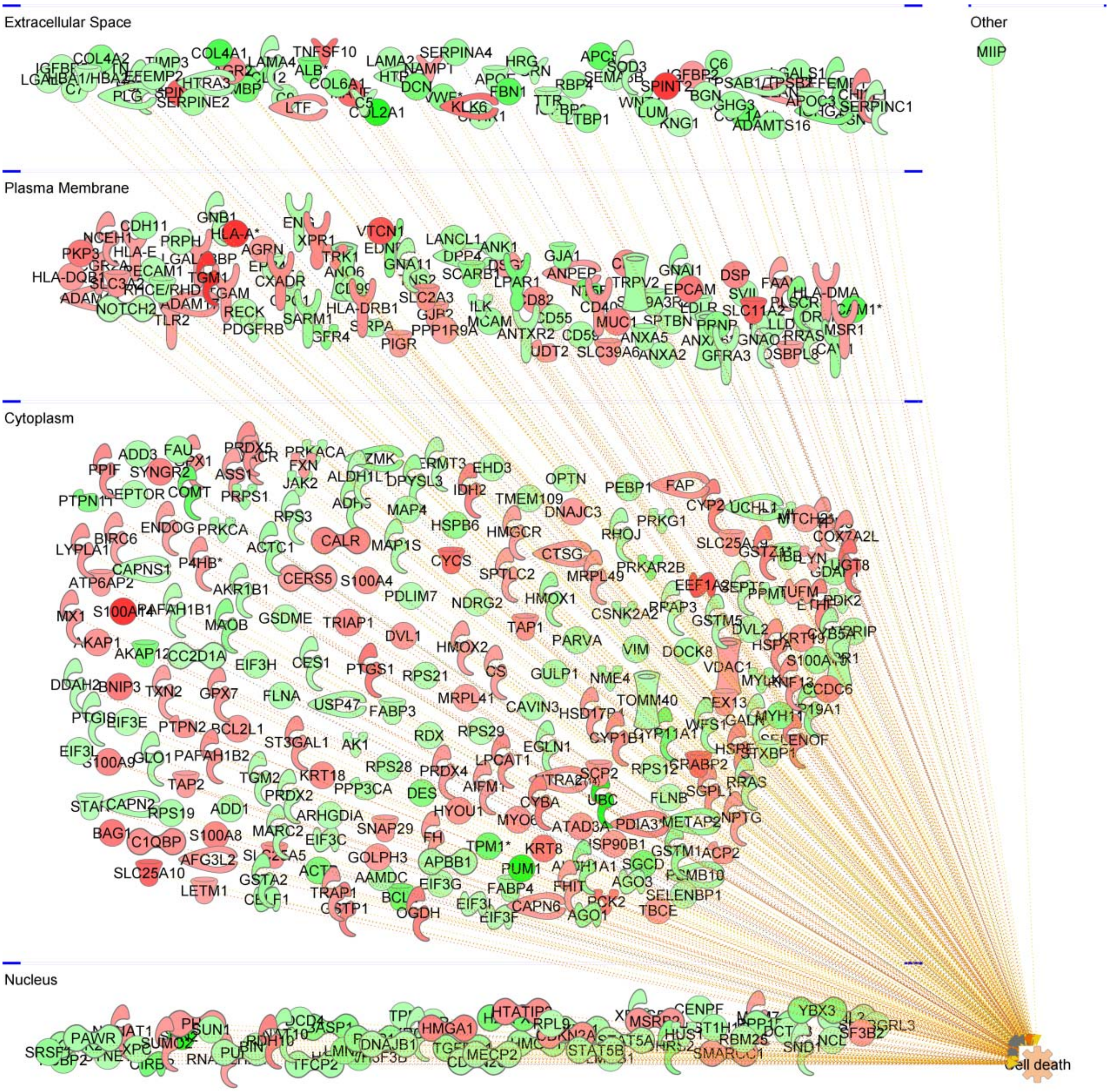

Extracellular Space
Other
MIIP
COL4A1
TNFSF10
SERPINA4
LAMA2
NAMPT
HRG
SOD3
IGFBP2
C6
LGALS1
TIMP3
HTRA3
PLG
SERPINE2
COL6A1
COL2A1
DCN
VWF*
KLK6
FBN1
LTF
RBP4
SPINT2
BGN
LUM
KNG1
LTBP1
ADAMTS16
APOC3
SERPINC1
Plasma Membrane
CDH11
NCEH1
PKP3
HLA-E
HLA-A*
VTCN1
LANCL1
ANK1
GJA1
HLA-DMA
HLA-DQB1
SLC3A2
PECAM1
TGM1
AGRN
XPR1
TRK1
ANO6
GNA11
DPP4
SCARB1
LPAR1
ANPEP
TRPV2
GNAI1
EPCAM
DSP
NOTCH2
TLR2
RECK
CXADR
PDGFRB
SARM1
HLA-DRB1
SLC2A3
PPP1R9A
PIGR
MCAM
ILK
CD82
CD55
ANTXR2
MUC1
CD59
SLC39A6
ANXA5
ANXA2
SPTBN1
PRNP
SLC11A2
GFRA3
RRAS
MSR1
Cytoplasm
ADD3
FAU
PRDX5
PRKACA
ZMK
EHD3
PPIF
SYNGR2
ASS1
FXN
JAK2
ALDH1L1
DPYSL3
IDH2
OPTN
PEBP1
FAP
PTPN11
COMT
PRPS1
MAP4
TMEM109
DNAJC3
PRKG1
UCHL1
RPS3
HSPB6
HMGCR
RHOJ
BIRC6
ENDOG
PRKCA
ACTC1
CALR
MAP1S
CYCS
CTSG
SLC25A
COX7A2L
LYPLA1
CAPNS1
P4HB*
CERS5
S100A4
SPTLC2
MRPL49
PRKAR2B
LYN
UGT8
ATP6AP2
AKR1B1
PDLIM7
NDRG2
HMOX1
EEF1A2
TUFM
PDK2
MX1
S100A14
PAFAH1B1
MAOB
GSDME
TRIAP1
TAP1
CSNK2A2
GSTM5
DVL2
AKAP1
AKAP12
CC2D1A
DVL1
HMOX2
PARVA
VIM
DOCK8
HSPA
EIF3H
CES1
PTGS1
RPS21
CS
GULP1
NME4
VDAC1
DDAH2
BNIP3
TXN2
GPX7
FLNA
USP47
FABP3
MRPL41
CAVIN3
TOMM40
CCDC6
PTGIS
EIF3E
PTPN2
BCL2L1
RDX
RPS29
EIF3L
S100A9
GLO1
PAFAH1B2
ST3GAL1
AK1
EGLN1
CYP1B1
CYP11A1
RPS28
PRDX4
LPCAT1
SCP2
SRABP2
STXBP1
TAP2
TGM2
KRT18
PPP3CA
DES
AIFM1
RPS12
SGPL1
RRAS
PRDX2
CAPN2
RPS19
ADD1
ARHGDIA
HYOU1
MYO6
CYBA
UBC
METAP2
NPTG
BAG1
C1QBP
S100A8
MARC2
SNAP29
FH
ATAD3A
PDIA3*
EIF3C
TPM1*
KRT8
GSTM1
ACP2
AFG3L2
GOLPH3
APBB1
SLC25A10
GSTA2
AAMDC
EIF3G
PUM1
SGCD
PSMB10
LETM1
TRAP1
FABP4
FHIT
AGO3
SELENBP1
OGDH
CAPN6
PCK2
AGO1
TBCE
Nucleus
PAWR
SUN1
HMGA1
HTATIP2
RPL9
MSRB2
CENPF
YBX3
SRSF1
XPC
TFCP2
DNAJB1
MECP2
STAT5A
STAT5B
SMARCC1
RBM25
SND1
SF3B2
Cell death

3. Cell survival

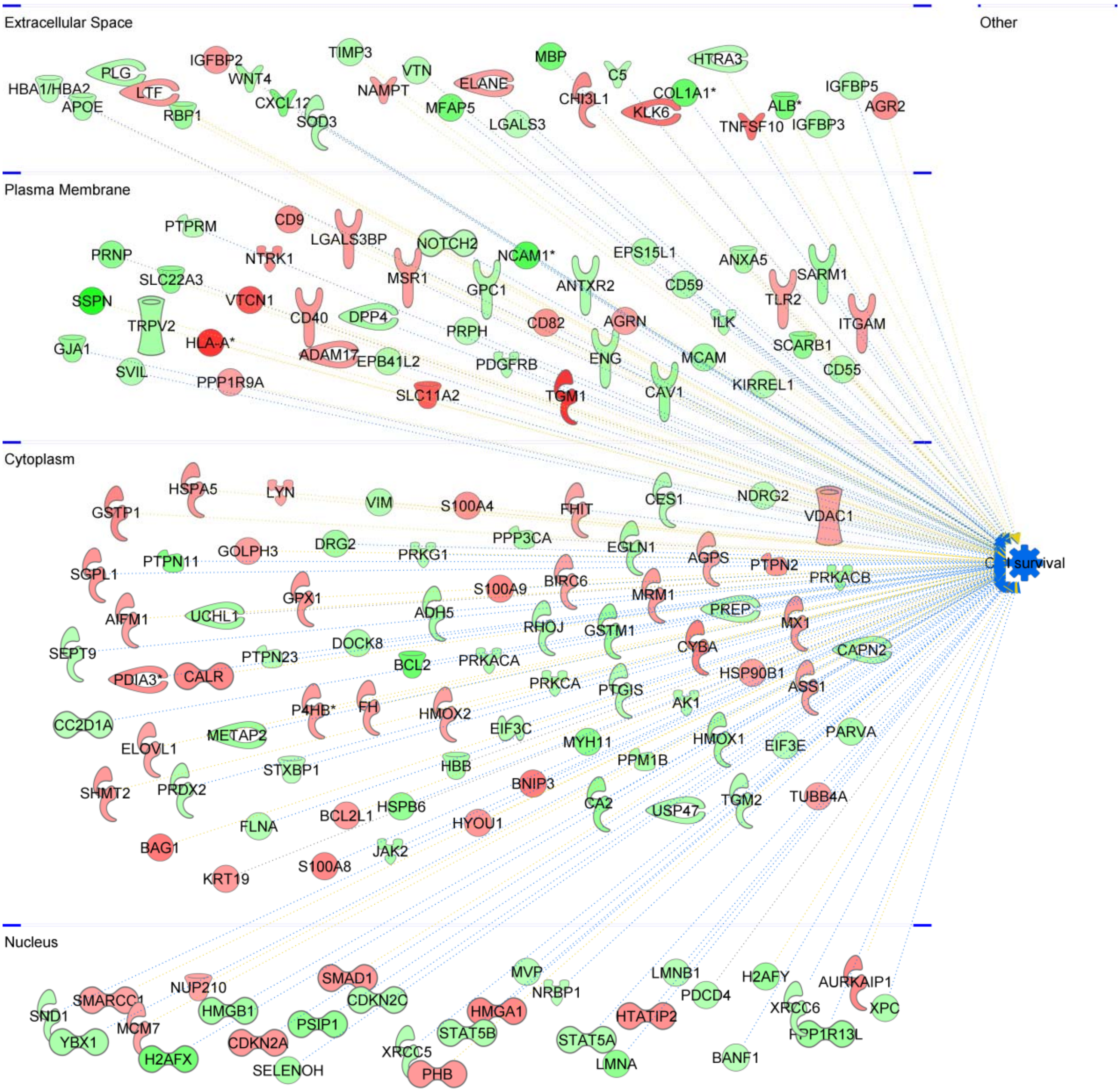

4. Cell viability

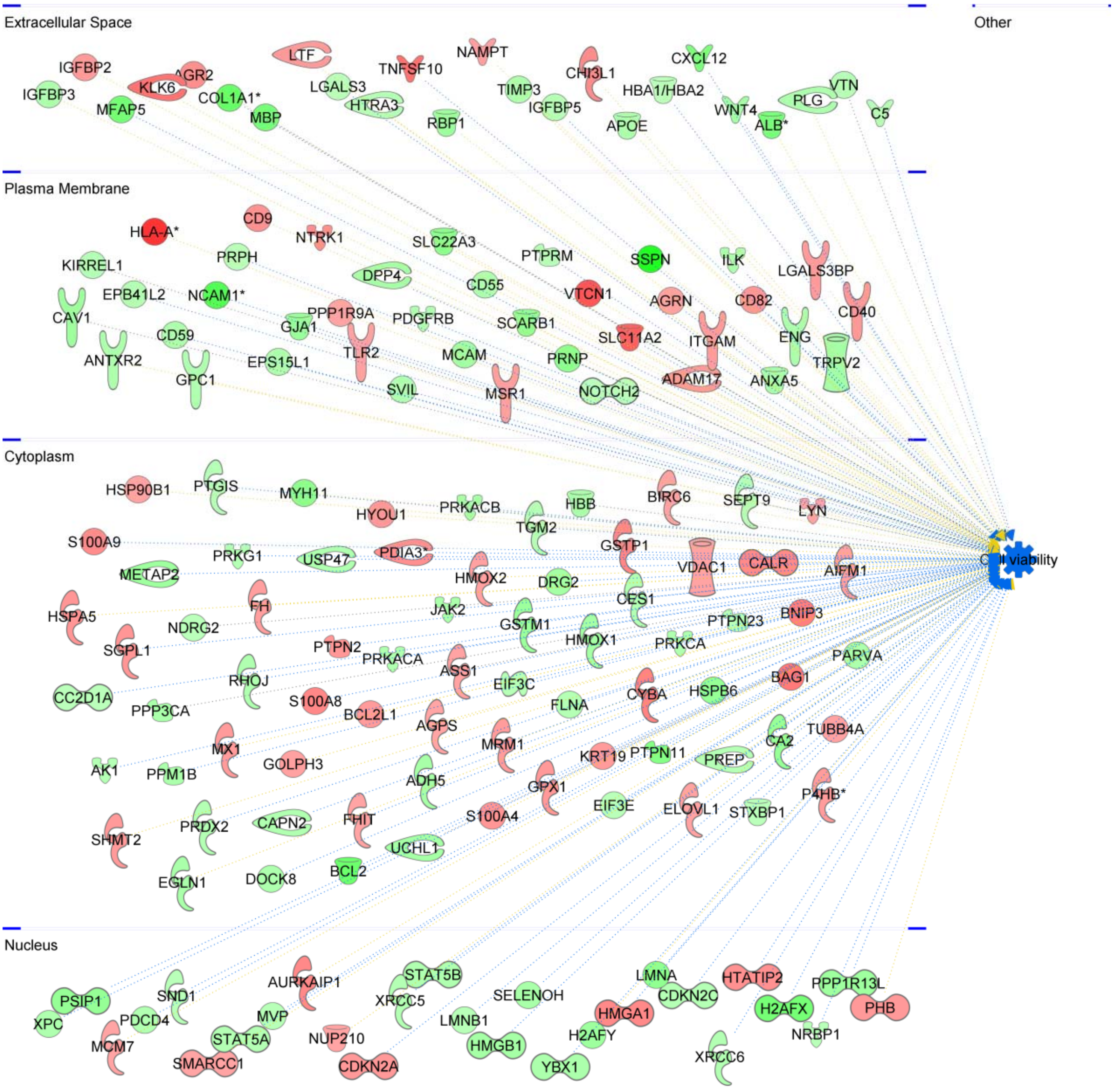

5. Necrosis

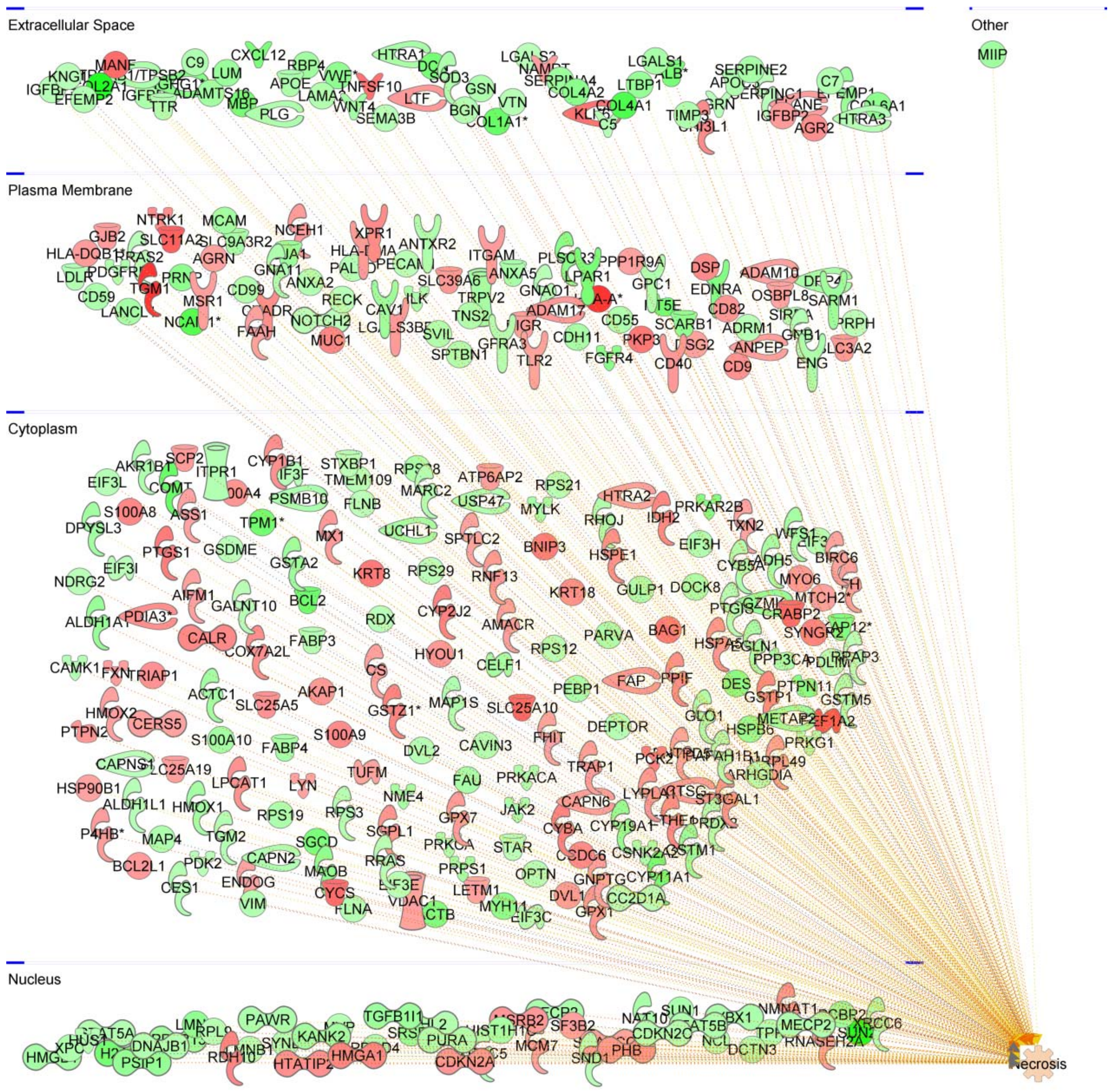
Extracellular Space
Other
MIIP
CXCL12
HTRA1
MANF
C9
LUM
RBP4
APOE
LAMA2
WNT4
SEMA3B
PLG
MBP
TTR
EFEMP2
LTF
BGN
GSN
VTN
COL1A1*
SOD3
COL4A2
COL4A1
C5
LTBP1
LGALS1
TIMP3
SERPINE2
IGFBP2
AGR2
HTRA3
C7
Plasma Membrane
NTRK1
MCAM
GJB2
NCEH1
XPR1
ANTXR2
ITGAM
HLA-DQB1
AGRN
LDLR
CD59
CD99
GNA11
ANXA2
RECK
NOTCH2
FAAH
MUC1
CAV1
SLC39A6
TRPV2
ANXA5
GNAO1
LPAR1
ADAM17
TNS2
SVIL
SPTBN1
GFRA3
TLR2
CDH11
FGFR4
CD55
PKP3
CD40
SCARB1
CD82
DSP
EDNRA
ADAM10
OSBPL8
SARM1
ADRM1
ANPEP
CD9
ENG
PRPH
PPP1R9A
GPC1
MSR1
TGM1
Cytoplasm
AKR1B1
SCP2
ITPR1
CYP1B1
IF3F
STXBP1
TMEM109
ATP6AP2
RPS21
EIF3L
COMT
PSMB10
FLNB
MARC2
USP47
MYLK
HTRA2
PRKAR2B
S100A8
ASS1
TPM1*
IDH2
RHOJ
DPYSL3
UCHL1
MX1
SPTLC2
BNIP3
HSPE1
TXN2
EIF3H
PTGS1
GSDME
GSTA2
EIF3I
KRT8
RPS29
RNF13
NDRG2
KRT18
GULP1
DOCK8
AIFM1
GALNT10
BCL2
CYP2J2
ALDH1A1
PDIA3*
RDX
AMACR
PARVA
BAG1
CALR
FABP3
COX7A2L
HYOU1
RPS12
CAMK1
FXN
TRIAP1
CELF1
FAP
PPIF
CS
ACTC1
SLC25A5
AKAP1
PEBP1
DES
PTPN11
GSTM5
MAP1S
SLC25A10
HMOX2
CERS5
GSTZ1*
PTPN2
S100A10
S100A9
FABP4
DVL2
CAVIN3
DEPTOR
FHIT
PRKG1
CAPNS1
SLC25A19
TUFM
TRAP1
HSP90B1
LPCAT1
LYN
FAU
PRKACA
ALDH1L1
HMOX1
NME4
CAPN6
RPS19
RPS3
GPX7
JAK2
P4HB*
MAP4
TGM2
SGPL1
CYBA
SGCD
PRKCA
STAR
BCL2L1
PDK2
CAPN2
MAOB
RRAS
PRPS1
OPTN
CSNK2A2
GSTM1
CES1
ENDOG
CYCS
EIF3E
LETM1
GNPTG
CYP11A1
VIM
FLNA
VDAC1
MYH11
DVL1
CC2D1A
EIF3C
GPX1
Nucleus
PAWR
TGFB1I1
NAT10
SUN1
NMNAT1
HMGB1
HUS1
PSIP1
DNAJB1
RPL9
RDH10
HTATIP2
KANK2
HMGA1
PURA
CDKN2A
MCM7
SF3B2
SND1
PHB
CDKN2C
DCTN3
MECP2
XRCC6
Necrosis

6. Cell movement

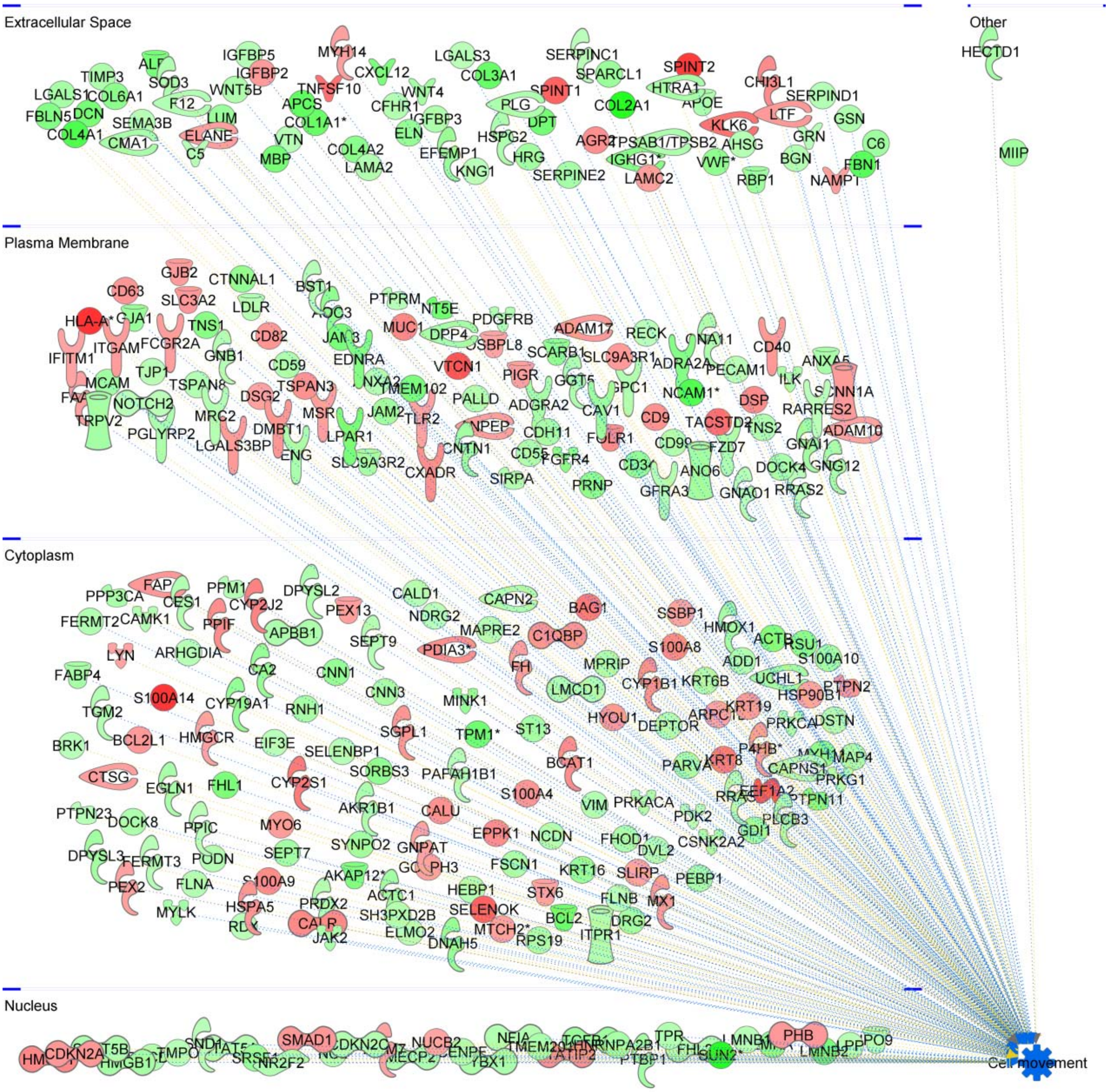

7. Cell-to-cell signaling and interaction

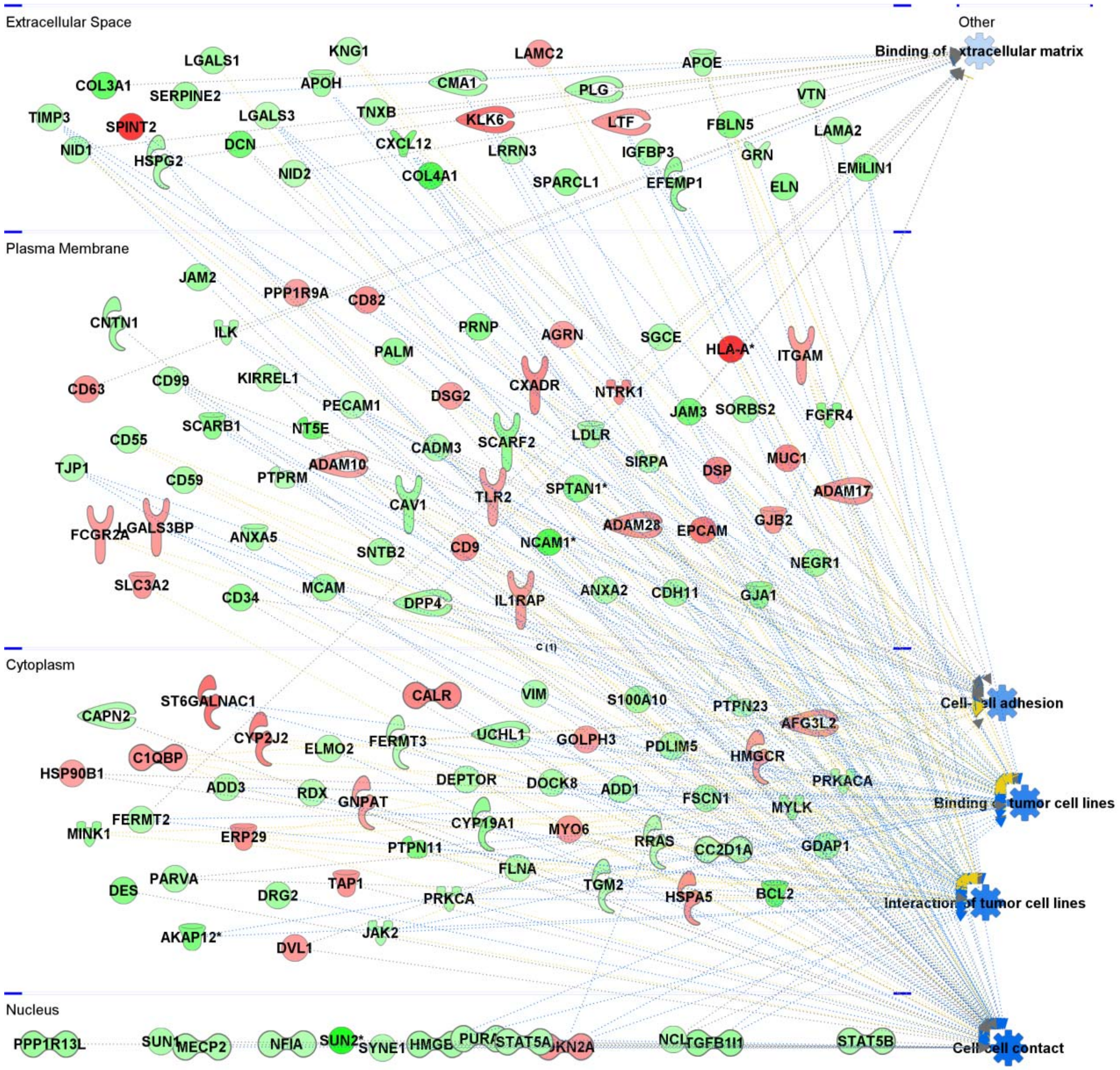

8. Free radical scavenging

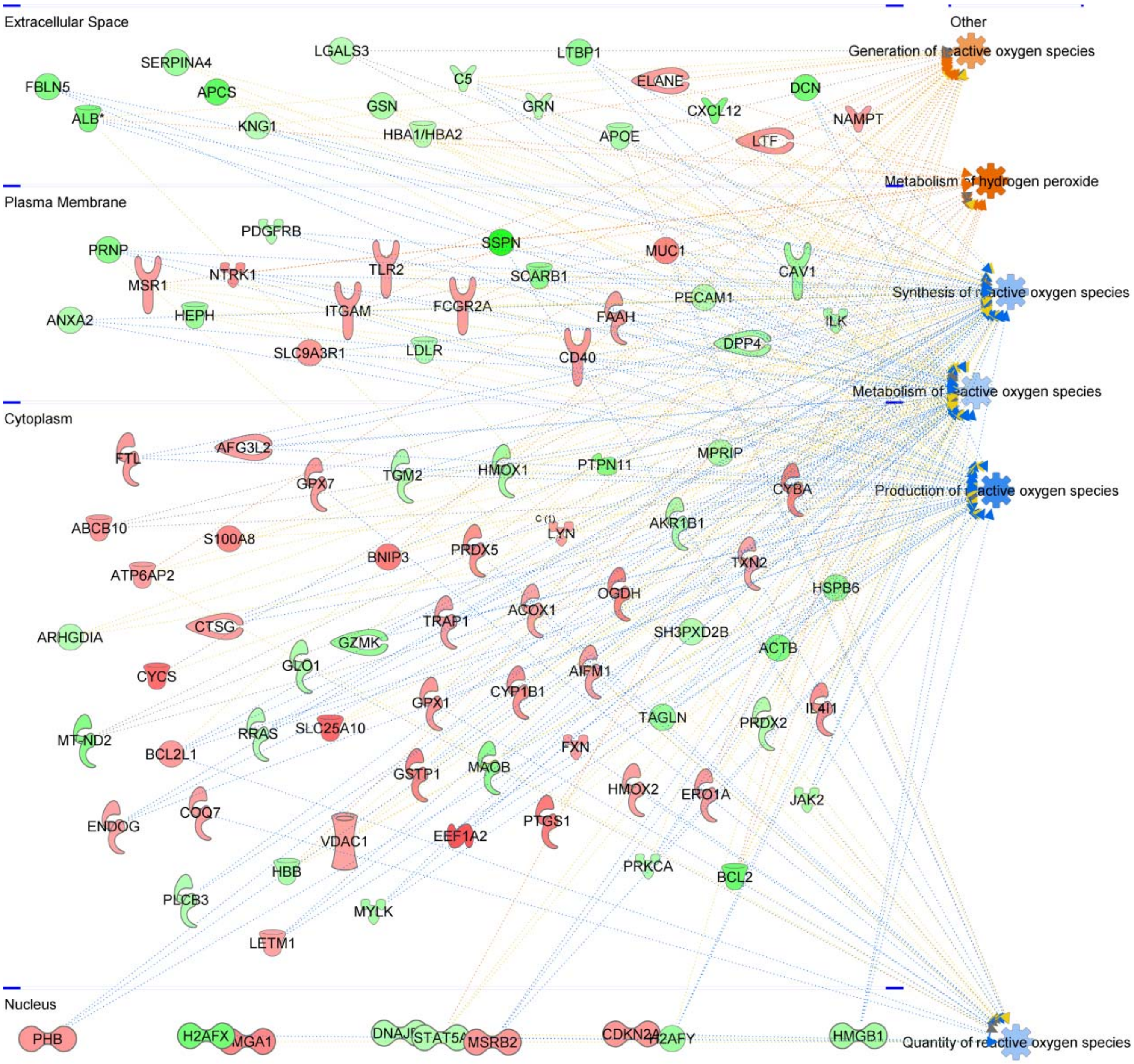

9. Lipid metabolism

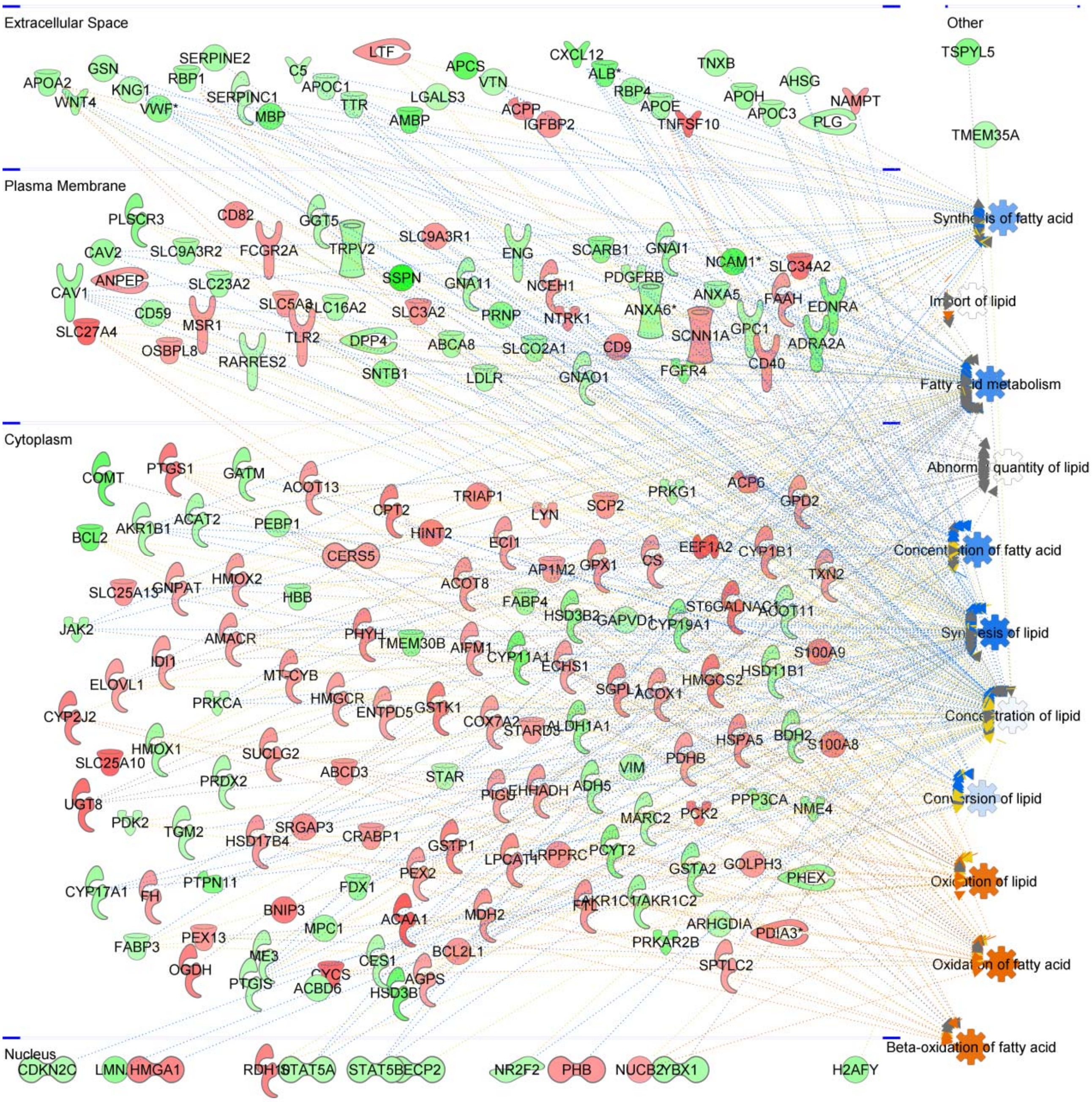

Supplement: Supplementary file 2 — (PDF 3550 kb) [file 13167_2019_170_MOESM2_ESM.pdf]
