# Supplementary material for: Signaling pathway network alterations in human ovarian cancers identified with quantitative mitochondrial proteomics
Source: EPMA J. 2019 Jun 8;10(2):153–72. doi: 10.1007/s13167-019-00170-5 (PMC6562010; doi:10.1007/s13167-019-00170-5)

Supplementary Figure 3

A total of 25 networks were identified to involve ovarian cancer mitochondrial differentially expressed proteins.

1. Cell morphology, cellular movement, connective tissue disorders.
2. Amino acid metabolism, small molecule biochemistry, post-translation modification.
3. Post-translation modification, protein folding, hereditary disorder.
4. Metabolic disease, neurological disease, organismal injury and abnormalities.
5. Developmental disorder, hereditary disorder, metabolic disease.
6. Post-translation modification, protein folding, hereditary disorder.
7. Cancer, connective tissue disorders, organismal injury and abnormalities.
8. Cellular assembly and organization, cellular function and maintenance, tissue development.
9. Cellular assembly and organization, cellular function and maintenance, development disorder.
10. Protein synthesis, cell morphology, cellular compromise.
11. Cell death and survival, embryonic development, cell morphology.
12. Gene expression, protein synthesis, cancer.
13. Hair and skin development and function, post-translational modification, protein folding.
14. Small molecule biochemistry, cancer, cellular development.
15. Hematological disease, infectious disease, molecular transport.
16. Post-translational modification, developmental disorder, hereditary disorder.
17. Drug metabolism, protein synthesis, small molecule biochemistry.
18. Connective tissue disorders, developmental disorder, hereditary disorder.
19. Nucleic acid metabolism, small molecule biochemistry, cancer.
20. Tissue development, connective tissue disorders, dermatological diseases and conditions.
21. Nucleic acid metabolism, small molecule, biochemistry, DNA replication, recombination, and repair.
22. Neurological disease, organismal injury and abnormalities, cell death and survival.
23. Cardiovascular disease, organismal injury and abnormalities, hematological disease.
24. Cellular development, cellular growth and proliferation, embryonic development.
25. Development disorder, hereditary disorder, neurological disease.

1. **Cell morphology, cellular movement, connective tissue disorders.**

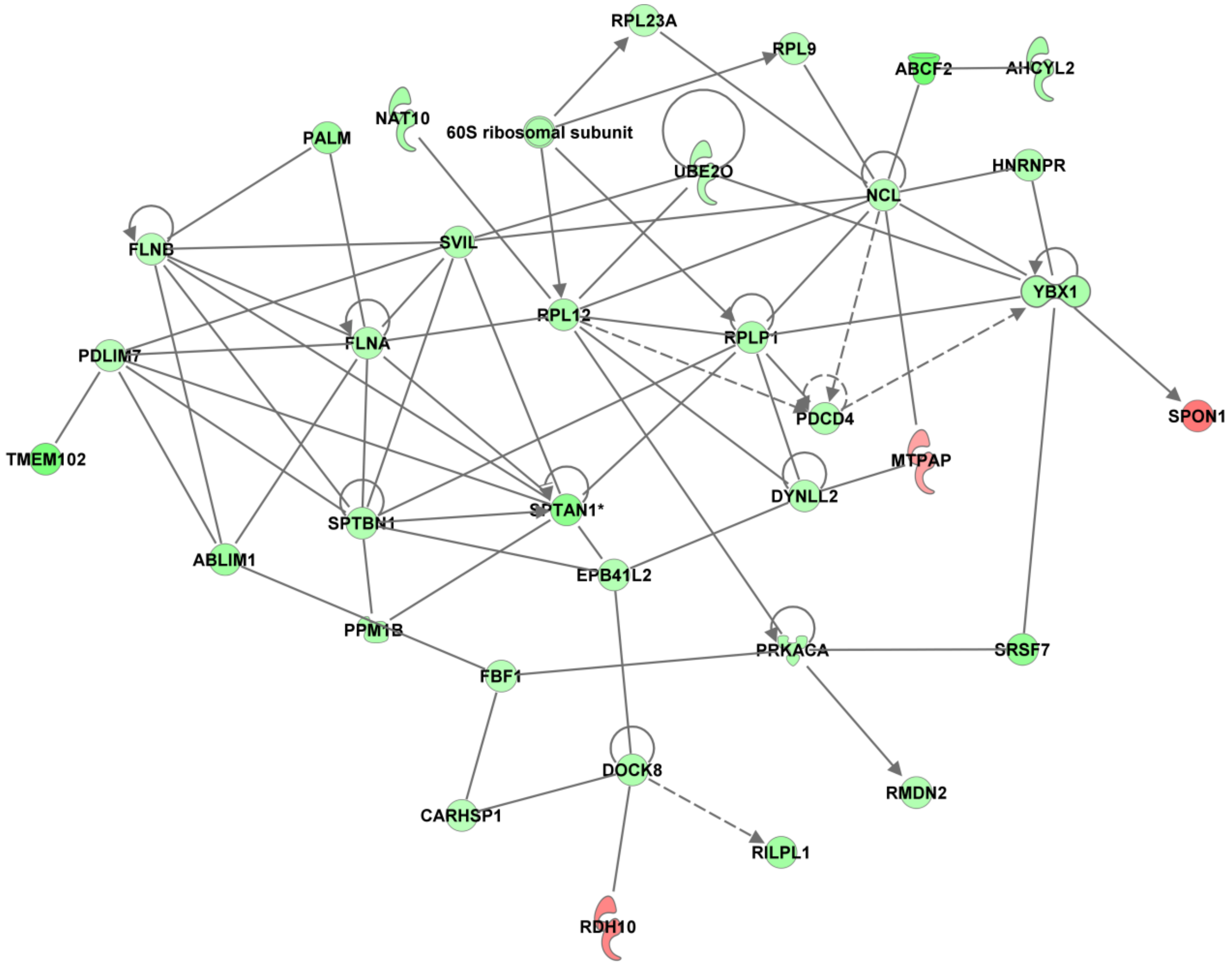

2. **Amino acid metabolism, small molecule biochemistry, post-translation modification.**

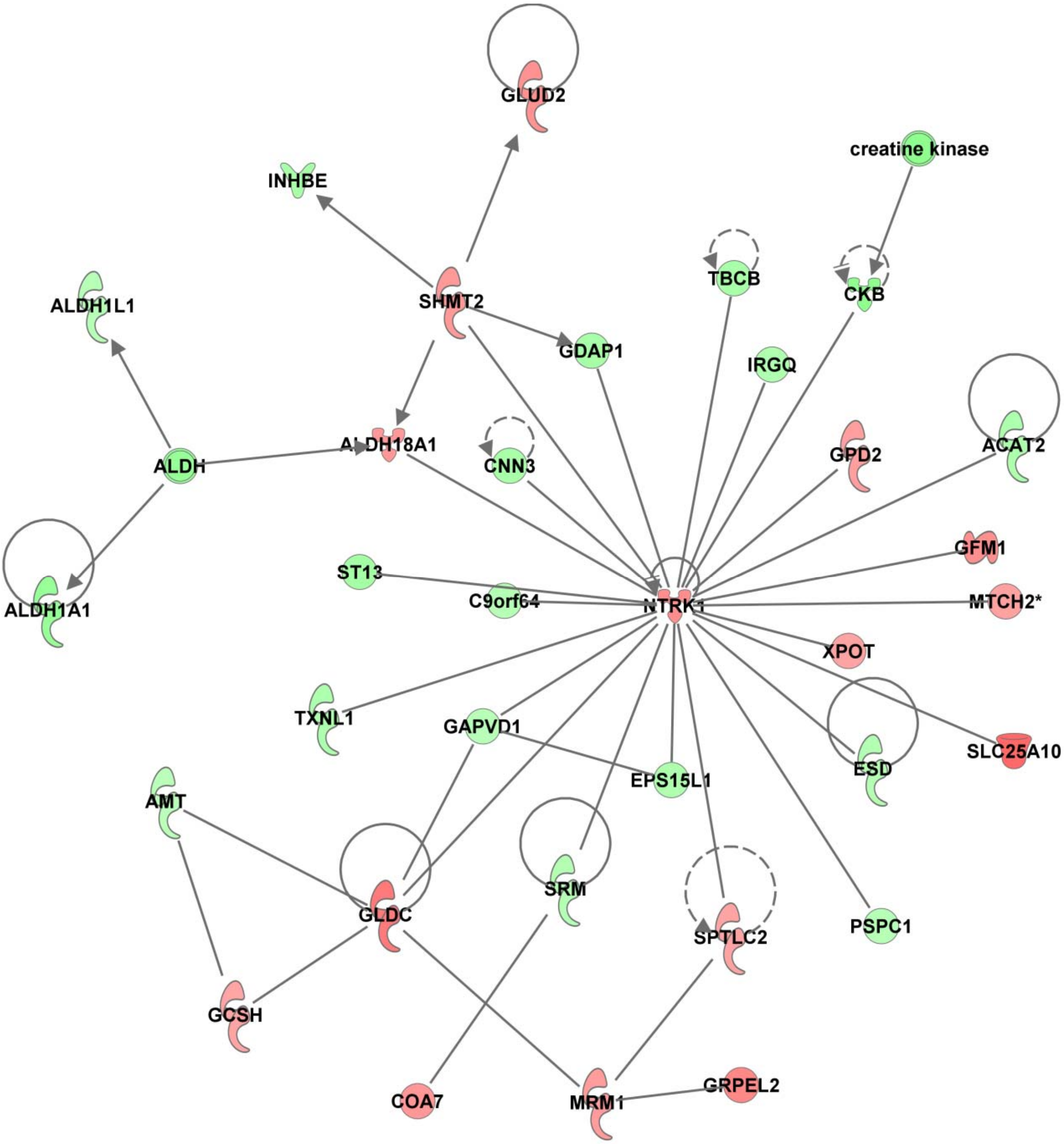

3. Post-translation modification, protein folding, hereditary disorder.

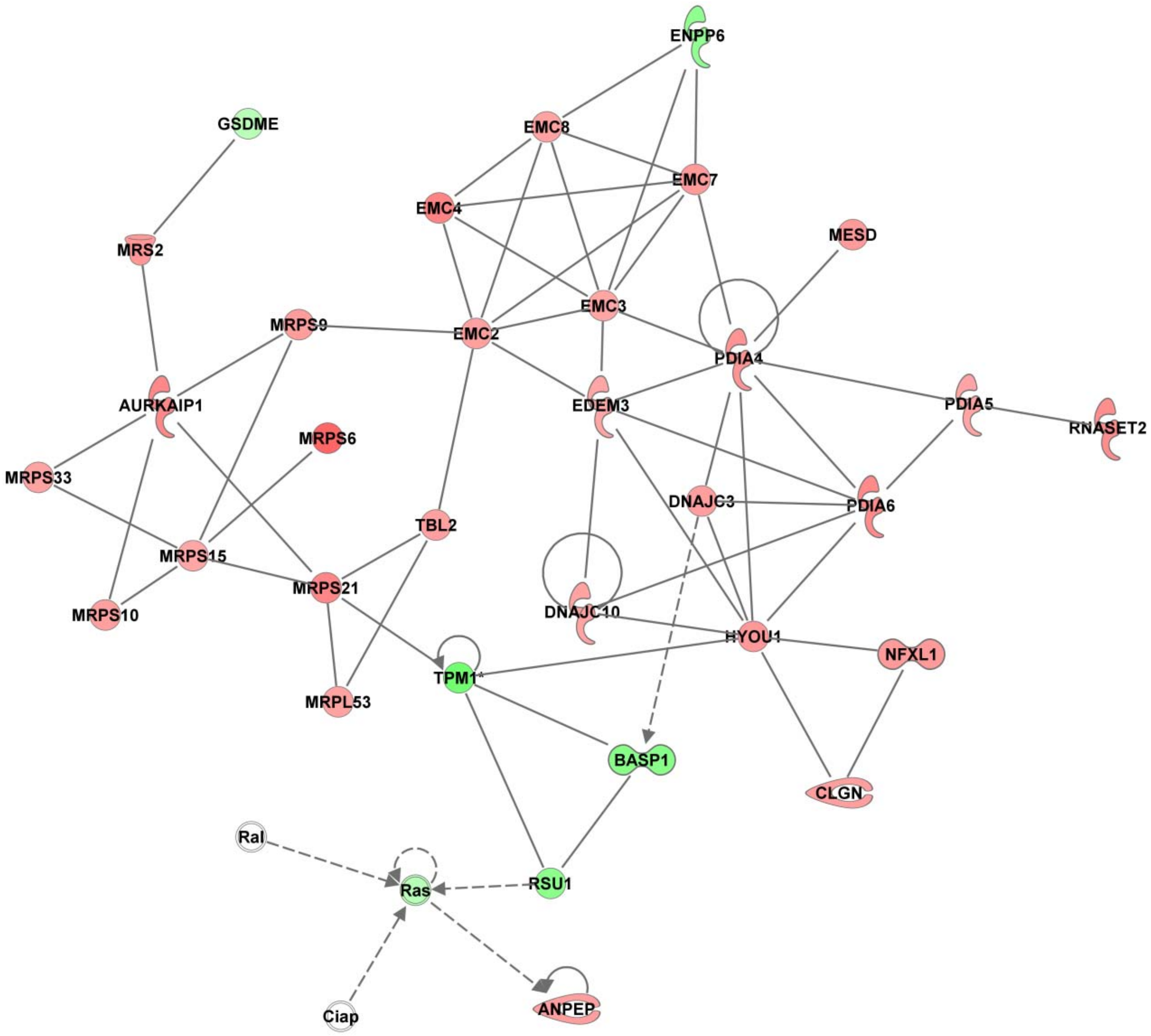

4. Metabolic disease, neurological disease, organismal injury and abnormalities.

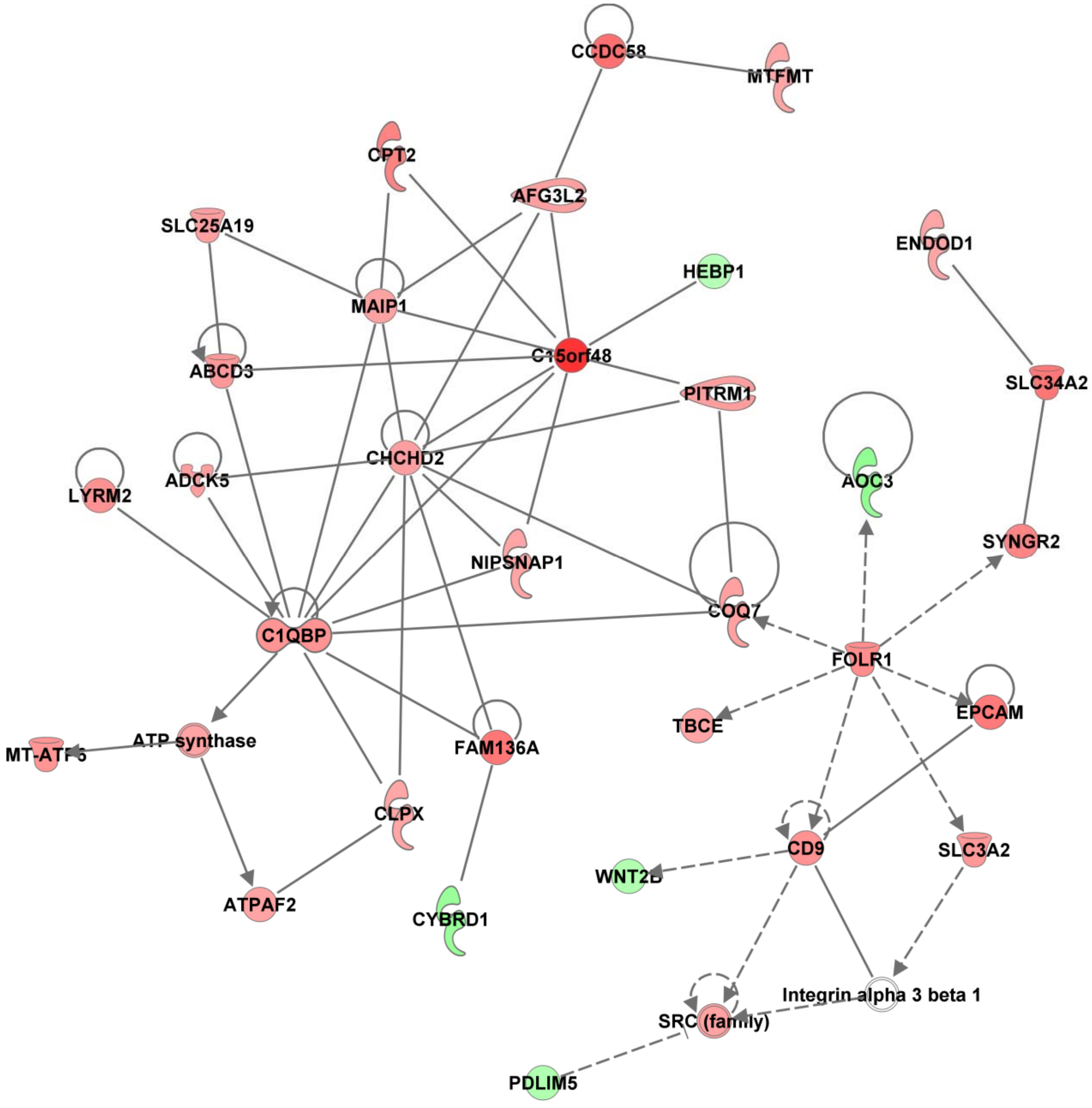

5. **Developmental disorder, hereditary disorder, metabolic disease.**

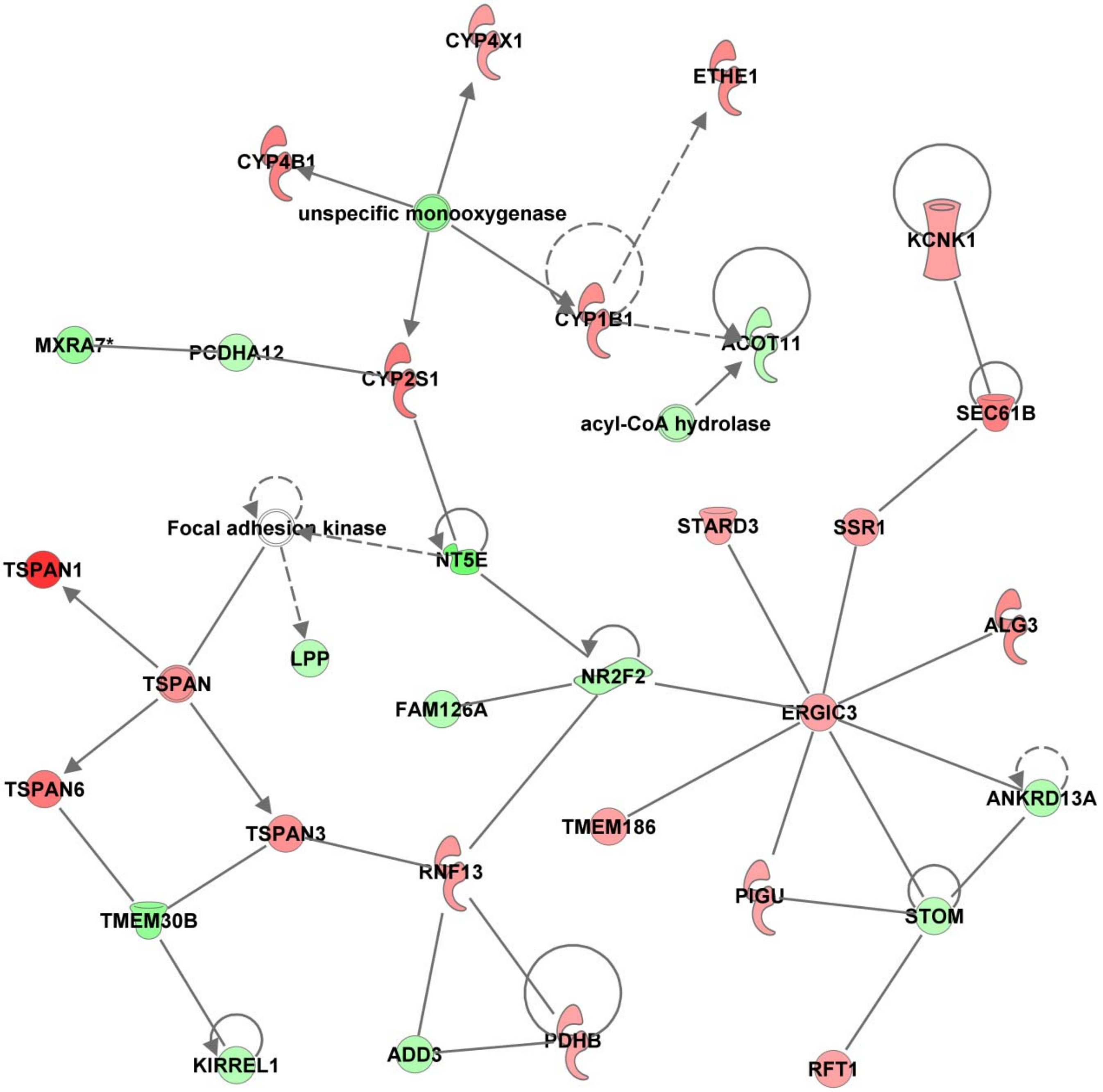

6. Post-translation modification, protein folding, hereditary disorder.

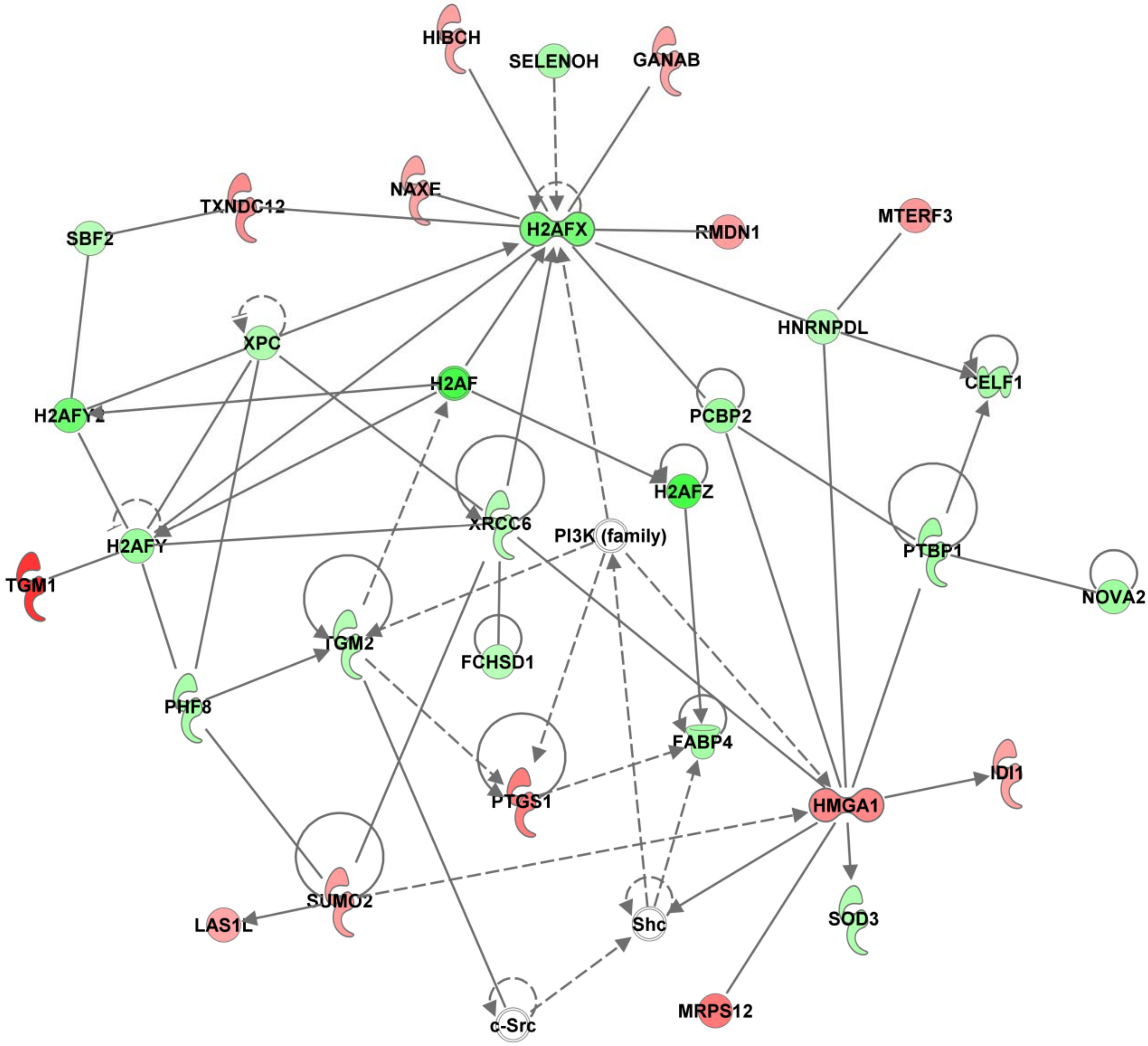

7. Cancer, connective tissue disorders, organismal injury and abnormalities.

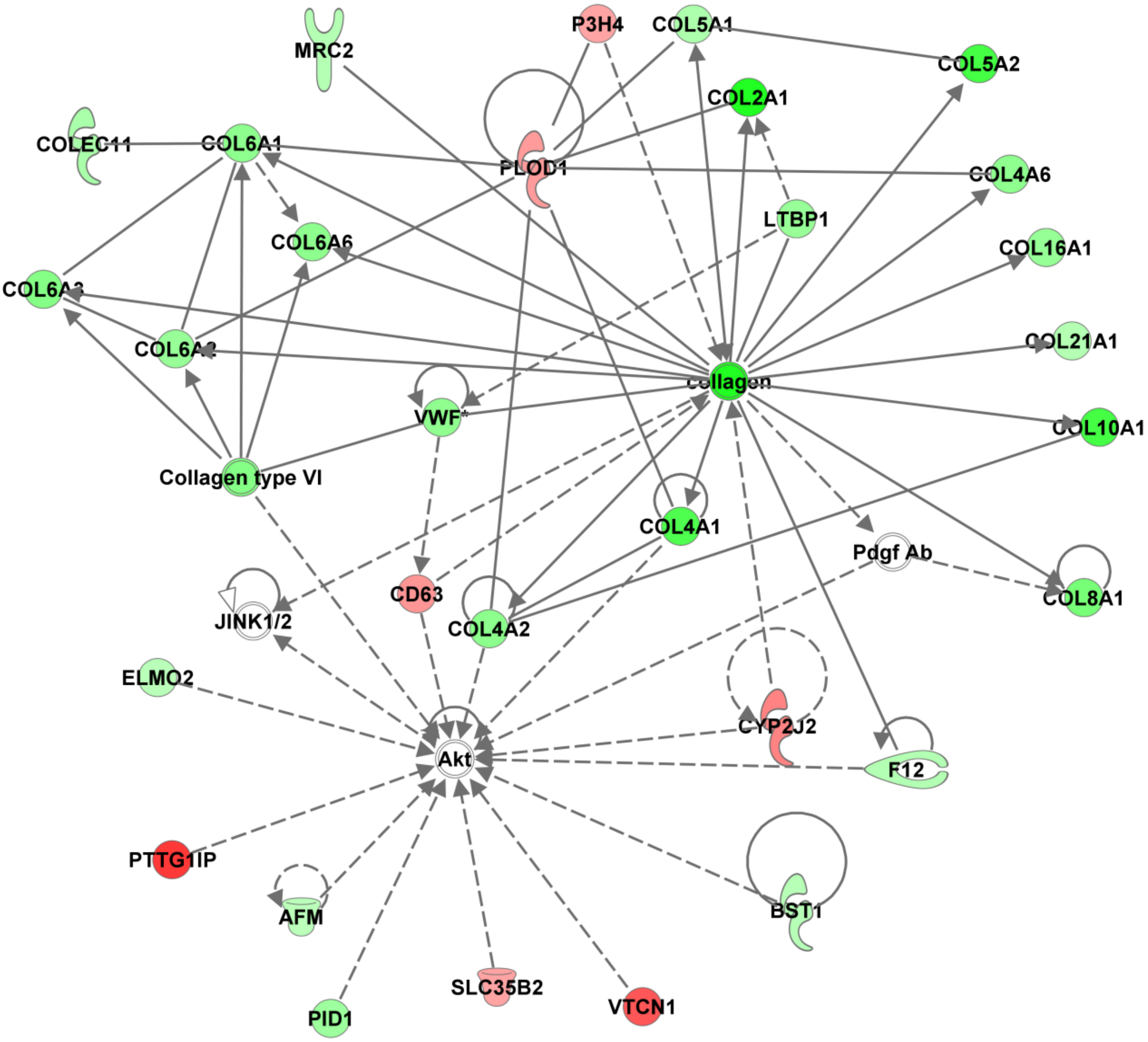

8. **Cellular assembly and organization, cellular function and maintenance, tissue development.**

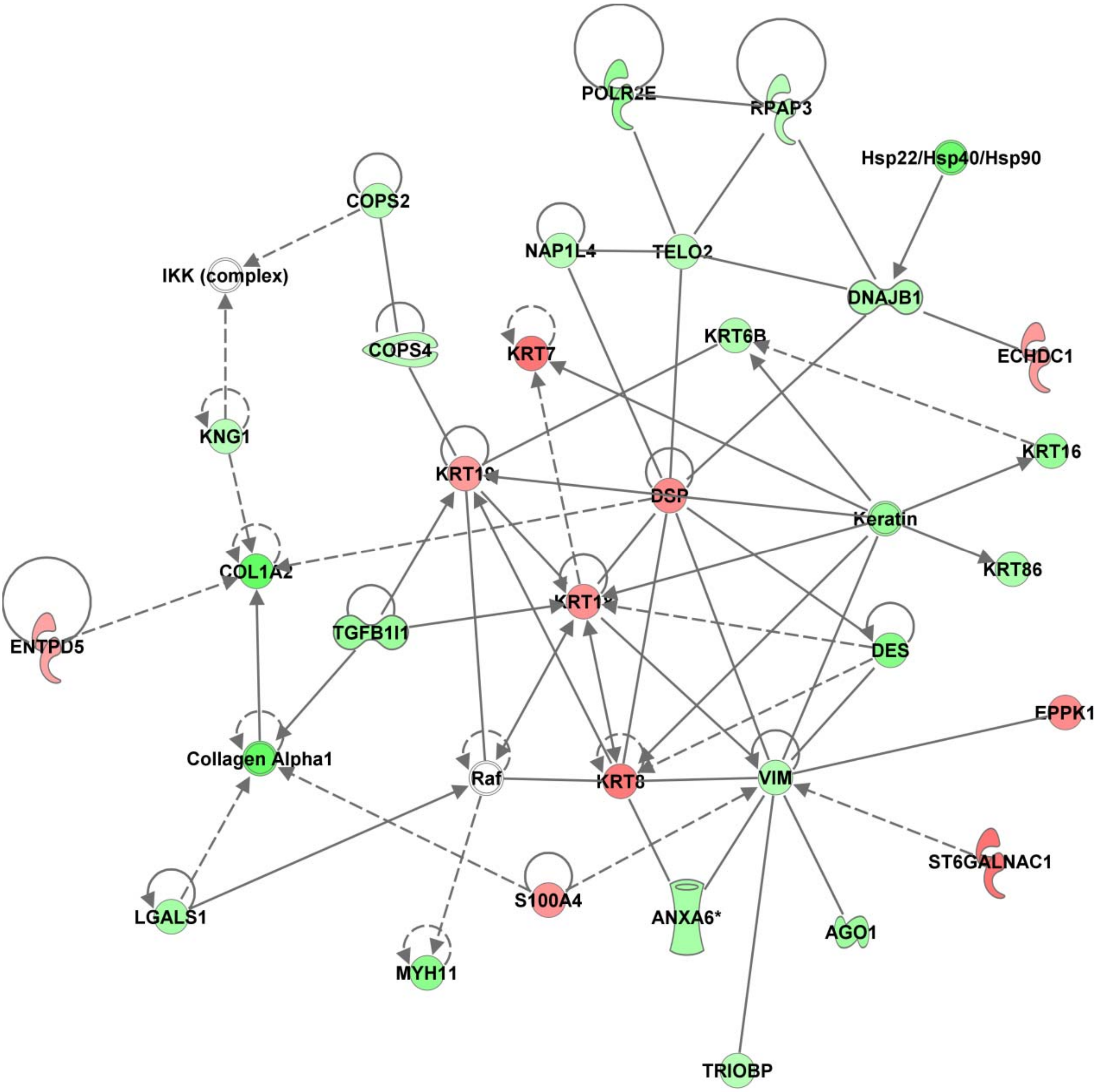

9. **Cellular assembly and organization, cellular function and maintenance, development disorder.**

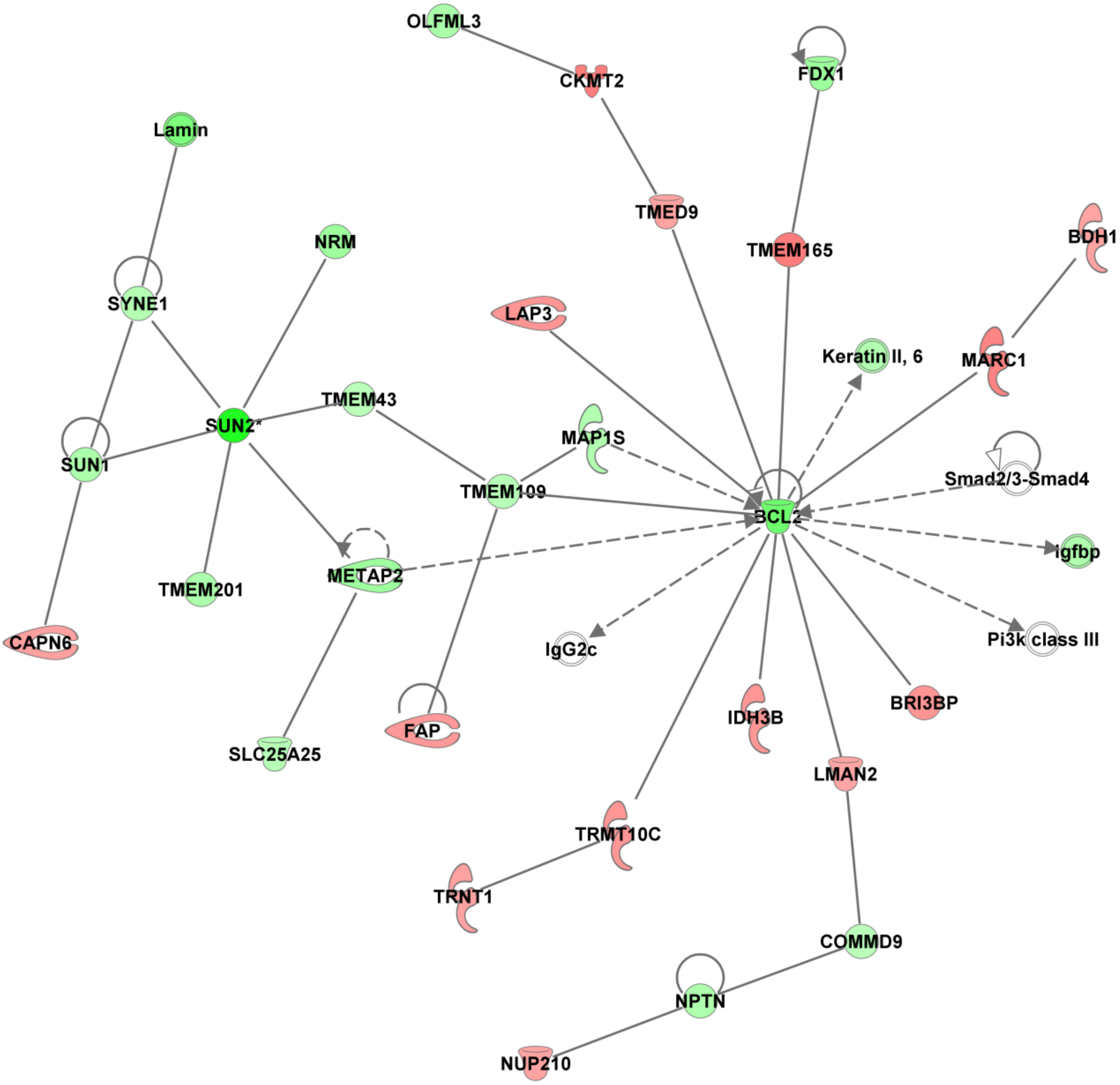

10. Protein synthesis, cell morphology, cellular compromise.

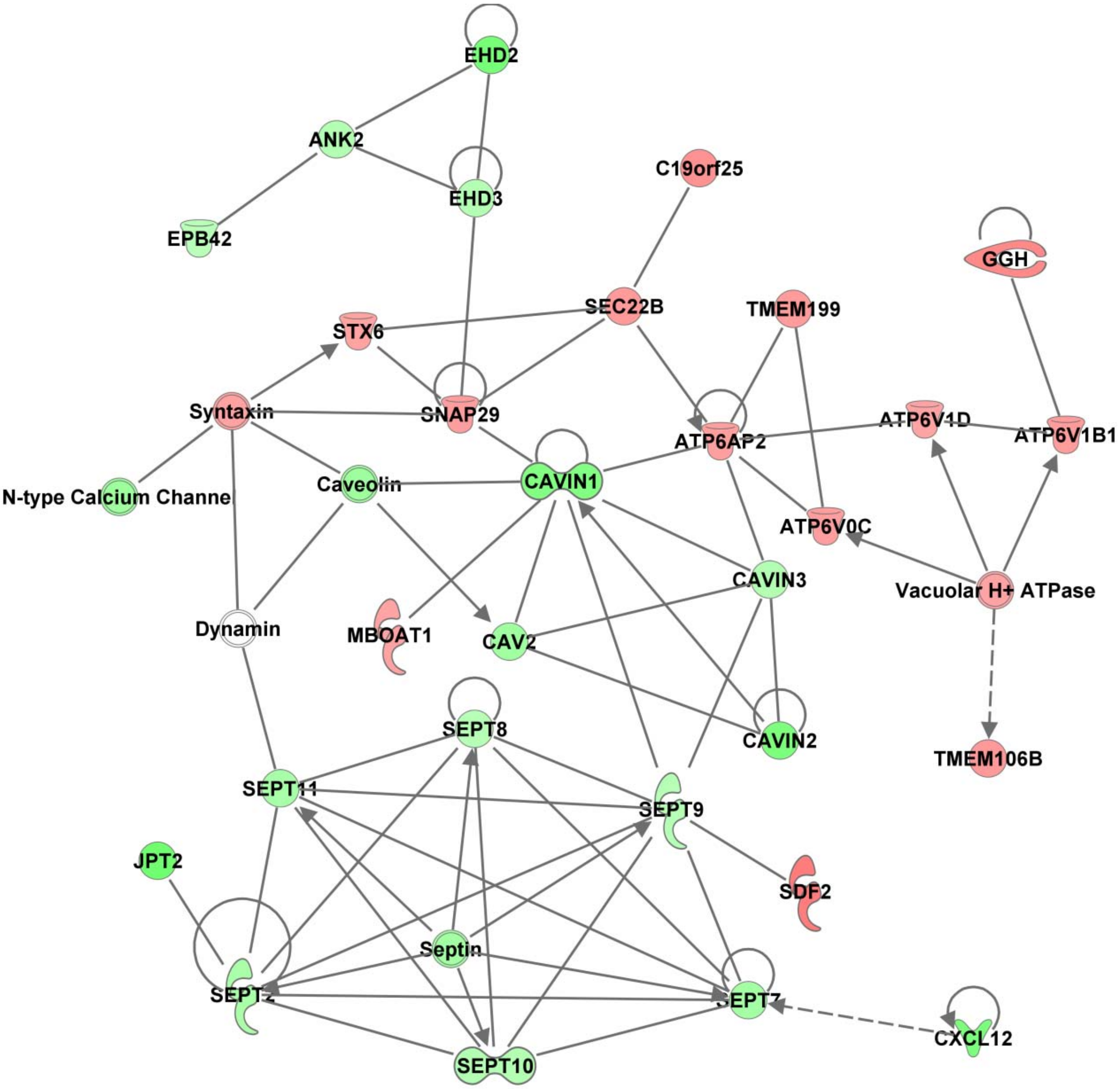

11. Cell death and survival, embryonic development, cell morphology.

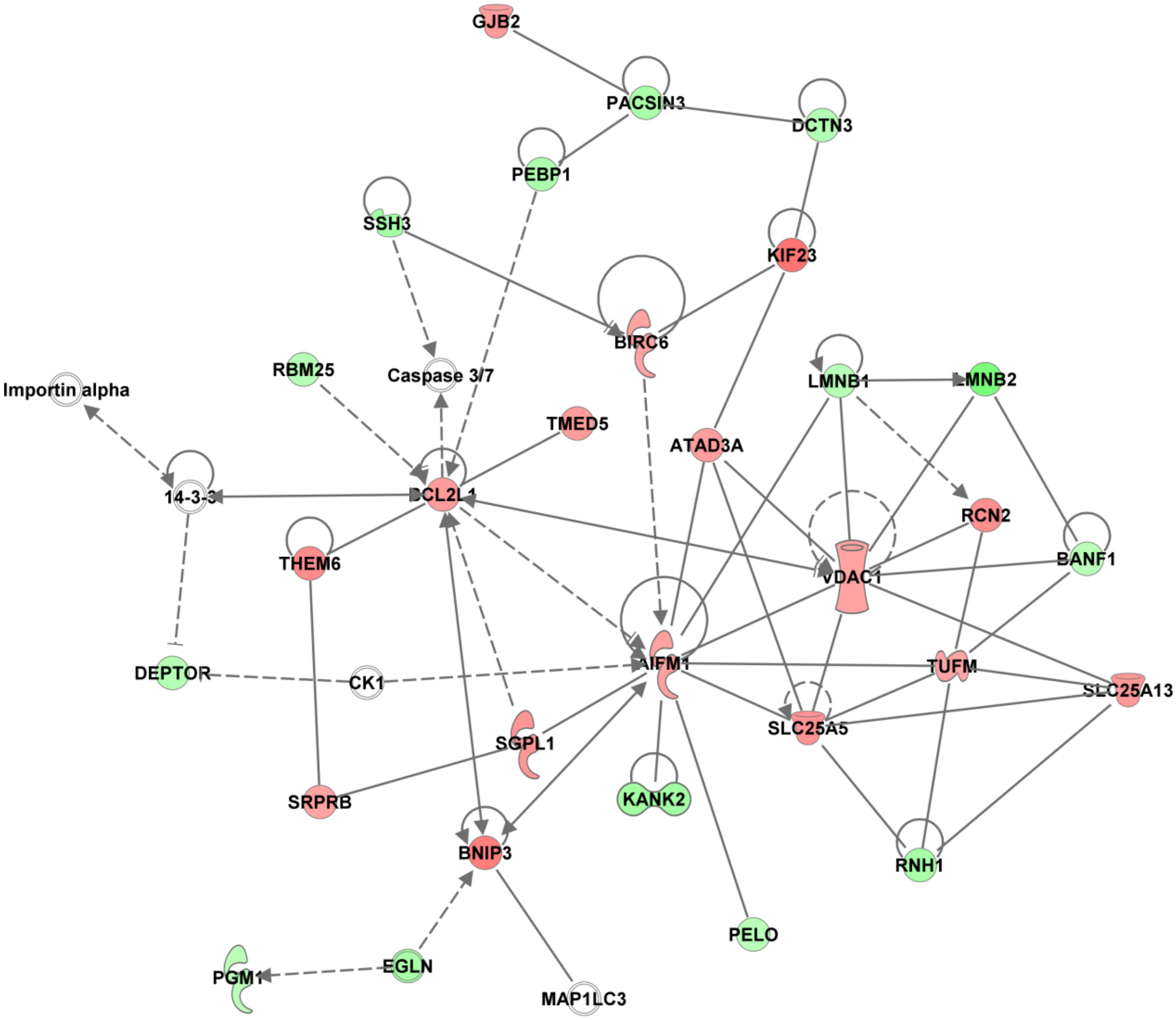

12. Gene expression, protein synthesis, cancer.

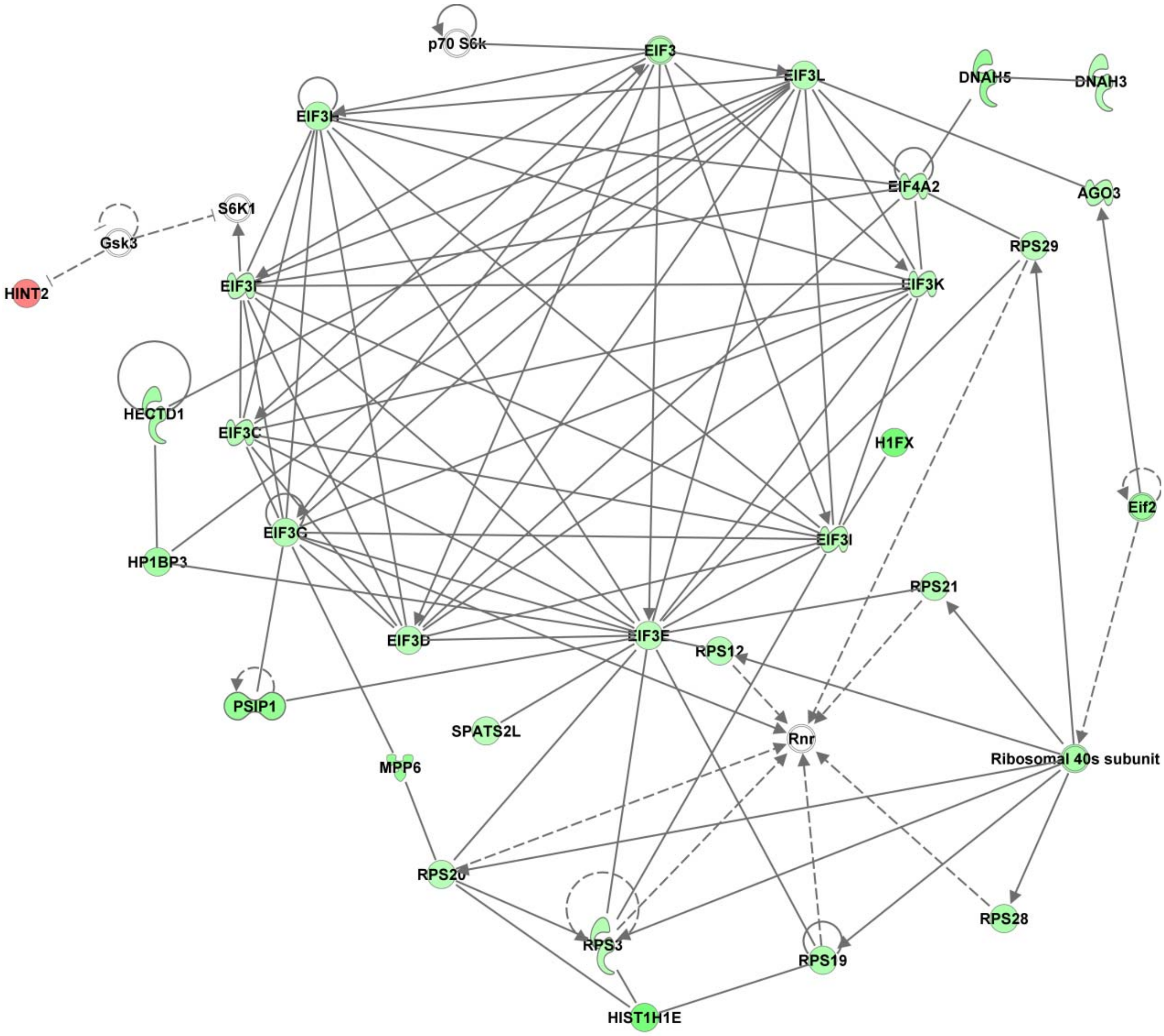

13. **Hair and skin development and function, post-translational modification, protein folding.**

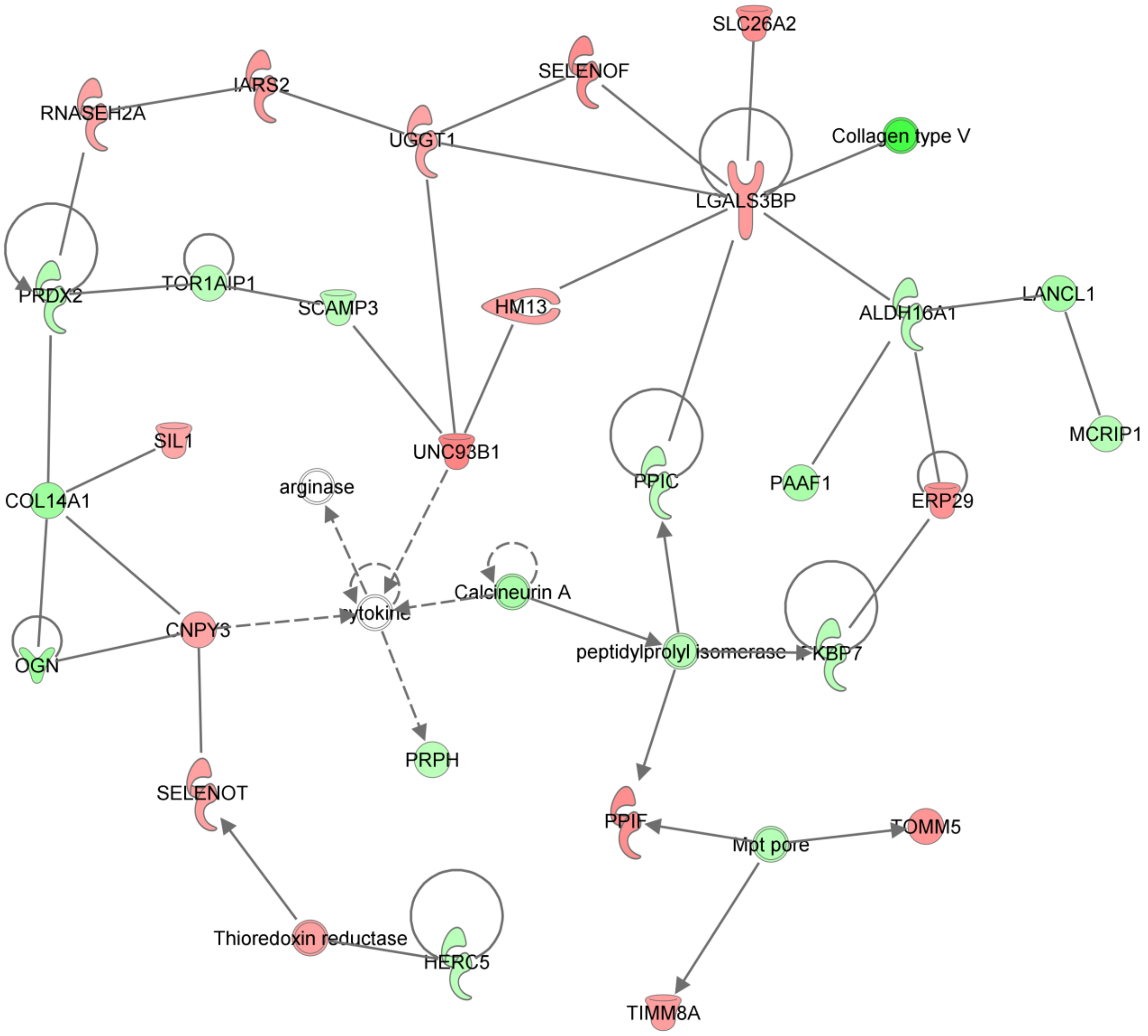

14. Small molecule biochemistry, cancer, cellular development.

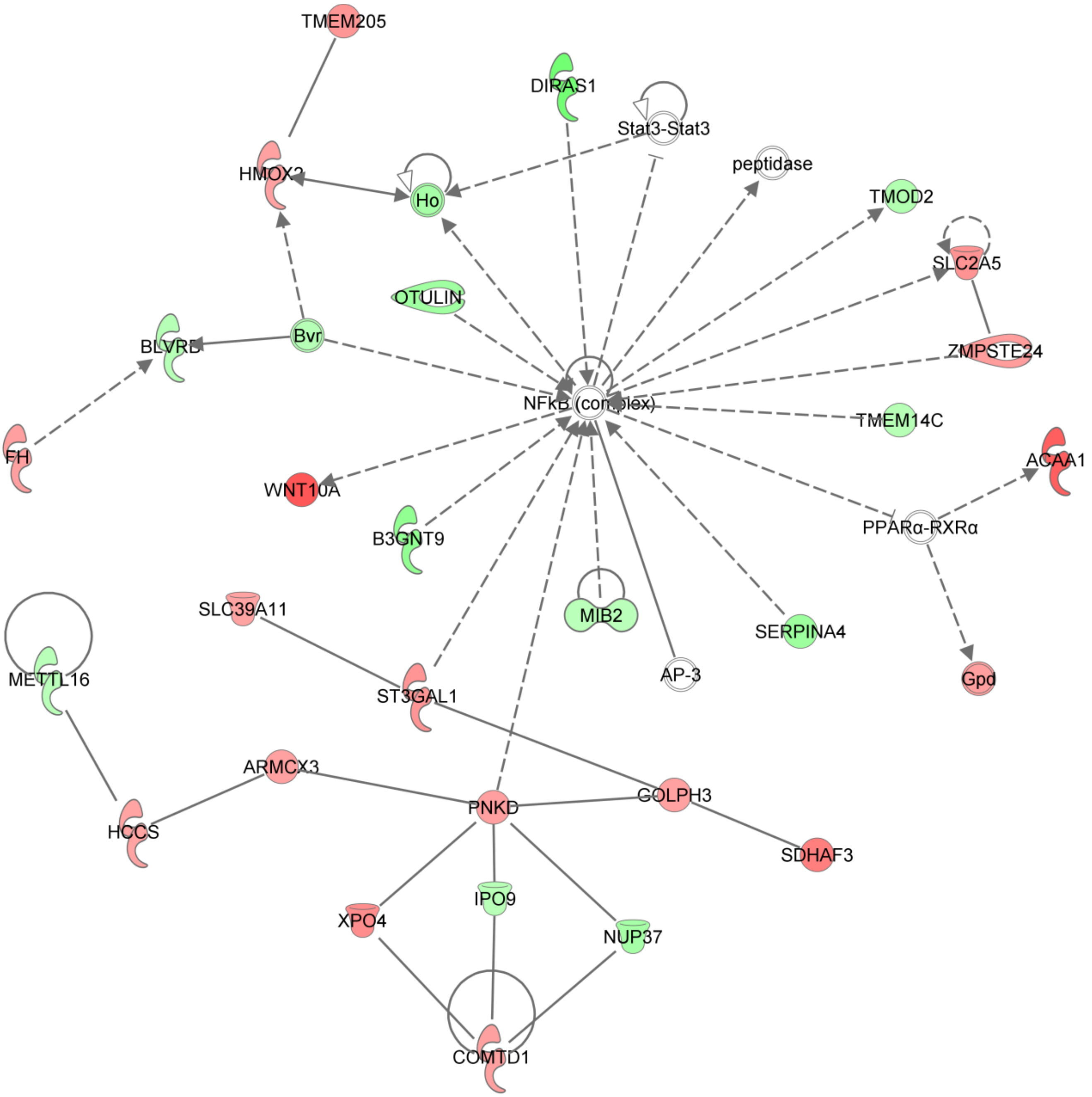

15. Hematological disease, infectious disease, molecular transport.

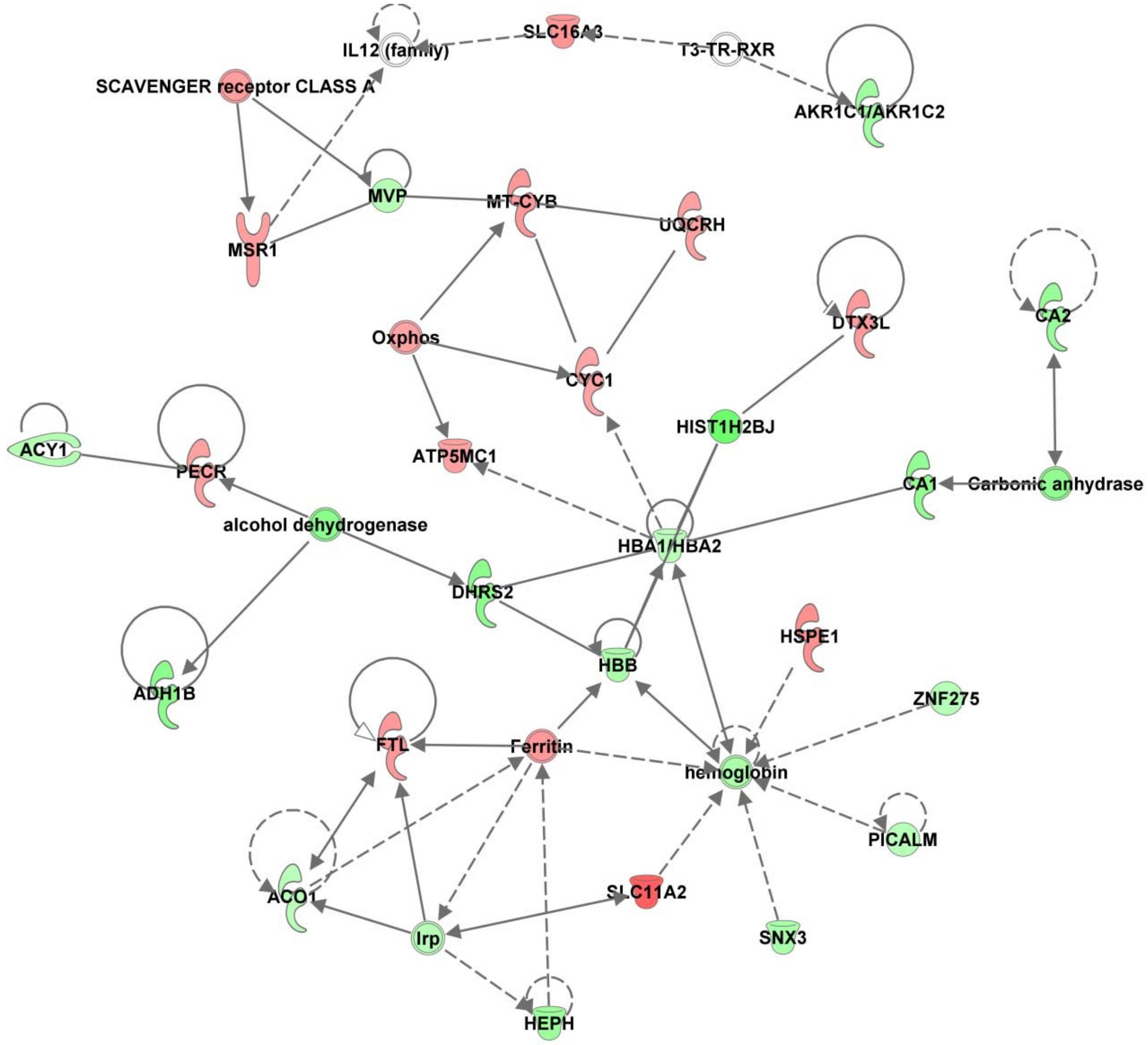

16. Post-translational modification, developmental disorder, hereditary disorder.

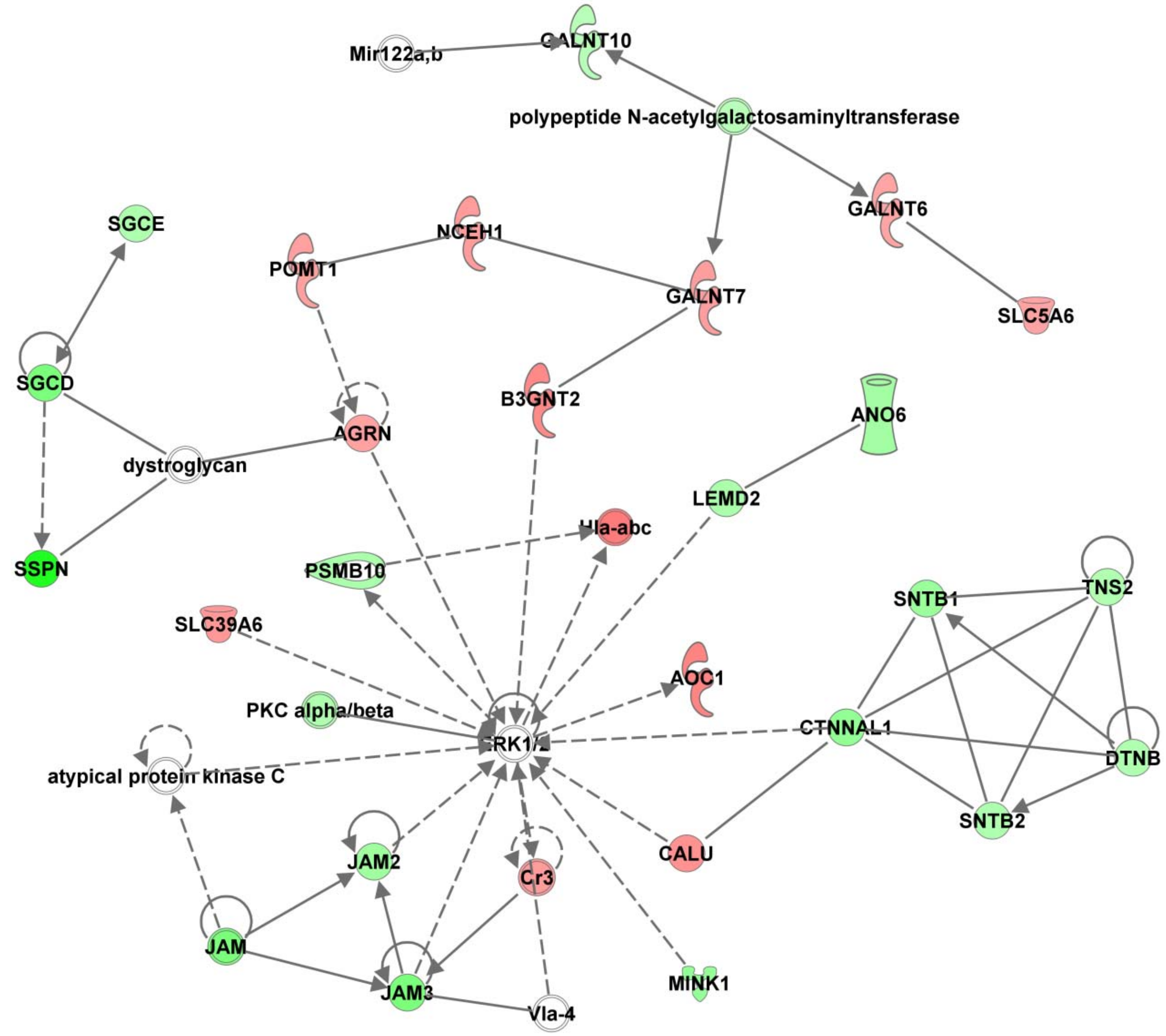

17. Drug metabolism, protein synthesis, small molecule biochemistry.

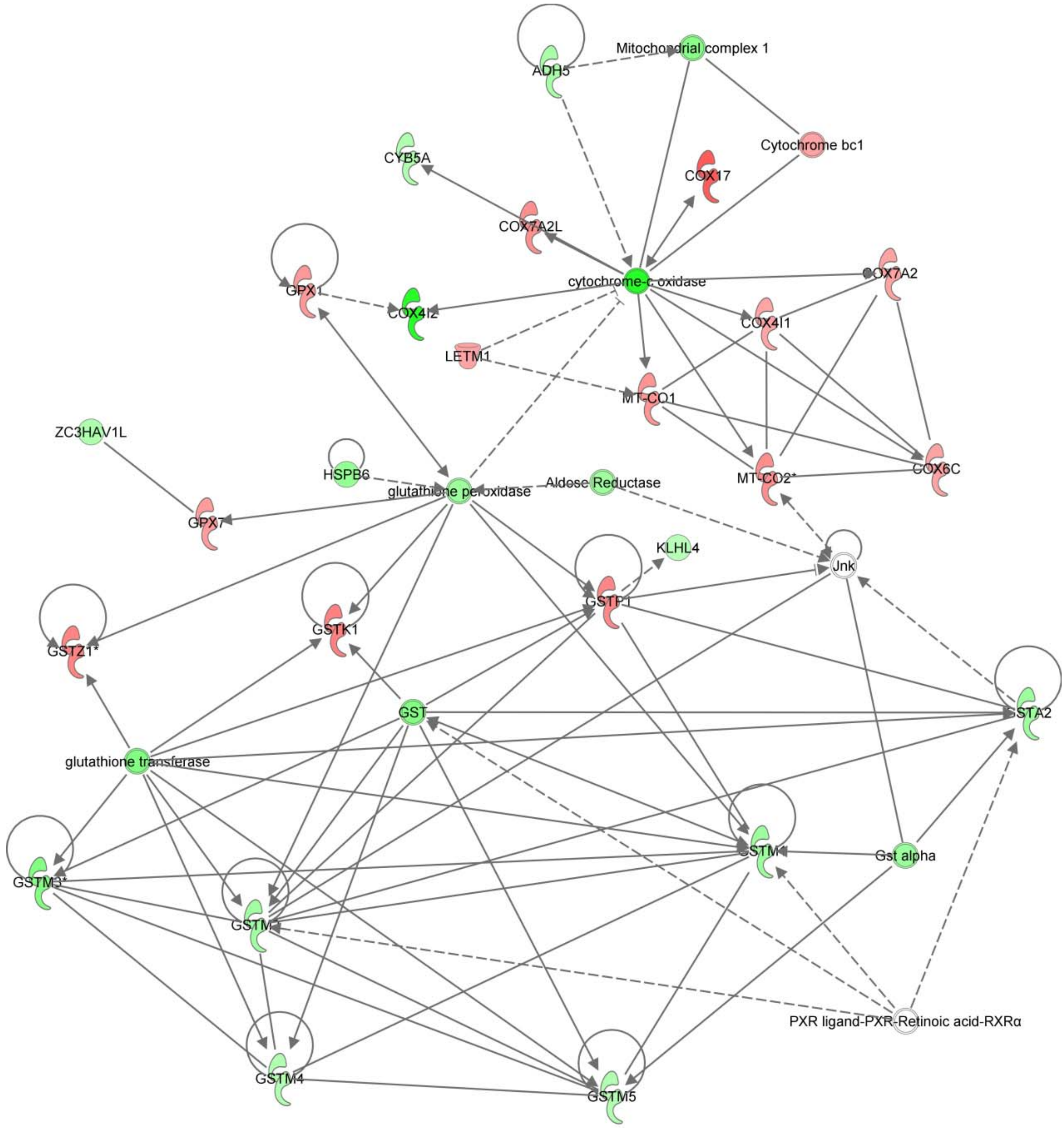

18. Connective tissue disorders, developmental disorder, hereditary disorder.

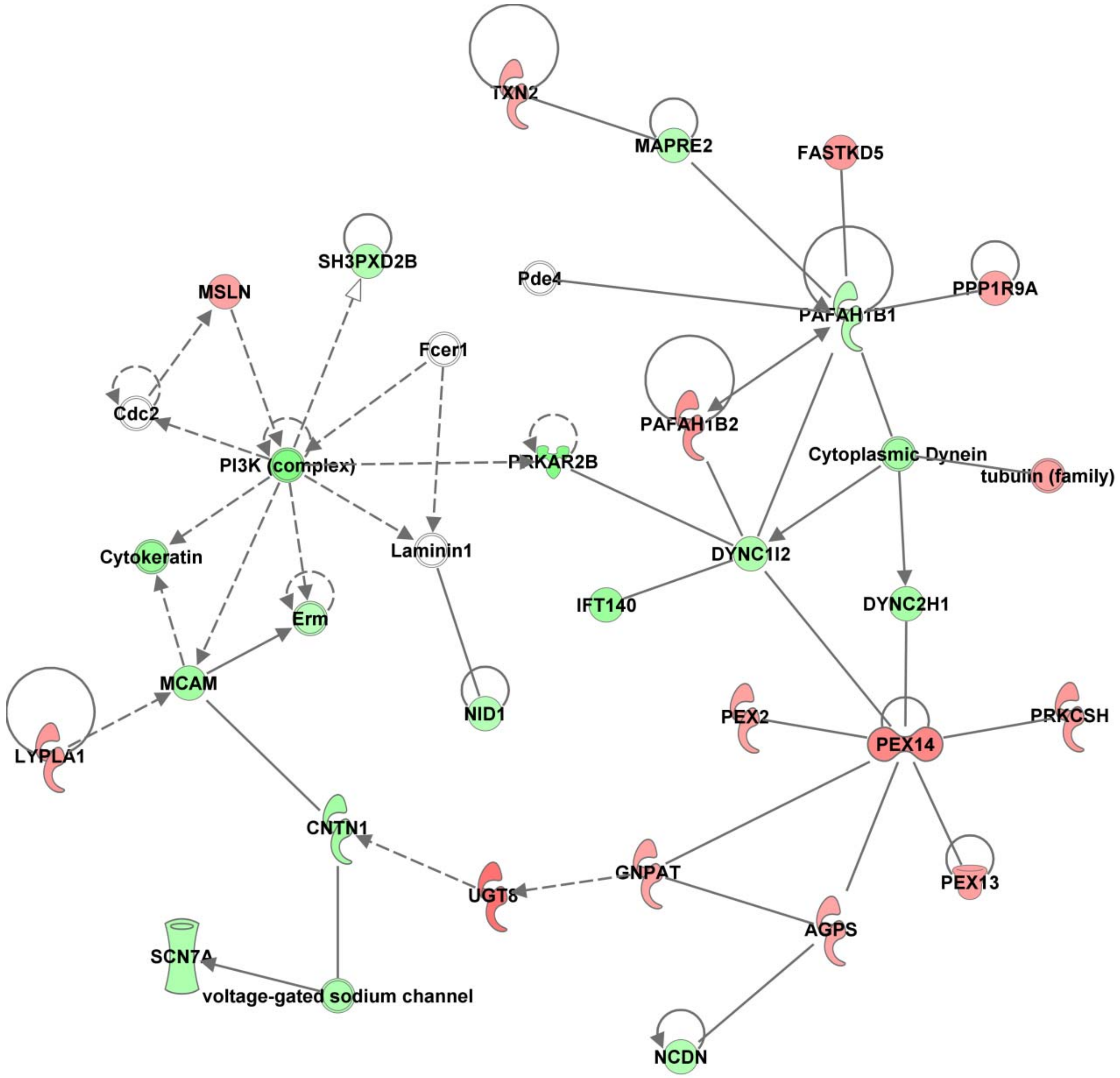

19. Nucleic acid metabolism, small molecule biochemistry, cancer.

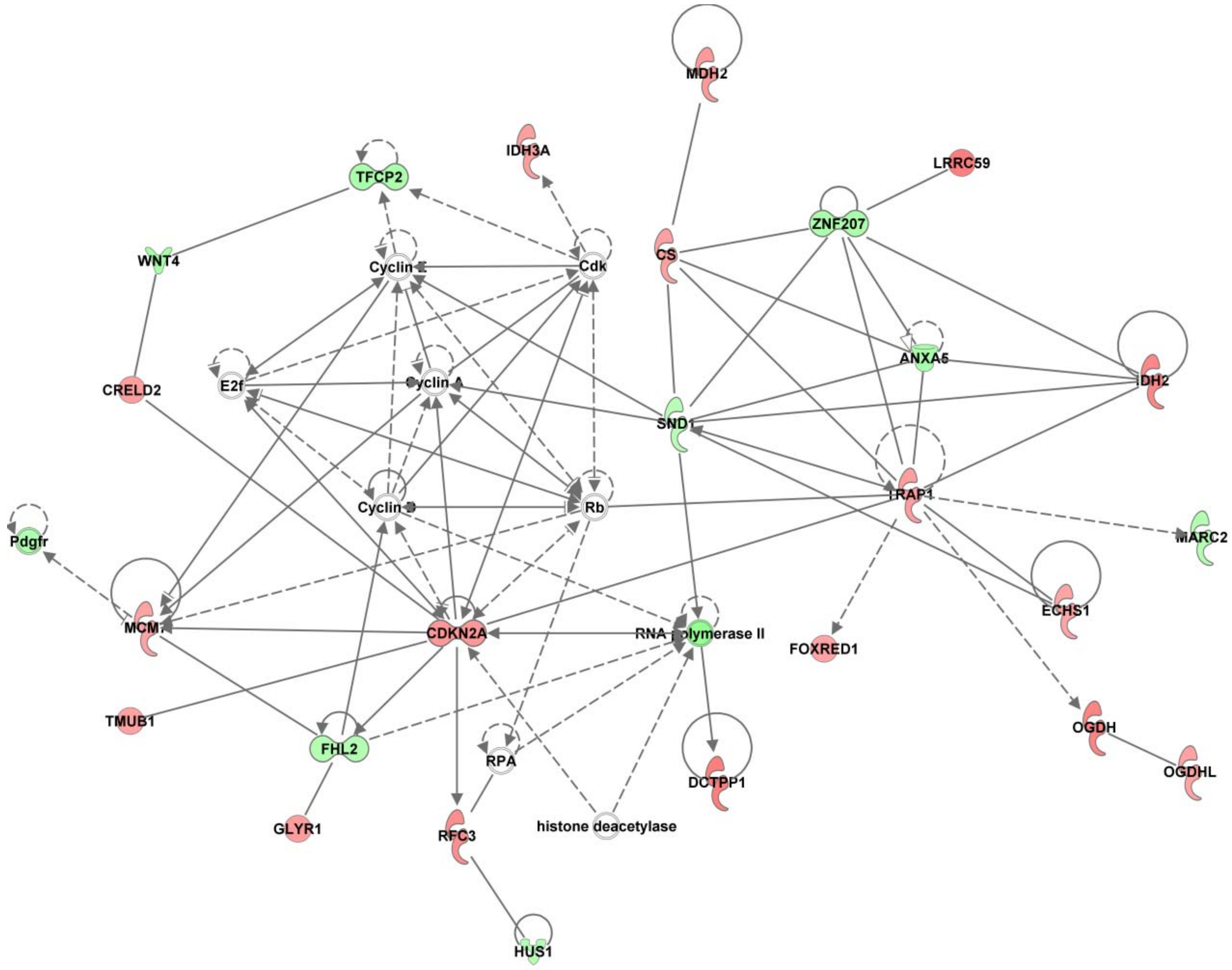

20. **Tissue development, connective tissue disorders, dermatological diseases and conditions.**

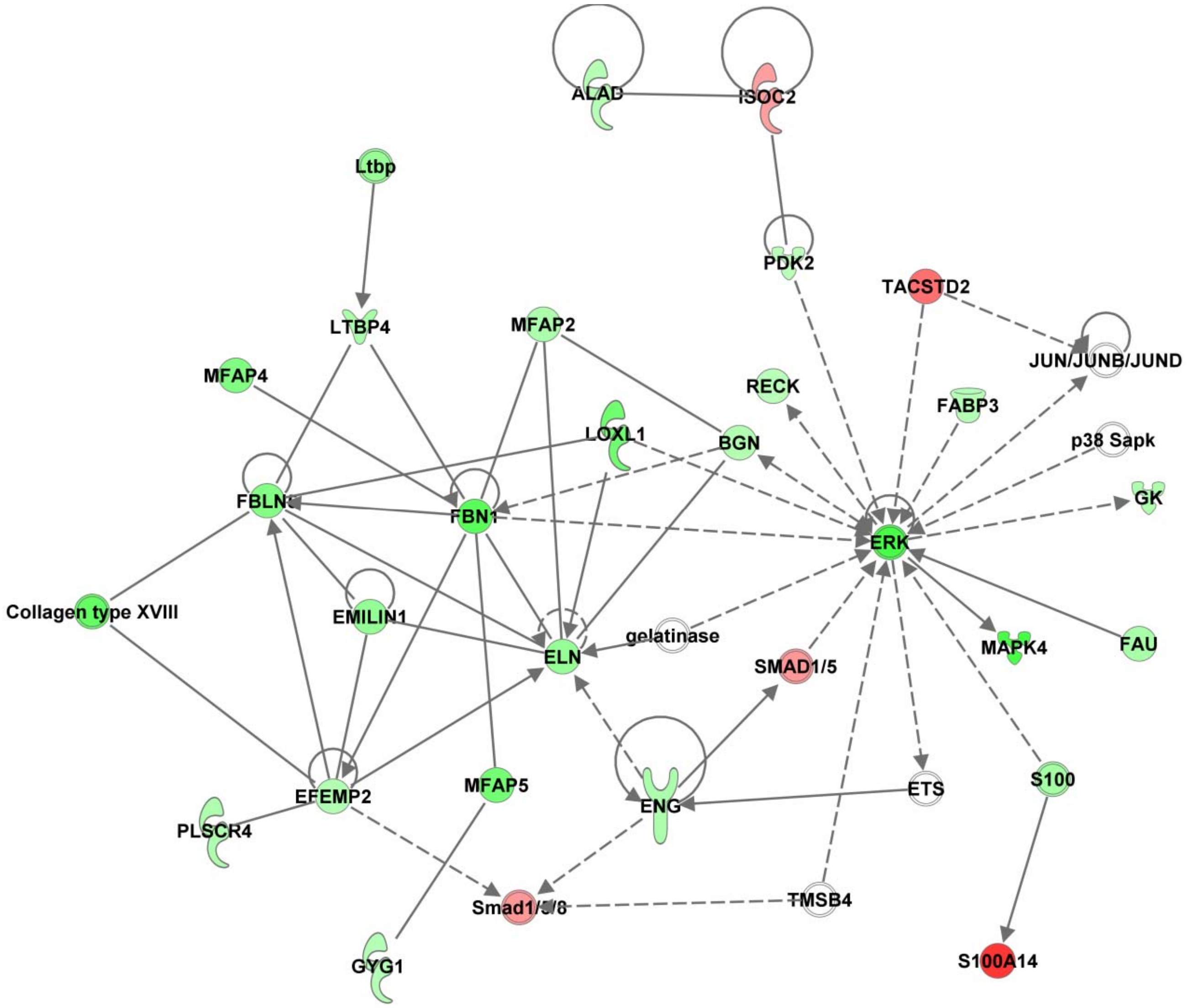

21. **Nucleic acid metabolism, small molecule, biochemistry, DNA replication, recombination, and repair.**

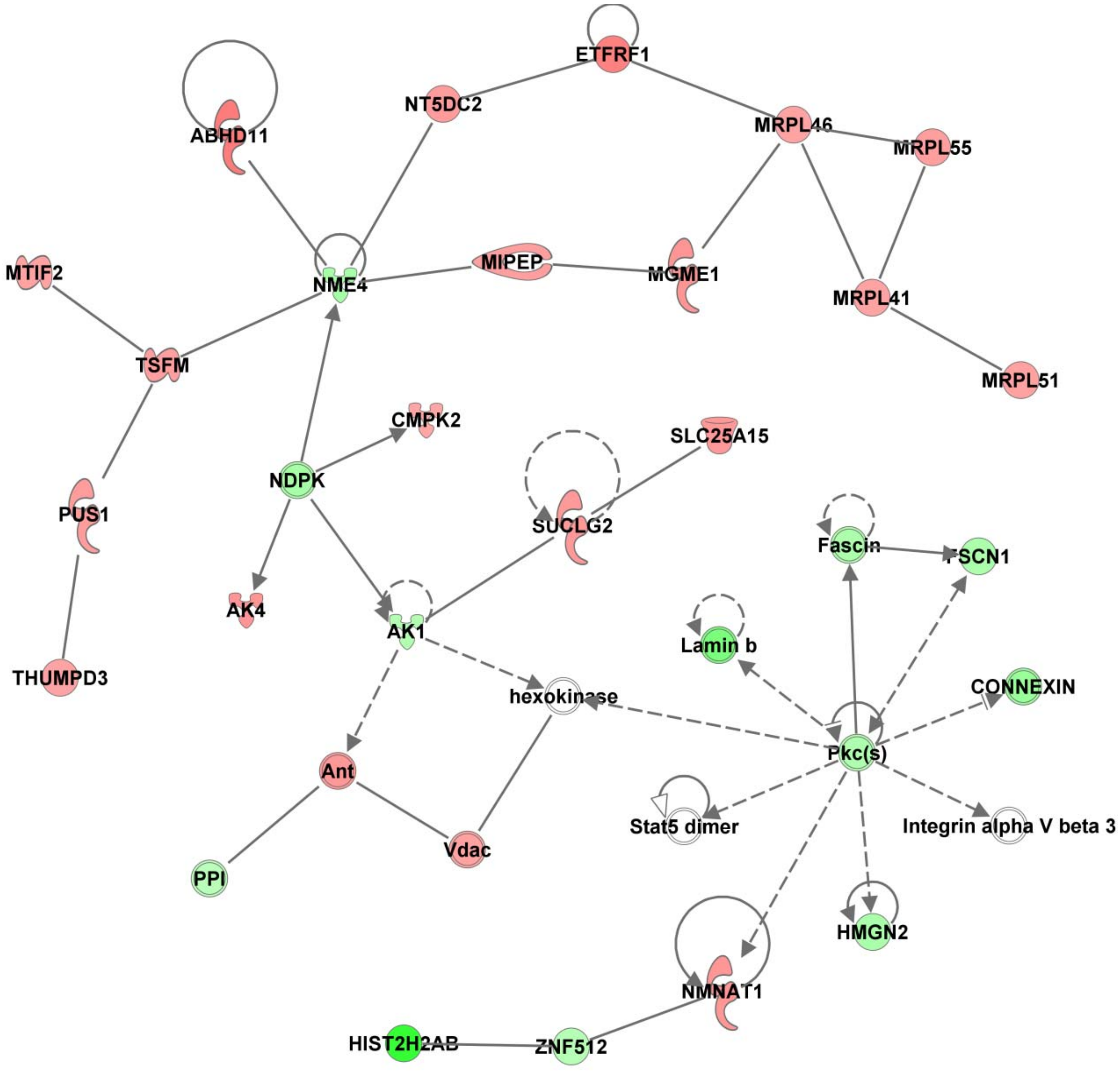

22. Neurological disease, organismal injury and abnormalities, cell death and survival.

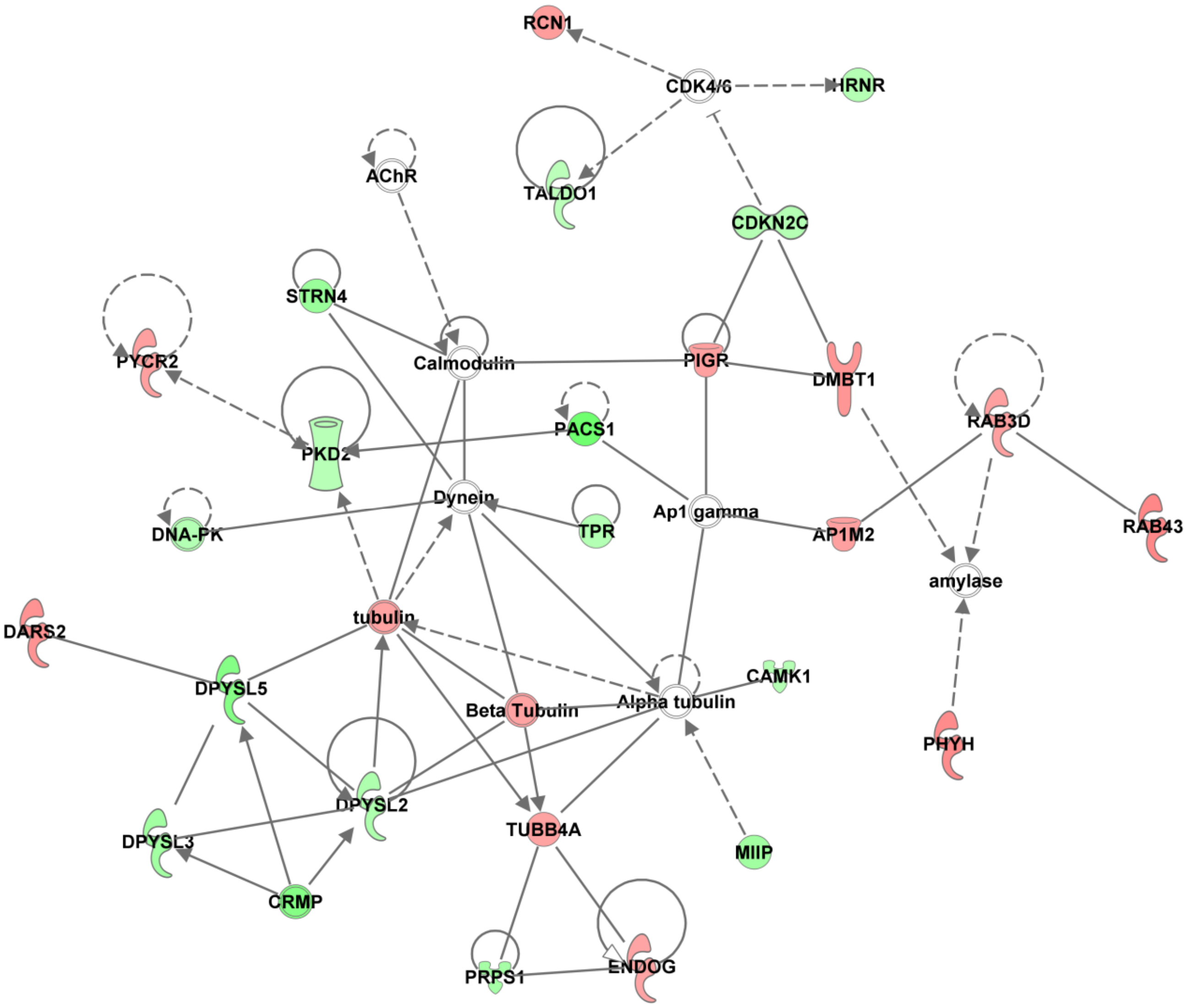

23. **Cardiovascular disease, organismal injury and abnormalities, hematological disease.**

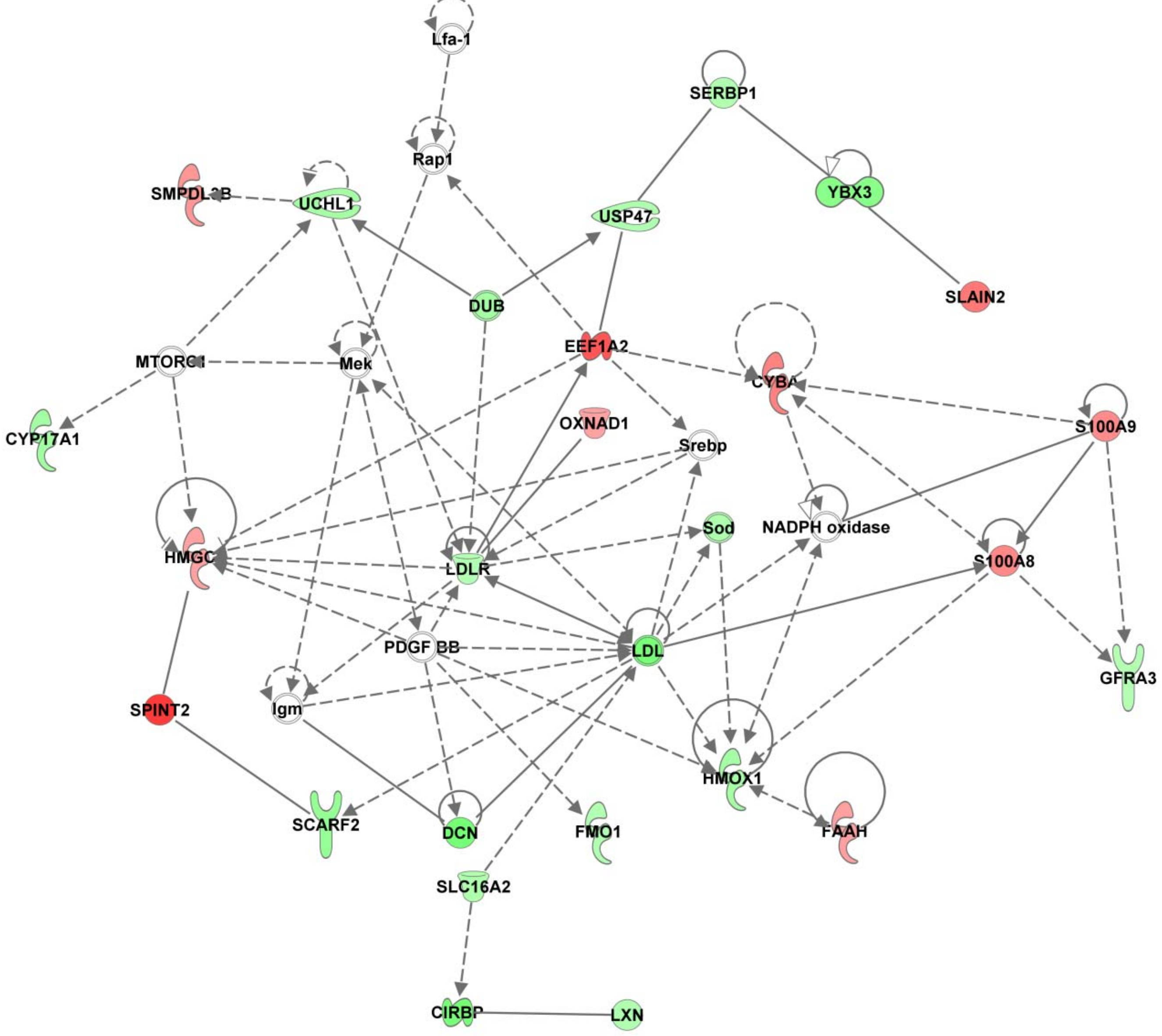

24. **Cellular development, cellular growth and proliferation, embryonic development.**

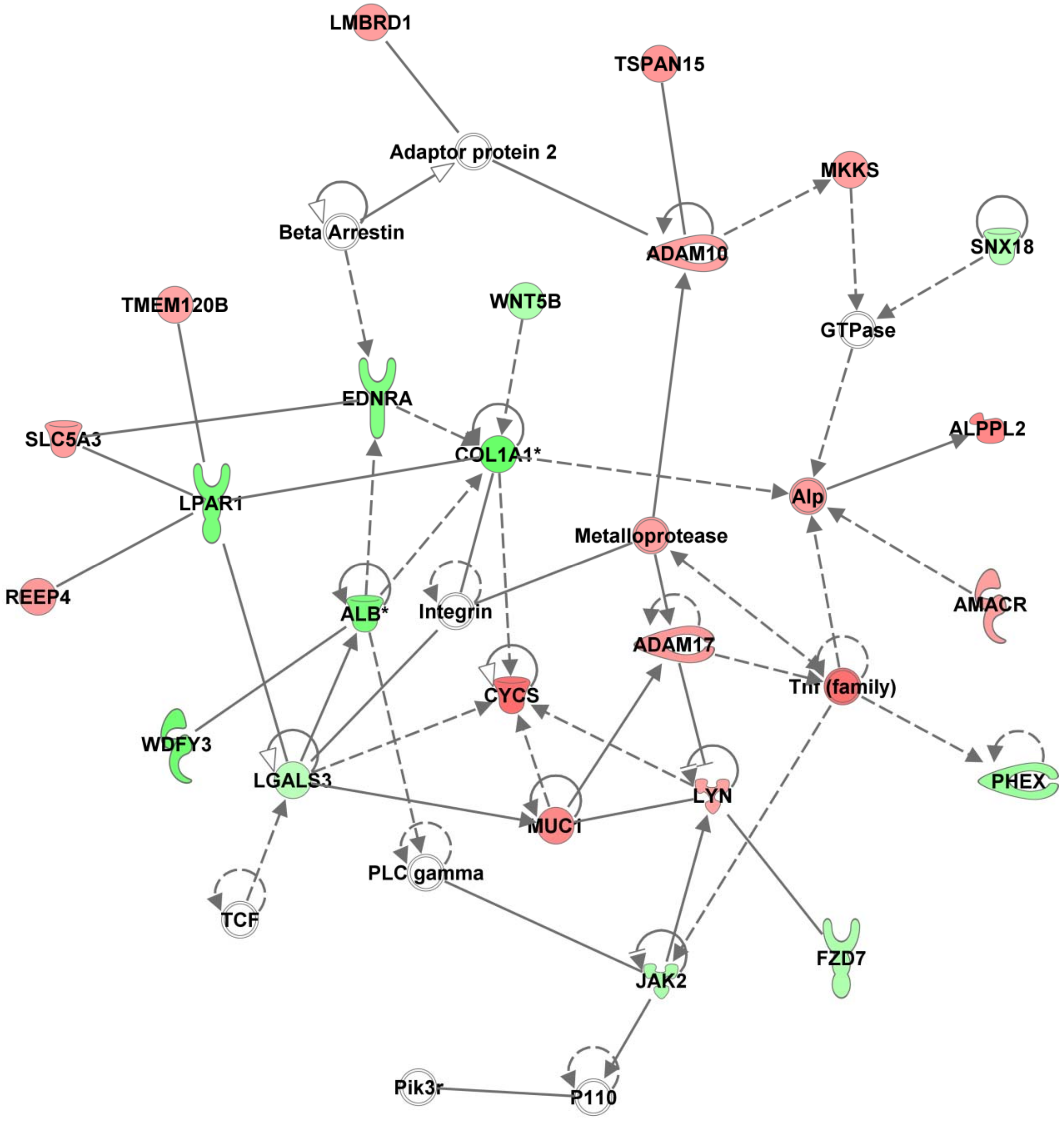

25. Development disorder, hereditary disorder, neurological disease.

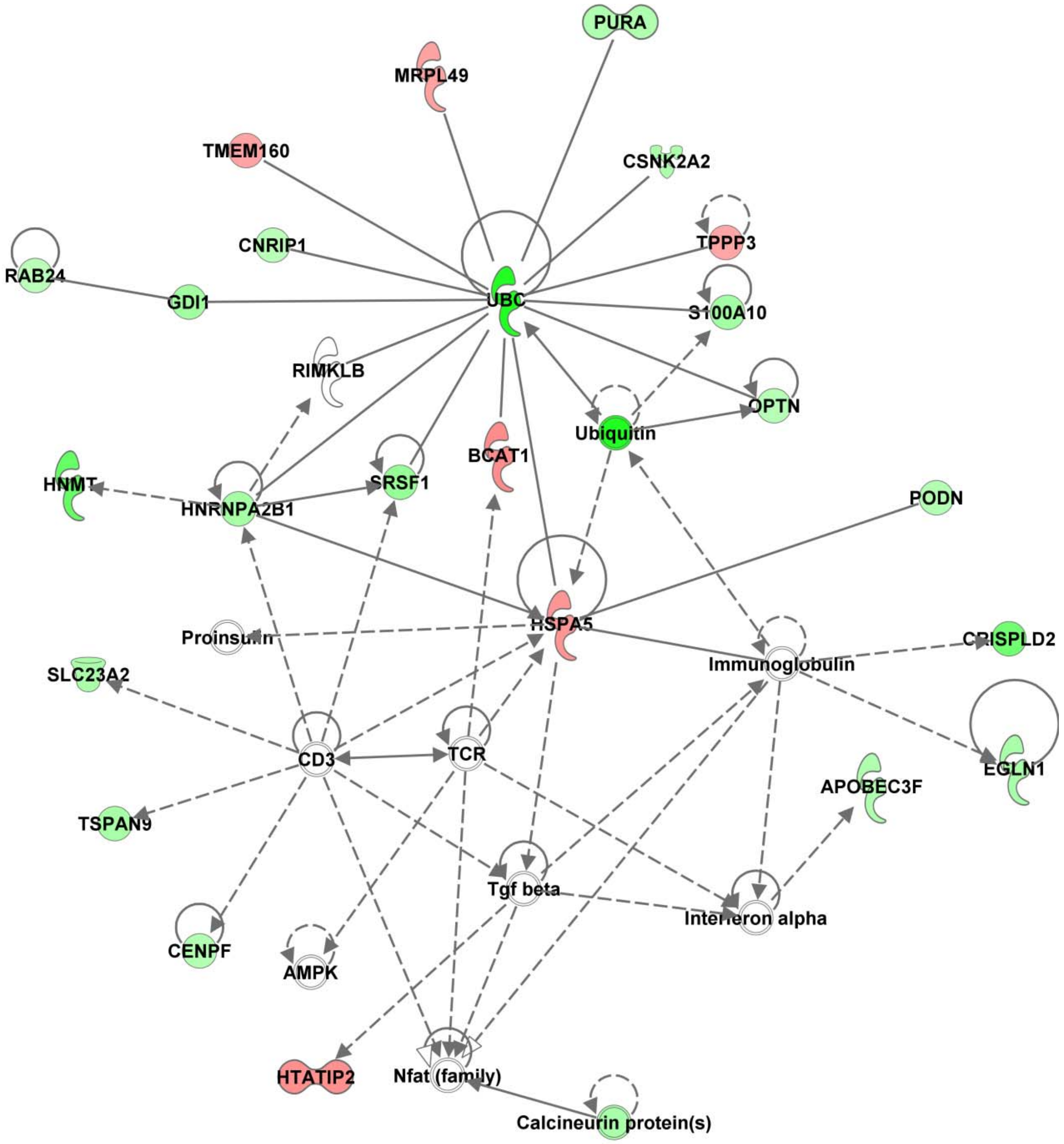

Supplement: Supplementary file 3 — (PDF 3766 kb) [file 13167_2019_170_MOESM3_ESM.pdf]
