# Supplementary material for: Signaling pathway network alterations in human ovarian cancers identified with quantitative mitochondrial proteomics
Source: EPMA J. 2019 Jun 8;10(2):153–72. doi: 10.1007/s13167-019-00170-5 (PMC6562010; doi:10.1007/s13167-019-00170-5)
Supplement: Supplementary file 4 — (PDF 2864 kb) [file 13167_2019_170_MOESM4_ESM.pdf]

Supplementary figure 4

The KMplot analysis revealed that 62 of 102 hub molecules resulted in significant ovarian cancer overall survival (50 none reported molecules).

1.ADH5

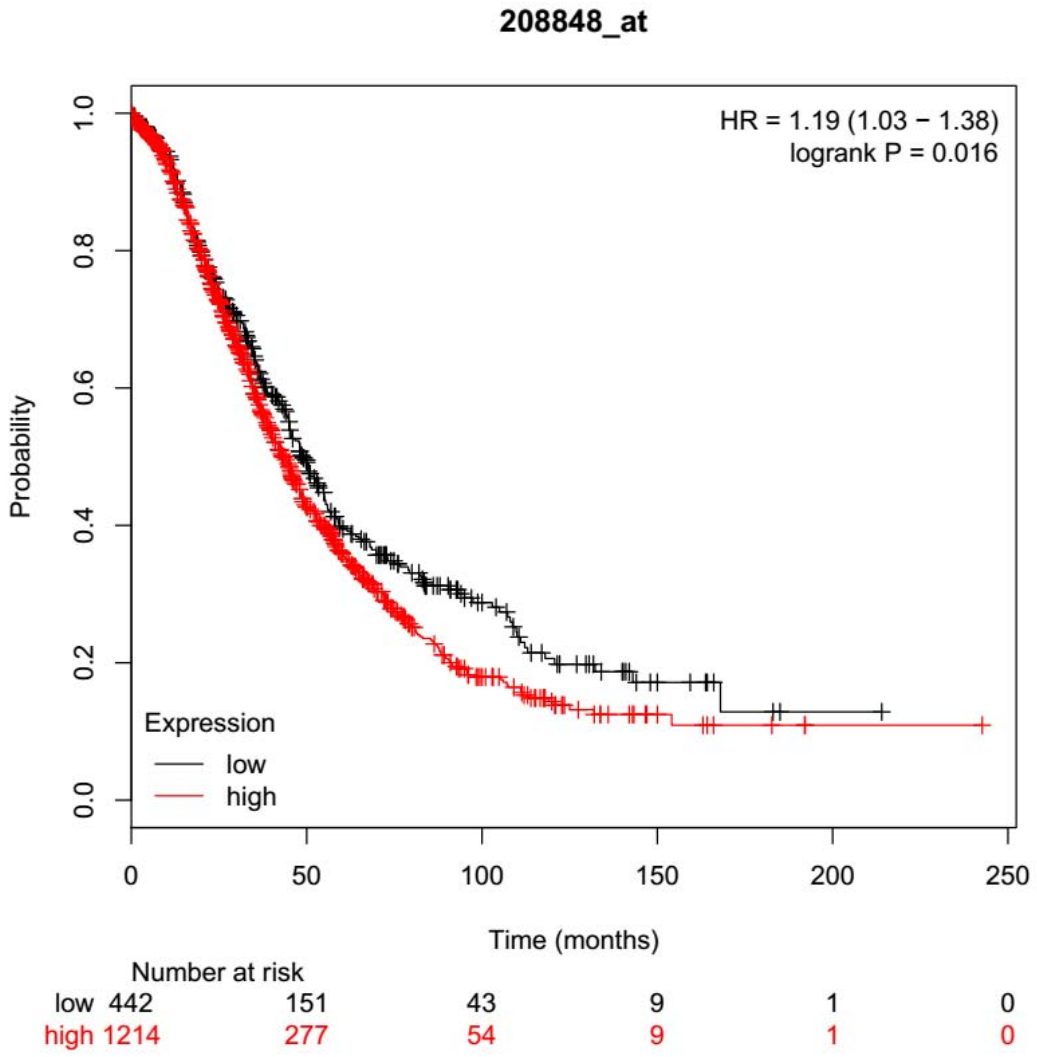

2. ADRA2A

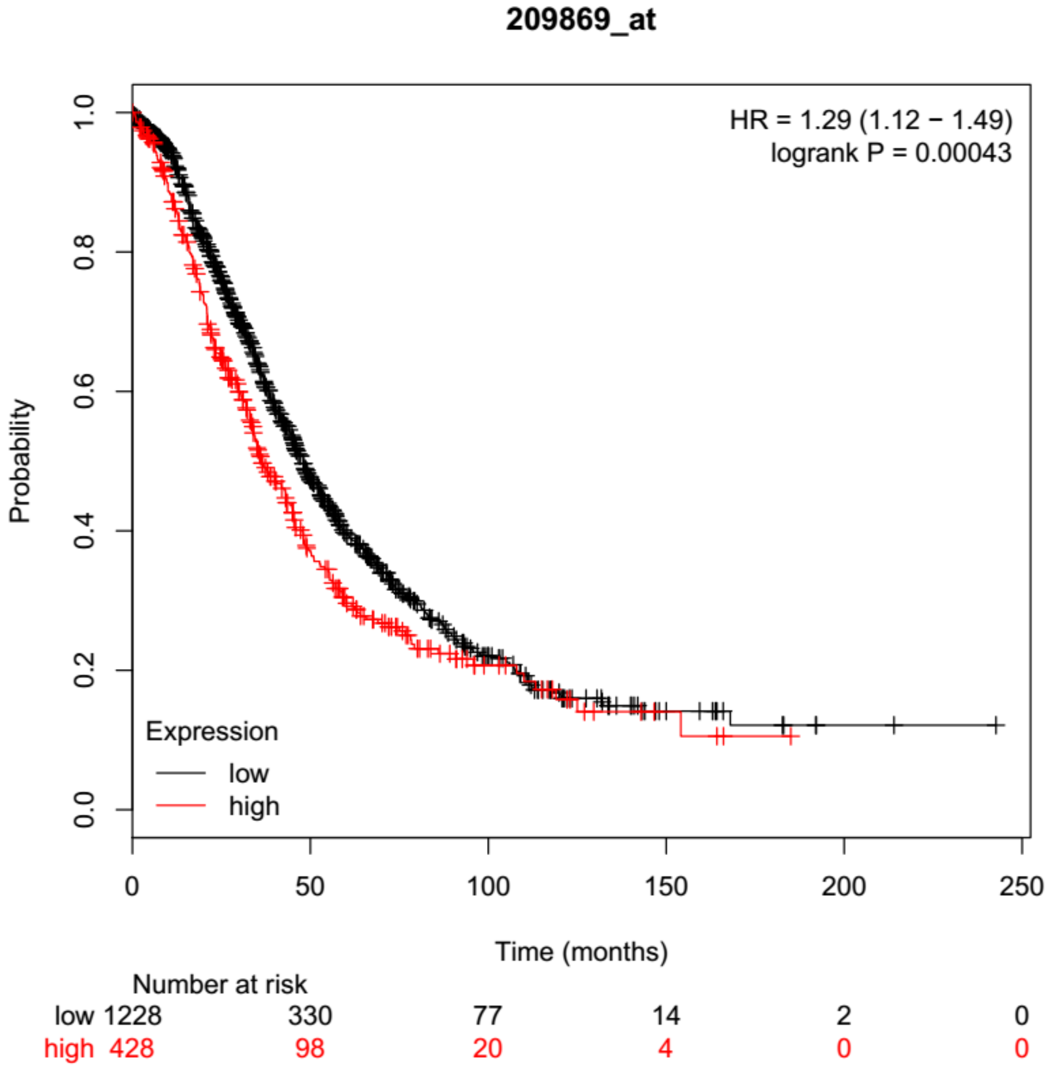
209869_at
HR = 1.29 (1.12 − 1.49)
logrank P = 0.00043
Probability
0.0
0.2
0.4
0.6
0.8
1.0
Expression
low
high
0
50
100
150
200
250
Time (months)
Number at risk
low 1228 330 77 14 2 0
high 428 98 20 4 0 0

3. ASPN

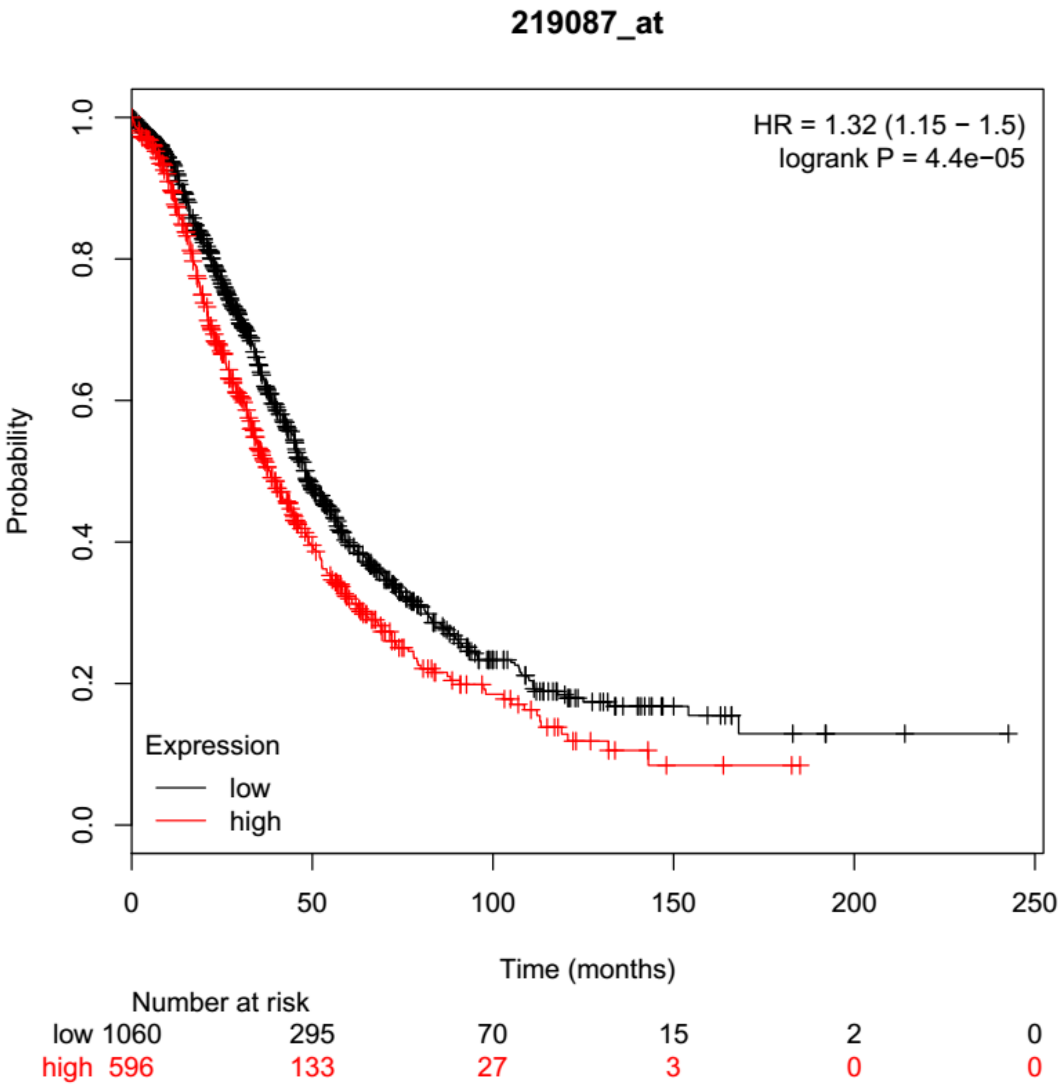

219087_at
HR = 1.32 (1.15 − 1.5)
logrank P = 4.4e−05
Probability
0.0
0.2
0.4
0.6
0.8
1.0
Expression
low
high
0
50
100
150
200
250
Time (months)
Number at risk
low 1060
295
70
15
2
0
high 596
133
27
3
0
0

4. BDH2

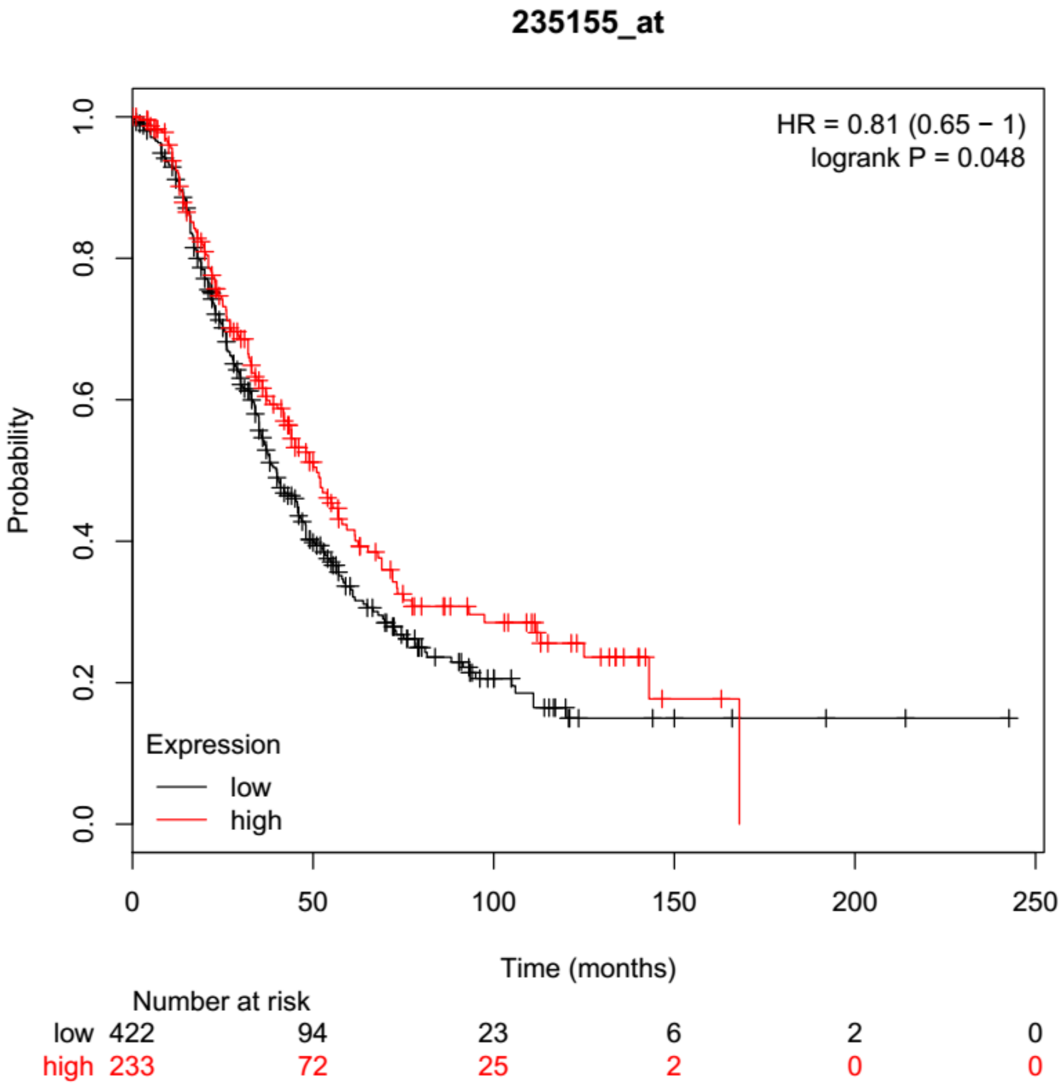

235155_at
HR = 0.81 (0.65 − 1)
logrank P = 0.048
Probability
1.0
0.8
0.6
0.4
0.2
0.0
Expression
low
high
0
50
100
150
200
250
Time (months)
Number at risk
low 422 94 23 6 2 0
high 233 72 25 2 0 0

5. COL3A1

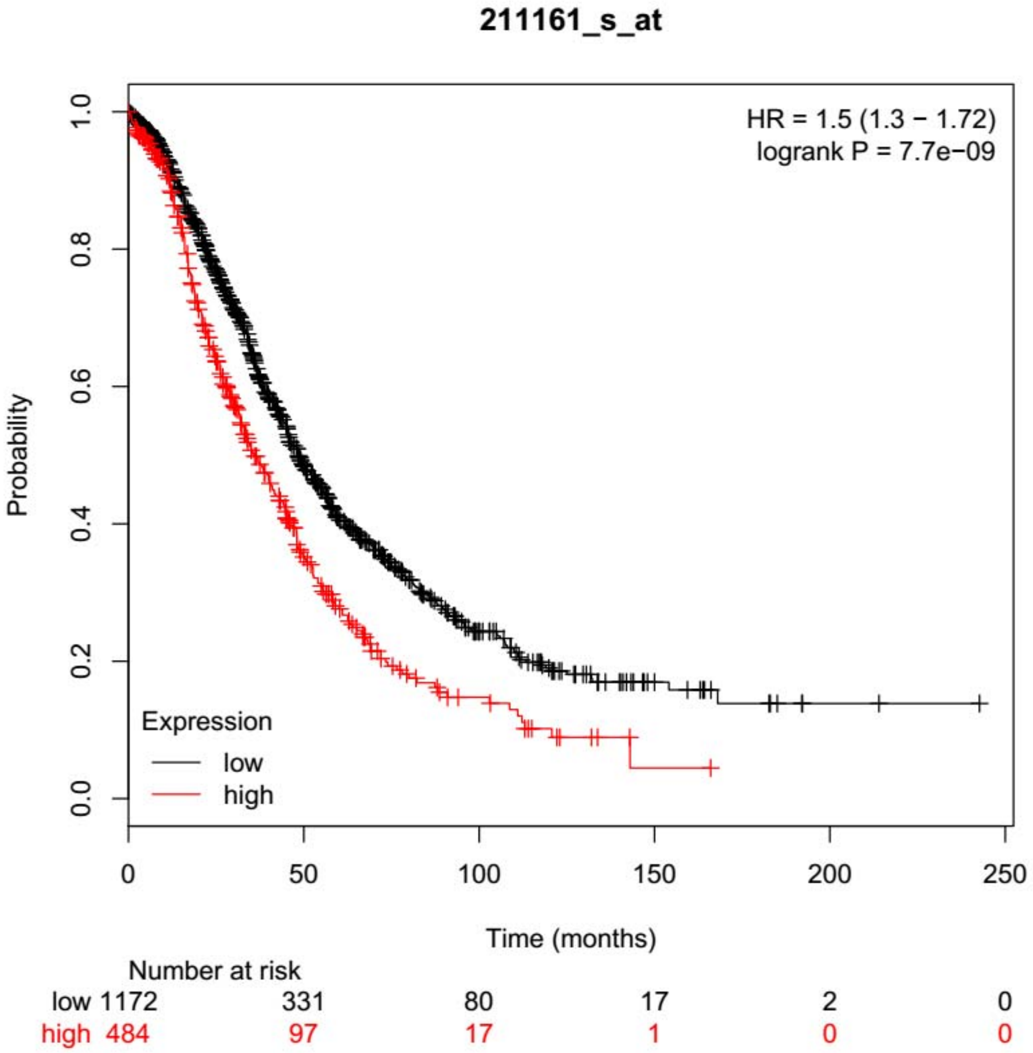
211161_s_at
HR = 1.5 (1.3 − 1.72)
logrank P = 7.7e−09
Probability
1.0
0.8
0.6
0.4
0.2
0.0
Expression
low
high
0
50
100
150
200
250
Time (months)
Number at risk
low 1172 331 80 17 2 0
high 484 97 17 1 0 0

6.COL6A6

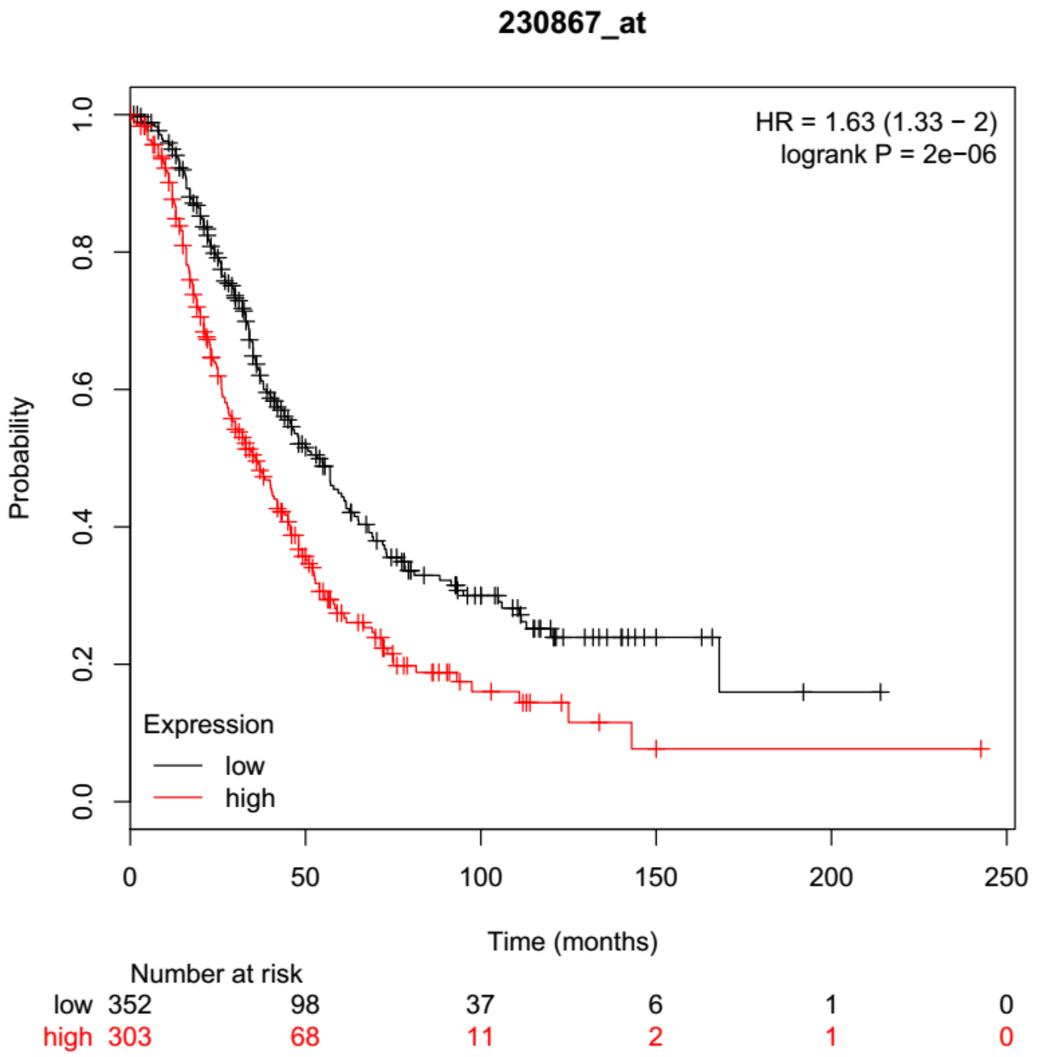
230867_at
HR = 1.63 (1.33 − 2)
logrank P = 2e−06
Probability
1.0
0.8
0.6
0.4
0.2
0.0
Expression
low
high
0
50
100
150
200
250
Time (months)
Number at risk
low 352 98 37 6 1 0
high 303 68 11 2 1 0

7. COL10A1

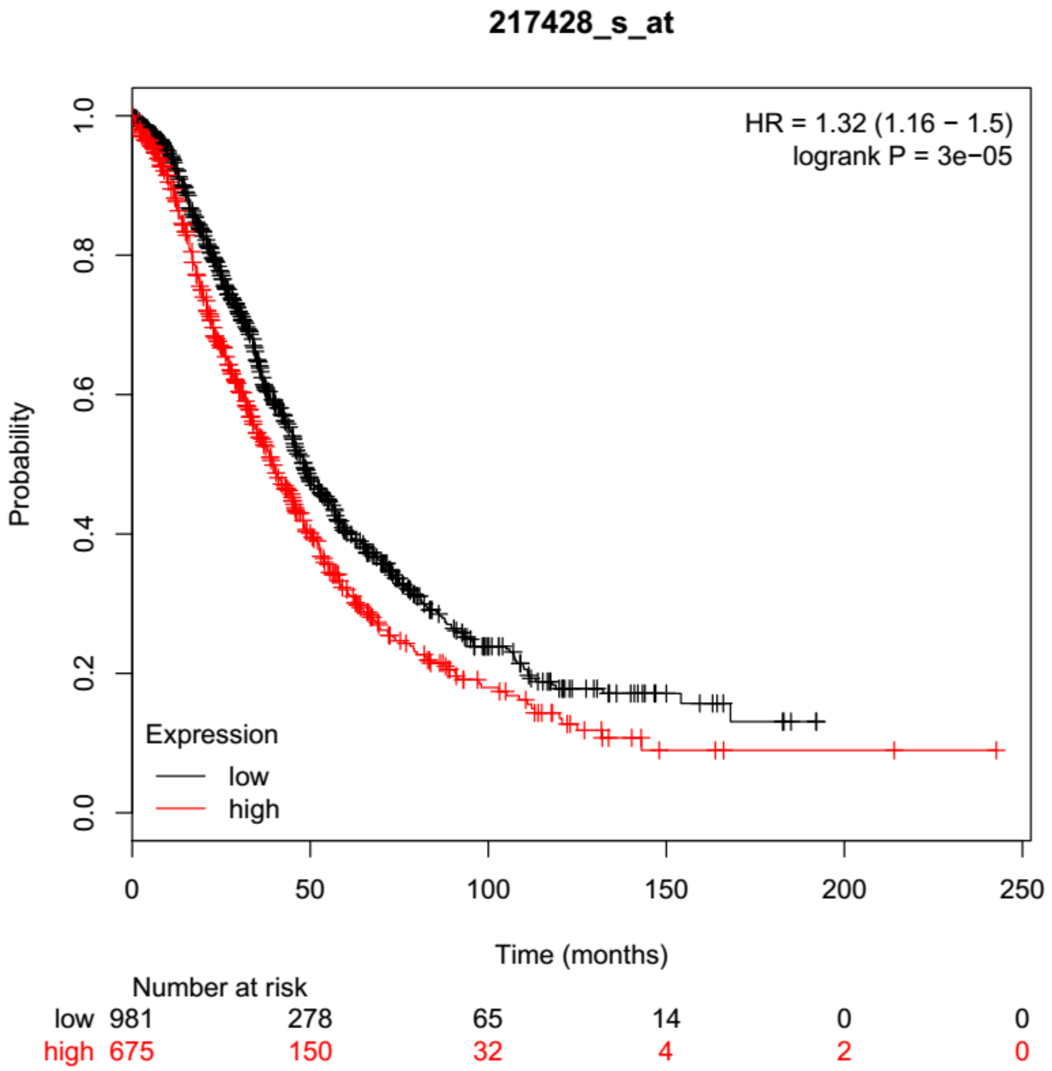
217428_s_at
HR = 1.32 (1.16 − 1.5)
logrank P = 3e−05
Probability
1.0
0.8
0.6
0.4
0.2
0.0
Expression
low
high
0
50
100
150
200
250
Time (months)
Number at risk
low 981 278 65 14 0 0
high 675 150 32 4 2 0

8. COL16A1

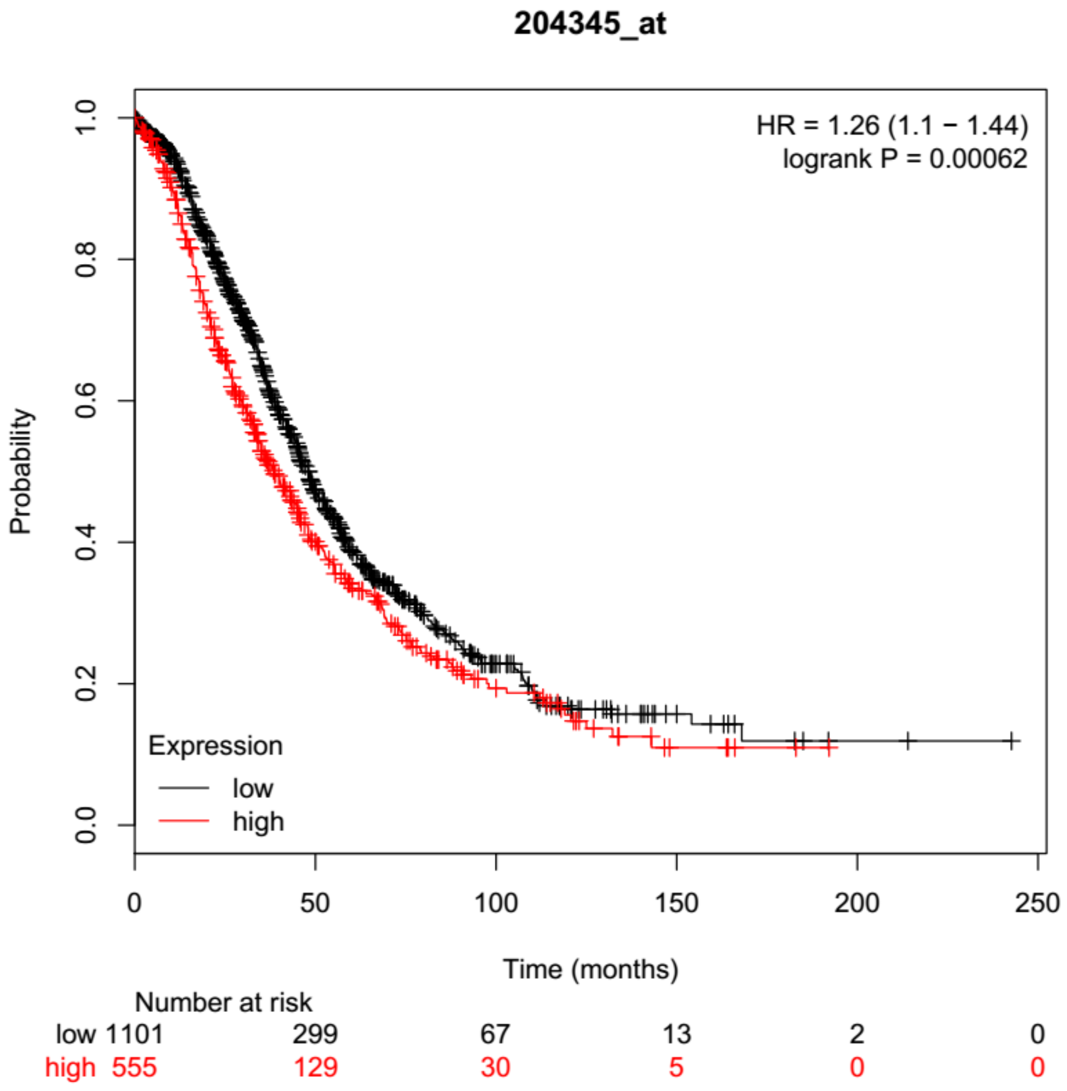

204345_at
HR = 1.26 (1.1 − 1.44)
logrank P = 0.00062
Probability
0.0
0.2
0.4
0.6
0.8
1.0
Expression
low
high
0
50
100
150
200
250
Time (months)
Number at risk
low 1101 299 67 13 2 0
high 555 129 30 5 0 0

9. DHRS2

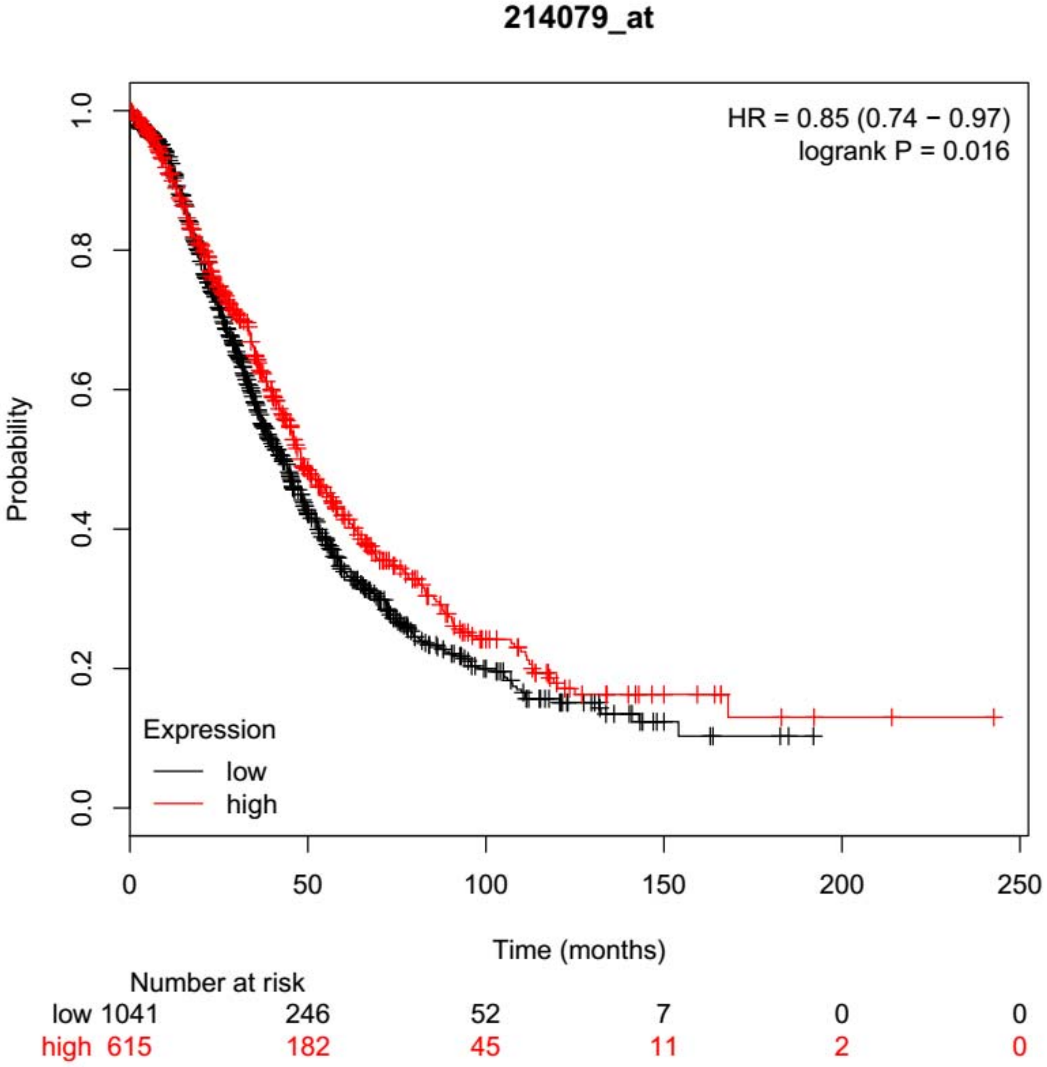
214079_at
HR = 0.85 (0.74 − 0.97)
logrank P = 0.016
Probability
0.0
0.2
0.4
0.6
0.8
1.0
Expression
low
high
0
50
100
150
200
250
Time (months)
Number at risk
low 1041 246 52 7 0 0
high 615 182 45 11 2 0

10. DNAJB1

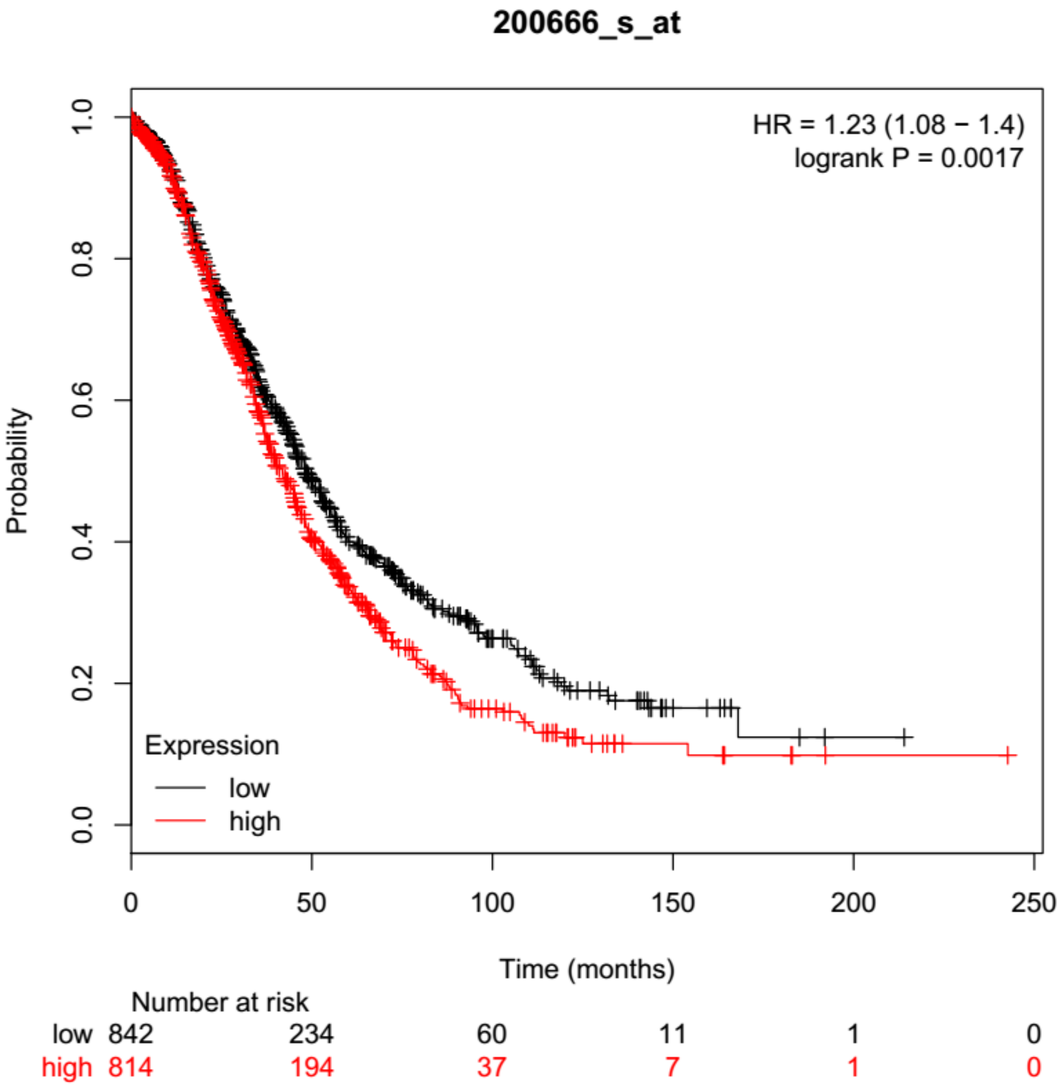

200666_s_at
HR = 1.23 (1.08 − 1.4)
logrank P = 0.0017
Probability
0.0
0.2
0.4
0.6
0.8
1.0
Expression
low
high
0
50
100
150
200
250
Time (months)
Number at risk
low 842 234 60 11 1 0
high 814 194 37 7 1 0

11. ERO1L

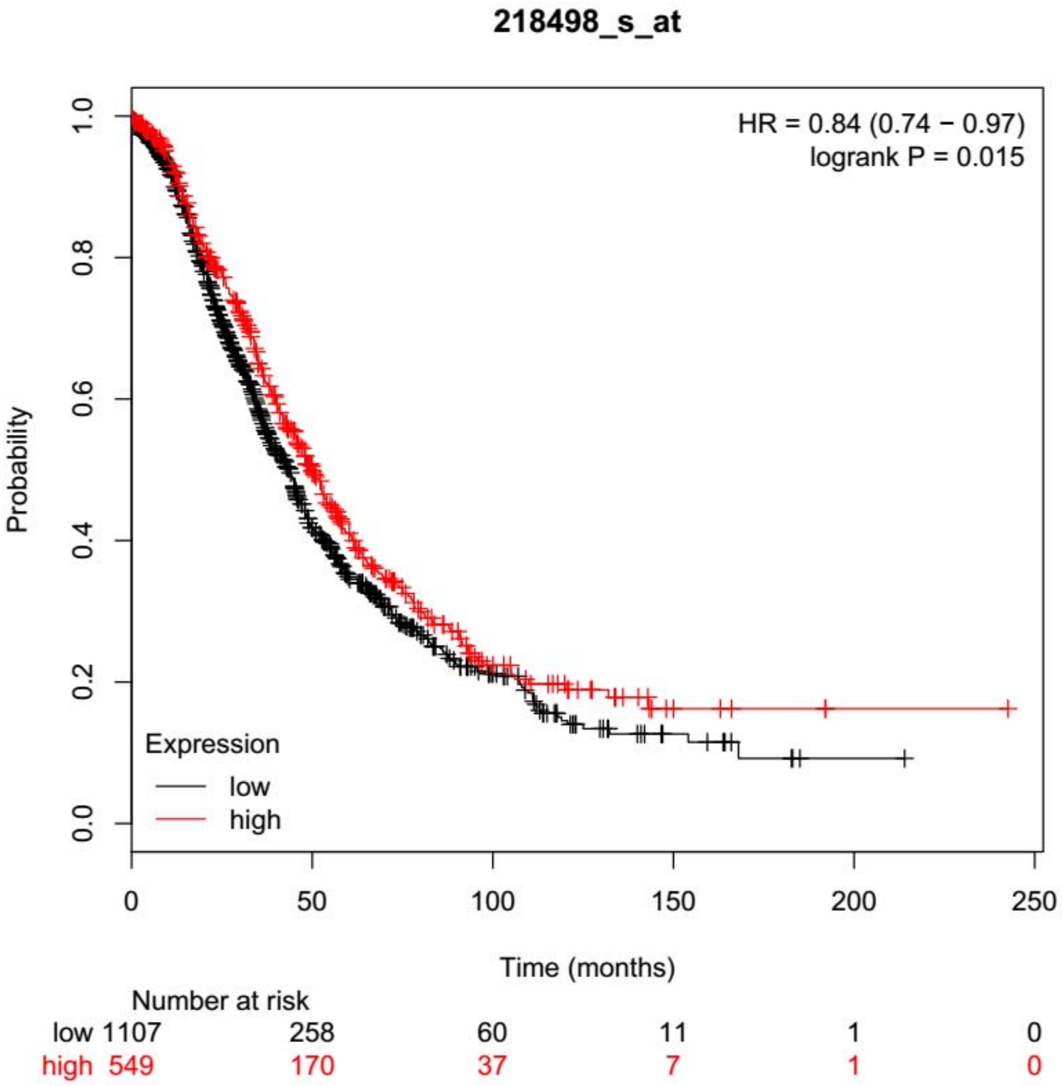
218498_s_at
HR = 0.84 (0.74 − 0.97)
logrank P = 0.015
Probability
0.0
0.2
0.4
0.6
0.8
1.0
Expression
low
high
0
50
100
150
200
250
Time (months)
Number at risk
low 1107
258
60
11
1
0
high 549
170
37
7
1
0

12. GFM1

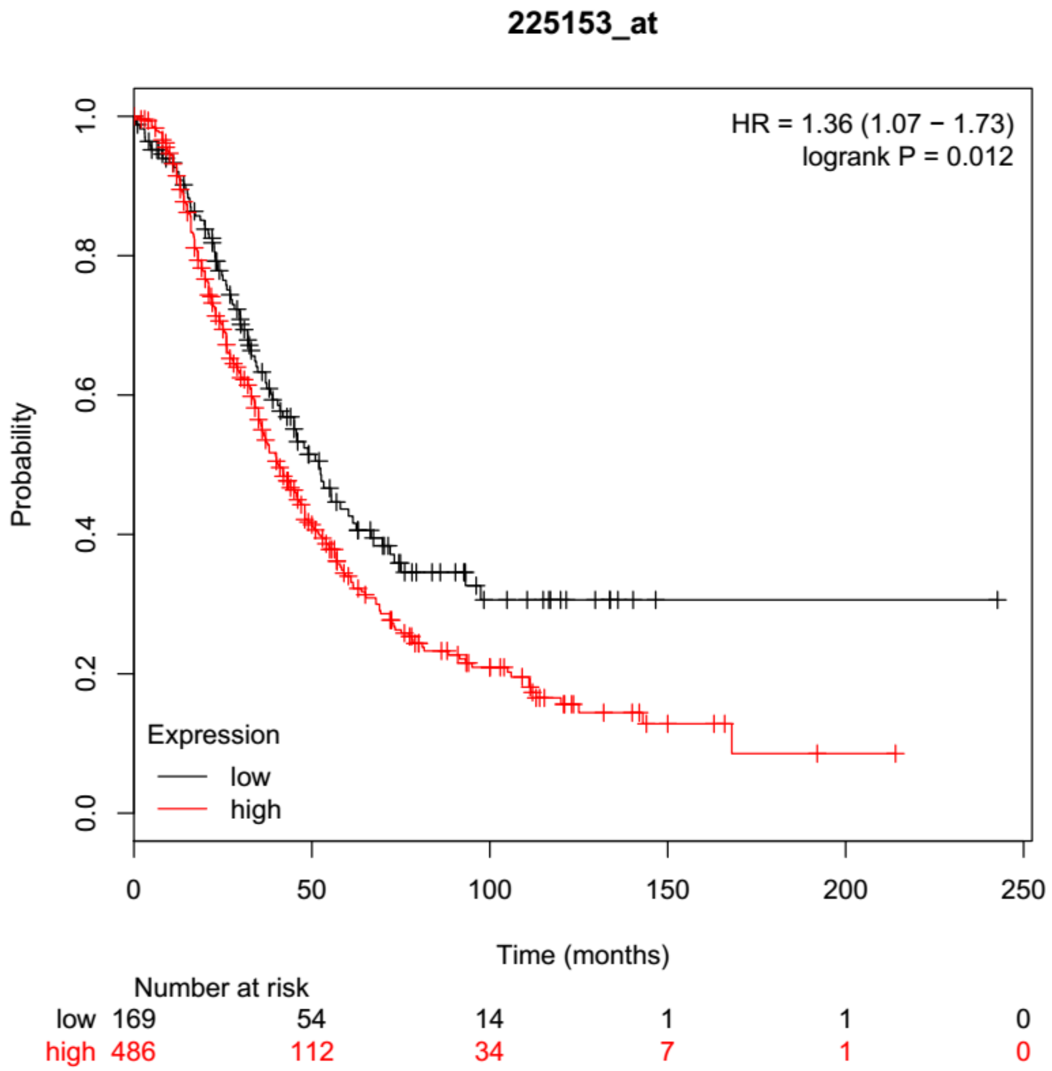

225153_at
HR = 1.36 (1.07 − 1.73)
logrank P = 0.012
Probability
0.0
0.2
0.4
0.6
0.8
1.0
Expression
low
high
0
50
100
150
200
250
Time (months)
Number at risk
low 169 54 14 1 1 0
high 486 112 34 7 1 0

13. GGT5

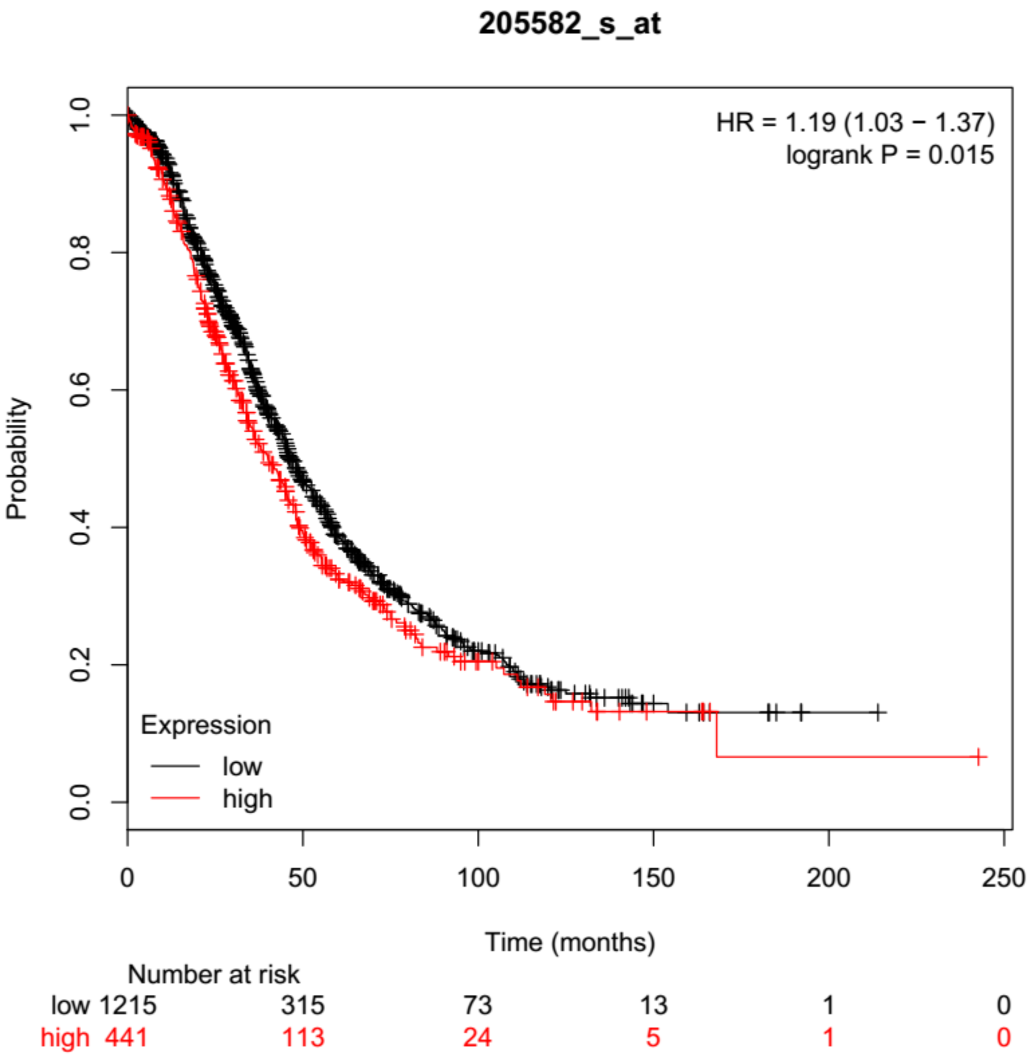
205582_s_at
HR = 1.19 (1.03 − 1.37)
logrank P = 0.015
Probability
0.0
0.2
0.4
0.6
0.8
1.0
Expression
low
high
0
50
100
150
200
250
Time (months)
Number at risk
low 1215
315
73
13
1
0
high 441
113
24
5
1
0

14. GLDC

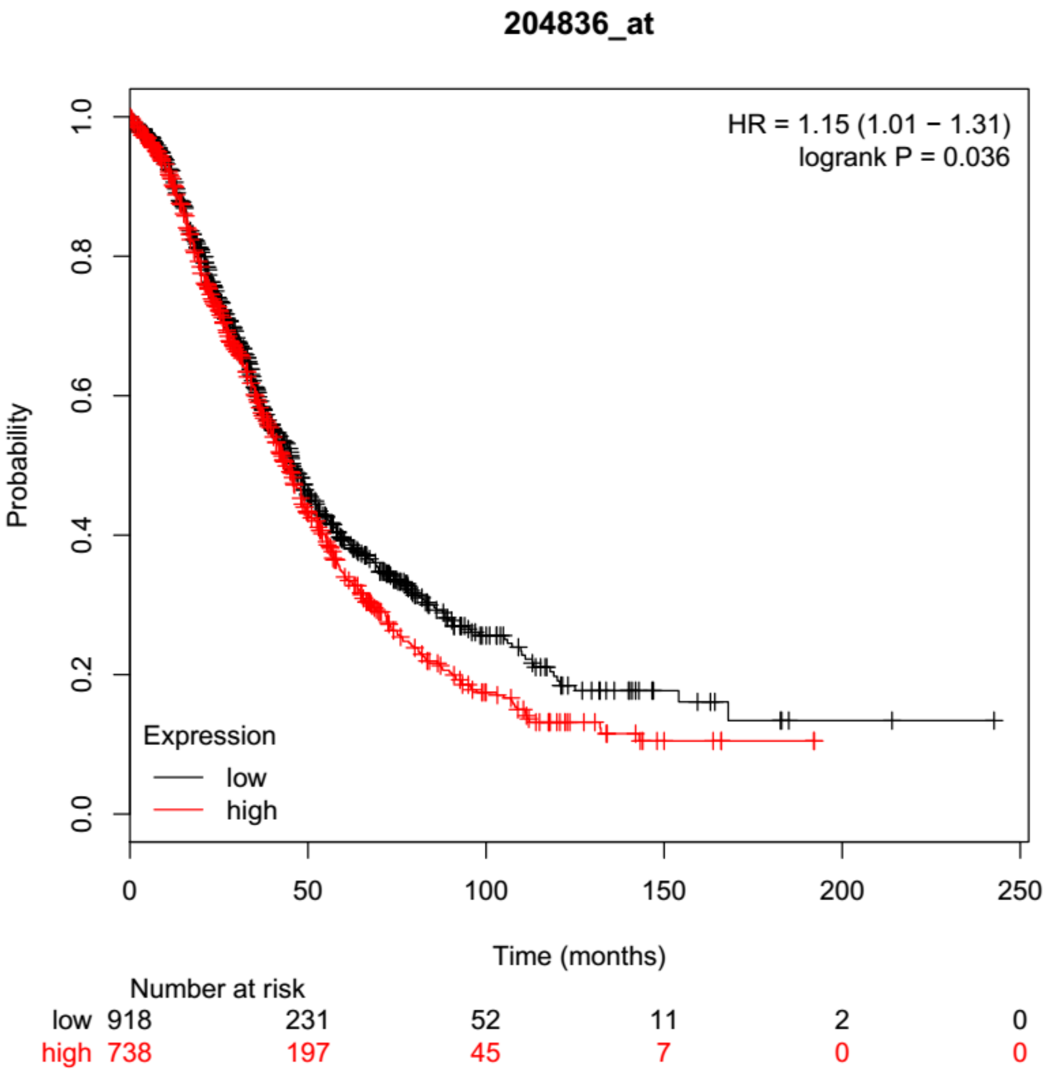

204836_at
HR = 1.15 (1.01 − 1.31)
logrank P = 0.036
Probability
1.0
0.8
0.6
0.4
0.2
0.0
Expression
low
high
0
50
100
150
200
250
Time (months)
Number at risk
low 918 231 52 11 2 0
high 738 197 45 7 0 0

15. GNAI1

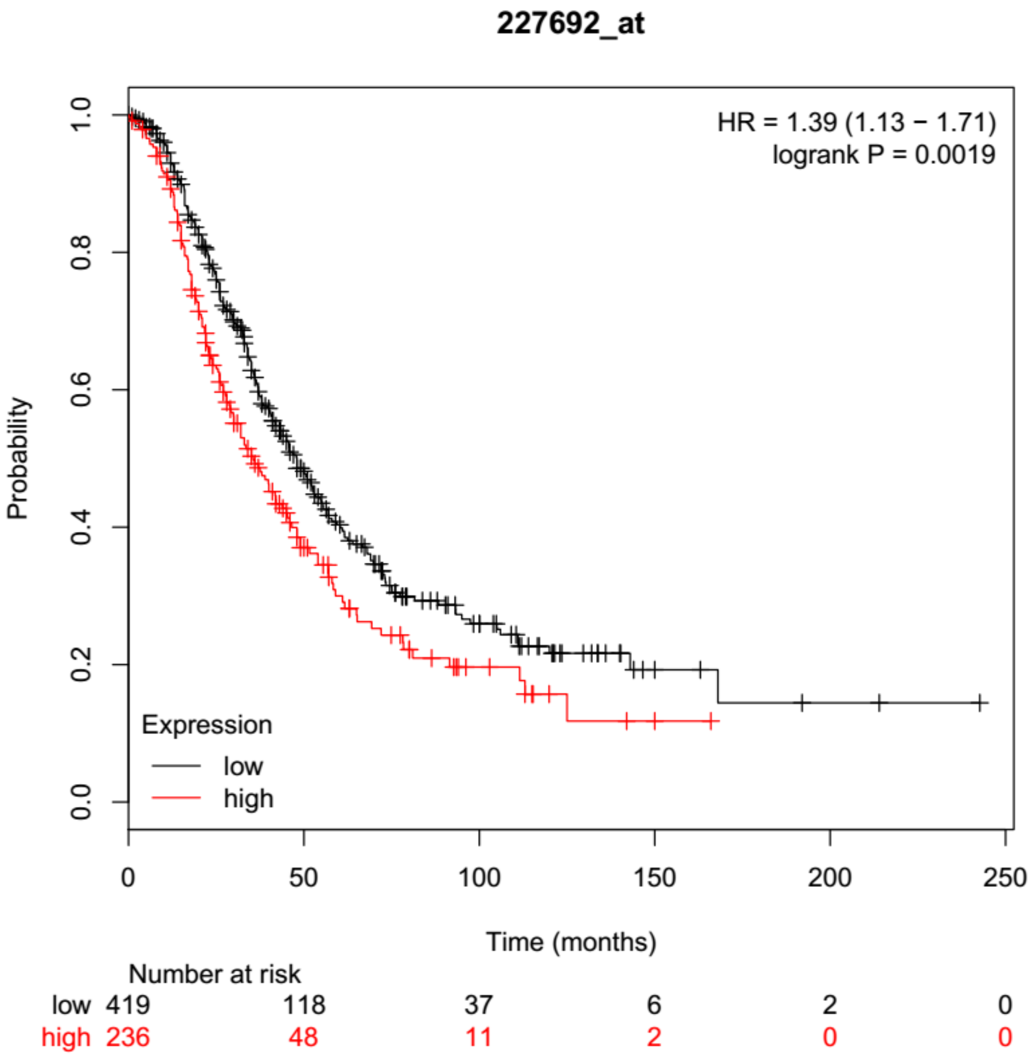

227692_at
HR = 1.39 (1.13 − 1.71)
logrank P = 0.0019
Probability
0.0
0.2
0.4
0.6
0.8
1.0
Expression
low
high
0
50
100
150
200
250
Time (months)
Number at risk
low 419 118 37 6 2 0
high 236 48 11 2 0 0

16. GNB4

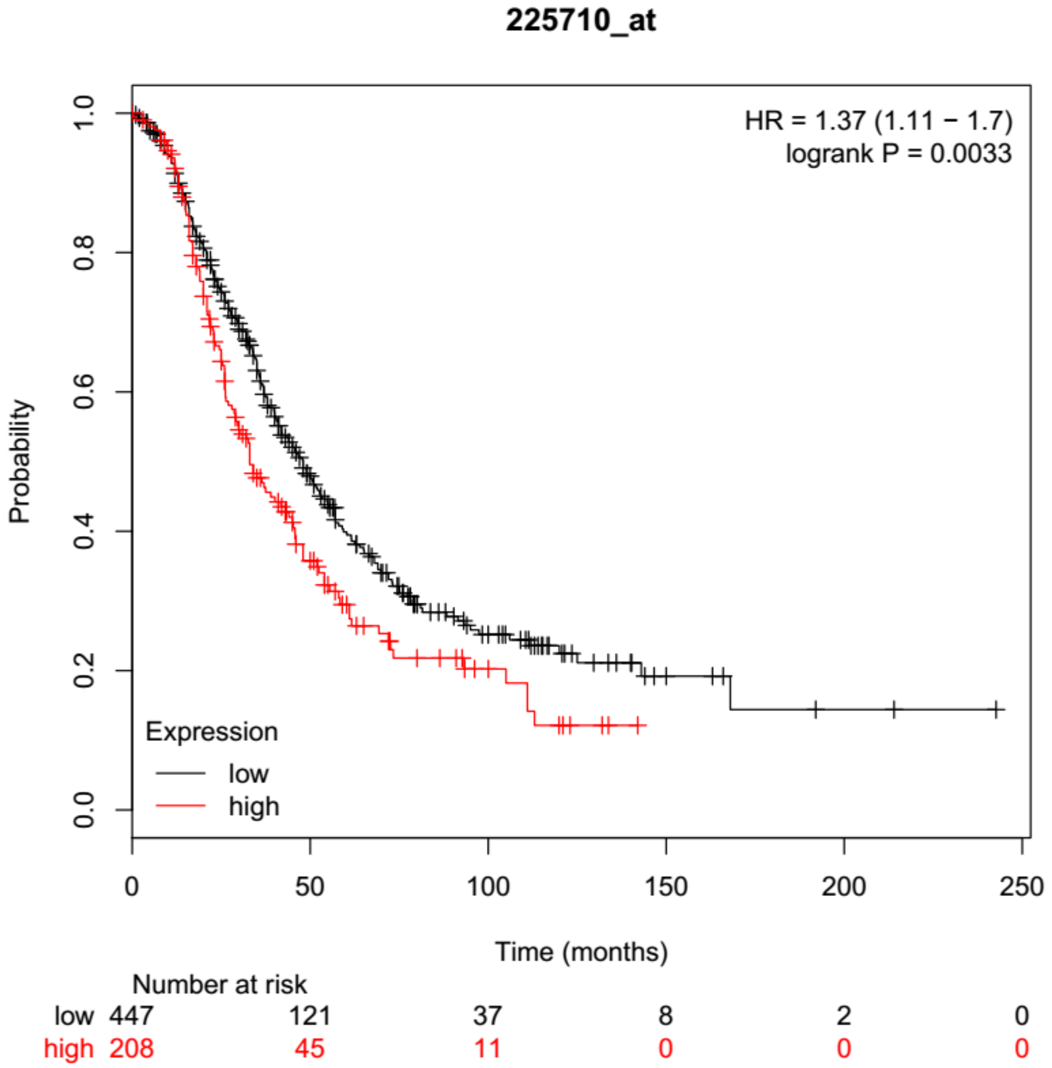

225710_at
HR = 1.37 (1.11 − 1.7)
logrank P = 0.0033
Probability
0.0
0.2
0.4
0.6
0.8
1.0
Expression
low
high
0
50
100
150
200
250
Time (months)
Number at risk
low 447 121 37 8 2 0
high 208 45 11 0 0 0

17. GSTM5

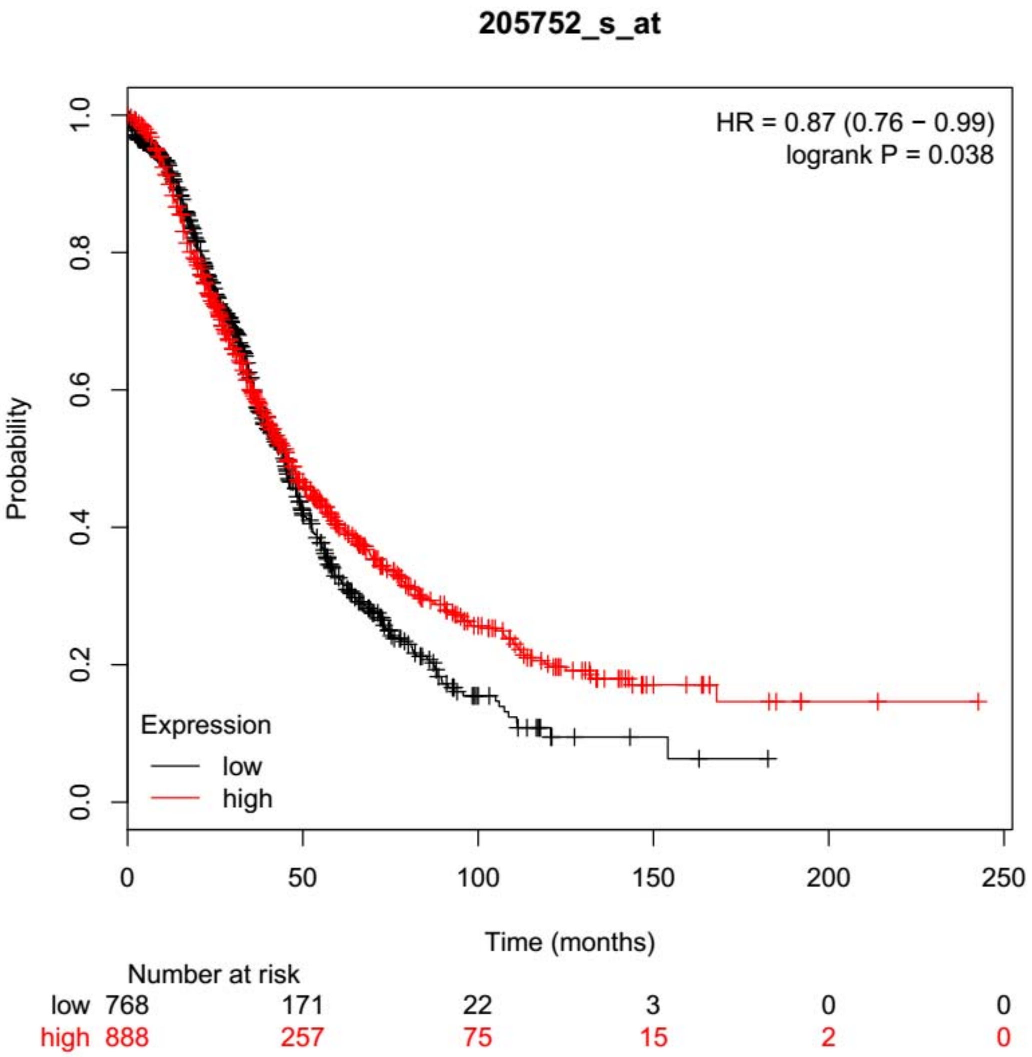
205752_s_at
HR = 0.87 (0.76 − 0.99)
logrank P = 0.038
Probability
0.0
0.2
0.4
0.6
0.8
1.0
Expression
low
high
0
50
100
150
200
250
Time (months)
Number at risk
low 768 171 22 3 0 0
high 888 257 75 15 2 0

18. H1F0

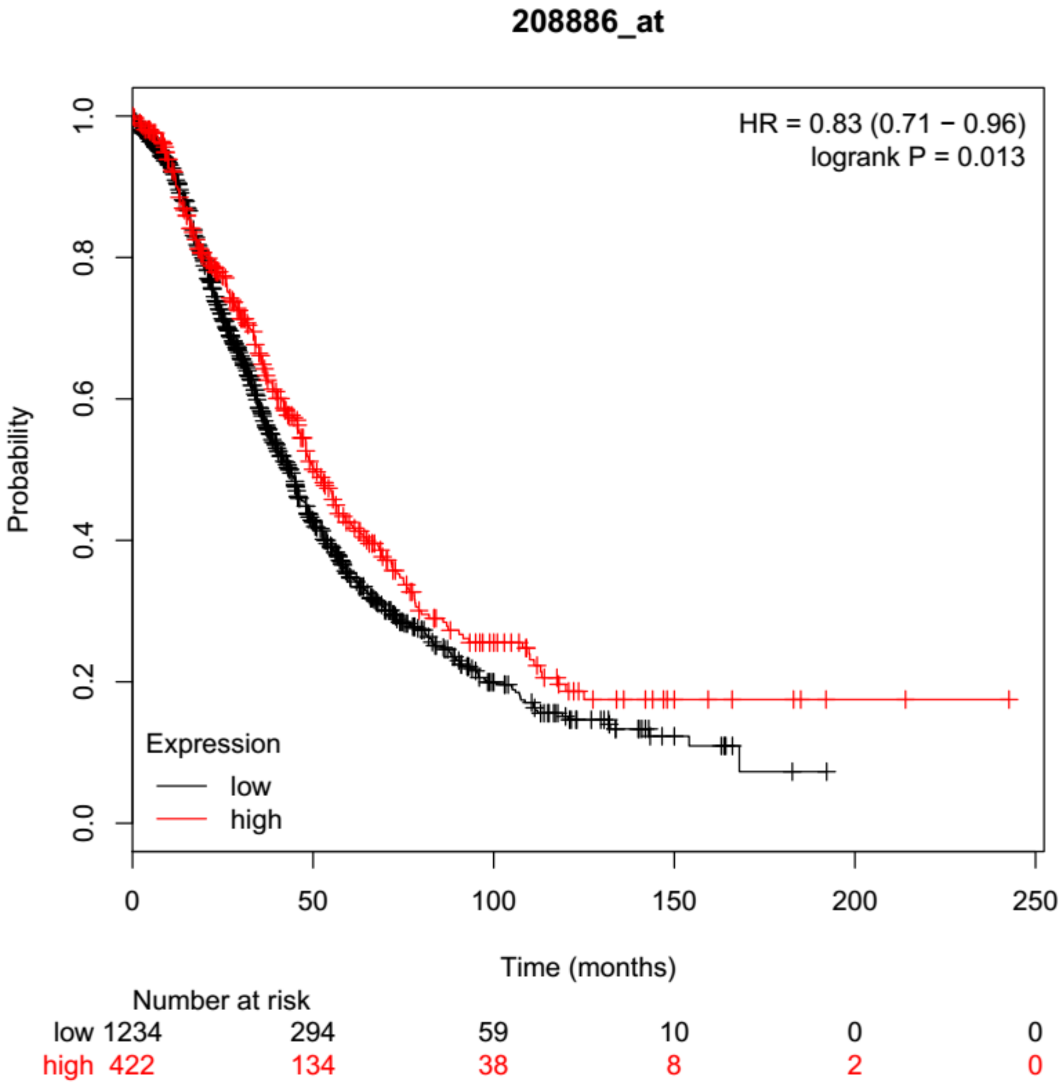
208886_at
HR = 0.83 (0.71 − 0.96)
logrank P = 0.013
Probability
0.0
0.2
0.4
0.6
0.8
1.0
Expression
low
high
0
50
100
150
200
250
Time (months)
Number at risk
low 1234
294
59
10
0
0
high 422
134
38
8
2
0

19. H1FX

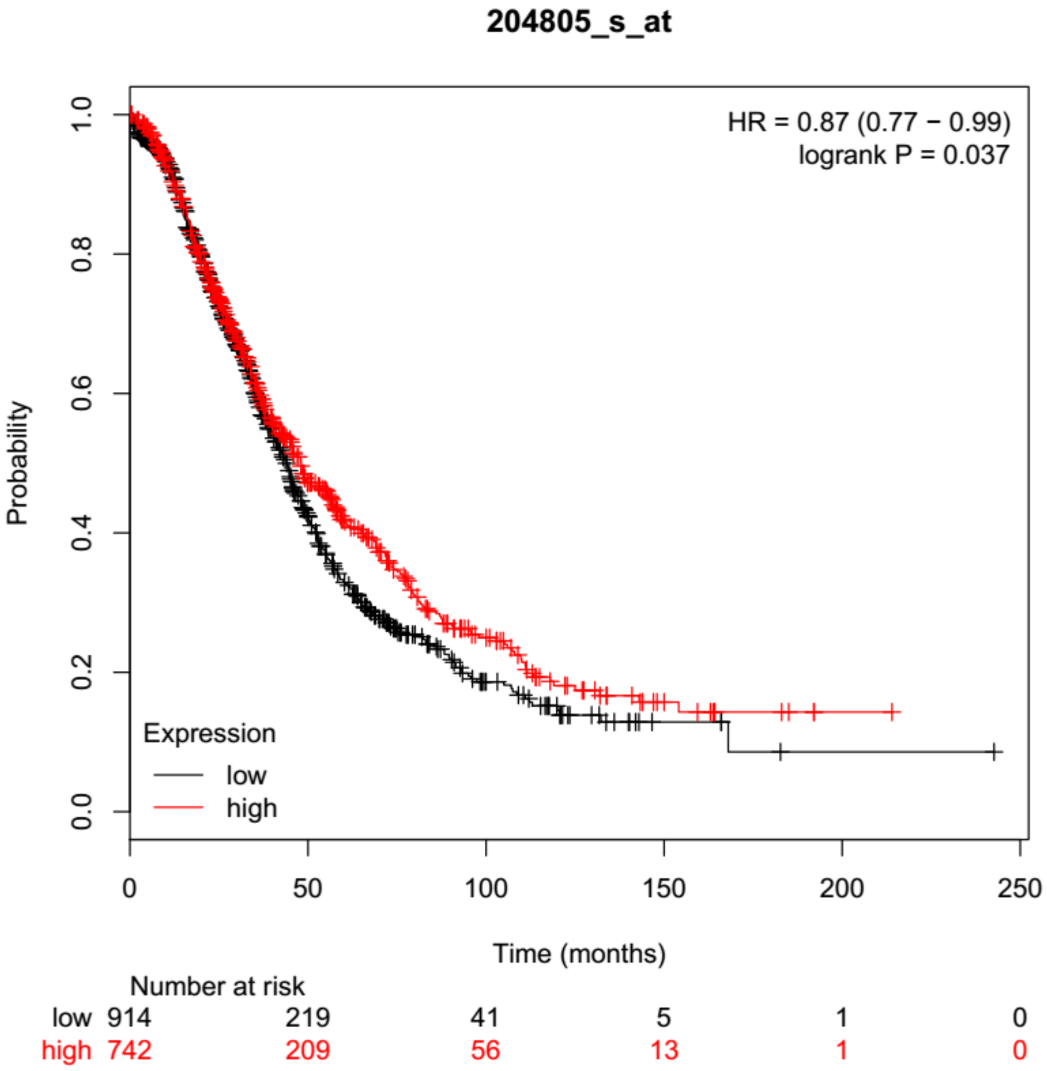
204805_s_at
HR = 0.87 (0.77 − 0.99)
logrank P = 0.037
Probability
0.0
0.2
0.4
0.6
0.8
1.0
Expression
low
high
0
50
100
150
200
250
Time (months)
Number at risk
low 914 219 41 5 1 0
high 742 209 56 13 1 0

20. H2AFY

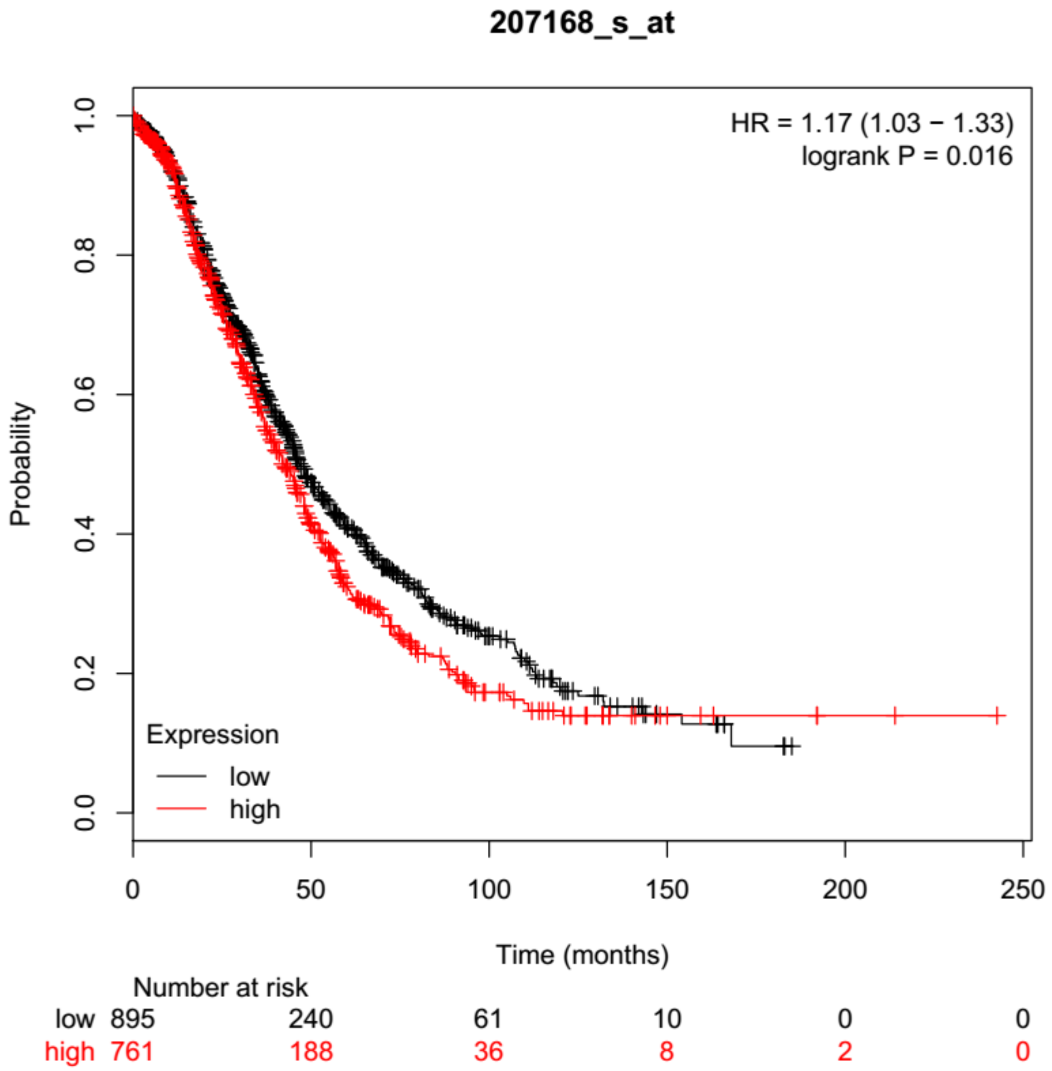
207168_s_at
HR = 1.17 (1.03 − 1.33)
logrank P = 0.016
Probability
0.0
0.2
0.4
0.6
0.8
1.0
Expression
low
high
0
50
100
150
200
250
Time (months)
Number at risk
low 895 240 61 10 0 0
high 761 188 36 8 2 0

21. H2AFZ

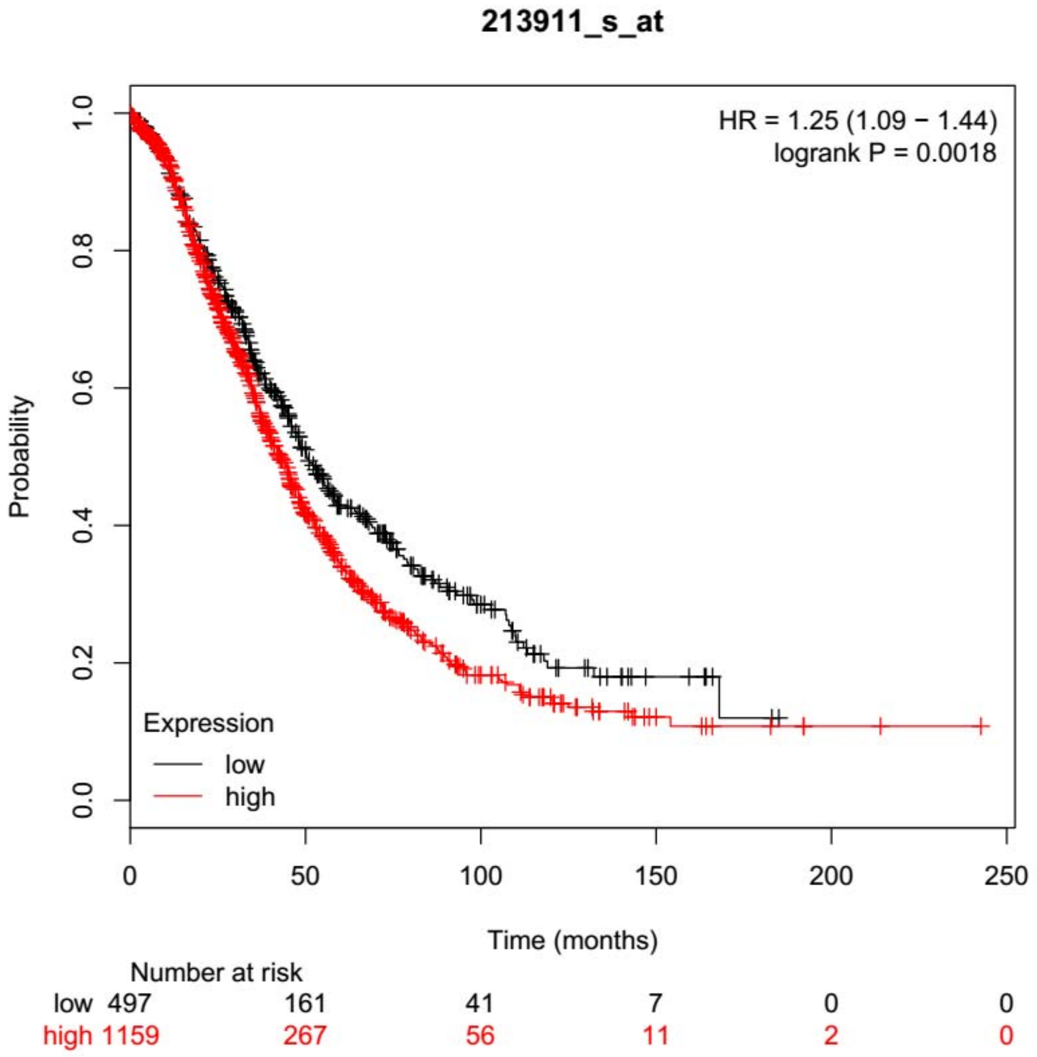

213911_s_at
HR = 1.25 (1.09 − 1.44)
logrank P = 0.0018
Probability
0.0
0.2
0.4
0.6
0.8
1.0
Expression
low
high
0
50
100
150
200
250
Time (months)
Number at risk
low 497 161 41 7 0 0
high 1159 267 56 11 2 0

22. HEBP1

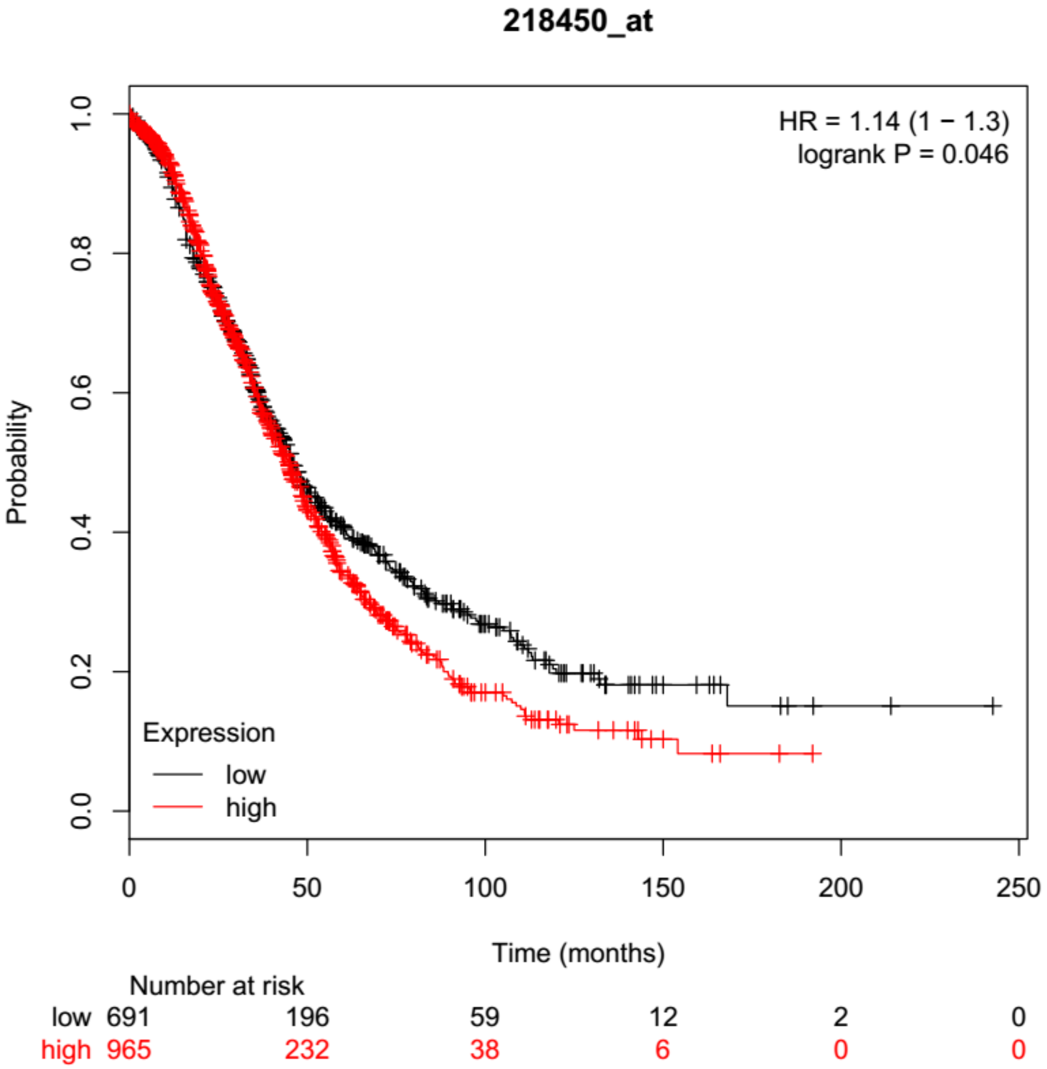

218450_at
HR = 1.14 (1 − 1.3)
logrank P = 0.046
Probability
0.0
0.2
0.4
0.6
0.8
1.0
Expression
low
high
0
50
100
150
200
250
Time (months)
Number at risk
low 691 196 59 12 2 0
high 965 232 38 6 0 0

23. HIBCH

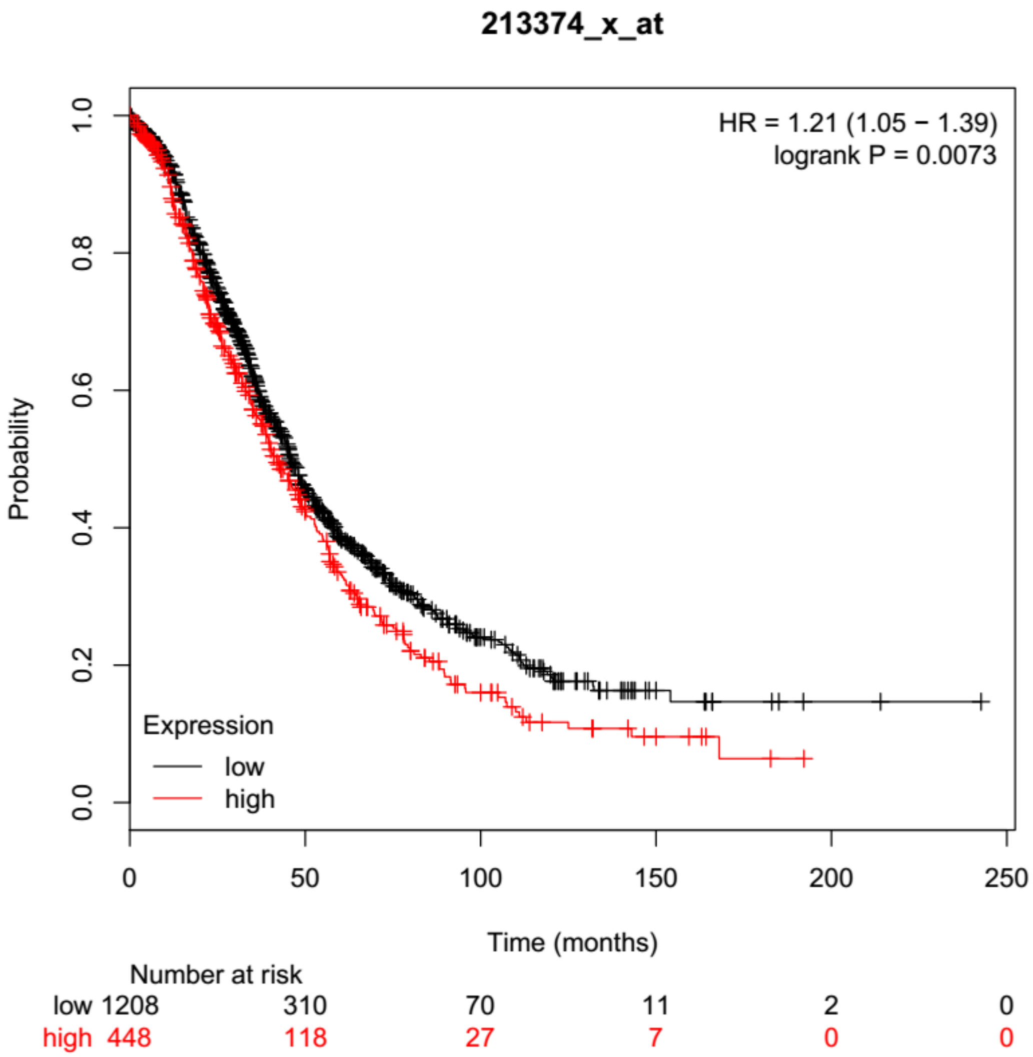
213374_x_at
HR = 1.21 (1.05 − 1.39)
logrank P = 0.0073
Probability
0.0
0.2
0.4
0.6
0.8
1.0
Expression
low
high
0
50
100
150
200
250
Time (months)
Number at risk
low 1208 310 70 11 2 0
high 448 118 27 7 0 0

24. HIST1H1C

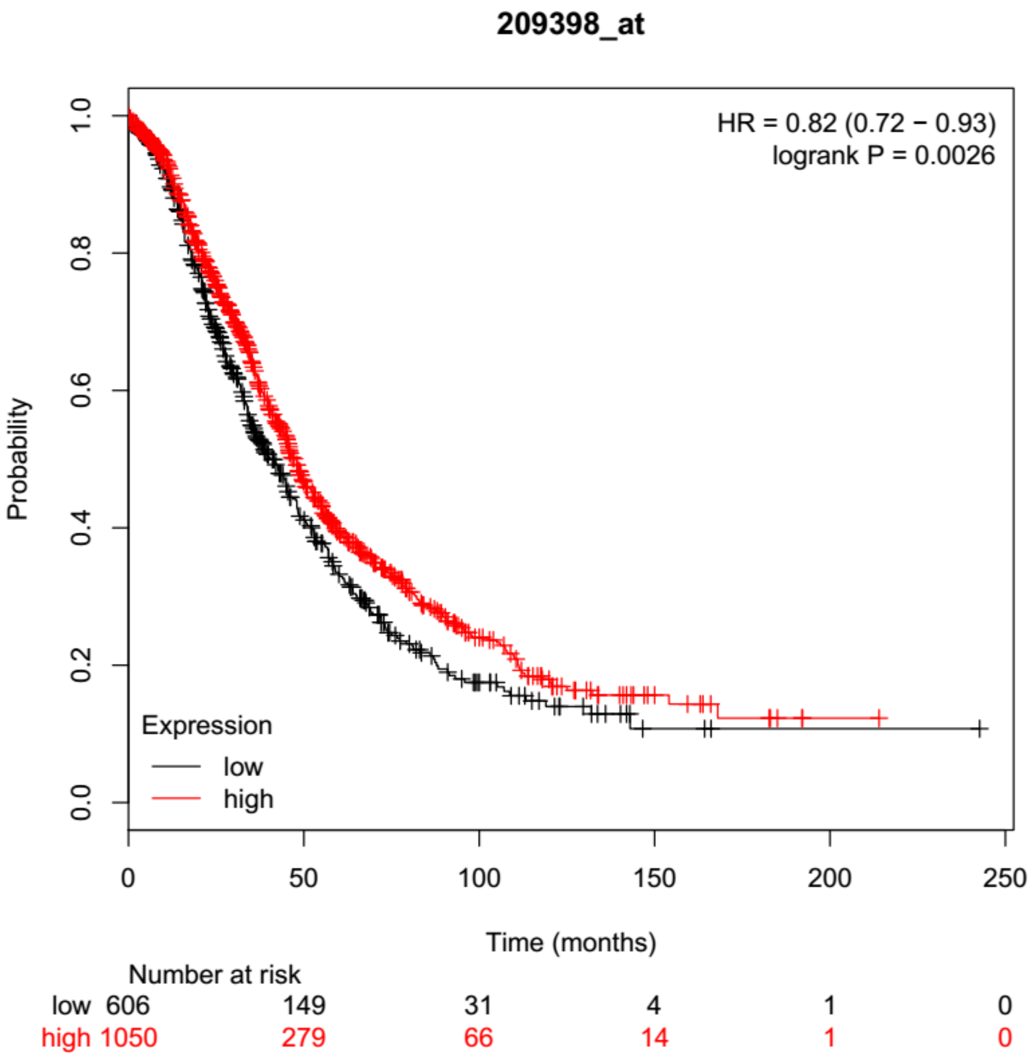

209398_at
HR = 0.82 (0.72 − 0.93)
logrank P = 0.0026
Probability
1.0
0.8
0.6
0.4
0.2
0.0
Expression
low
high
0
50
100
150
200
250
Time (months)
Number at risk
low 606 149 31 4 1 0
high 1050 279 66 14 1 0

25. HIST1H2BJ

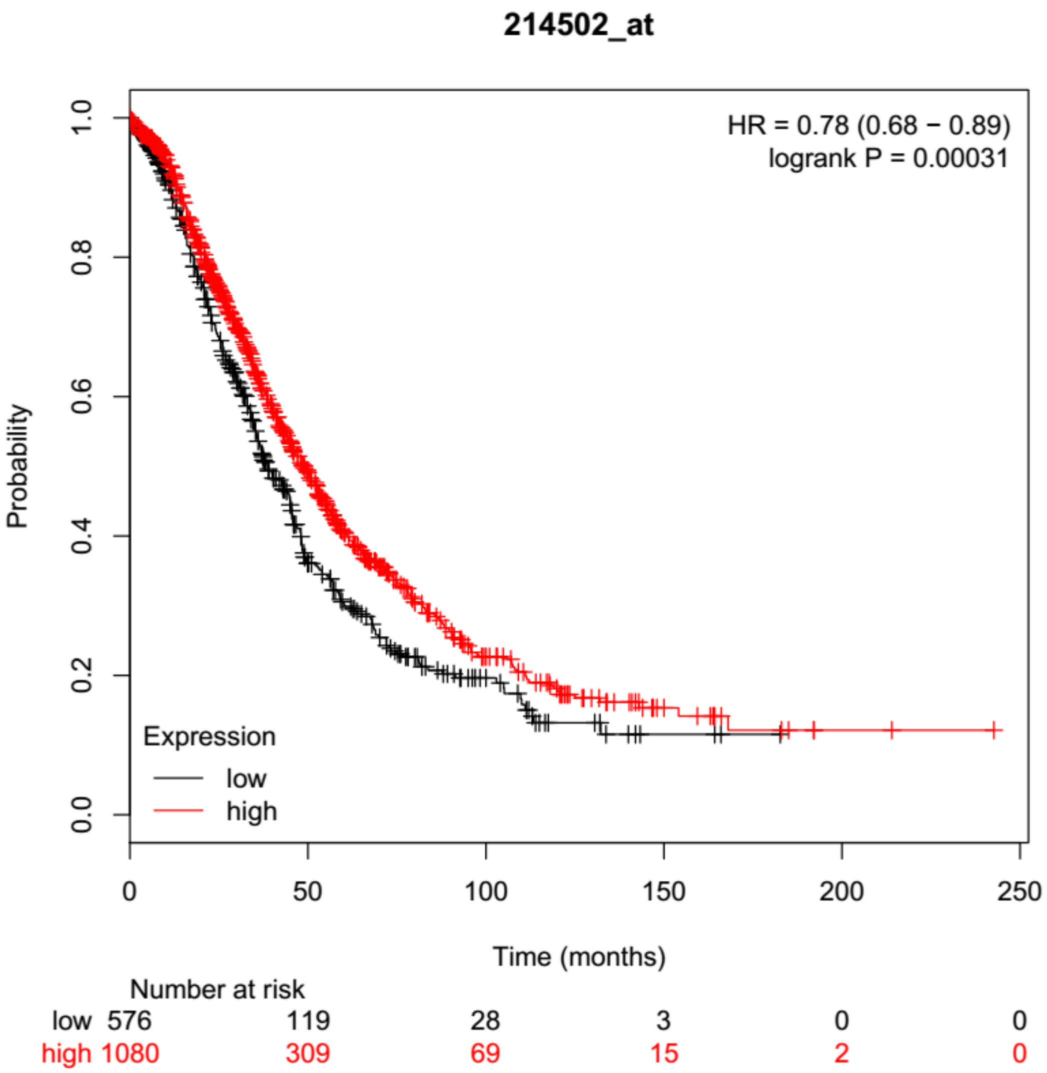

26. HIST1H2BK

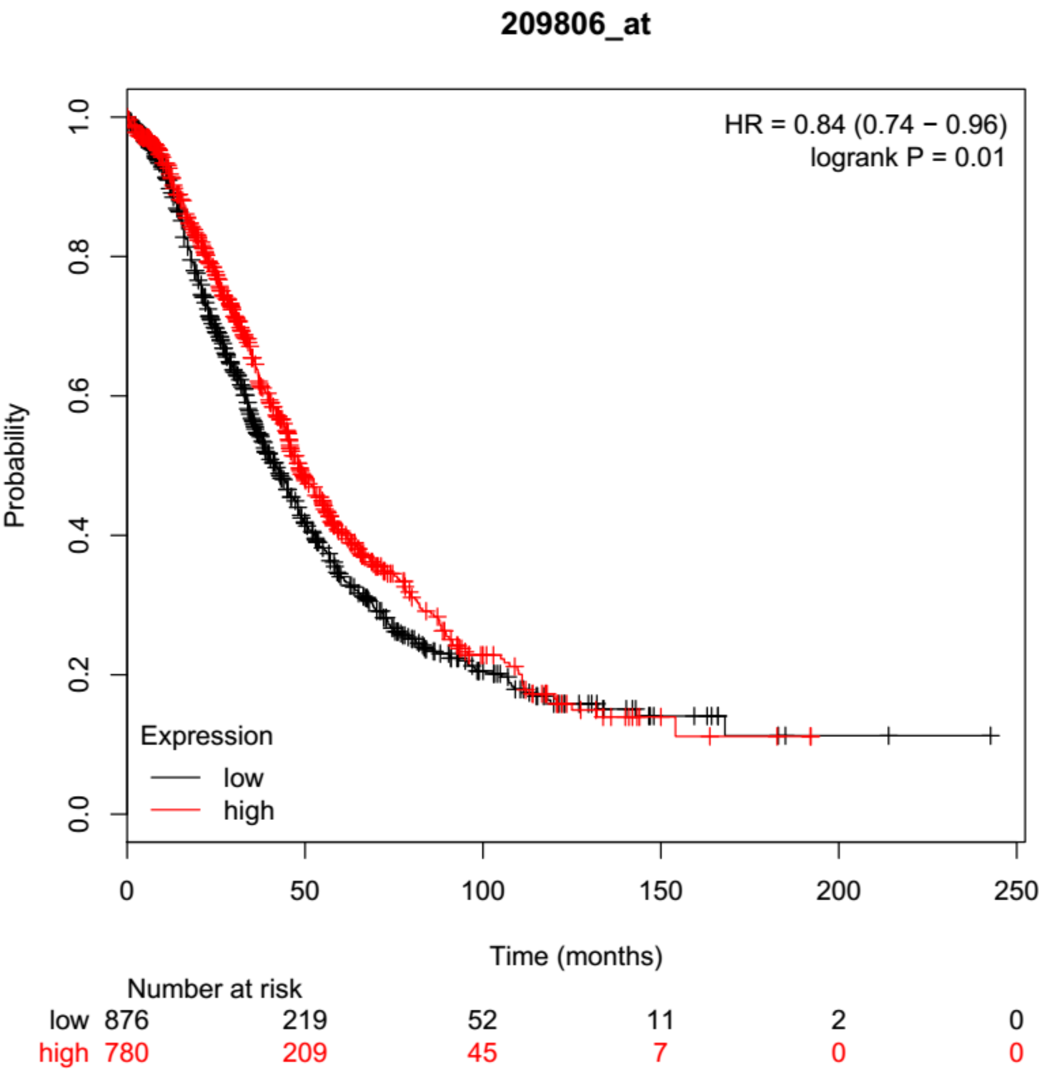

27. HIST1H4B

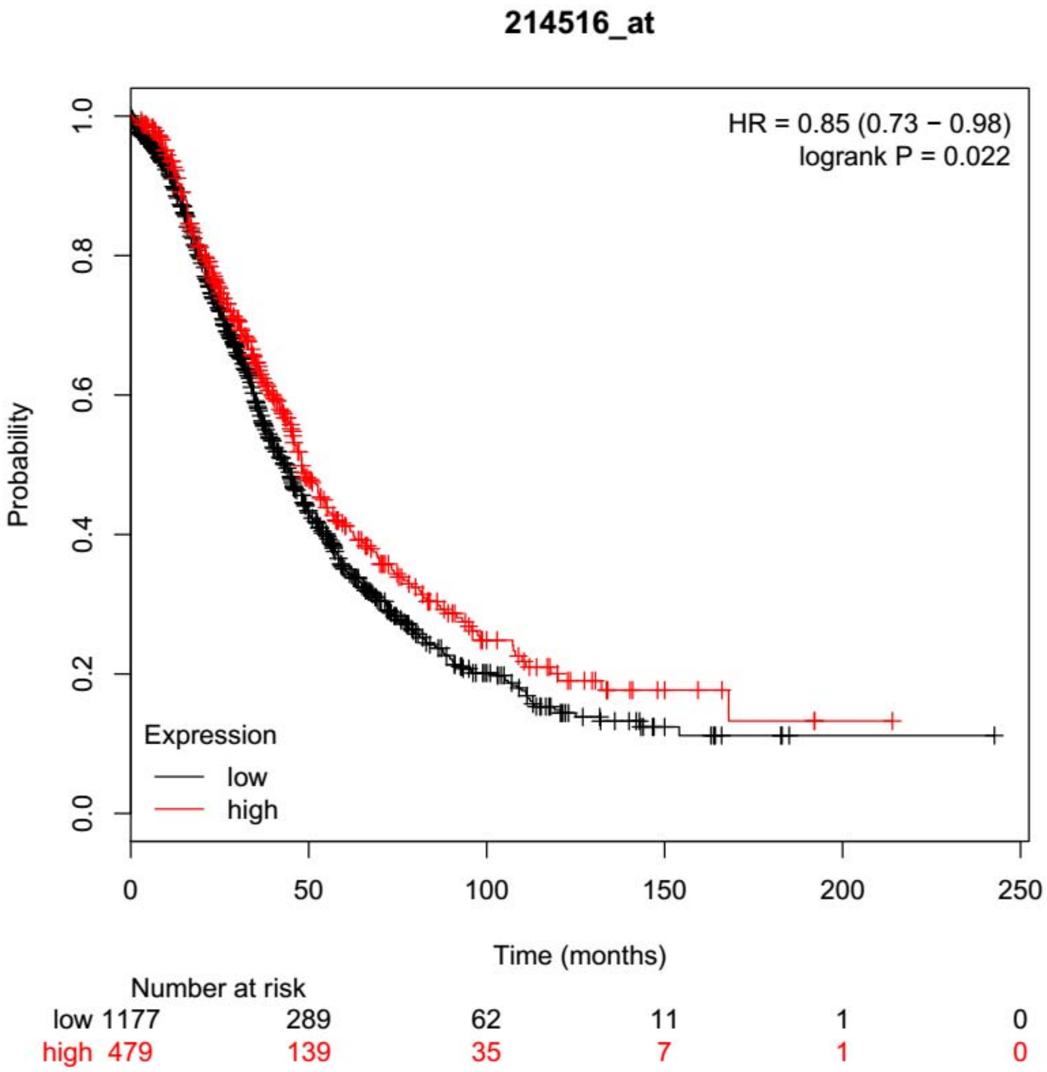
214516_at
HR = 0.85 (0.73 − 0.98)
logrank P = 0.022
Probability
1.0
0.8
0.6
0.4
0.2
0.0
Expression
low
high
0
50
100
150
200
250
Time (months)
Number at risk
low 1177
289
62
11
1
0
high 479
139
35
7
1
0

28. HIST1H4D

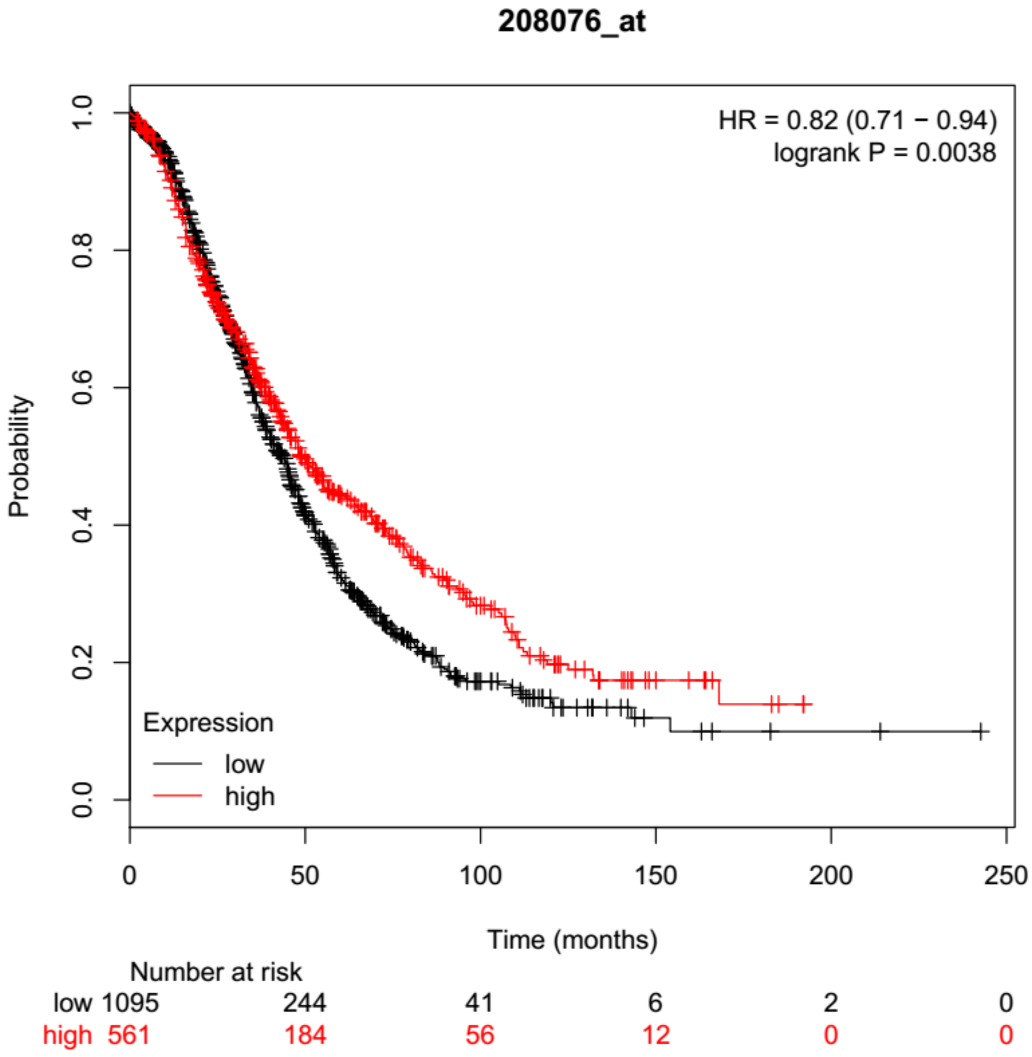

208076_at
HR = 0.82 (0.71 − 0.94)
logrank P = 0.0038
Probability
1.0
0.8
0.6
0.4
0.2
0.0
Expression
low
high
0
50
100
150
200
250
Time (months)
Number at risk
low 1095 244 41 6 2 0
high 561 184 56 12 0 0

29. HIST1H4F

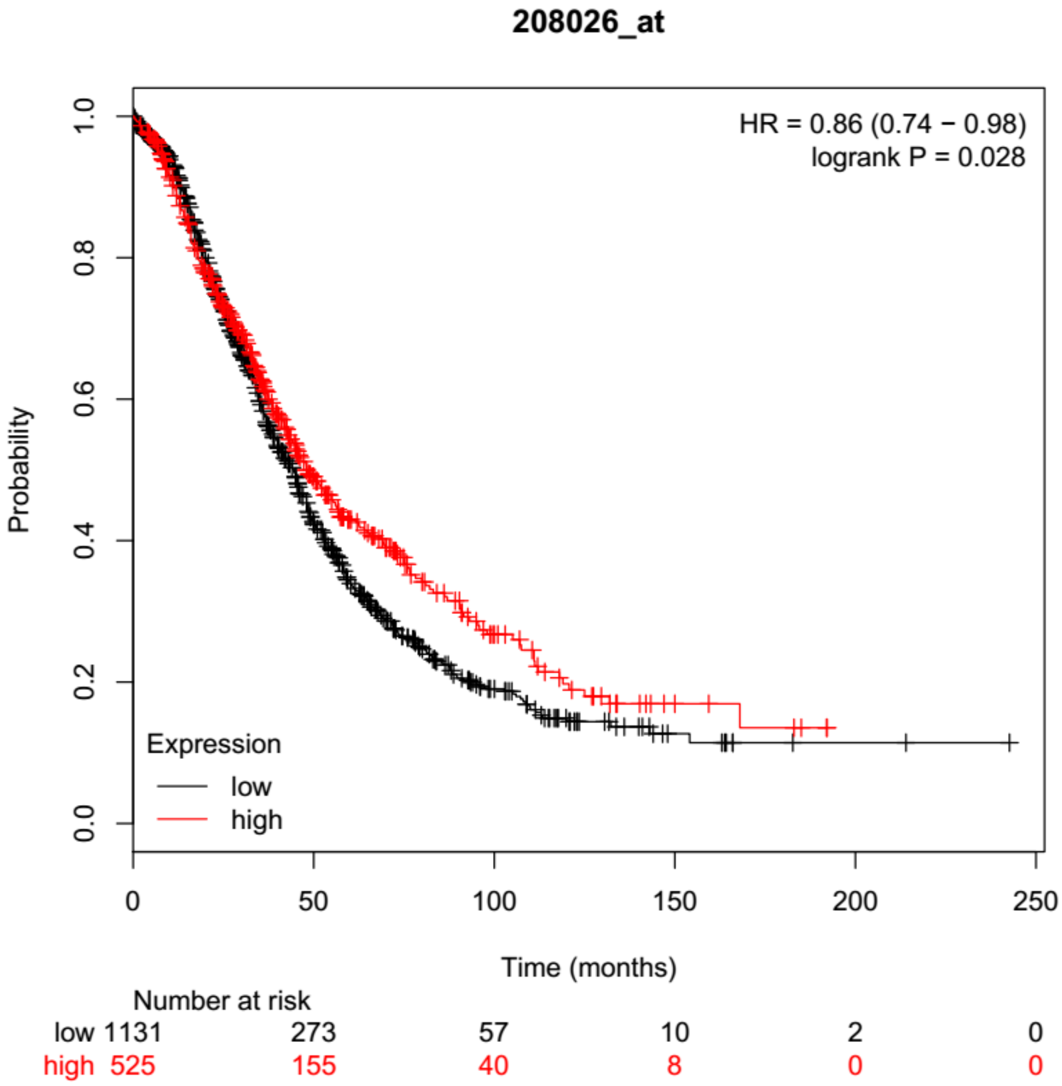
208026_at
HR = 0.86 (0.74 − 0.98)
logrank P = 0.028
Probability
0.0
0.2
0.4
0.6
0.8
1.0
Expression
low
high
0
50
100
150
200
250
Time (months)
Number at risk
low 1131 273 57 10 2 0
high 525 155 40 8 0 0

30. HIST1H4H

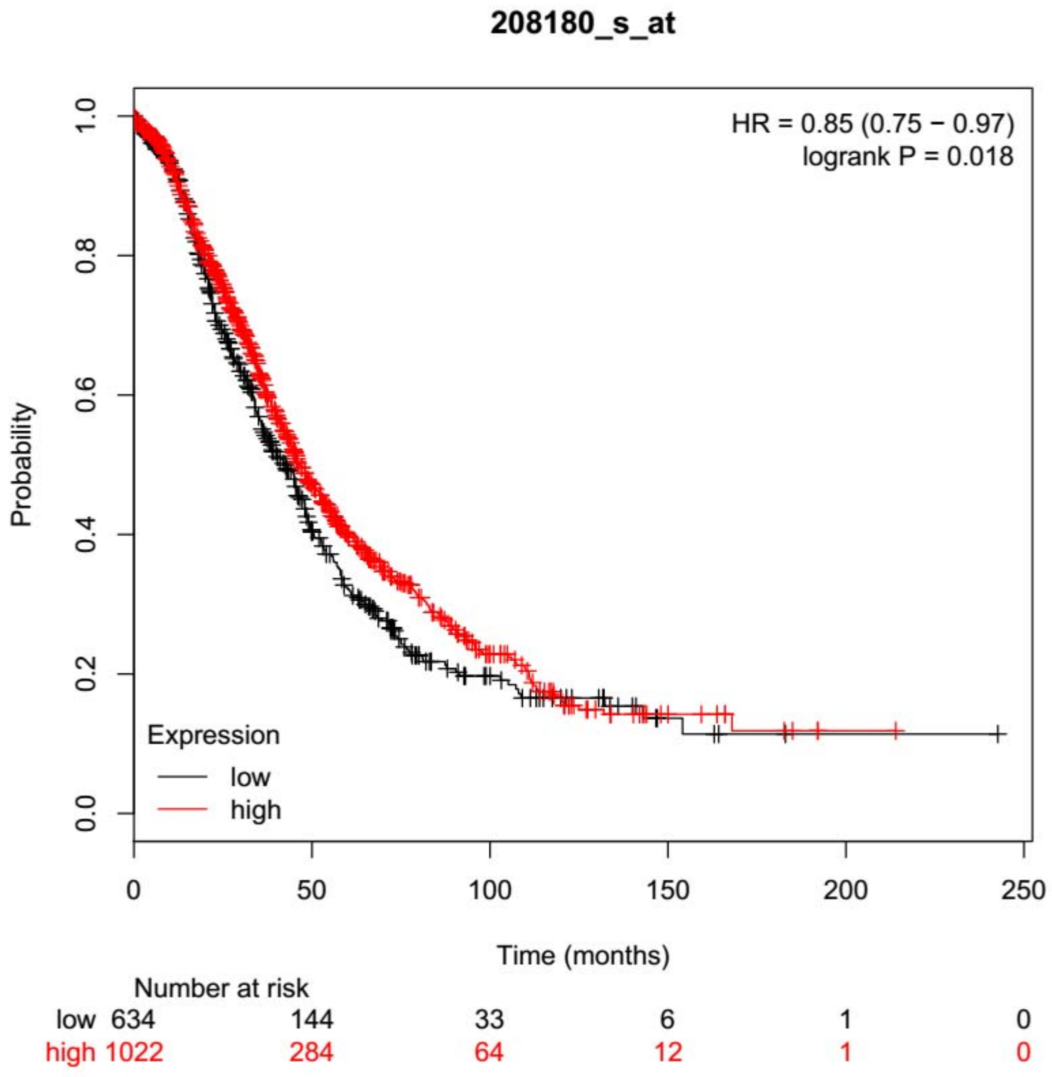

208180_s_at
HR = 0.85 (0.75 − 0.97)
logrank P = 0.018
Probability
0.0
0.2
0.4
0.6
0.8
1.0
Expression
low
high
0
50
100
150
200
250
Time (months)
Number at risk
low 634 144 33 6 1 0
high 1022 284 64 12 1 0

31. HIST1H4J

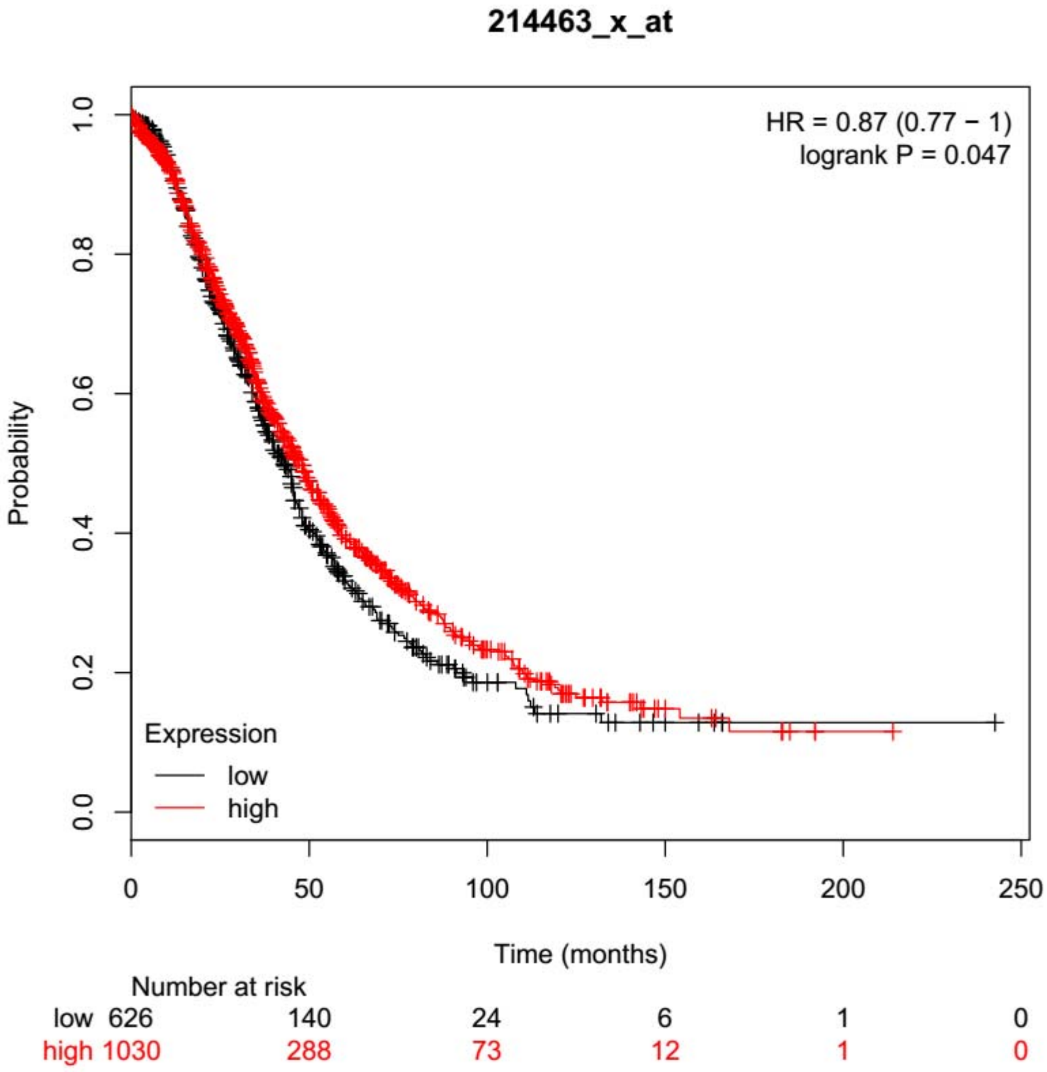
214463_x_at
HR = 0.87 (0.77 − 1)
logrank P = 0.047
Probability
0.0
0.2
0.4
0.6
0.8
1.0
Expression
low
high
0
50
100
150
200
250
Time (months)
Number at risk
low 626 140 24 6 1 0
high 1030 288 73 12 1 0

32. HSD11B1

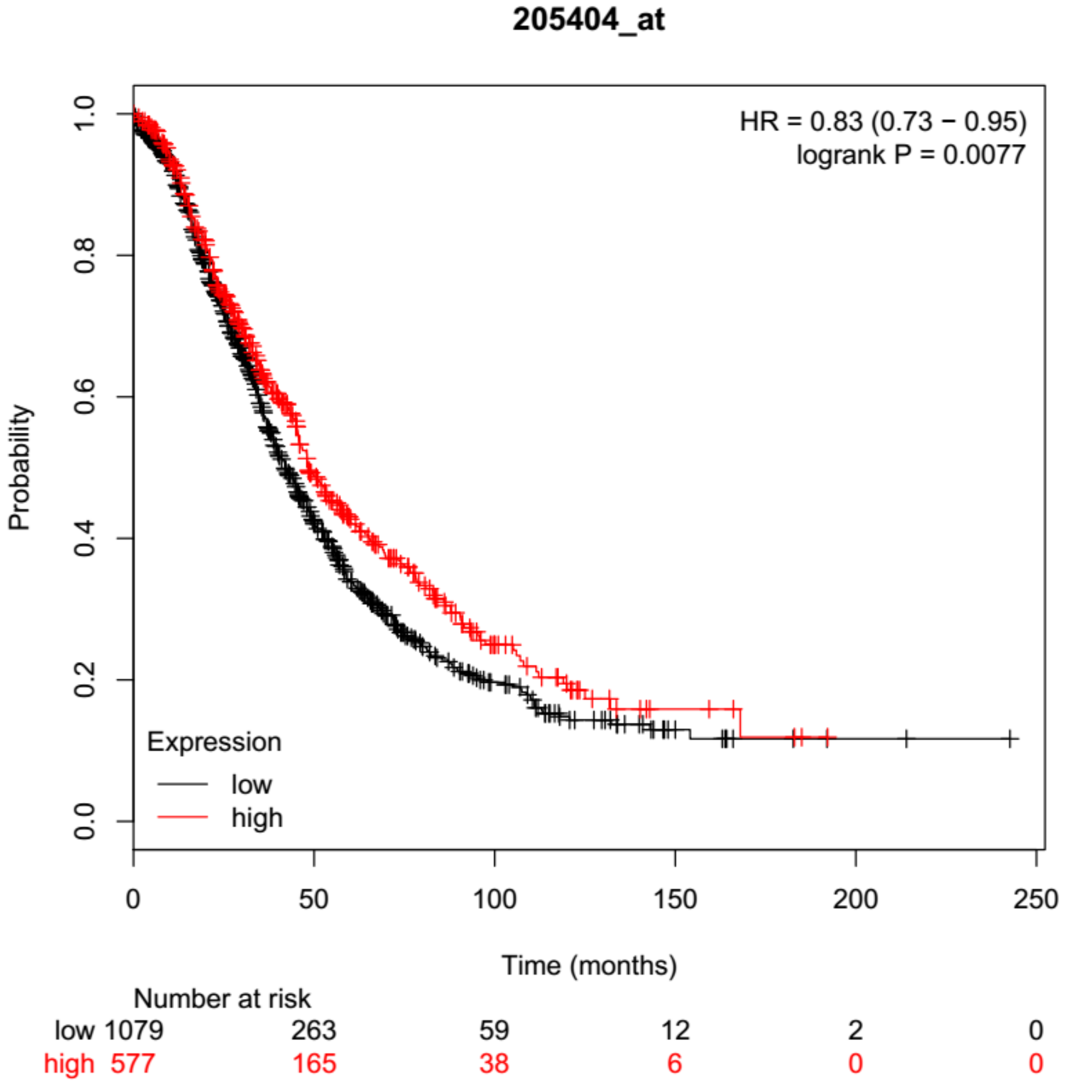
205404_at
HR = 0.83 (0.73 − 0.95)
logrank P = 0.0077
Probability
1.0
0.8
0.6
0.4
0.2
0.0
Expression
low
high
0
50
100
150
200
250
Time (months)
Number at risk
low 1079 263 59 12 2 0
high 577 165 38 6 0 0

33. HYOU1

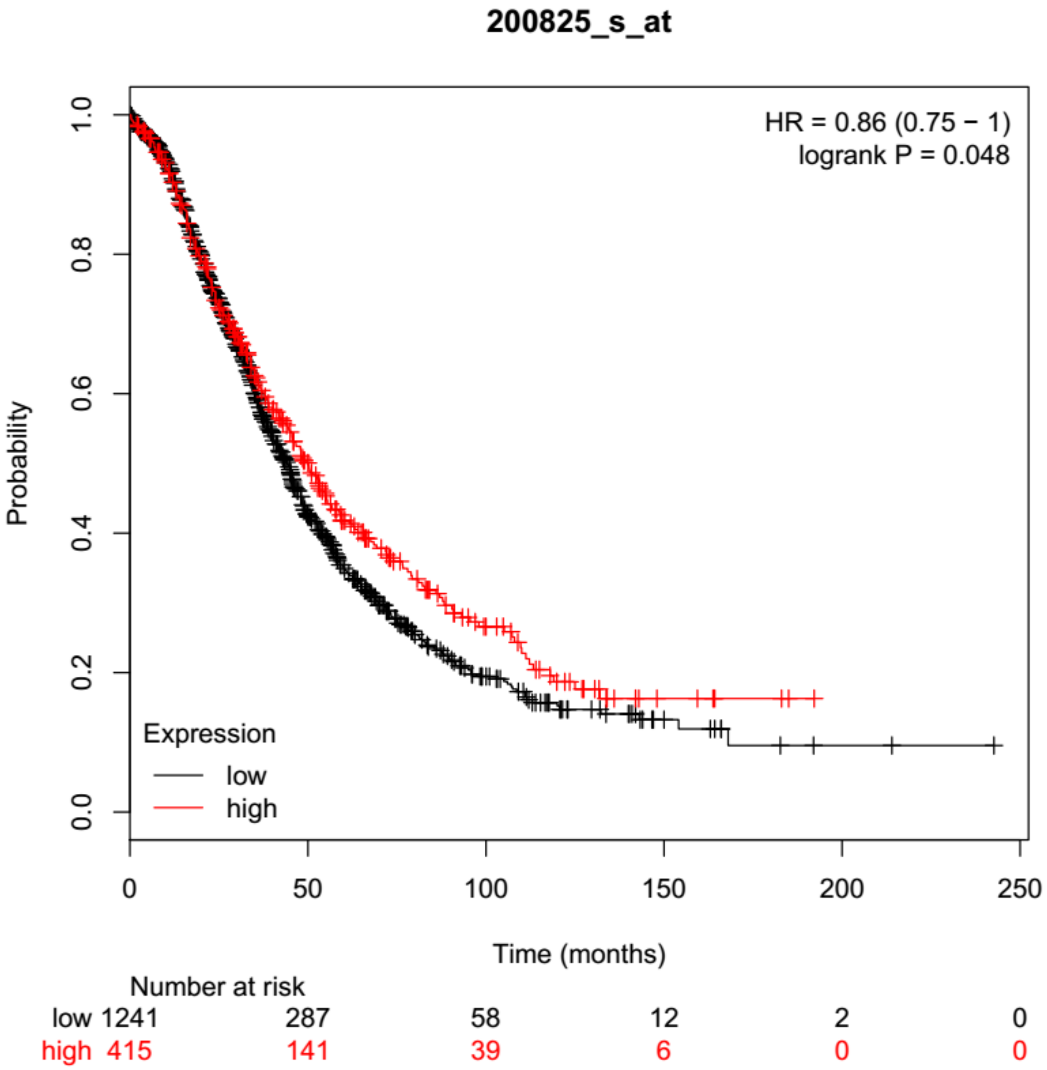
200825_s_at
HR = 0.86 (0.75 − 1)
logrank P = 0.048
Probability
0.0
0.2
0.4
0.6
0.8
1.0
Expression
low
high
0
50
100
150
200
250
Time (months)
Number at risk
low 1241 287 58 12 2 0
high 415 141 39 6 0 0

34. MRPL41

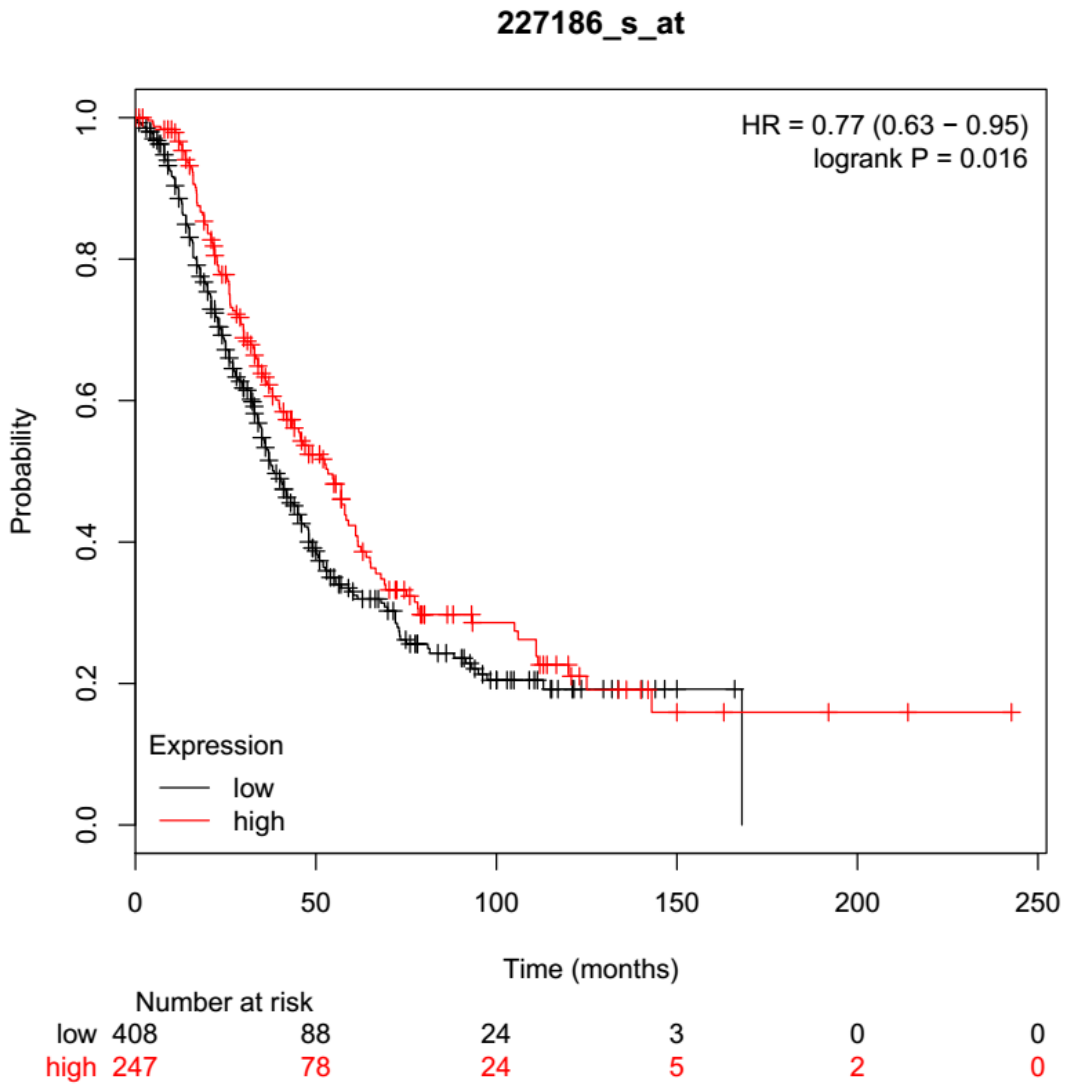
227186_s_at
HR = 0.77 (0.63 − 0.95)
logrank P = 0.016
Probability
0.0
0.2
0.4
0.6
0.8
1.0
Expression
low
high
0
50
100
150
200
250
Time (months)
Number at risk
low 408 88 24 3 0 0
high 247 78 24 5 2 0

35. MRPL46

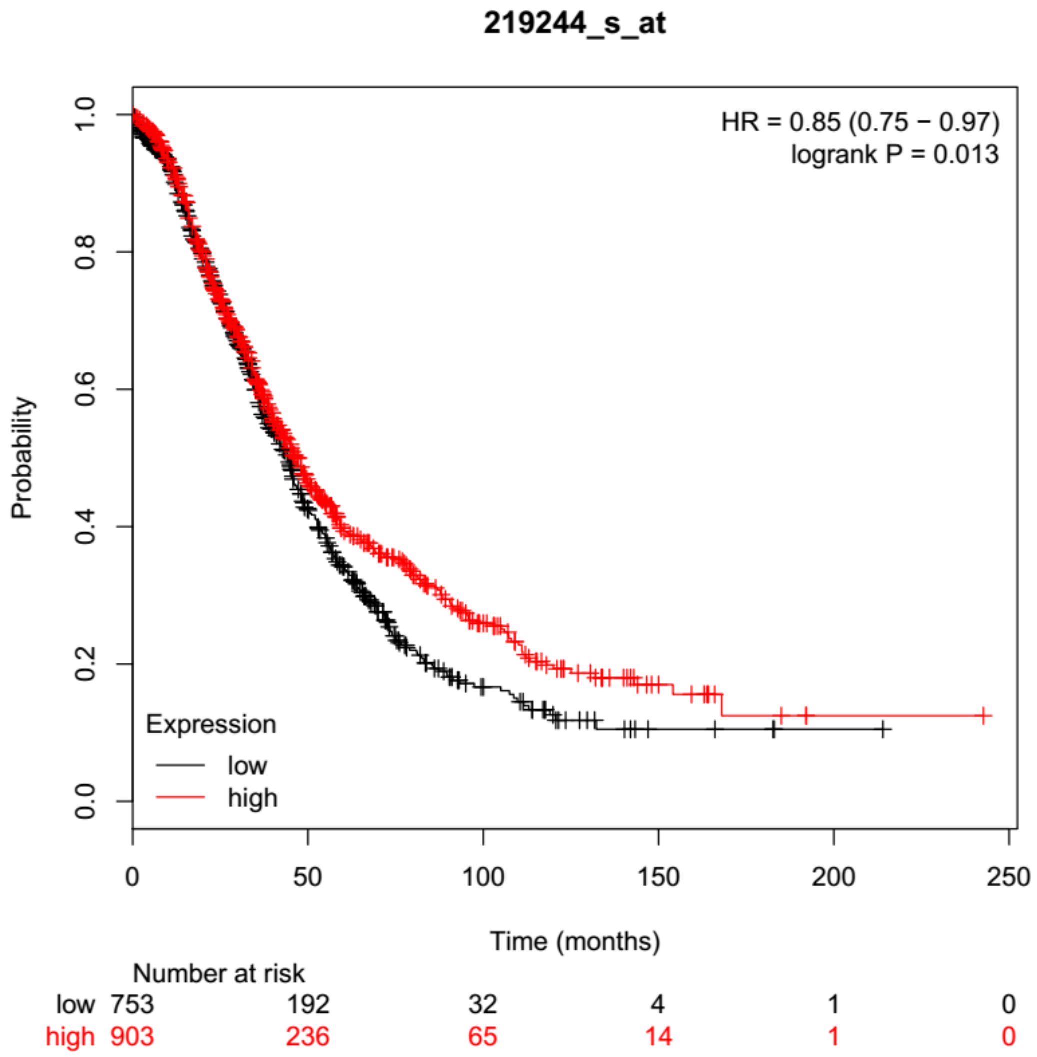

219244_s_at
HR = 0.85 (0.75 − 0.97)
logrank P = 0.013
Probability
0.0
0.2
0.4
0.6
0.8
1.0
Expression
low
high
0
50
100
150
200
250
Time (months)
Number at risk
low 753 192 32 4 1 0
high 903 236 65 14 1 0

36. MRPL53

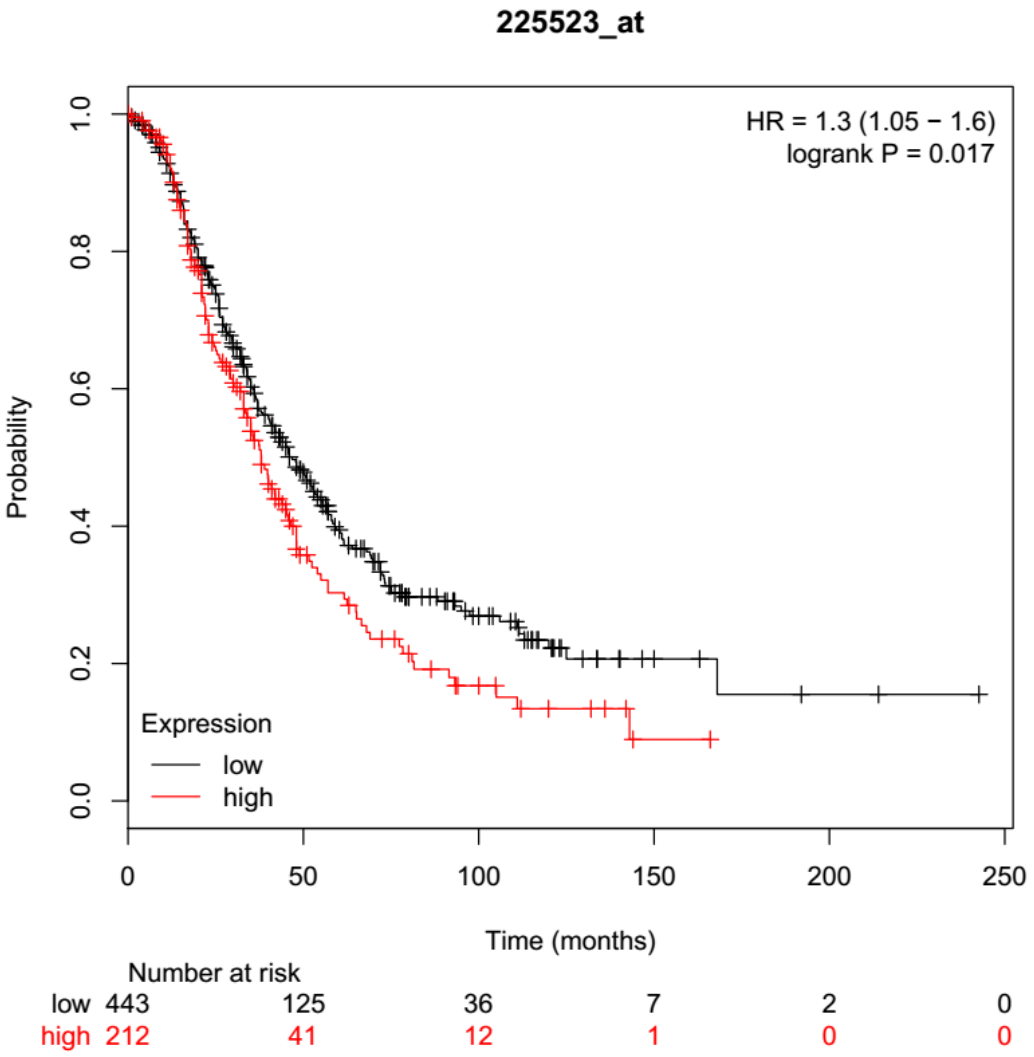

225523_at
HR = 1.3 (1.05 − 1.6)
logrank P = 0.017
Probability
1.0
0.8
0.6
0.4
0.2
0.0
Expression
low
high
0
50
100
150
200
250
Time (months)
Number at risk
low 443 125 36 7 2 0
high 212 41 12 1 0 0

37. MRPL54

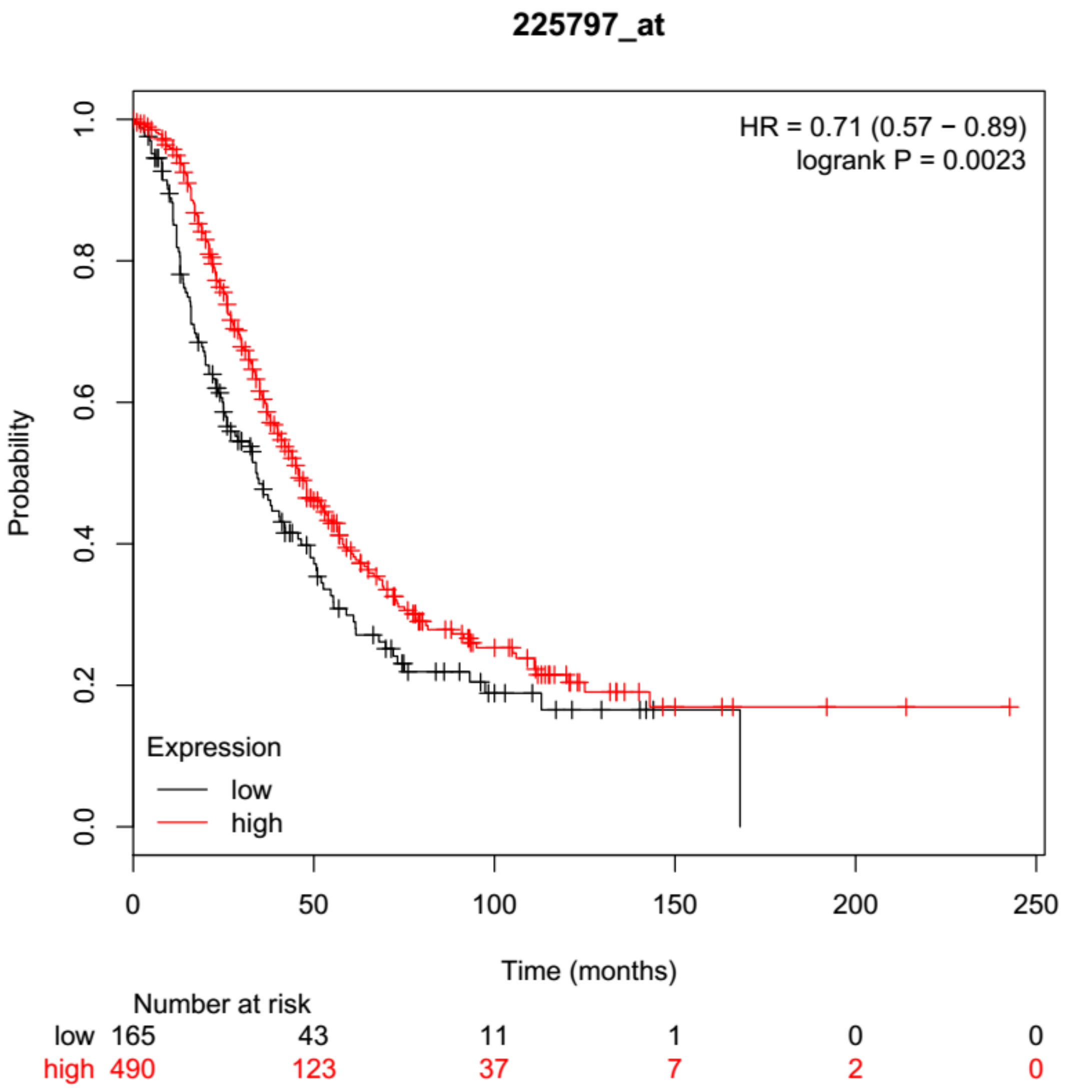
225797_at
HR = 0.71 (0.57 − 0.89)
logrank P = 0.0023
Probability
0.0
0.2
0.4
0.6
0.8
1.0
Expression
low
high
0
50
100
150
200
250
Time (months)
Number at risk
low 165 43 11 1 0 0
high 490 123 37 7 2 0

38. MRPS9

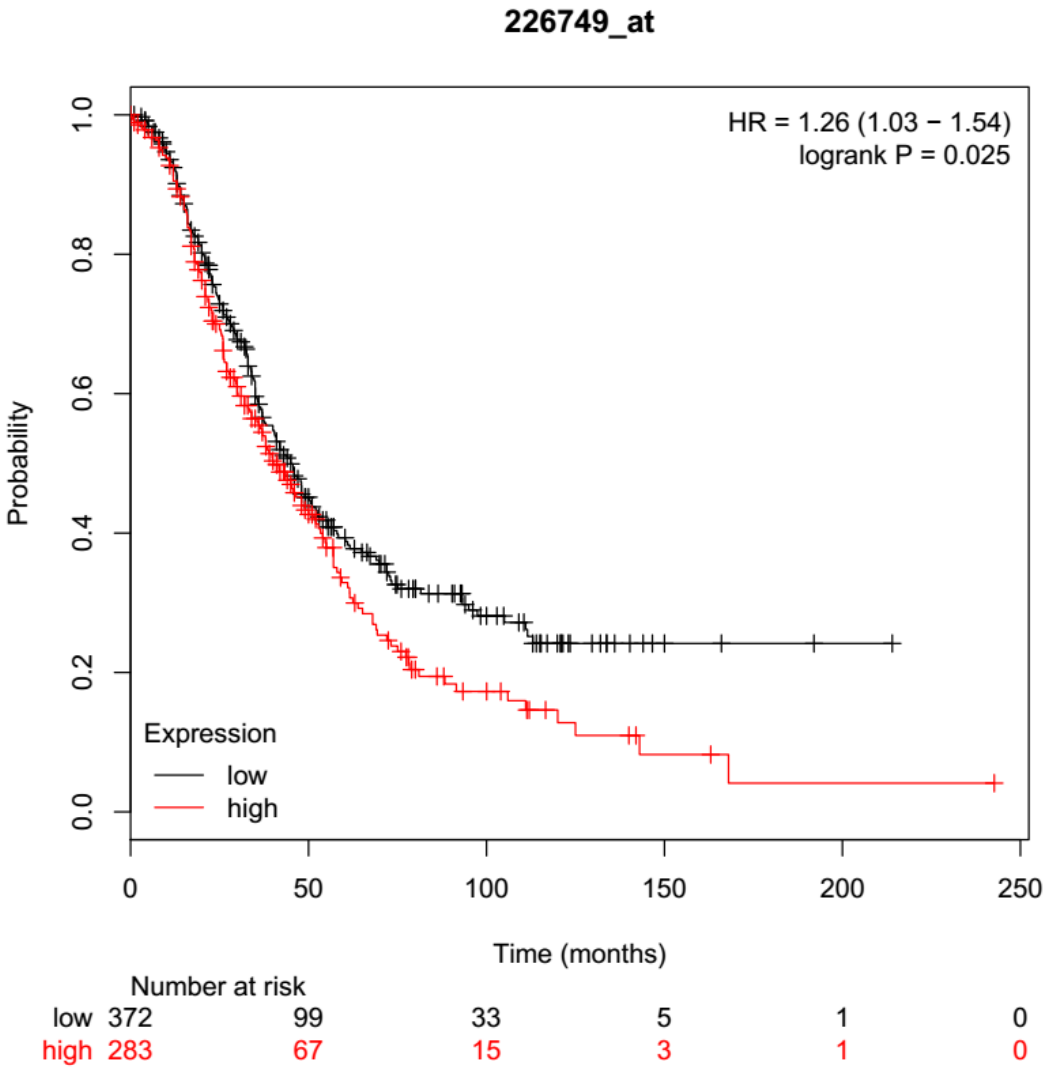

226749_at
HR = 1.26 (1.03 − 1.54)
logrank P = 0.025
Probability
0.0
0.2
0.4
0.6
0.8
1.0
Expression
low
high
0
50
100
150
200
250
Time (months)
Number at risk
low 372 99 33 5 1 0
high 283 67 15 3 1 0

39. MRPS12

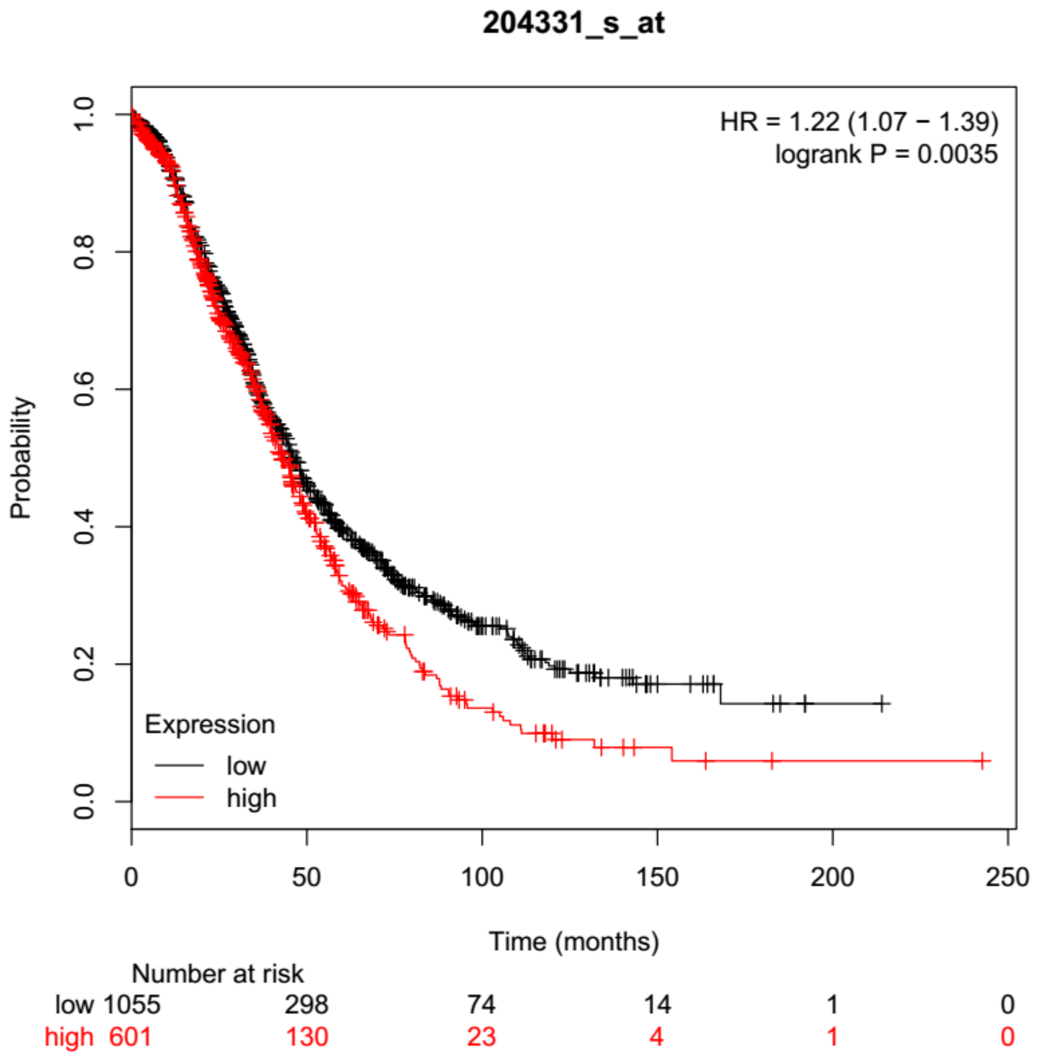

204331_s_at
HR = 1.22 (1.07 − 1.39)
logrank P = 0.0035
Probability
0.0
0.2
0.4
0.6
0.8
1.0
Expression
low
high
0
50
100
150
200
250
Time (months)
Number at risk
low 1055 298 74 14 1 0
high 601 130 23 4 1 0

40. MRPS15

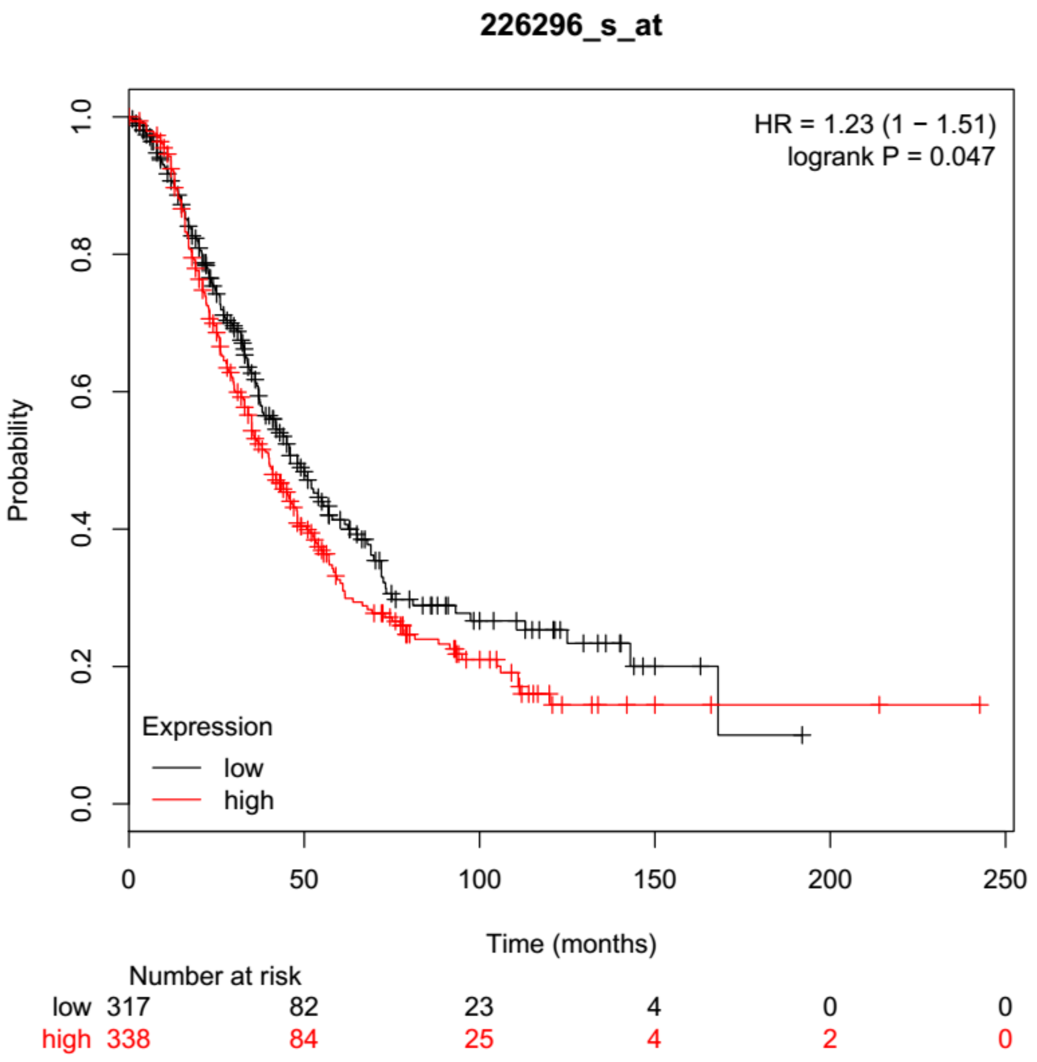
226296_s_at
HR = 1.23 (1 − 1.51)
logrank P = 0.047
Probability
0.0
0.2
0.4
0.6
0.8
1.0
Expression
low
high
0
50
100
150
200
250
Time (months)
Number at risk
low 317 82 23 4 0 0
high 338 84 25 4 2 0

41. OGN

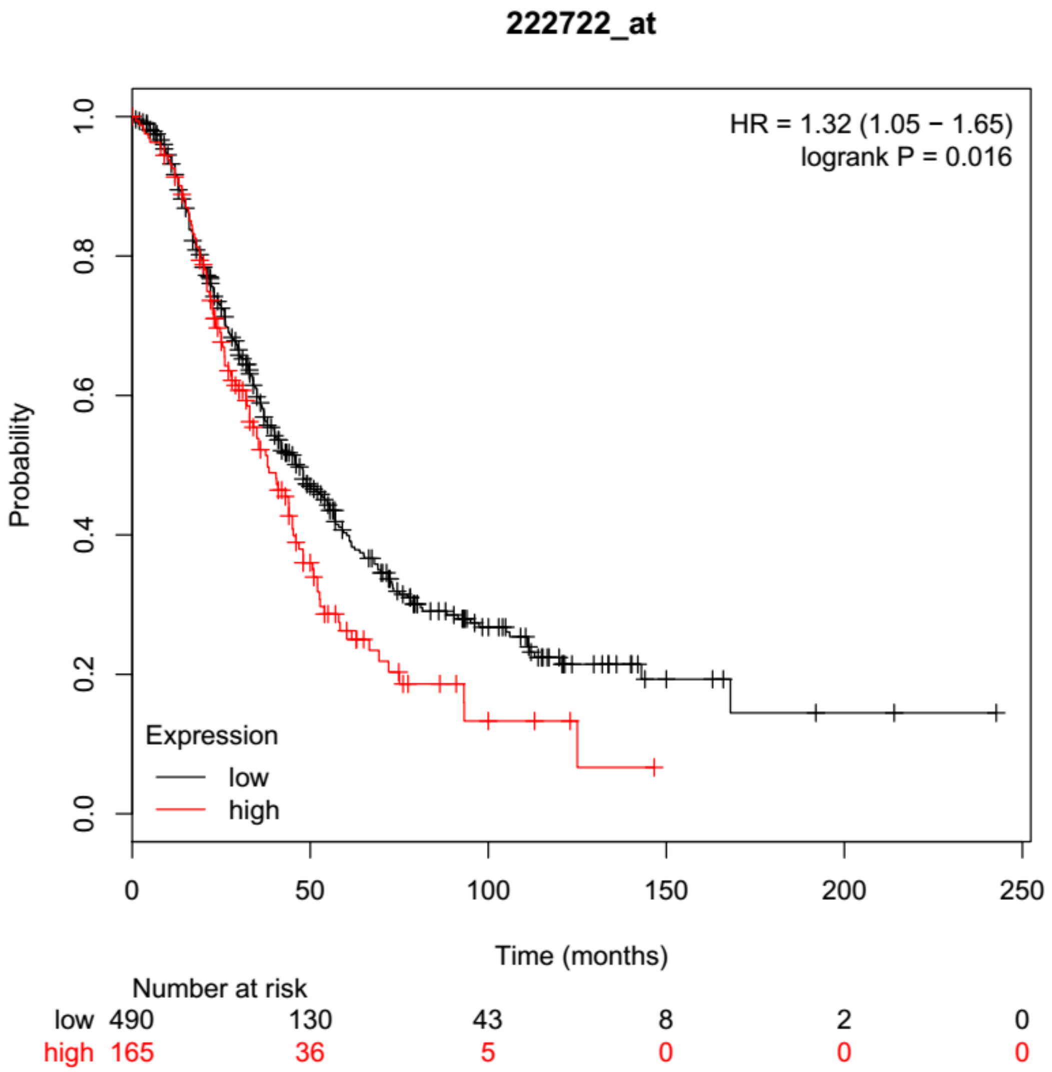

222722_at
HR = 1.32 (1.05 − 1.65)
logrank P = 0.016
Probability
0.0
0.2
0.4
0.6
0.8
1.0
Expression
low
high
0
50
100
150
200
250
Time (months)
Number at risk
low 490 130 43 8 2 0
high 165 36 5 0 0 0

42. PDIA5

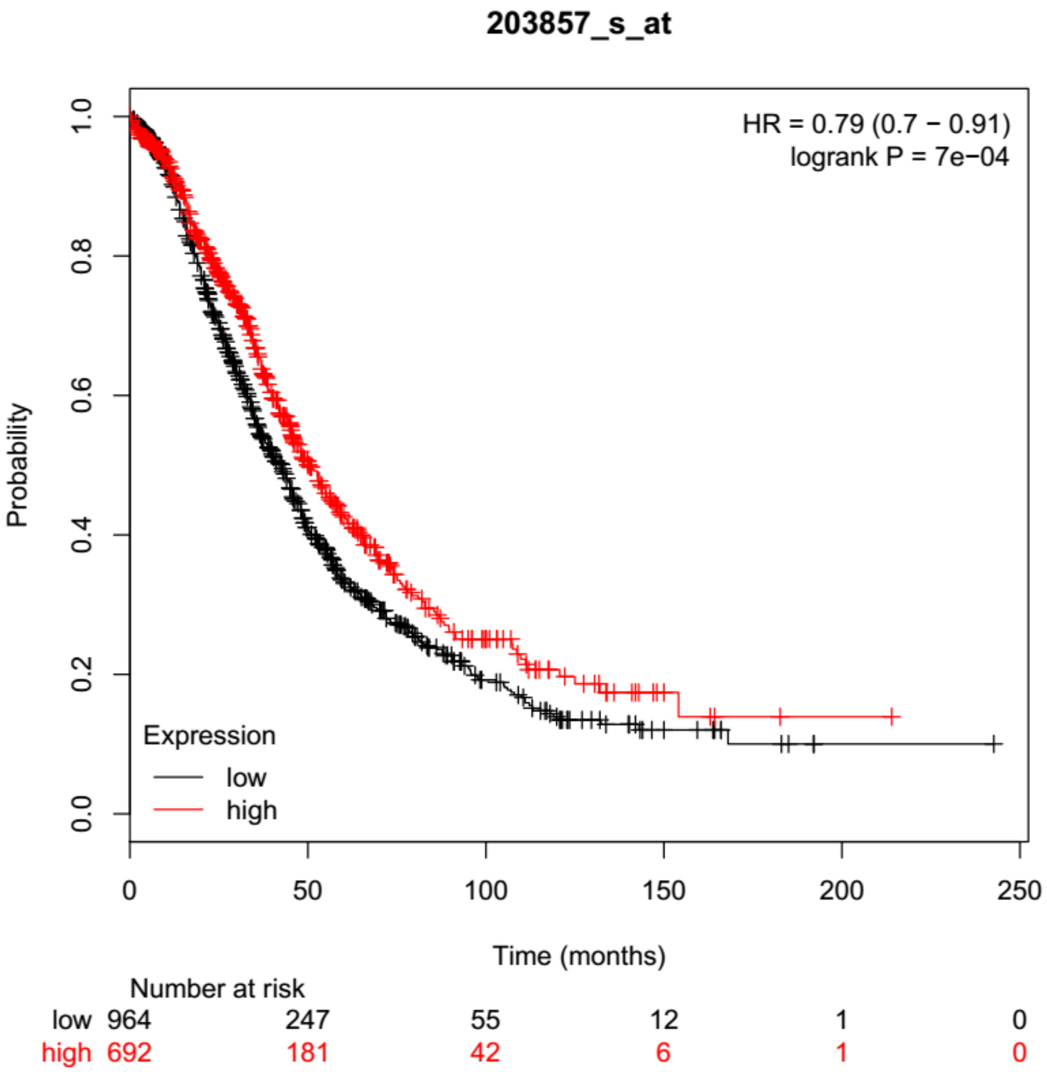
203857_s_at
HR = 0.79 (0.7 − 0.91)
logrank P = 7e−04
Probability
0.0
0.2
0.4
0.6
0.8
1.0
Expression
low
high
0
50
100
150
200
250
Time (months)
Number at risk
low 964 247 55 12 1 0
high 692 181 42 6 1 0

43. PECR

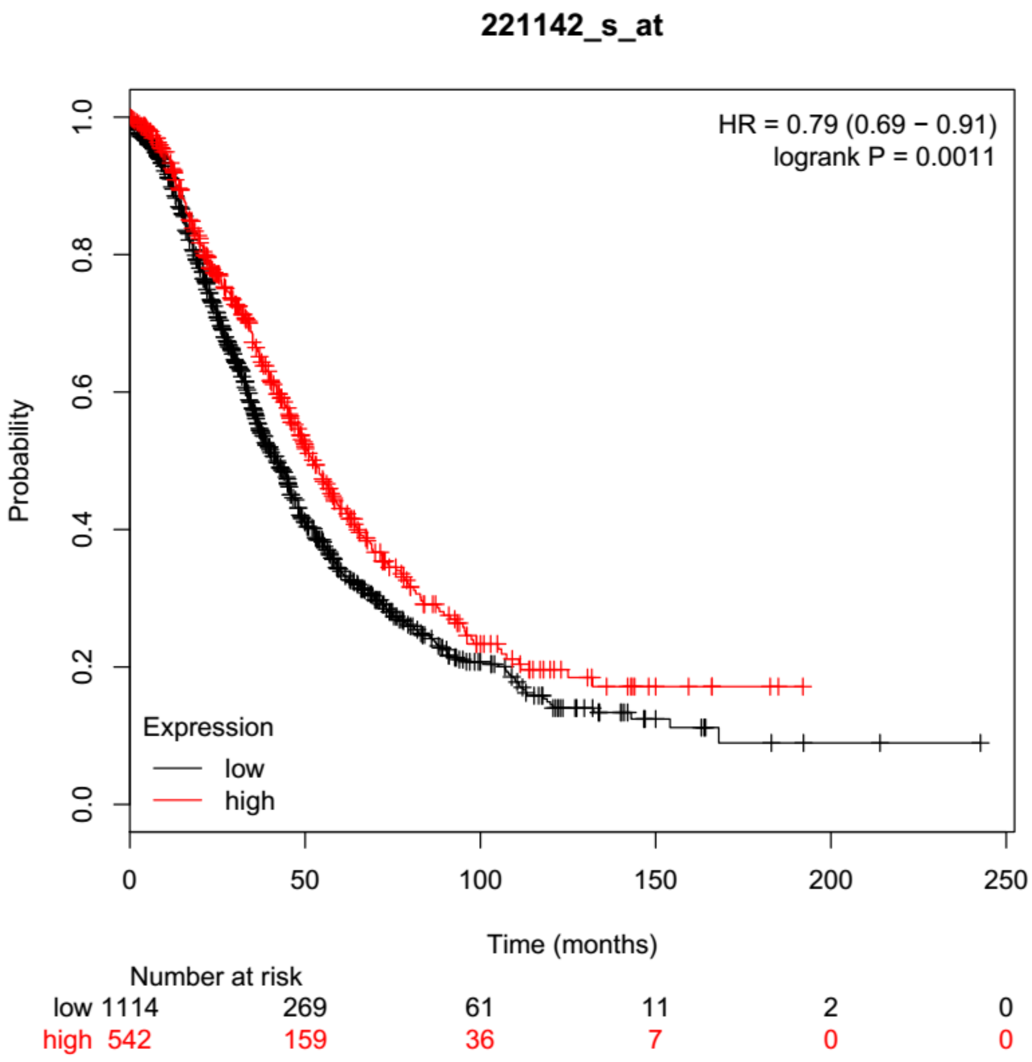

221142_s_at
HR = 0.79 (0.69 − 0.91)
logrank P = 0.0011
Probability
1.0
0.8
0.6
0.4
0.2
0.0
Expression
low
high
0
50
100
150
200
250
Time (months)
Number at risk
low 1114 269 61 11 2 0
high 542 159 36 7 0 0

44. PRELP

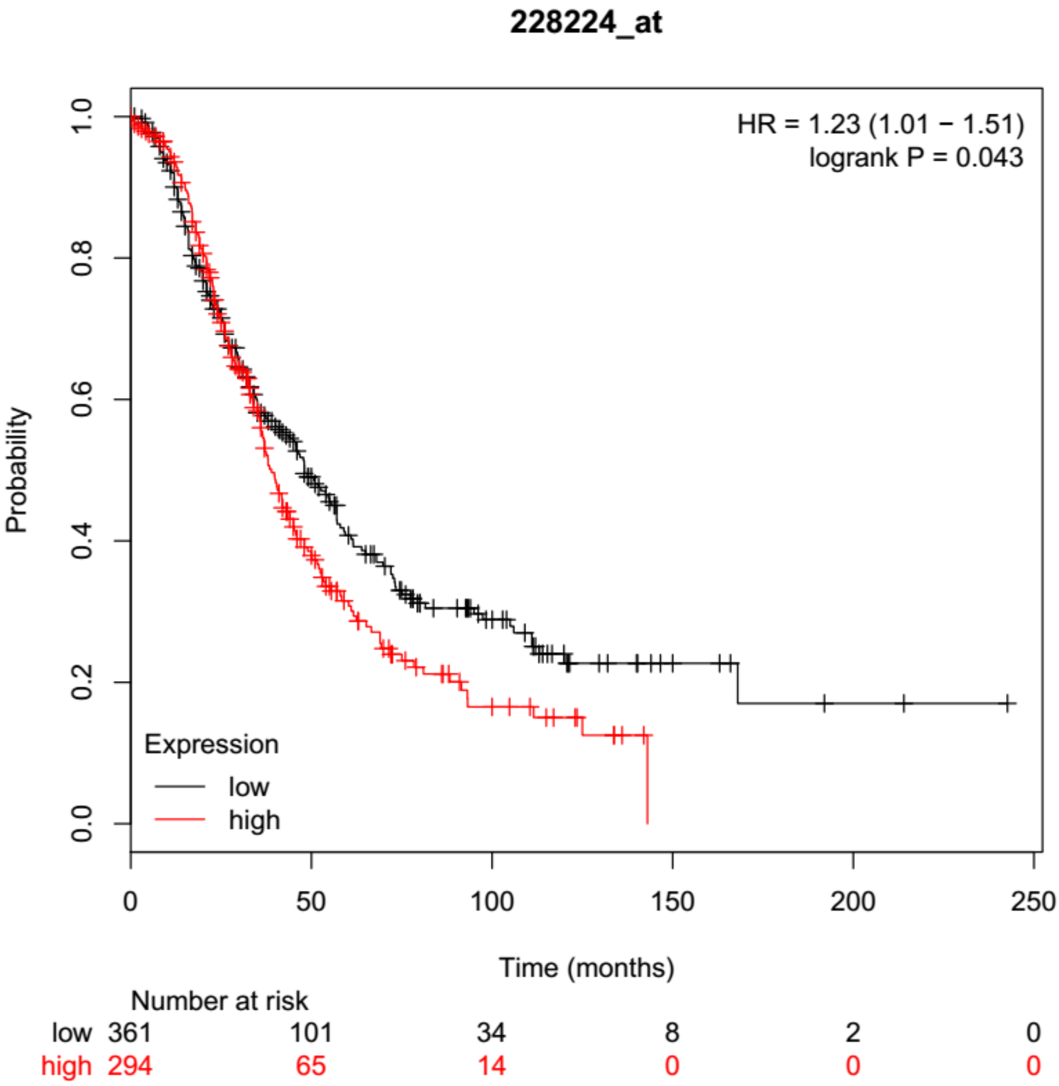

228224_at
HR = 1.23 (1.01 − 1.51)
logrank P = 0.043
Probability
0.0
0.2
0.4
0.6
0.8
1.0
Expression
low
high
0
50
100
150
200
250
Time (months)
Number at risk
low 361 101 34 8 2 0
high 294 65 14 0 0 0

45. RDH10

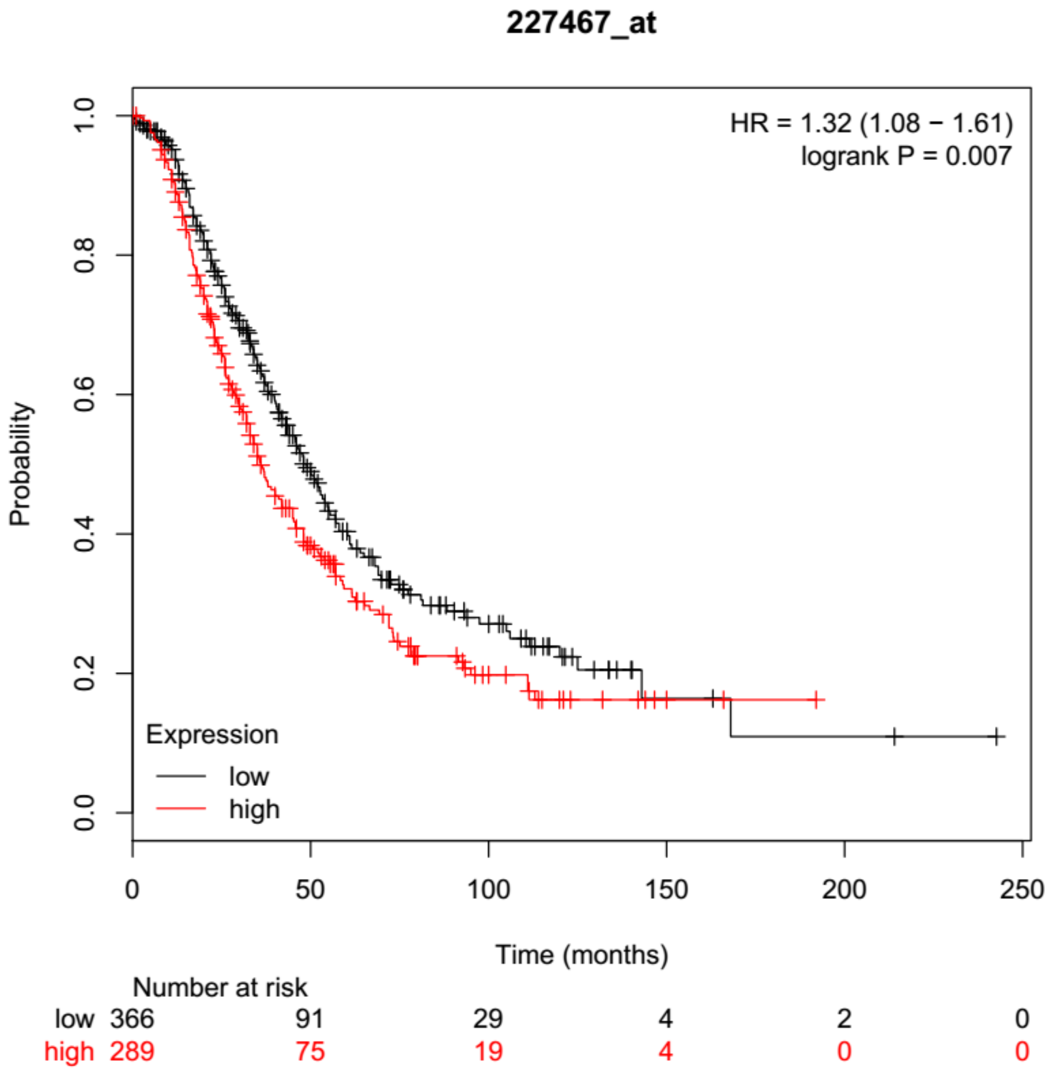
227467_at
HR = 1.32 (1.08 − 1.61)
logrank P = 0.007
Probability
0.0
0.2
0.4
0.6
0.8
1.0
Expression
low
high
0
50
100
150
200
250
Time (months)
Number at risk
low 366 91 29 4 2 0
high 289 75 19 4 0 0

46. RPS3

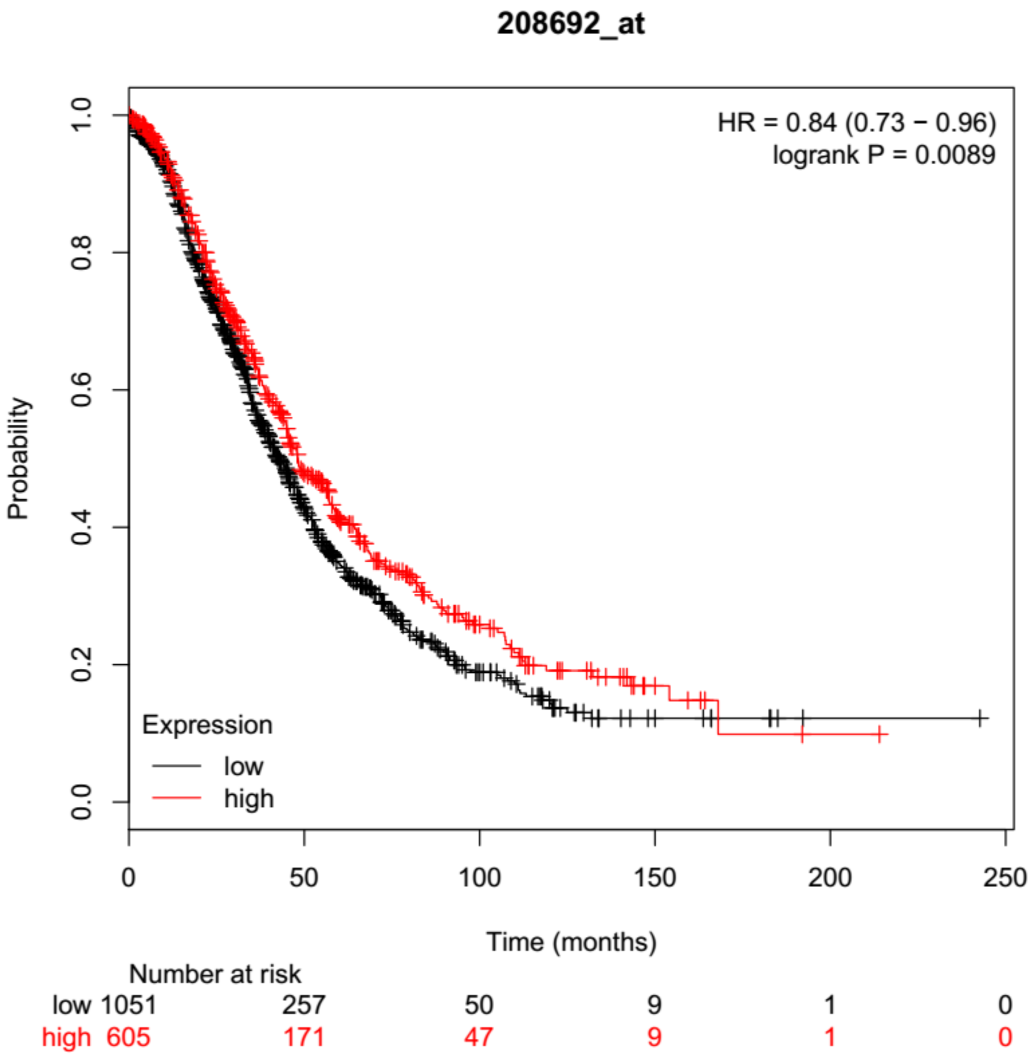
208692_at
HR = 0.84 (0.73 − 0.96)
logrank P = 0.0089
Probability
0.0
0.2
0.4
0.6
0.8
1.0
Expression
low
high
0
50
100
150
200
250
Time (months)
Number at risk
low 1051 257 50 9 1 0
high 605 171 47 9 1 0

47. RRAS

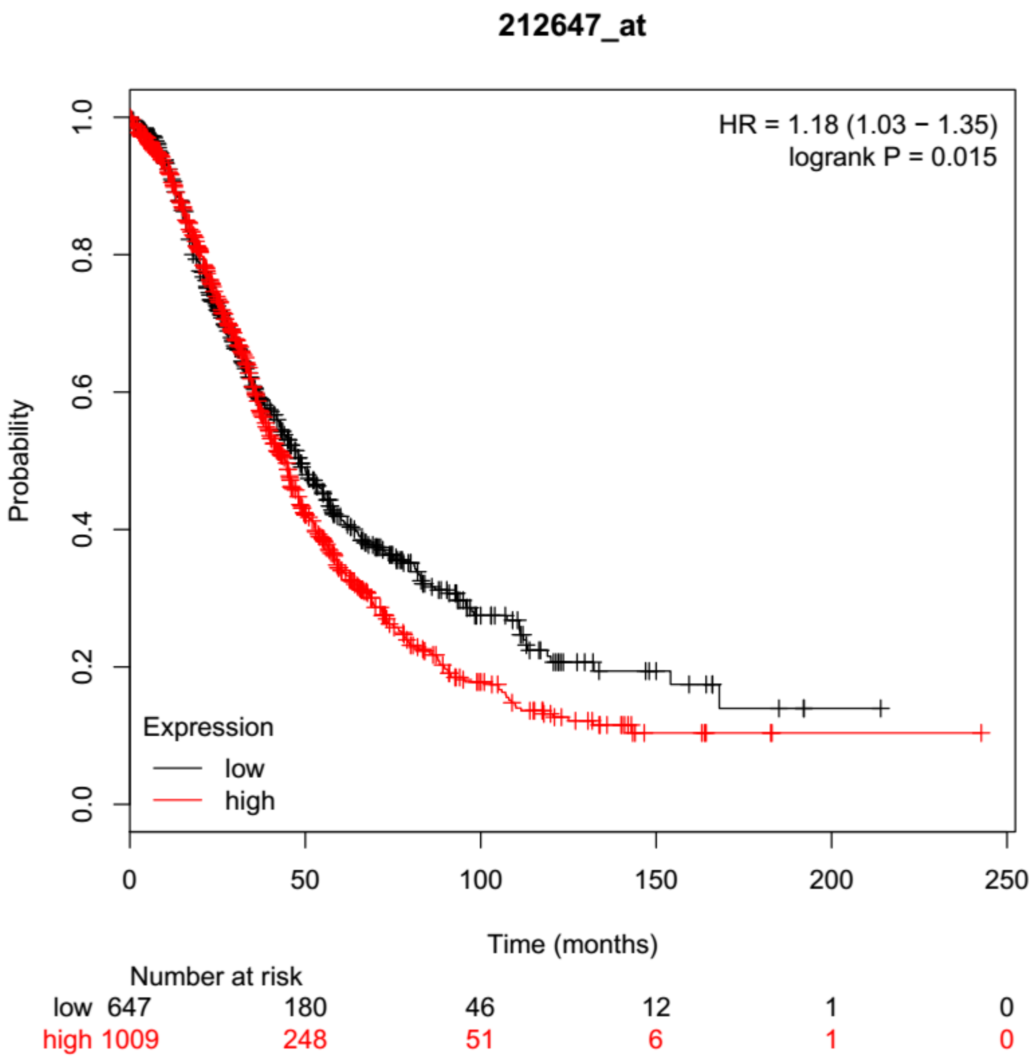
212647_at
HR = 1.18 (1.03 − 1.35)
logrank P = 0.015
Probability
1.0
0.8
0.6
0.4
0.2
0.0
Expression
low
high
0
50
100
150
200
250
Time (months)
Number at risk
low 647 180 46 12 1 0
high 1009 248 51 6 1 0

48. RRAS2

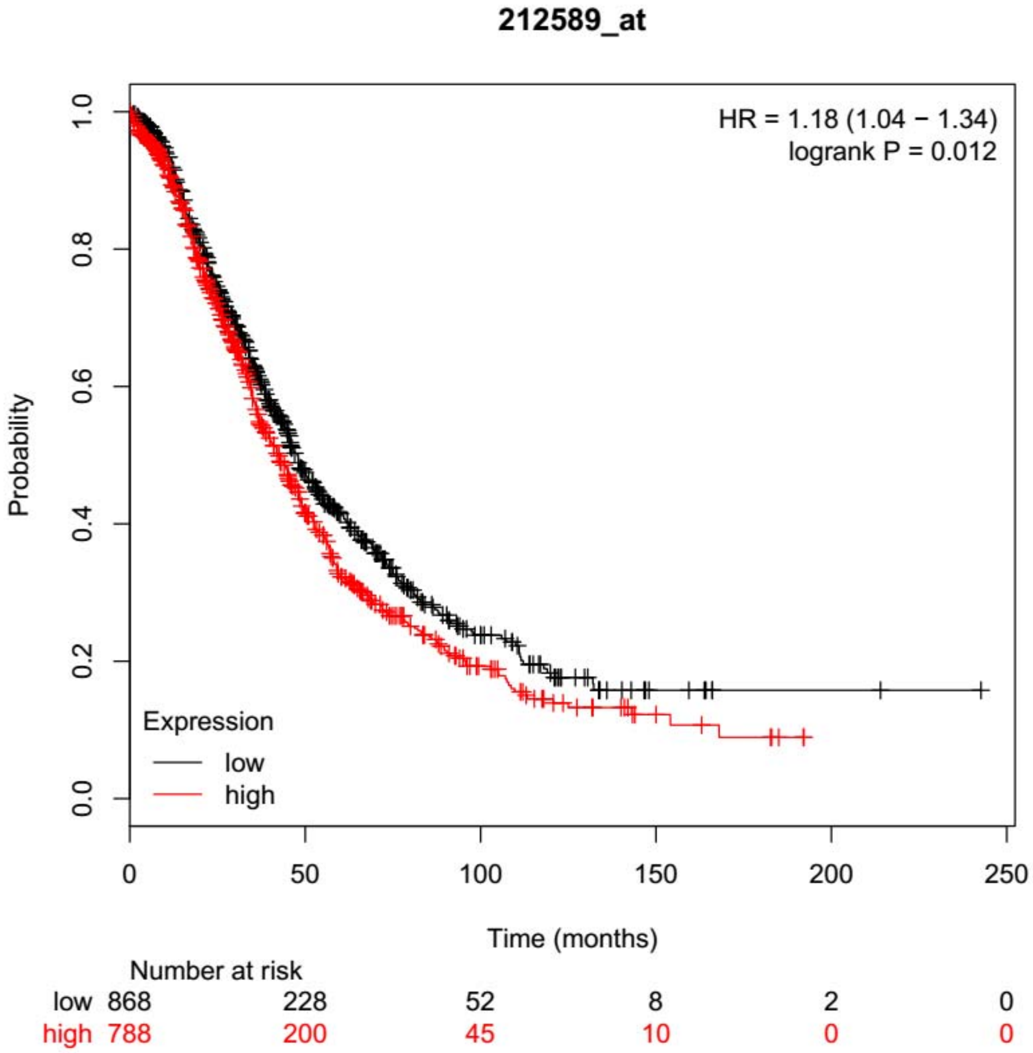
212589_at
HR = 1.18 (1.04 − 1.34)
logrank P = 0.012
Probability
0.0
0.2
0.4
0.6
0.8
1.0
Expression
low
high
0
50
100
150
200
250
Time (months)
Number at risk
low 868 228 52 8 2 0
high 788 200 45 10 0 0

49. SRPRB

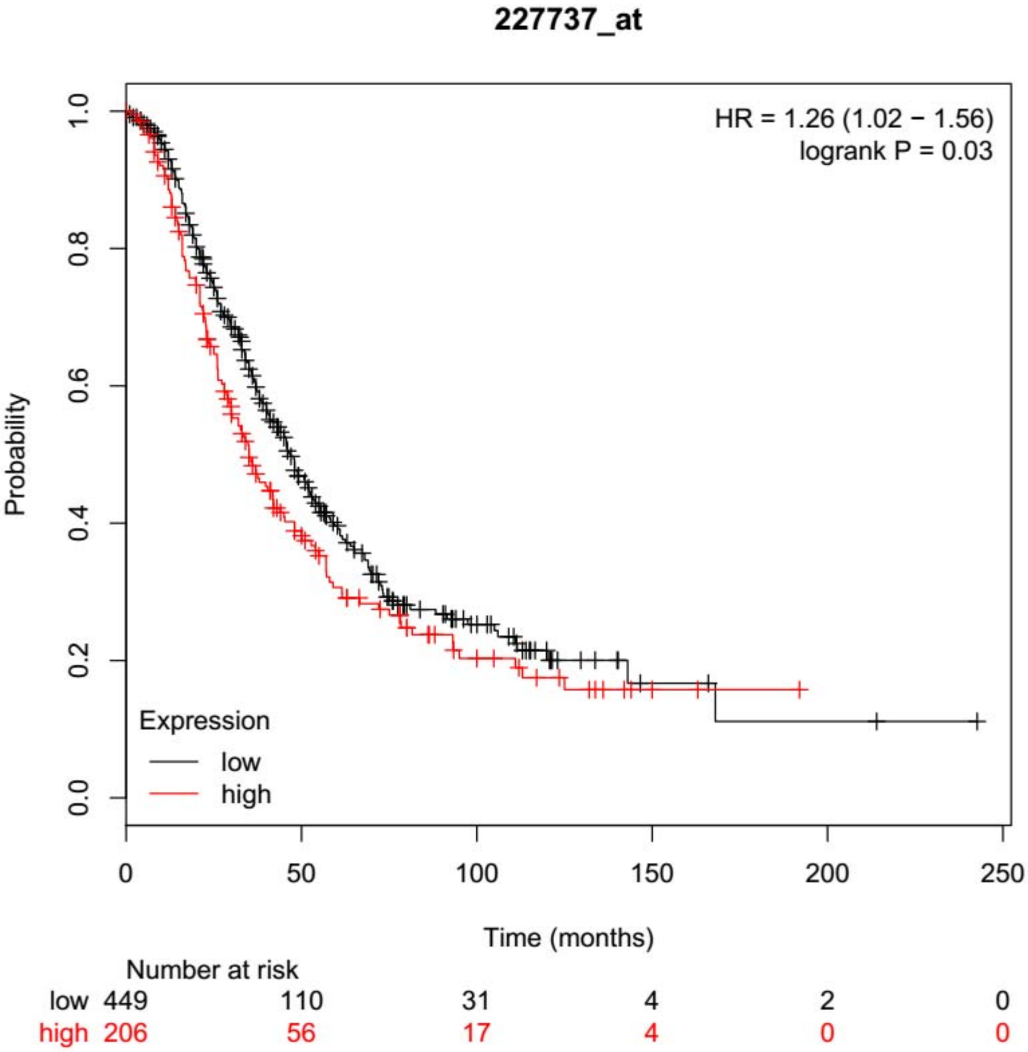
227737_at
HR = 1.26 (1.02 − 1.56)
logrank P = 0.03
Probability
1.0
0.8
0.6
0.4
0.2
0.0
Expression
low
high
0
50
100
150
200
250
Time (months)
Number at risk
low 449 110 31 4 2 0
high 206 56 17 4 0 0

50. TXNDC12

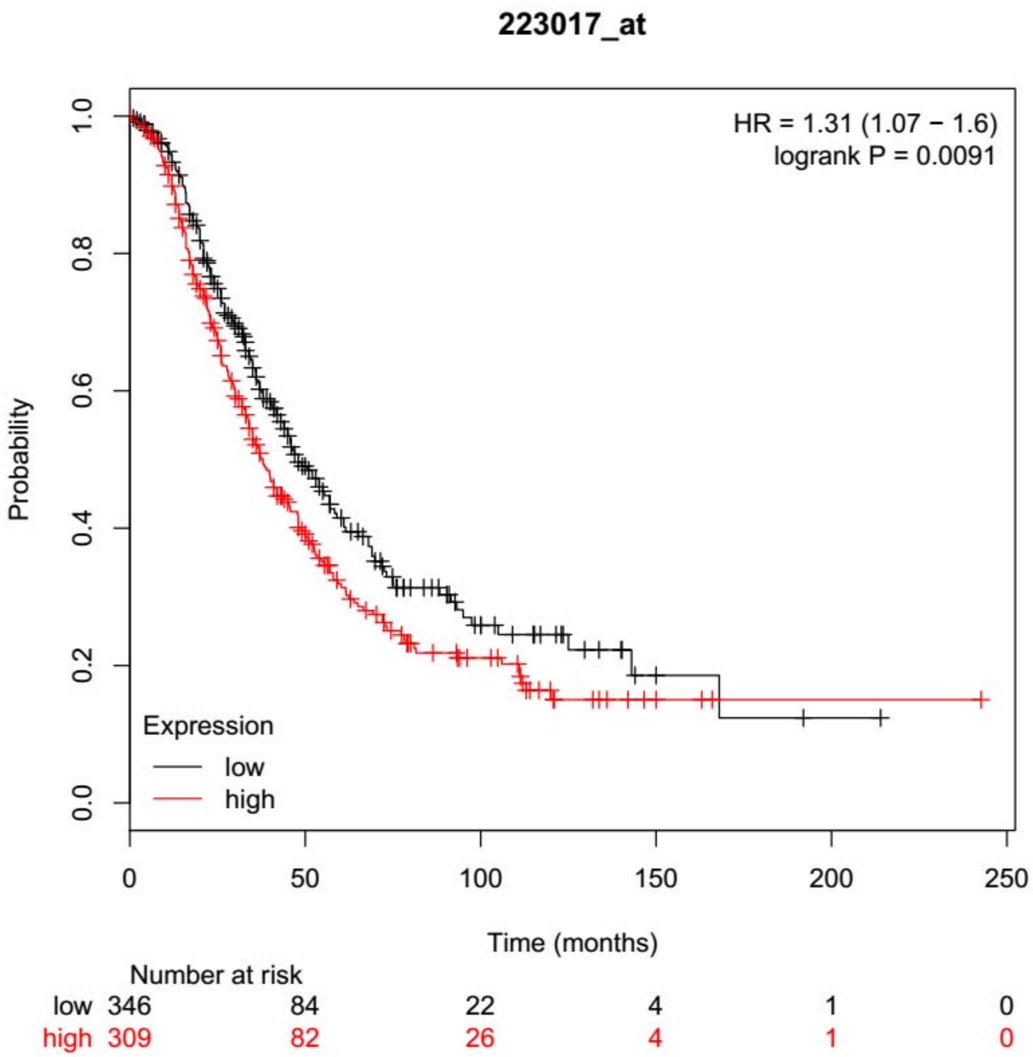
223017_at
HR = 1.31 (1.07 − 1.6)
logrank P = 0.0091
Probability
0.0
0.2
0.4
0.6
0.8
1.0
Expression
low
high
0
50
100
150
200
250
Time (months)
Number at risk
low 346 84 22 4 1 0
high 309 82 26 4 1 0
